# Acidosis-induced p38-kinase activation triggers an IL-6-mediated crosstalk of renal proximal tubule cells with fibroblasts leading to their inflammatory response

**DOI:** 10.1186/s12964-025-02180-5

**Published:** 2025-04-11

**Authors:** Marie-Christin Schulz, Nathalie Wolff, Michael Kopf, Micheal Gekle

**Affiliations:** Julius-Bernstein-Institut für Physiologie, Universität Halle- Wittenberg, Magdeburger Straße 6, 06112 Halle (Saale), Germany

**Keywords:** Cellular crosstalk, Chronic kidney diseases, Local interstitial acidosis, Local inflammation, IL-6 effects, p38-signaling

## Abstract

**Background:**

Local interstitial acidosis in chronic kidney disease (CKD) induces inflammatory responses and dedifferentiation of proximal tubule cells (PTCs), disrupting cellular crosstalk through cytokine and COX-2 metabolite secretion. This promotes a switch to an inflammatory fibroblast phenotype, further exacerbating inflammation and PTC dedifferentiation. p38-signaling and downstream transcription factors, including P-CREB and c-fos, contribute to these responses. This study investigates the impact of acidosis on inflammatory responses in PTCs and fibroblasts, focusing on cellular crosstalk and the role of p38-signaling.

**Methods:**

HK-2 (human PTCs) and CCD-1092Sk (human fibroblasts) were exposed to acidic or control media in mono- and coculture for 30 min, 3 h, or 48 h. Protein expression of IL-6, phosphorylated (P-) and total CREB, P- and total SRF, c-fos, and P- and total p38 was analyzed by western blot. IL-6 secretion was measured using ELISA. The impact of p38 and IL-6 receptor activity was assessed by pharmacological intervention.

**Results:**

In coculture, acidosis initially caused a transient decrease in IL-6 secretion but significantly increased IL-6 levels after 48 h. Acidosis induced intracellular IL-6 expression in HK-2 cells within 3 h independent of culture conditions, with sustained IL-6 protein increase after 48 h only in coculture. Acidosis also enhanced P-CREB and c-fos expression in coculture during the first 3 h. Regardless of culture conditions, acidosis increased IL-6, c-fos, and P-SRF expression in CCDSK cells after 48 h. P-CREB and COX-2 expression were elevated in CCDSK in coculture. Acidosis-mediated effects on IL-6, P-CREB, and P-SRF expression were p38-dependent in both cell lines. Finally, we assessed the pH-dependency of IL-6 action and found that IL-6 addition increased COX-2 expression via the IL-6 receptor in acidic but not control media. Thus, acidosis enhances IL-6 secretion and potentiates its receptor-mediated biological effects.

**Conclusion:**

This study identifies IL-6 as a key mediator of tubule-fibroblast crosstalk in an acidic milieu, promoting inflammatory processes. Acidosis induces IL-6 expression, secretion, and biological effects, with p38 kinase as a crucial mediator. If validated in vivo, these findings could enhance understanding of CKD and support early interventions.

**Supplementary Information:**

The online version contains supplementary material available at 10.1186/s12964-025-02180-5.

## Graphical Abstract

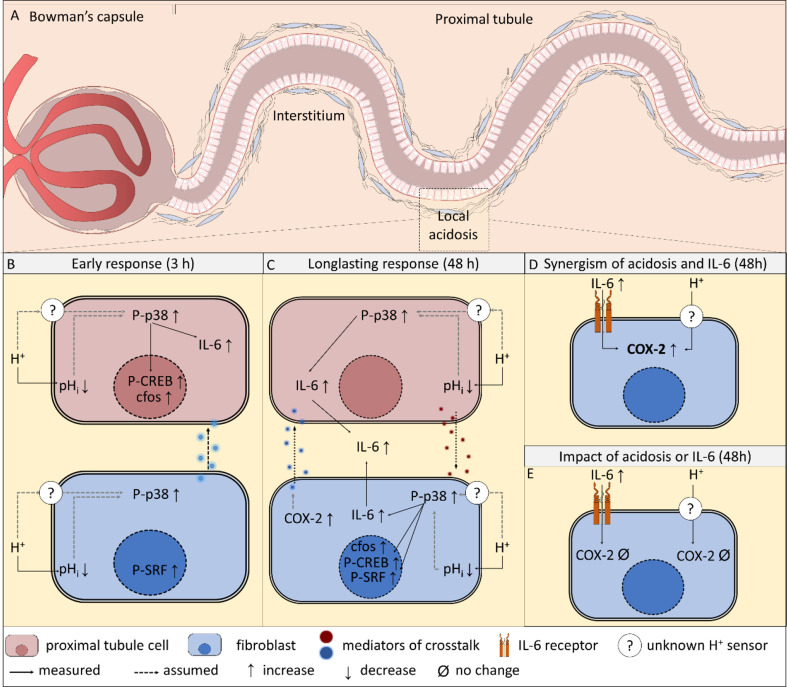

## Introduction

According to Francis et al. chronic kidney diseases (CKD) belong to the top 10 causes of death [1]. CKD is typically diagnosed at advanced stages, when irreversible loss of function has occurred. With no causal treatments available, current options are limited to burdensome procedures like dialysis or transplantation. Understanding the early mechanisms of CKD is therefore crucial for developing new therapies that could reduce the need for renal replacement and associated fatalities [2]. It has been described that in injured proximal tubular cells, fatty acid oxidation is reduced in favor of anaerobic glycolysis. As a consequence, lactic acid accumulates in the tubulointerstitial space, leading to local acidosis [3]. In addition to the increased glycolysis rate an enhanced expression of lactate dyhydrogenase is described, exacerbating the accumulation of lactic acid [4]. Beside the metabolic switch, mitochondrial dysfunction in tubule cells is common during the development of CKD and provides another source of lactic acid [5]. Beside the accumulation of lactic acid a decreased tubular net production of bicarbonate can also contribute to local acidification [6]. A further mechanism which supports the development of local acidosis is an impaired bicarbonate resorption e.g. by a changed expression of the sodium hydrogen exchanger (NHE3) [7]. Beside proximal tubule cells, other cell types like fibroblasts or migrating leucocytes can contribute to the development of acidosis in the tubule interstitial-space. Especially in the early phase of CKD, dyshomeostasis of the tubulointerstitial space, comprising inflammation with dysregulated crosstalk between proximal tubule cells (PTC) and fibroblasts play a major role [8]. There is evidence that acidosis increases the concentration of angiotensin II in the kidney. As a result, the expression of proton transporters such as NHE and proton ATPase is increased, thereby temporarily enhancing net proton excretion. In the long term, however, elevated angiotensin II levels trigger the development of inflammation and tubular atrophy [9]. Stromal fibroblasts are described as key drivers for local inflammation in chronical diseases like CKD due to the secretion of cytokines, like IL-6 [10, 11],. Additionally, data from a previous study by our group unveiled that a switch to an inflammatory phenotype can be triggered by acidosis in combination with the crosstalk of proximal tubule cells and fibroblasts [12]. It is known that elevated IL-6 blood concentration correlates negatively with kidney function [13]. It is also stated that IL-6 signaling participates in the pathogenesis and progression of diabetic nephropathy [14]. Due to its importance for kidney diseases, one aim of the present study is to identify IL-6 as a possible mediator of the cellular crosstalk in an acidic milieu. Additionally, our previous study showed that extracellular acidosis triggers the fibroblast phenotype switch via the p38-pathway [12]. MAPKs, such as p38, mediate the effects of extracellular stimuli on cellular responses by phosphorylating downstream signaling molecules, such as transcription factors e.g. NF-κB or, P-CREB or AP-1 [15–17]. These transcription factors regulate the expression of target genes like cytokines or signaling molecules and induce cellular response like inflammation or dedifferentiation [18]. However, a knowledge gap remains between acidosis-induced p38-signaling and the observed phenotype switch, which this study aims to partially close. The transcription factors CREB and SRF were assessed in the present study because they fulfil the following criteria: (I) they can be phosphorylated by p38 (II) they are inducible by acidosis and (III) they induce the expression of IL-6 [19–24].

CREB and SRF initiate the transcription of IL-6 indirectly, by inducing the expression of further transcription factors like cfos [25, 26]. The activity of signaling molecules such as MAPK, SRF, or CREB can fluctuate over time. Different temporal modes of operation are known, which allow demand-driven regulation of signaling pathways. The rapid, transient activation mode enables acute response and the sustained activation mode mediates long-lasting effects [27–31]. To account for these modes of operation, the acidosis effect was measured at different time points. The renal pH landscape is highly complex thus, it is difficult to establish clear thresholds for pathophysiological and physiological conditions. Both physiological and pathophysiological pH values in the kidney cover a wide range [32, 33]. Under pathophysiological conditions (inflammation, ischemia, hypoxia) tissue pH-values decrease well below pH 6.8 [34, 35]. Therefore, we have chosen a pH value of 7.4 as an example for the physiological range and a pH value of 6.4 as an example for the pathophysiological range.

Aim of our study was to investigate the role of IL-6 as a possible mediator of the cellular crosstalk in an acidic milieu in a coculture model of a human proximal tubule cell line (HK-2) and human fibroblasts (CCD-1092Sk). Furthermore, the impact of acidosis in coculture on CREB, SRF and cfos expression was determined, to verify them as possible mediators between the p38-signaling and the inflammatory outcome. Finally, the role of p38-activity was assessed.

## Materials and methods

If not stated otherwise, chemicals were purchased from Sigma-Aldrich, Munich, Germany.

### Cell culture

Normal human kidney epithelial cells (HK-2, CRL-2190™) and normal human fibroblasts (CCD-1092Sk, ATCC^®^ CRL-2114™) were grown in DMEM medium supplemented with 10% fetal calf serum (FCS) and 2 g/l NaHCO_3_ at 37 °C under a humidified 5% CO_2_ atmosphere and subcultivated once per week before confluence.

### Experimental setup

**Fig. 1:**
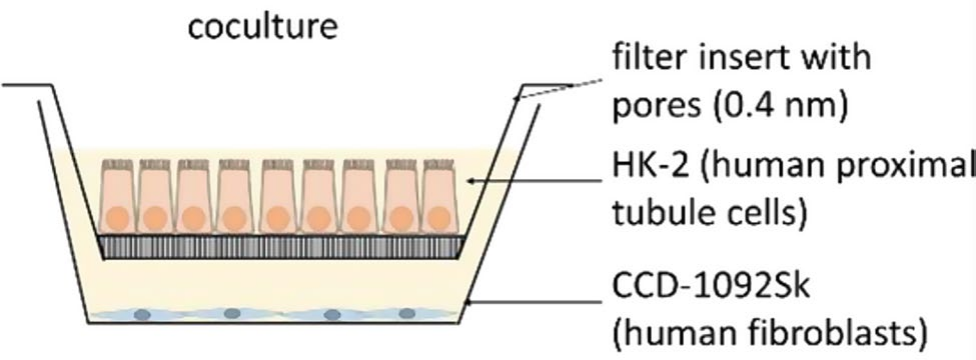
Scheme of coculture, created with filter inserts

HK-2 cells were grown on permeable filter inserts (pore size 0.4 μm), and CCD-1092Sk cells were grown in 6-well plates. When cells were confluent on the filter inserts, containing HK-2 cells, they were transferred to the 6-well plates, containing CCD-1092Sk cells (Fig. 1). This indirect coculture was used for the following experiments. To gain acidic media with a pH value of 6.4, 18.5 mM HCl was added to the media. After overnight equilibration in the incubator, the media pH-value was measured with a pH electrode InLab Solids Go-ISM (Mettler Toledo, Gießen, Germany) and adjusted. The coculture was transferred to medium without additional FCS supplementation for 24 h and then incubated with experimental conditions for 30 min, 3 h or 48 h. Control cells were exposed to media with a pH value of 7.4, and the acidosis group was incubated with media with a pH value of 6.4. Only a minor reduction in pHe of the medium was observed during the chosen incubation periods like we described in [12]. Value of pH 7.4 (physiological) was compared with pH 6.4 (pathophysiological), representing typical values, as interstitial pH near the late proximal tubule can drop below 7.0, with pathological conditions beginning around pH 6.8.

### Fractionation of cytosol, nucleus and chromatin

To assess the location of P-CREB in HK-2 cells, a fractionation into cytosolic, nuclear and chromatin was performed. First, the cells were solved in 1 x PBS (see supplementary Table 3) + protease inhibitor cocktail (PIC, 1:500, Merck KGaA, Darmstadt, P8340). The cells were centrifuged for 5 min, at 400 g. The supernatant was removed and the cell pellet was resuspended in 180 µl equilibration buffer (see supplementary Table 3) + PIC (1:100). The solution was centrifuged for 1 min, at 14000 g and the supernatant was removed. For hypotonic lysis, 50 µl lysis buffer (see supplementary Table 3) + PIC (1:100) was added to the pellet and incubated for 10 min. The mixture was centrifuged for 5 min, at 14000 g. The supernatant contained cytosolic proteins. The pellet contained nuclear and chromatin protein and was incubated with 50 µl extraction buffer (see supplementary Table 3) + PIC (1:100) for 30 min. The mixture was centrifuged for 5 min, at 14000 g. The supernatant contained nuclear proteins and the chromatin proteins remained in the pellet. The pellet was purified with 1 x PBS + PIC (1:100) and centrifuged for 5 min, at 14000 g. Finally, the pellet was dissolved in 50 µl Ripa buffer (see supplementary Table 3). It was incubated for 30 min and ultrasonic-lysed for two times for 15 cycles. The samples were mixed with 16.6% of total volume 6 × Laemmli buffer (see supplementary Table 3) and used for western like described in 2.4.

### Western blot

Cells were washed with 1 x PBS (see supplementary Table 3) lysed in 50 µl of ice-cold RIPA buffer (see supplementary Table 3). Afterward, cells were centrifuged at 14,000 g for 10 min (4 °C) and filled up with 16.6% of the total volume of 6 × Laemmli buffer (see supplementary Table 3). Before loading the gel, samples were heated to 95 °C for 5–10 min. Proteins were separated by sodium dodecyl sulfate–polyacrylamide gel electrophoresis (SDS-PAGE) (Gel contains 14% bis-acrylamide to detect IL-6, 10% to detect P-CREB, P-SRF, cfos, P-p38, COX-2 and 7% to detect IL-receptor). Afterwards, the proteins were transferred onto a nitrocellulose membrane. The proteins on the nitrocellulose membrane were stained with Ponceau S solution (AppliChem GmbH, Darmstadt, Germany). We took a picture of the stained membrane with the Bio-Rad ChemiDoc™ XRS gel documentation system (Bio-Rad Laboratories GmbH, Feldkirchen, Germany). After staining, the membrane was blocked with 5% nonfat dry milk powder in TRIS-buffered saline (TBS) (see supplementary Table 3) and incubated with primary antibody (supplementary Table 2) diluted in 5% bovine serum (BSA) in TBS Tween20 overnight. After removing the primary antibody and washing the membrane, a secondary antibody coupled to horseradish peroxidase was diluted 1:1000 in 5% nonfat dry milk powder in TBS Tween20 and added to the membrane. After removal of the secondary antibody solution, three wash steps in TBS Tween20 were performed. Finally, the membrane was incubated for 5 min with Clarity™ Western ECL Substrate (Bio-Rad, Munich, Germany), and the peroxidase activity-based light emission was recorded by an imaging system (Image Quant LAS4000, GE Healthcare, Buckinghamshire, GB). The density of the total protein, made visible with Ponceau S solution, and protein target bands were quantified using Image Lab Software 6.1 (Bio-Rad, Munich, Germany, 2020). The normalization was performed with the Ponceau S-stained total protein. When proteins have similar sizes, the membranes were incubated for 15 min at 65 °C with Restore™ Western Blot Stripping Buffer (Thermo Fisher Scientific GmbH, Dreieich, Germany) to remove the antibodies. Afterward, the membranes were reused for detection.

### IL-6 ELISA

The IL-6 concentration in the media was measured by the IL-6 immunoassay kit (# A35573), of ProQuantum according to the instructions of the company. In principle, this assay uses a matched pair of IL-6-specific antibodies, which are conjugated to a DNA oligonucleotide. If the antibodies bind to IL-6, the two DNA oligos are brought into close proximity. The two strands can be ligated and build a template for strand amplification. The detection was performed by TaqMan-PCR.

### IL-6 receptor occupancy

The occupancy of IL-6 receptor was calculated according to [36], be the equation $\:\frac{F}{Kd\:\times\:F}\times\:100$, whereas F is the IL-6 concentration.

### Calculation of the additive effect

The additive value represents the expected effect if the effects of both treatments act independently and add up linearly and was calculated as follows: Additive value = E_A_ + E_IL6_, whereas E_A_ is the effect of acidosis and E_IL6_ is the effect of IL-6. If a synergistic or antagonistic interaction occurs between the treatments, the observed effect would deviate from this additive value.

### Data analysis

All data are presented as median ± confidence interval. Statistical significance was determined by unpaired Student’s t-test or one-way ANOVA with Holm-Sidak as post-hoc test for normally distributed data. For data without normal distribution, rank sum or ANOVA on ranks with DUNN as post-hoc test was applied. The comparison for the ANOVA-tests was performed to the associated control group. Differences were considered statistically significant when *p* < 0.05.

### Language and writing assistance

The language, phrasing, and wording of this manuscript were reviewed and refined with the assistance of ChatGPT, an AI language model developed by OpenAI. ChatGPT was utilized to check the grammar, clarity, and consistency of the text, as well as to enhance the overall readability. The final version of the manuscript was reviewed and approved by the authors to ensure that it accurately reflects their intended meaning and scholarly standards.

## Results

### Impact of extracellular acidosis on IL-6 expression and secretion

Our previous study showed that an acidic milieu acts synergistically with mediators of PTC-fibroblasts crosstalk [12].

**Fig. 2:**
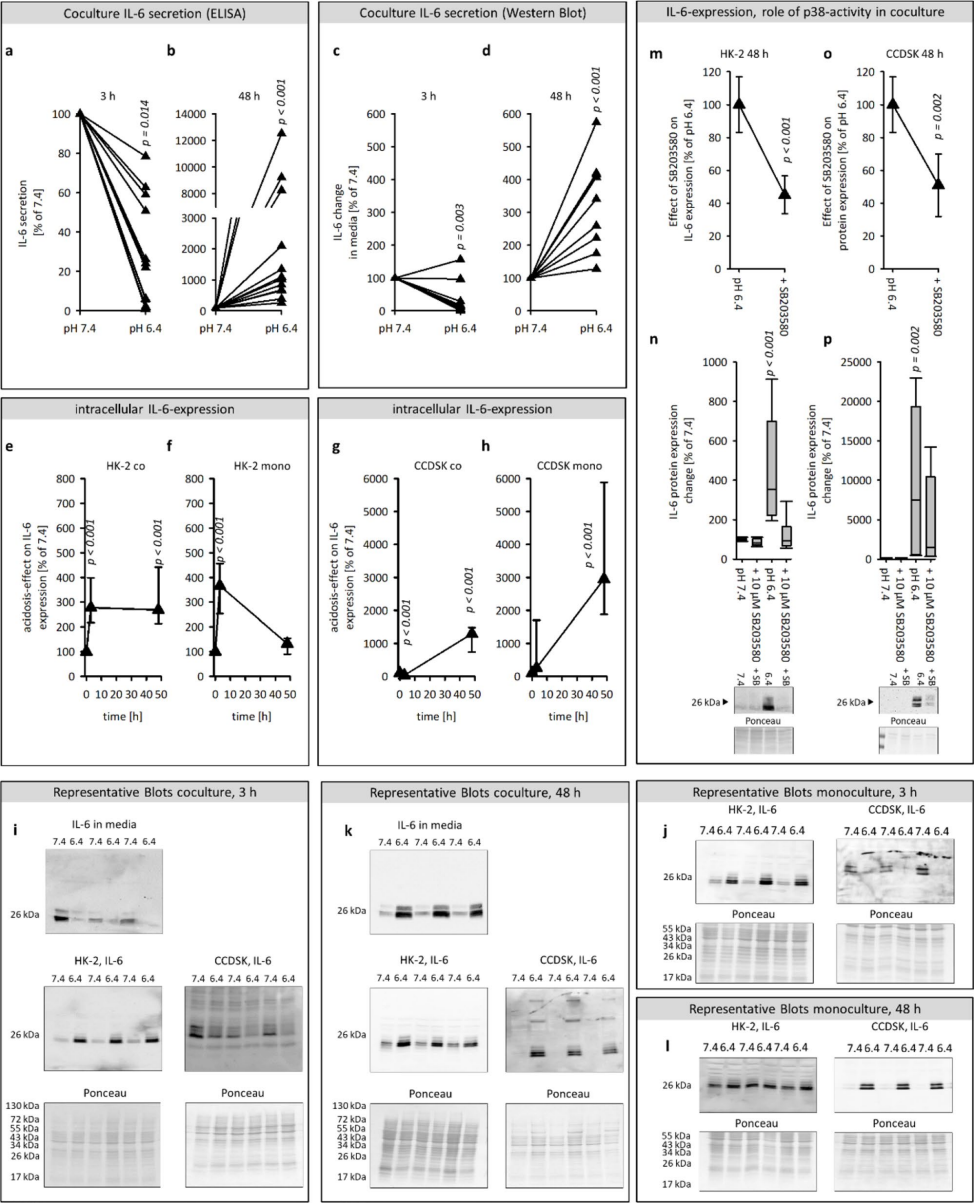
Effect of acidic media on the secretion and expression of IL-6 and the role of p38 activity therein. Acidosis effect on IL-6 secretion after 3 h and 48 h in media from coculture measured by ELISA (**a**, **b**) and western blot (**c**, **d**). Time courses of the acidosis effect on the intracellular protein expression of IL-6 in HK-2 in co-and monoculture (**e**, **f**) and in CCDSK in co-and monoculture (**g**, **h**). Impact of p38 inhibition on IL-6 expression in acidic and control media after 48 h in HK-2 and CCDSK (**m**-**q**). Representative western blots of proteins isolated from cells exposed to acidic or control media (**k** - **j**). *n* = 9–22, significant changes compared to pH 7.4 = *p* < 0.05, for the ANOVA in n-p the comparison was against the associated control group

A crucial aspect of the present study is to verify IL-6 as mediators of the cellular crosstalk. Additionally, the former study pointed out p38-activity as mediator of the acidosis-induced inflammation, therefore the role of p38-activity, for the acidosis-effects on measured targets was assessed. The p38-activity was inhibited by using 10 µM SB203580 in control and acidic media.

#### Extracellular acidosis increased the IL-6 concentration in the coculture media

As shown in Fig. 2a-d incubation to acidic media exerts a biphasic effect on IL-6 level in the media from cells in coculture. This was not the case for media from cells in monoculture (S1). In coculture, the IL-6 concentration in control media was 8.2 pg/ml after 3 h and 238 pg/ml after 48 h, but the secretion rate per hour was unaltered during 48 h at 3.8 pg/ml/h. An acidification of the media led to an initial transient decrease of IL-6 level to 1.7 pg/ml, followed by a sustained increase of IL-6-abundance in the media to 5831 pg/ml (see Table 1).

**Table 1:** IL-6 concentration in media, secretion rate and calculated receptor occupancy. Shown is the median and in brackets the confidence interval (CI)

IL-6 concentration [pg/ml] median, 95% CI		
	3 h	48 h
Coculture 7.4	8.2 [1.8, 39]	238 [108, 940]
Coculture 6.4	1.7 [0.24, 8.6]	5831 [1582, 91039]
IL-6 secretion rate [pg/ml/h]		
Coculture 7.4	3.8 [0.6, 19]	3.7 [1.5, 18.8]
Coculture 6.4	0.8 [0.3, 2.9]	121 [33, 1896]
Saturation of IL-6R		
Coculture 7.4	2.1%	38.8%
Coculture 6.4	0.46%	93.5%

#### Acidic media increased the intracellular protein expression of IL-6

Since the cell type responsible for a media component, like IL-6, cannot be determined by analysing the coculture media, we assessed intracellular IL-6 protein expression in both cell types by western blot. In HK-2 cells, acidic media caused an increase in IL-6 expression after 3 h, independent of the culture condition. However, exclusively in coculture, an acidosis-induced increase was observed after 48 h (Fig. 2e, f). In fibroblasts, acidic media led to a decrease in IL-6 expression after 3 h solely in coculture. In contrast, after 48 h, acidosis caused an increase in IL-6 expression, independent of the culture conditions. We conclude, that the cellular crosstalk is necessary for an acidosis-induced IL-6-secretion in both cell lines. Cellular cross talk is also crucial for a longlasting acidosis driven synthesis of IL-6 in HK-2 cells but not in CCDSK.

#### Acidosis-induced expression of IL-6 is mediated by p38 kinase

The acidosis-induced increase of IL-6 expression after 48 h in HK-2 and CCDSK cells in coculture was attenuated after inhibition of p38-activity (Fig. 2m-p).

### Impact of acidosis on the abundance of CREB, SRF and Cfos

Our next aim was to assess possible upstream pathways of IL-6 expression. Therefore, we measured the impact of acidosis on the expression of transcription factors, which can induce inflammatory responses as well as cellular dedifferentiation. Additionally, the role of p38-activity was assessed.

#### Extracellular acidosis increased the phosphorylation of CREB in coculture

**Fig. 3:**
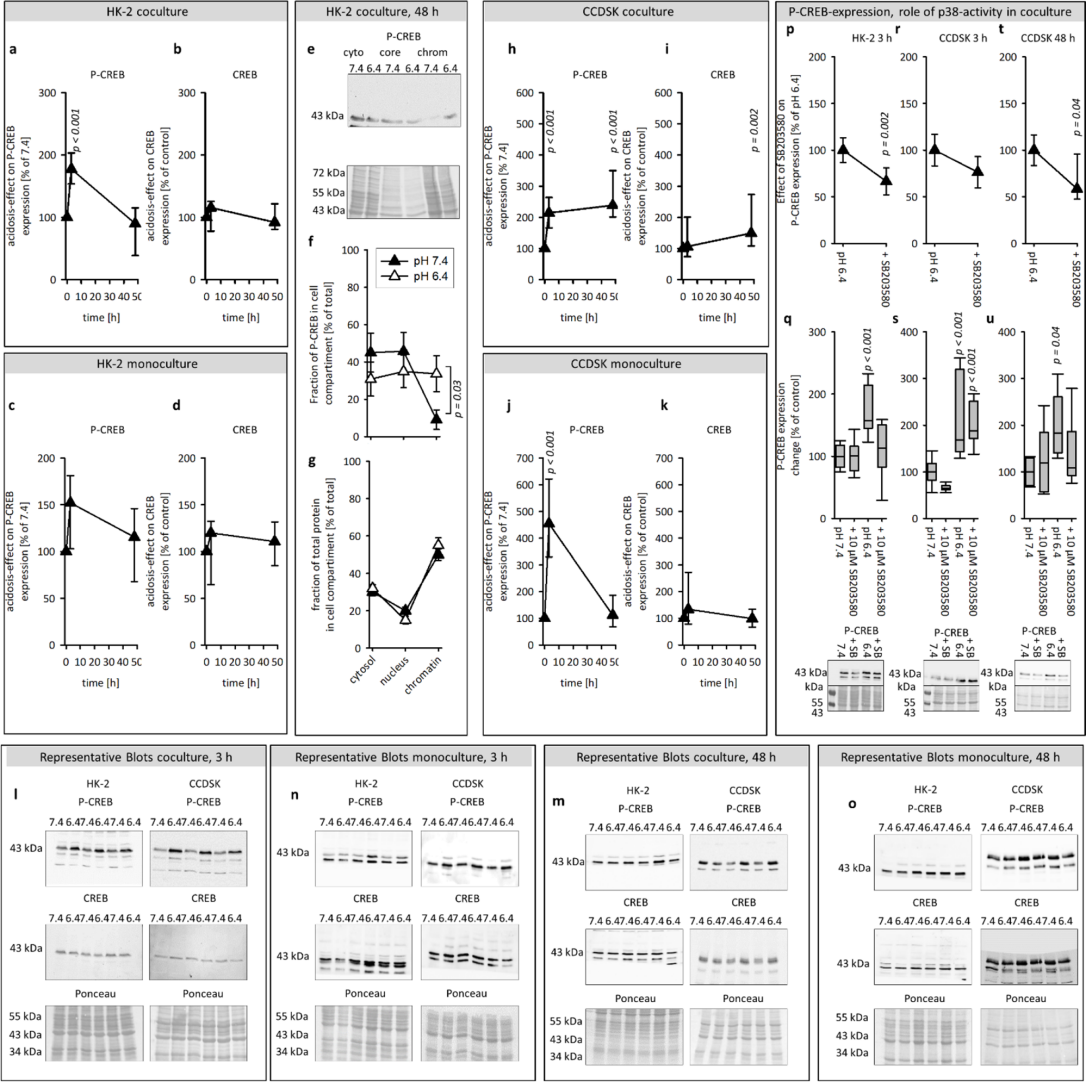
Effect of acidic media on the protein expression of total an P-CREB and the role of p38 activity therein. Time courses of the acidosis effect on protein expression of P-and total CREB in HK-2 in co-and monoculture (**a**-**d**) and in CCDSK in co-and monoculture (**h**-**k**). Effect of acidic or control media on the distribution of P-CREB or total protein in the cytosol, nucleus and chromatin of HK-2 cells in coculture after 48 h (**f**, **g**). Impact of p38 inhibition on P-CREB expression in acidic and control media after 3 h in HK-2 and after 3 h and 48 h in CCDSK (**p**-**u**). Representative western blots of proteins isolated from cells or cell core extracts exposed to acidic or control media (**e**, **l**-**o**). *n* = 8–12, significant changes compared to pH 7.4 = *p* < 0.05 for the ANOVA in q-u the comparison was against the associated control group

P-CREB acts as transcription factor, which can be activated by an enhanced phosphorylation and by an increased expression of total CREB. Consequently, the expression of P- and total CREB was measured in total cell lysates. Figure 3a-d shows that acidosis induced a transient increase of P-CREB in HK-2 cells in coculture. Beside the phosphorylation, the subcellular localisation of P-CREB is crucial for the transcriptional activity. Therefore, we assessed the distribution of P-CREB to cytosol, nucleus and chromatin in HK-2 cells. Figure 3e, f shows that acidosis increased the proportion of P-CREB at the chromatin after 48 h in HK-2 cells. We measured the distribution of total protein by ponceau, to exclude that the measured effects on P-CREB are the results of a general protein shift. Figure 3 g shows that acidosis had no general impact on the distribution of the total protein. In CCDSK, the incubation to acidic media caused a transient increase of P-CREB expression in monoculture, whereas in coculture this effect appears early and is long-lasting (Fig. 3h-k).

#### Acidosis-induced CREB-phosphorylation is mediated by p38 kinase

Figure 3p-u shows that an inhibition of p38-activity vanished the acidosis-impact on P-CREB in HK-2 after 3 h and in CCDSK after 48 h. These results suggest that the acidosis-induced increase of CREB-phosphorylation is mediated by p38-activity.

#### Impact of acidosis on SRF expression and phosphorylation

**Fig. 4:**
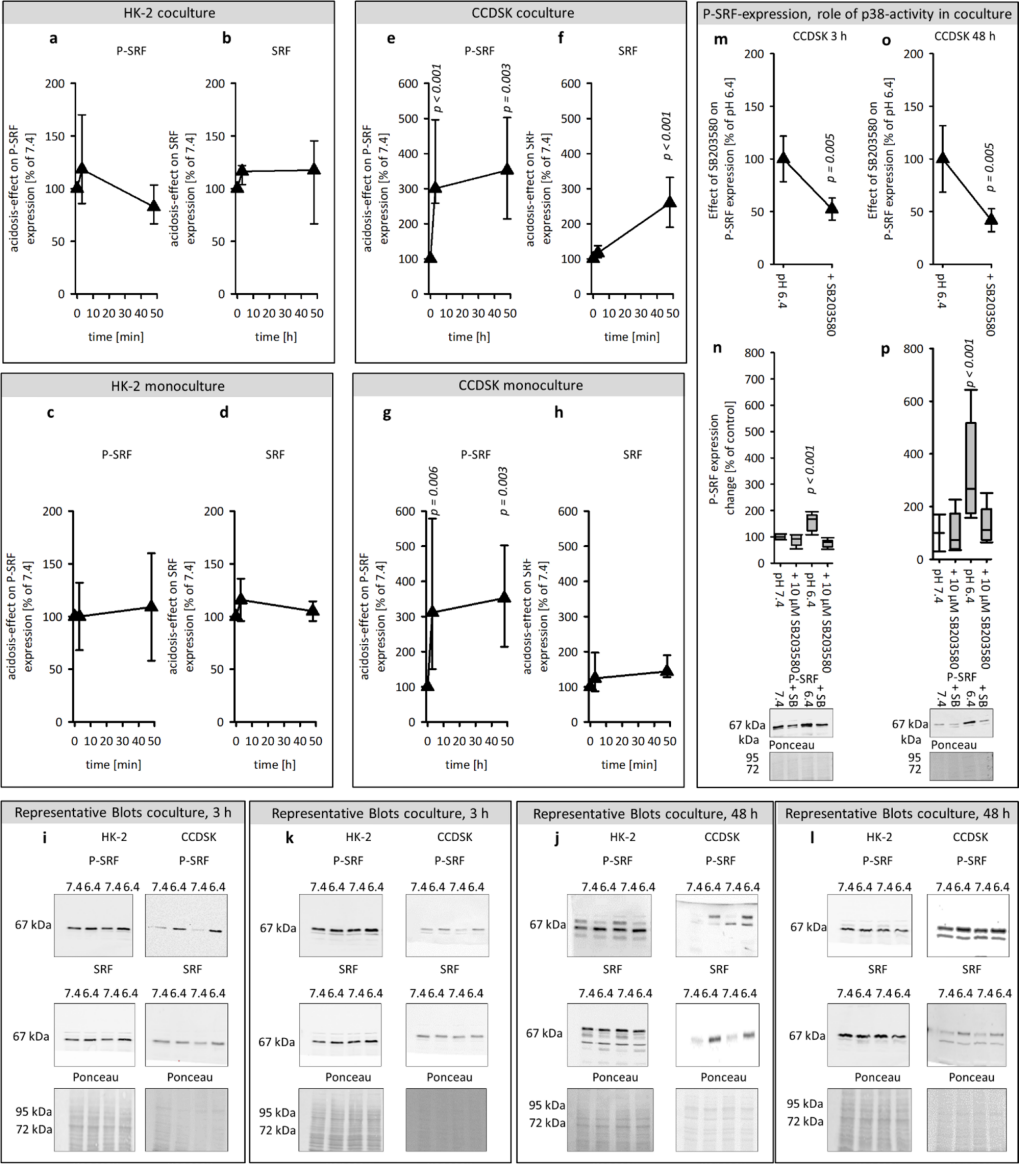
Effect of acidic media on the protein expression of total an P-SRF and the role of p38 activity therein. Time courses of the acidosis effect on protein expression of P-and total SRF in HK-2 in co-and monoculture (**a**-**d**) and in CCDSK in co-and monoculture (**e**-**h**). Impact of p38 inhibition on P-CREB expression in acidic and control media after 3 h and 48 h in CCDSK (**m**-**p**). Representative western blots of proteins isolated from cells exposed to acidic or control media (**i**-**l**). *n* = 6–12, significant changes compared to pH 7.4 = *p* < 0.05, for the ANOVA in n-p the comparison was against the associated control group

SRF is a transcription factor, which mediates the response to numerous extracellular stimuli. Figure 4a-d shows that acidosis had no impact on the expression of SRF or P-SRF in HK-2 cells, under any condition. However, in CCDSK acidic media caused an early and long-lasting increase of the SRF-phosphorylation independent of the culture-condition and led to an increase of total SRF after 48 h in coculture only (Fig. 4e-h).

#### Acidosis-induced phosphorylation of SRF is mediated by p38 kinase

Figure 4m-p shows that an inhibition of p38-activity prevented the acidosis-impact on P-SRF after 3 h and 48 h in CCDSK. These data provide hints that the acidosis-induced increase of SRF-phosphorylation is mediated by p38-activity.

#### Impact of acidosis on cfos expression

**Fig. 5:**
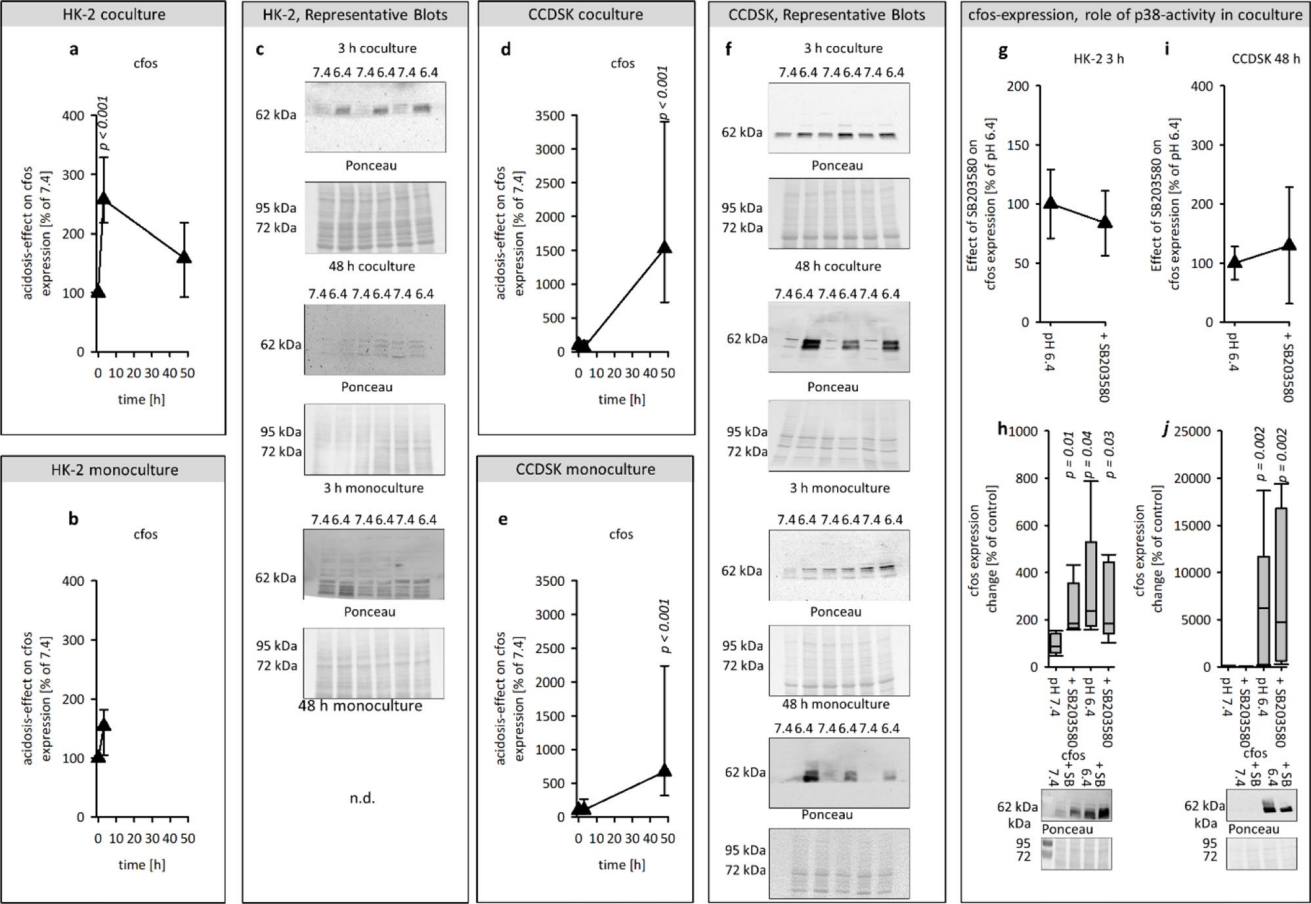
Effect of acidic media on the protein expression of cfos and the role of p38 activity therein. Time courses of the acidosis effect on protein expression of cfos in HK-2 in co-and monoculture (**a**, **b**) and in CCDSK in co-and monoculture (**d**, **e**). Impact of p38 inhibition on P-CREB expression in acidic and control media after 3 h in HK-2 and after 3 h and 48 h in CCDSK (p-u). Representative western blots of proteins isolated from cells exposed to acidic or control media (**c**, **f**). *n* = 6–9, significant changes compared to pH 7.4 = *p* < 0.05 for the ANOVA in h-j the comparison was against the associated control group. n.d. = not detectable

The early response protein cfos is a member of the AP-1 family which mediates the transcription of e.g. cytokines. Moreover, cfos is a target of P-CREB and SRF. In HK-2 cells an incubation with acidic media led to a transient increase of cfos in coculture only (Fig. 5a-c). After 48 h exposure to acidic media in monoculture, the protein expression of cfos was below the detection limit (n.d. = not detectable). In CCDSK acidosis led to an immense sustained increase of cfos after 48 h this effect is independent of the culture conditions (Fig. 5d-f).

#### Acidosis-induced expression of Cfos is independent of p38 kinase

Figure 5g-j shows that the acidosis induced increase of cfos expression is independent of p38-activity.

**Fig. 6:**
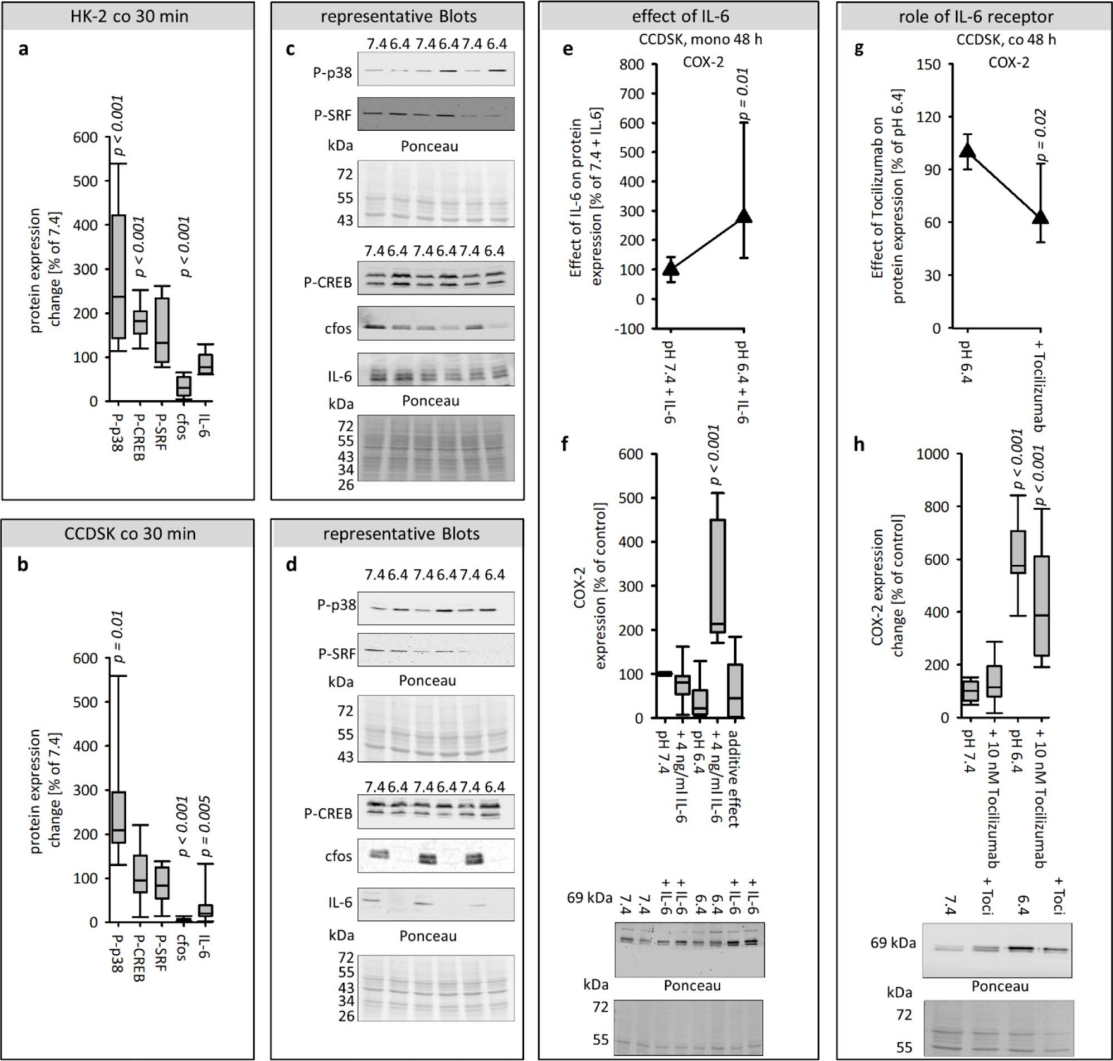
Effect of acidic media on protein expression after 30 min and the role of IL-6 for acidosis-effects. Impact of acidic media on the protein expression of P-p38, P-CREB, P-SRF, cfos and IL-6 after 30 min exposure in HK-2 and CCD-1092Sk cells and representative western blots (**a**-**d**). Influence of IL-6 in acidic or control media on COX-2 expression after 48 h, in CCDSK, in monoculture, and representative western blots (**e**-**f**). The additive value represents the expected effect if the effects of both treatments act independently and add up linearly. This serves as a reference value for comparison with the actual combined effect of the two treatments. Significance of interleukin-6 receptor in coculture, for acidosis-induced changes of COX-2-expression in CCDSK after 48 h, and representative western blots (**g**-**h**). *n* = 4–10, significant changes compared to pH 7.4 = *p* < 0.05 for the ANOVA in g-h the comparison was against the associated control group

### Impact of acidosis on the expression of P-p38, P-CREB, P-SRF, cfos and IL-6 after 30 min

To get more hints for the sequence in which the signaling pathways are activated, we measured the effect of acidosis after 30 min. Figure 6a shows that 30 min exposure to acidic media led to an increase of P-p38 and P-CREB, but a decrease of cfos in HK-2 cells. In CCDSK only the expression of P-p38 was increased after 30 min, whereas the expression of cfos and IL-6 was decreased (Fig. 6b). These results indicate that p38-phosphorylation is one of the initial cellular responses. This reaction is common to both cell types.

### Acidosis enhances not only IL-6 expression but also its impact on fibroblasts

The results show that IL-6 increased in the medium of the coculture only after 48 h. Therefore, the aim of the subsequent experiments was to investigate whether the addition of IL-6 to the acidic and control media of the CCDSK in monoculture can induce effects that were observed exclusively in coculture. Therefore, the expression of COX-2, a typical target of the IL-6 pathway, was measured. Figure 6e-f shows that an addition of IL-6 to acidic media but not to the control media caused an increase of COX-2 in CCDSK in monoculture. A comparison between calculated additive effect and the measured effect allows to the conclusion that IL-6 and an acidosis act synergistically.

### Acidosis-induced COX-2 expression depends on the interleukin-6 receptor

It was shown that IL-6 and the acidic media act synergistical on the protein expression of COX-2. The next step was to measure the role of the IL-6 receptor, for the acidosis-induced effects in coculture, by inhibiting the IL-6 receptor with 10 nM tocilizumab (abcam, ab275982). Figure 6g-h shows that the IL-6 receptor is crucial for the acidosis-induced increase of COX-2 in CCDSK after 48 h.

## Discussion

In this study, we utilized a human coculture model to investigate the role of IL-6 as a possible mediator of the cellular crosstalk in an acidic milieu. It is important to note that both cell lines used are immortalized, which poses some limitations in terms of accurately replicating in vivo conditions. However, this model is well suited for mechanistic investigations at the cellular level. The HK-2 cells, derived from the proximal tubule, exhibit polarized morphology and express key transporters typical of the proximal tubule, such as sodium-glucose symporter-2 (SGLT-2) and glucose transporter 1 (GLUT1) [37]. Additionally, HK-2 cells have been shown to respond to extracellular stimuli like drugs and toxins, making them a suitable model for studying the effect of extracellular acidosis on cell fate [38, 39]. The CCD-1092Sk cells, though originating from the skin, are appropriate for our coculture model. Studies by Korsunsky et al. and Büchler et al. suggest that the microenvironment plays a more significant role in shaping fibroblast phenotype than their tissue of origin [10, 40]. This supports the use of CCD-1092Sk cells for coculture experiments. It must be considered that cellular crosstalk is even more complex in vivo, and under pathophysiological conditions, signals from immune cells such as macrophages or endothelial are crucial for the fate of proximale tubule cells during kidney injury [41, 42]. Therefore, to obtain a more comprehensive understanding of the significance of cellular communication, future studies should consider cocultures of tubular cells with endothelial cells and/or macrophages. Due to the he central role of tubulo-fibroblast crosstalk in both physiological and pathophysiological processes of the kidney, we used a model which enables the precise investigation of their specific interactions, independent of additional cell types.

Like described in our former study, there is no single physiological or pathophysiological pH value, but rather ranges for both [12]. We selected pH 7.4 as an example from the physiological range and pH 6.4 from the pathophysiological range. This allowed us to explore the role of cellular crosstalk in acidosis. Given that proximal tubular pH can drop to around 6.8 under physiological conditions, it is reasonable to assume a transition to the pathophysiological range near this value, making pH 6.4 representative of a pathological environment. The choice of pH 6.4 also took into account cell viability, avoiding necrosis or apoptosis that would not reflect in vivo conditions.

Local acidosis can be caused by an increase in CO₂ or by the accumulation of fixed acids (= metabolic or non-respiratory acidosis; frequently caused by lactic acid), leading to a decrease in standard bicarbonate. Our aim was to investigate specifically the effects of non-respiratory acidosis, as observed in inflammation, ischemia, or under nephrotoxic circumstances. In our initial approach, the primary objective was to lower pH while minimizing changes in anion concentrations. However, complete constancy of anion concentrations is not achievable, and a trade-off was made using HCl, resulting in an increased chloride concentration by 20 mM. This results in a 1.17-fold increase in chloride concentration, while the proton concentration must be increased 10-fold to lower the pH from 7.4 to 6.4. The comparison of these differences suggests that the increase in proton concentration is primarily responsible for the observed effects. Although chloride has been discussed in the literature as a potential signaling molecule, experimental evidence supporting a functional role of extracellular chloride changes in signaling processes remains limited. Lüscher et al. reported that there is currently little evidence suggesting that modulation of extracellular chloride concentration directly acts as a signaling mechanism [43].

In previous studies, we demonstrated that communication between PTC and fibroblasts modifies the impact of extracellular stimuli on inflammatory response, cell differentiation and the mediating signaling pathways [44, 45]. Further research revealed a synergistic action of tubulo-fibroblasts crosstalk and extracellular acidosis on the inflammatory response of human cell lines [12]. The cytokine IL-6 is a significant driver of inflammatory processes and additional it is stated that IL-6 signaling supports the progression of chonic kidney diseases [14]. Furthermore, it is known that an acidic micromilieu elevates the IL-6 expression in skeletal muscle cells ad immune cells [19, 46]. The current study aimed to investigate whether IL-6 acts as a mediator in the communication between tubule cells and fibroblasts. In control media (pH 7.4) from coculture, the IL-6 concentration was 0.3 pM (8.2 pg/ml) after 3 h and reached 9.5 pM (238 pg/ml) after 48 h (Table 1). IL-6 secretion seemed to be constant with a rate of 0.15 pM/h (3.8 pg/ml/h). Table 1 shows an acidosis-induced increase of IL-6 to 233 pM (5831 pg/ml) after 48 h, whereas after 3 h it was reduced to 0.07 pM (1.7 pg/ml). In contrast, under monoculture conditions, acidic media caused no increase of secreted IL-6 after 3 h or 48 h in HK-2-cells or CCDSK. Thus, only in coculture extracellular acidosis acts as stimuli for the IL-6 secretion after 48 h. Under inflammatory conditions serum IL-6 can increase from 1 to 5 pg/ml up to 150 ng/ml [47, 48], suggesting that the measured IL-6 concentration in coculture can be achieved in vivo under pathophysiological conditions. To induce a cellular response, IL-6 has to bind its receptor that will activate downstream signaling pathways. Supplementary Table 4 shows that both cell lines express both subunits of the IL-6 receptor, providing the necessary requirement for IL-6 action.

Another aspect, which has to be taken in account it the receptor occupancy. The Kd value of the IL-6 receptor is 15 pM, thus exclusively in coculture media after 48 h IL-6 levels that can sufficiently activate the receptor are reached [36]. After 48 h incubation in coculture the IL-6 concentration would lead to a receptor occupancy of 39% in control groups and 94% in acidic groups. Receptor occupancy after 3 h is only 2.1% in the control group (Table 1). Consequently, IL-6 can act as mediator of long-lasting inflammatory responses in coculture. A limitation of using coculture models is that in most cases the source of secreted proteins in the medium cannot be definitely attributed to one of the cell types. Instead, it can be determined whether the overall concentration of a given protein changes, providing insights into the composition and dynamics of the microenvironment. However, in order to obtain an indication as to which of the two cell types is the probable source of IL-6 secretion the impact of acidosis on intracellular IL-6 expression was determined in mono- and coculture was determined and compared. In coculture, the intracellular IL-6 protein and mRNA was increased in both cell types over 48 h, making both cell types a possible source of extracellular IL-6. However, when examining IL-6 secretion in monoculture under control conditions, showed that HK-2 cells secrete 148 times more IL-6 than CCDSK cells per total cellular protein (supplementary Table 1). Thus, we suggest that HK-2 cells are the primary source of IL-6. We are aware of the limitations for this conclusion and that at present unequivocal evidence is still missing. However, currently there is no experimental approach available for a more direct proof. In monoculture, incubation with an acidic medium also leads to an increase in intracellular IL-6 expression. In HK-2 cells, this effect was transient, whereas in CCDSK cells it was prolonged. However, since this intracellular increase in IL-6 is not reflected in the medium of the monoculture, it cannot contribute to the activation of the classical IL-6 signaling pathway. According to current knowledge, intracellular synthesized IL-6 is immediately released into the extracellular space. Neither intracellular IL-6 storages, nor regulation mechanisms targeting the secretion are known. In light of this fact, the observed acidosis effects in monoculture are surprising. Possible explanations for the discrepancy between intracellular and extracellular IL-6 expression changes can be an enhanced receptor-mediated internalization of IL-6 by the cells and the inhibition of the sIL-6R-IL-6 complex by soluble GP130. First, cells can internalize and degrade the IL-6-IL-6 receptor complex, to mitigate the IL-6 signaling [48]. This could explain an increase in intracellular IL-6 without a corresponding rise in IL-6 levels in the medium. Second, beside the membrane-bound form of the IL-6 receptor, a soluble form (sIL-6R) is described. To prevent excessive activity of the sIL-6R-IL-6 complex, soluble GP130 can bind to this complex and thereby inactivate it [48, 49]. Possibly the antibody binding site of IL-6 is masked in this complex and therefore not detectable by ELISA. In summary, our findings indicate that IL-6 emerges as a mediator of cellular communication exclusively in coculture after 48 h. In our previous study, we demonstrated that the MAPK p38 is a key mediator of acidosis-induced changes and that acidosis induced p38 activation in both cell types in coculture [12]. Consistent with these findings, the present study demonstrated that the acidosis impact on IL-6 expression in coculture depends on p38 activity. Beside the increased IL-6 protein expression, RNA-sequencing data (not published) show that after 48 h in coculture, an acidic medium lead to an increase in IL-6 RNA (table S4). This suggests the involvement of transcriptional regulation, mediated by transcription factors. In addition to the classical factors like NF-κB, C/EBP or CREB, SRF may also play a role [50]. Thus, CREB and SRF can be phosphorylated by p38, they emerge as promising candidates to link p38 activity with IL-6 expression [17, 51]. The data presented here demonstrate that an acidic media induced increased CREB activation. This effect was observed in both cell types in coculture and aligns with the acidosis-driven CREB activation described in the literature [52, 53]. The inhibitory effect of SB203580 on acidosis-induced CREB-phosphorylation was determined in both cell types, indicating that the CREB-phosphorylation is mediated by p38. Riemann et al. also described an acidosis-induced activation of CREB via p38 in a former study with tumor cells [54]. Taken together with CREB-induced IL-6 expression, an H^+^-p38-CREB-IL-6 axis is likely [55]. The present data further demonstrate that the culture conditions play a role in the acidosis-induced activation of CREB. For HK-2 cells, it can be stated that CREB activation is the result of a synergistic effect of acidic environment and cellular communication. In contrast, for fibroblasts, transient CREB-phosphorylation occurs independently of the culture conditions, but the effect is only sustained in coculture. This suggests that extracellular acidosis initiates the activation of the CREB-signaling pathway, with cellular communication being crucial to sustain its activity. Furthermore, it is shown that in HK-2 cells, only p-p38 and p-CREB are enhanced after 30 min of acid exposure. Given that CREB-phosphorylation depends on p38 activity, it is evident that p38 plays an initiating role here. A similar observation was made in fibroblasts, where only P-p38 levels were elevated after 30 min of acid treatment. In contrast to CREB, SRF-phosphorylation has been found to be cell-specific, occurring exclusively in fibroblasts. Additionally, it has been demonstrated that SRF-phosphorylation is mediated by p38 but is independent of the culture conditions. The role of SRF-phosphorylation is unclear up to now. Some hints indicate that phosphorylation at Ser103 enhances the DNA-affinity of SRF and thus its activity [55]. Based on this assumption, the present data reveal the SRF-signaling as further pathway, which can mediate the p38-signaling to an increased IL-6 transcription in fibroblasts.

CREB or SRF can promote the expression of further transcription factors that mediate the effect on IL-6. A common target gene of both CREB and SRF is c-Fos [25, 56, 57]. As an early response protein, c-Fos serves as a potential mediator linking the CREB and/or SRF-signaling pathways to the upregulation of IL-6. Although, extracellular acidosis leads to an increase in c-Fos expression in both cell lines, it seems not to be part of the H^+^-p38-CREB/SRF-IL-6 axis, as p38 inhibition does not affect the acidosis-induced c-Fos expression. These findings suggest that acidosis-induced cFos expression is mediated by parallel signaling pathways, such as other MAPKs, particularly JNK [58]. This implies that cFos is not directly integrated into the p38-MAPK-dependent signaling cascade but rather plays an independent role within the acidosis-induced cellular response network. This would suggest an additional, parallel regulatory layer for the inflammatory response to acidosis. However, direct experimental validation of this hypothesis remains challenging due to the lack of specific cFos inhibitors. Nevertheless, our findings highlight the relevance of this signaling pathway, and future studies will focus on further investigating the functional role of cFos-mediated regulation in this context. In summary, the present data reveal that the upstream signaling of IL-6 involves a complex interplay of pathways. Furthermore, in fibroblasts, a p38-driven phosphorylation of SRF is observed, which can also contribute to IL-6 expression regulation.

To assess not only the expression of IL-6 during acidosis but also a possible impact of acidosis on IL-6-induced effects we investigated the interplay of IL-6 and acidosis in monoculture. Therefore, cells were incubated with control and acidic media containing IL-6. As previous findings demonstrated that acidosis-induced COX-2 expression in fibroblasts occurs only in coculture, we used the COX-2 expression in fibroblasts as outcome parameter [12]. Our results show that neither acidosis alone nor IL-6 alone induced the expression of COX-2 in fibroblasts under monoculture conditions. In contrast, IL-6 in an acidic milieu increased the COX-2 expression showing a synergism between IL-6 and acidosis. These results emphasize the significance of IL-6 as mediator under acidic conditions that potentiate its impact. In addition, the significance of IL-6 signaling in coculture was confirmed by pharmacological inhibition of the IL-6 receptor. Thus, the IL-6 receptor is crucial for the acidosis-induced expression of COX-2 in fibroblasts in coculture. As the acidosis-induced increase of COX-2 is also dependent on p38, we conclude that the increased COX-2 expression in fibroblasts in coculture results from a synergism between acidosis and IL-6, mediated by p38 [12]. In summary, our results provide experimental evidence that intercellular communication occurs via IL-6. However, the specific mediator responsible for the upstream signaling leading to p38 activation remains to be identified. The observed synergism in monoculture may indicate that cellular communication is mediated by IL-6 receptor transsignaling [48]. Transsignaling requires IL-6 and the IL-6 receptor. If only one of the two is present, no signaling occurs. It is possible that acidosis in fibroblasts leads to the cleavage of the soluble receptor, while IL-6 secretion is not increased. Conversely, in pH-neutral media, when IL-6 is added, there is a lack of its soluble receptor, and in both cases, no transsignalling is initiated. However, the combination of an acidic medium and sufficient IL-6 would lead to an inflammatory response, represented by increased COX-2 expression. This mechanism may also be relevant in coculture. Maybe acidosis induces IL-6 receptor shedding in CCDSK cells while promoting the secretion of IL-6 at sufficient concentrations in HK-2 cells. Therefore, a key objective of future studies is to investigate the effects of an acidic medium on IL-6 transsignaling to elucidate a potential mechanism of cellular crosstalk.It is described that a micromilieu changes of proximal tubule cells, containing hypoxia, hyperglycaemia or inflammation can induce the COX-2 pathway. Zhuang et al. showed that an activity of COX-2 mediates proximal tubule injury [59]. Moreover, cytokines like IL-6 or TNF can drive an inflammatory process by inducing COX-2 expression in chronic kidney disease [60, 61]. As consequence, COX-2 can also modulate the cellular communication by secreting metabolites like PGE2. Thus, COX-2 activity can induce the expression of cytokines like IL-6 and create a vicious cycle [59]. Due to this knowledge, the COX-2-PGE2-EP2 axis is discussed as therapeutic target to prevent kidney injury [48, 62] COX-2 inhibitors have been widely studied for their potential therapeutic effects in kidney diseases, including their ability to modulate inflammatory pathways, reduce proteinuria and as treatment for Nephrogenic diabetic insipidus [63–65].A key objective of future studies is to determine the role of COX-2 in acidosis-induced cellular changes. To address this question, specific COX-2 inhibitors such as indomethacin or SC-58,125 can be used. Our data show, that only under coculture conditions, acidosis induced a longlasting IL-6 expression in HK-2 and, it was shown that in coculture solely the COX-2 expression was increased in CCDSK after 48 h. Therefore, a future issue will be if the COX-2 activity is sufficient for the longlasting IL-6 expression and secretion. Moreover, the secretion of prostaglandin E2 will be tested to estimate if COX-2 metabolites produced by fibroblasts, mediate the longlasting IL-6 expression in HK-2 cells.

The present study reveals IL-6 as potential mediator of the tubule-fibroblast crosstalk in an acidic milieu, which drives the inflammatory process. IL-6 and acidosis are interconnected in two ways: acidosis induced IL-6 expression and IL-6 action on target cells. This process is mediated through a signal network in which p38 plays a crucial and initiating role. If these effects prove to be true in vivo, they could significantly contribute to understanding the development of chronic kidney diseases and to developing early interventions.

## Electronic supplementary material

Below is the link to the electronic supplementary material.

Supplementary Material 1

Supplementary Material 2

## Data Availability

The datasets used and/or analysed during the current study are available from the corresponding author on reasonable request.

## Abbrevations

BSA: Bovine serum
CDK: Chronic kidney diseases
COX: Cyclooxygenase
CREB: CAMP response element-binding protein
DNA: Deoxyribonucleic acid
ELISA: Enzyme-linked immunosorbent assay
FCS: Fetal calf serum
GLUT: Glucose transporter
IL: Interleukin
MAPK: Mitogen-activated protein kinase
NFκB: Nuclear factor κB P in front = phosphorylated
PBS: Phosphate-buffered saline
PCR: Polymerase chain reaction
pHe: Extracellular pH value
PTC: Proximal tubule cells
RIPA: Radioimmunoprecipitation buffer
SDS-PAGE: Sodium dodecyl sulfate–polyacrylamide gel electrophoresis
SEM: Standard error of the mean
SGLT: Sodium-glucose symporter
SRF: Serum response factor
TBS: TRIS-buffered saline
