# Supplementary material for: Acidosis-induced p38-kinase activation triggers an IL-6-mediated crosstalk of renal proximal tubule cells with fibroblasts leading to their inflammatory response
Source: Cell Commun Signal. 2025 Apr 11;23:180. doi: 10.1186/s12964-025-02180-5 (PMC11987431; doi:10.1186/s12964-025-02180-5)
Supplement: Supplementary file 1 — Supplementary Material 1 [file 12964_2025_2180_MOESM1_ESM.pptx]

## Slide 1
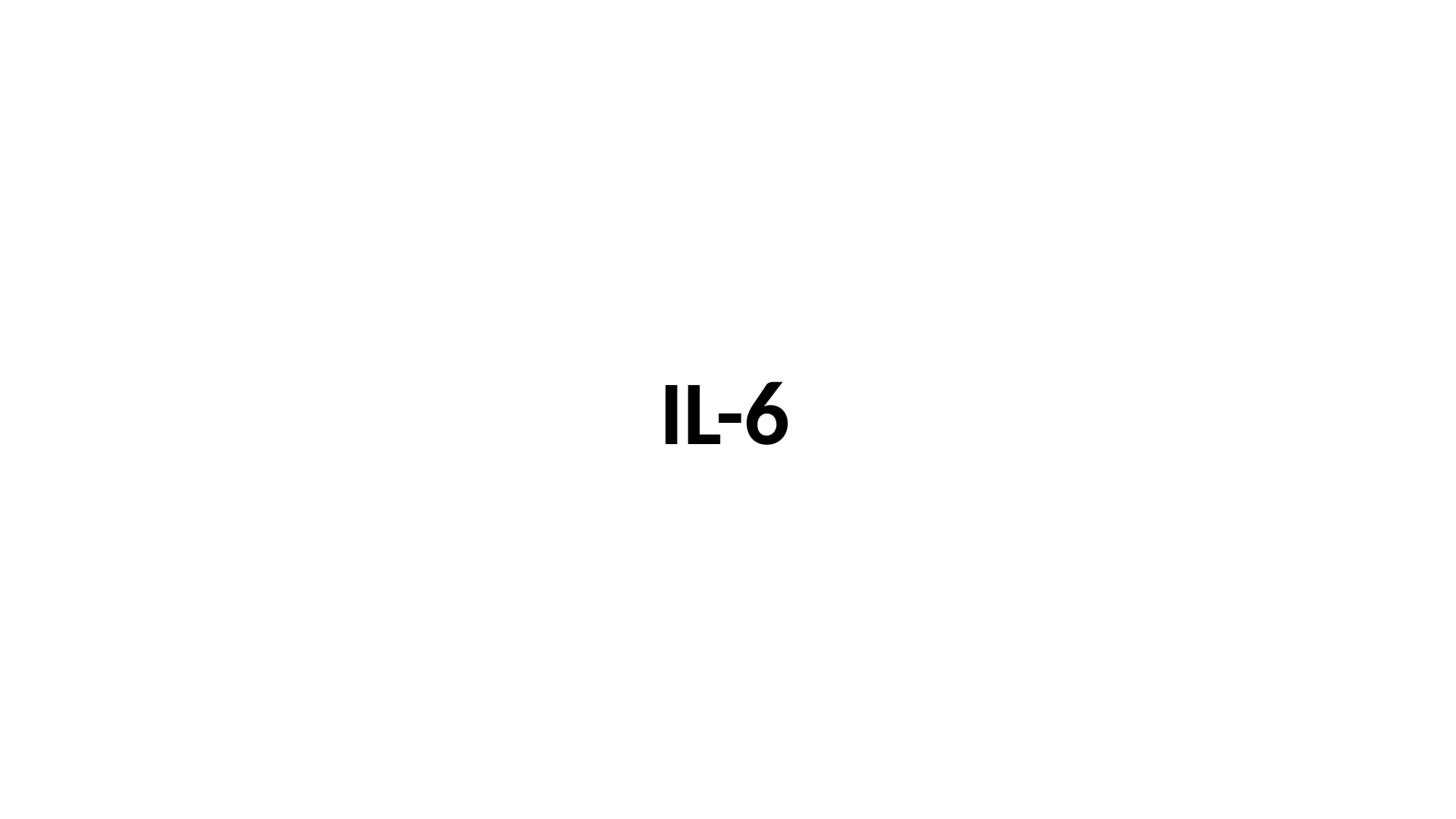

IL-6

## Slide 2
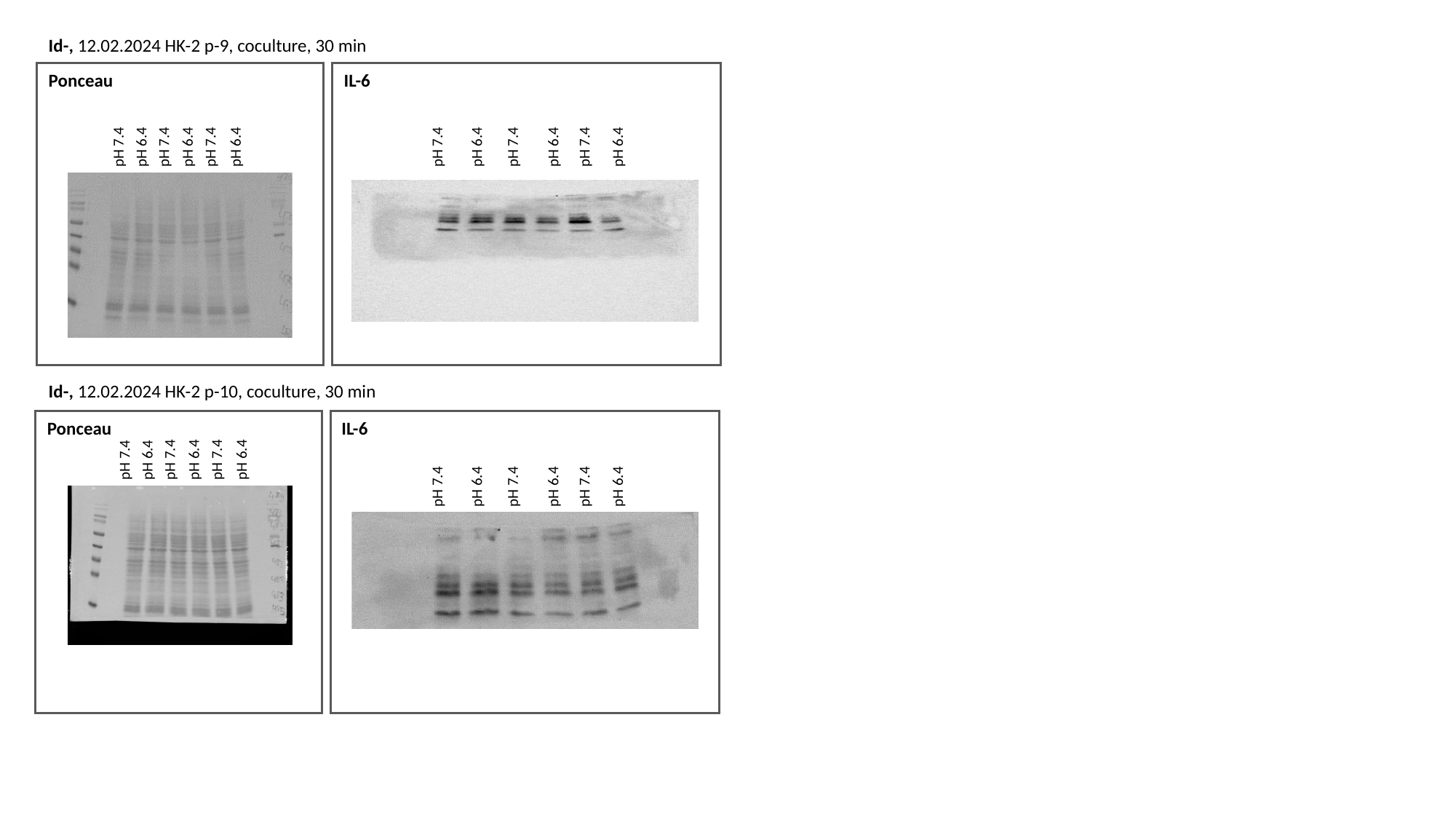

Id-, 12.02.2024 HK-2 p-9, coculture, 30 min
IL-6
Ponceau
pH 6.4
pH 6.4
pH 6.4
pH 6.4
pH 6.4
pH 6.4
pH 7.4
pH 7.4
pH 7.4
pH 7.4
pH 7.4
pH 7.4
Id-, 12.02.2024 HK-2 p-10, coculture, 30 min
Ponceau
IL-6
pH 6.4
pH 6.4
pH 6.4
pH 7.4
pH 7.4
pH 7.4
pH 6.4
pH 6.4
pH 6.4
pH 7.4
pH 7.4
pH 7.4

## Slide 3
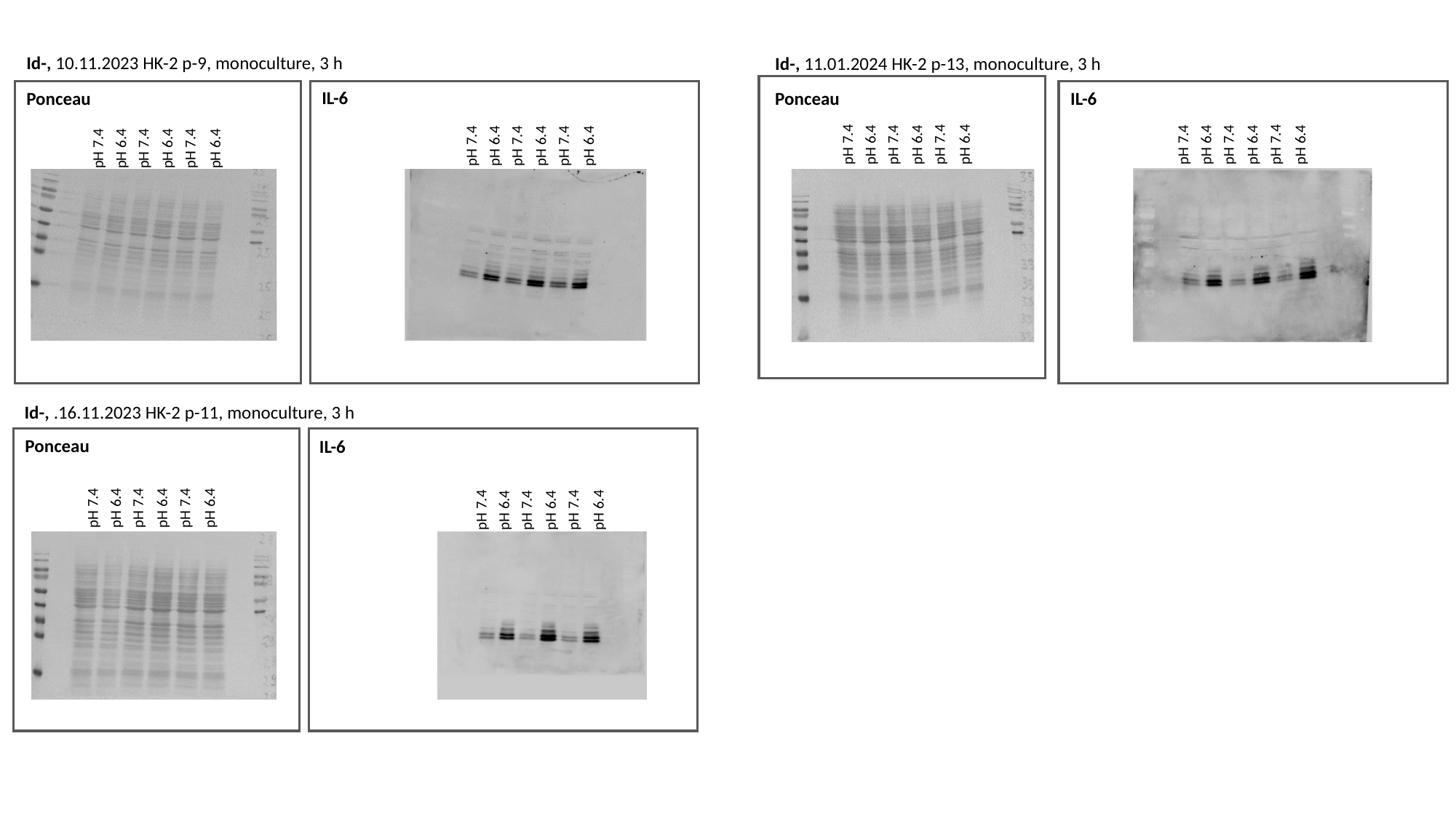

Id-, 10.11.2023 HK-2 p-9, monoculture, 3 h
Id-, 11.01.2024 HK-2 p-13, monoculture, 3 h
IL-6
Ponceau
IL-6
Ponceau
pH 6.4
pH 6.4
pH 6.4
pH 6.4
pH 6.4
pH 6.4
pH 6.4
pH 6.4
pH 6.4
pH 6.4
pH 6.4
pH 6.4
pH 7.4
pH 7.4
pH 7.4
pH 7.4
pH 7.4
pH 7.4
pH 7.4
pH 7.4
pH 7.4
pH 7.4
pH 7.4
pH 7.4
Id-, .16.11.2023 HK-2 p-11, monoculture, 3 h
Ponceau
IL-6
pH 6.4
pH 6.4
pH 6.4
pH 6.4
pH 6.4
pH 6.4
pH 7.4
pH 7.4
pH 7.4
pH 7.4
pH 7.4
pH 7.4

## Slide 4
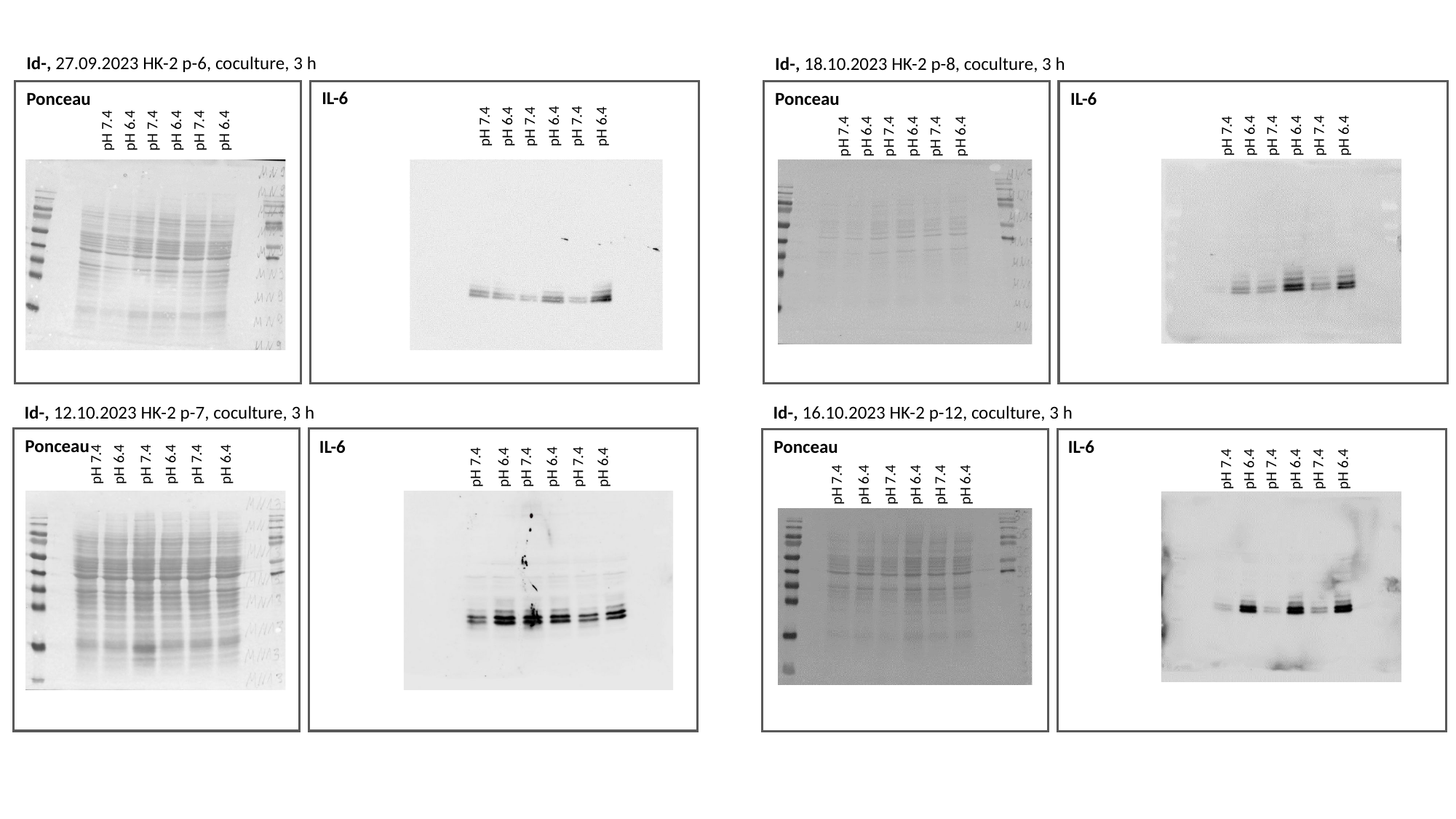

Id-, 27.09.2023 HK-2 p-6, coculture, 3 h
Id-, 18.10.2023 HK-2 p-8, coculture, 3 h
IL-6
Ponceau
IL-6
Ponceau
pH 6.4
pH 6.4
pH 6.4
pH 6.4
pH 6.4
pH 6.4
pH 7.4
pH 7.4
pH 7.4
pH 6.4
pH 6.4
pH 6.4
pH 6.4
pH 6.4
pH 6.4
pH 7.4
pH 7.4
pH 7.4
pH 7.4
pH 7.4
pH 7.4
pH 7.4
pH 7.4
pH 7.4
Id-, 12.10.2023 HK-2 p-7, coculture, 3 h
Id-, 16.10.2023 HK-2 p-12, coculture, 3 h
Ponceau
IL-6
Ponceau
IL-6
pH 6.4
pH 6.4
pH 6.4
pH 6.4
pH 6.4
pH 6.4
pH 6.4
pH 6.4
pH 6.4
pH 7.4
pH 7.4
pH 7.4
pH 7.4
pH 7.4
pH 7.4
pH 7.4
pH 7.4
pH 7.4
pH 6.4
pH 6.4
pH 6.4
pH 7.4
pH 7.4
pH 7.4

## Slide 5
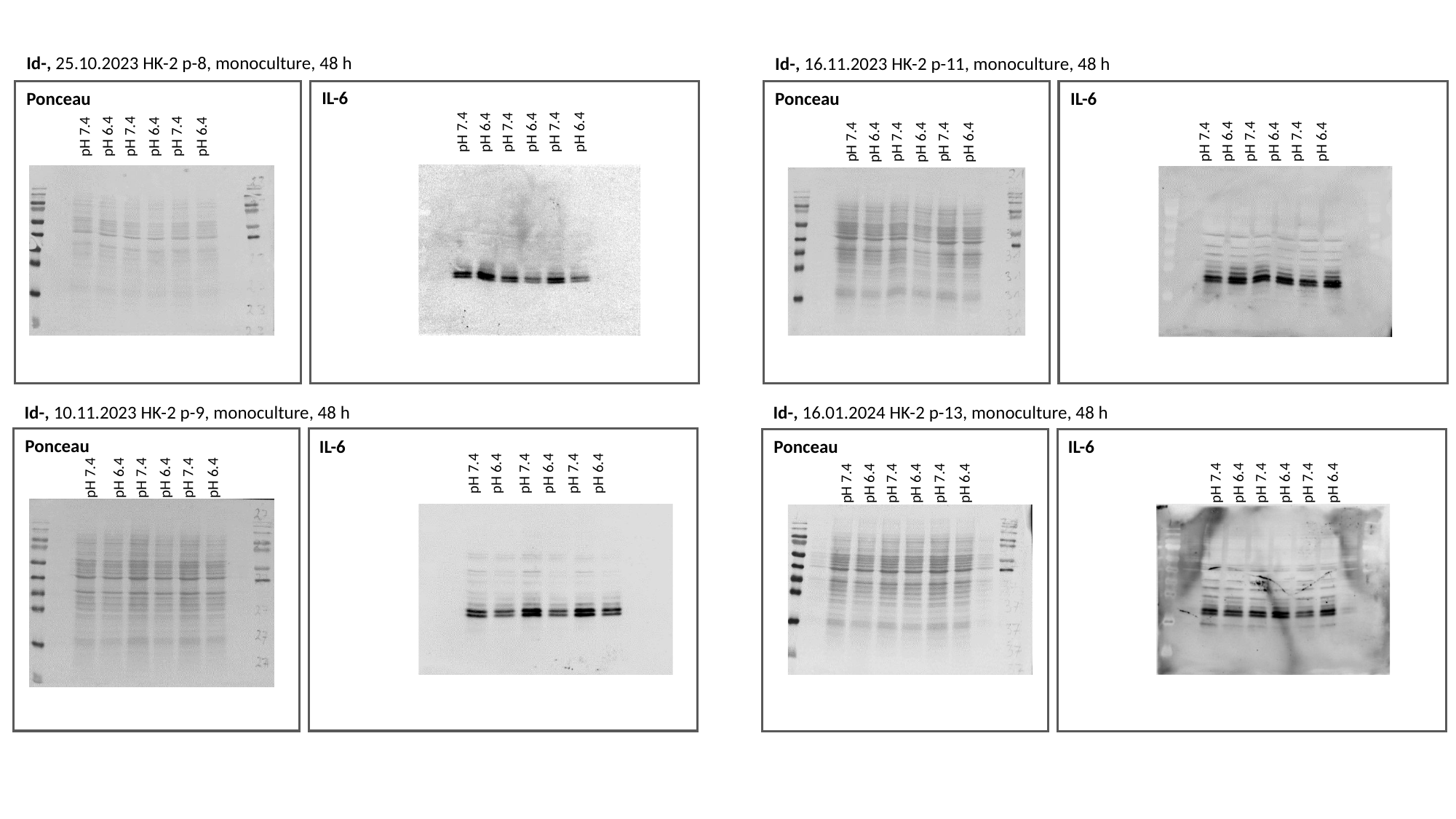

Id-, 25.10.2023 HK-2 p-8, monoculture, 48 h
Id-, 16.11.2023 HK-2 p-11, monoculture, 48 h
IL-6
Ponceau
IL-6
Ponceau
pH 6.4
pH 6.4
pH 6.4
pH 6.4
pH 6.4
pH 6.4
pH 7.4
pH 7.4
pH 7.4
pH 6.4
pH 6.4
pH 6.4
pH 6.4
pH 6.4
pH 6.4
pH 7.4
pH 7.4
pH 7.4
pH 7.4
pH 7.4
pH 7.4
pH 7.4
pH 7.4
pH 7.4
Id-, 10.11.2023 HK-2 p-9, monoculture, 48 h
Id-, 16.01.2024 HK-2 p-13, monoculture, 48 h
Ponceau
IL-6
Ponceau
IL-6
pH 6.4
pH 6.4
pH 6.4
pH 6.4
pH 6.4
pH 6.4
pH 7.4
pH 7.4
pH 7.4
pH 6.4
pH 6.4
pH 6.4
pH 6.4
pH 6.4
pH 6.4
pH 7.4
pH 7.4
pH 7.4
pH 7.4
pH 7.4
pH 7.4
pH 7.4
pH 7.4
pH 7.4

## Slide 6
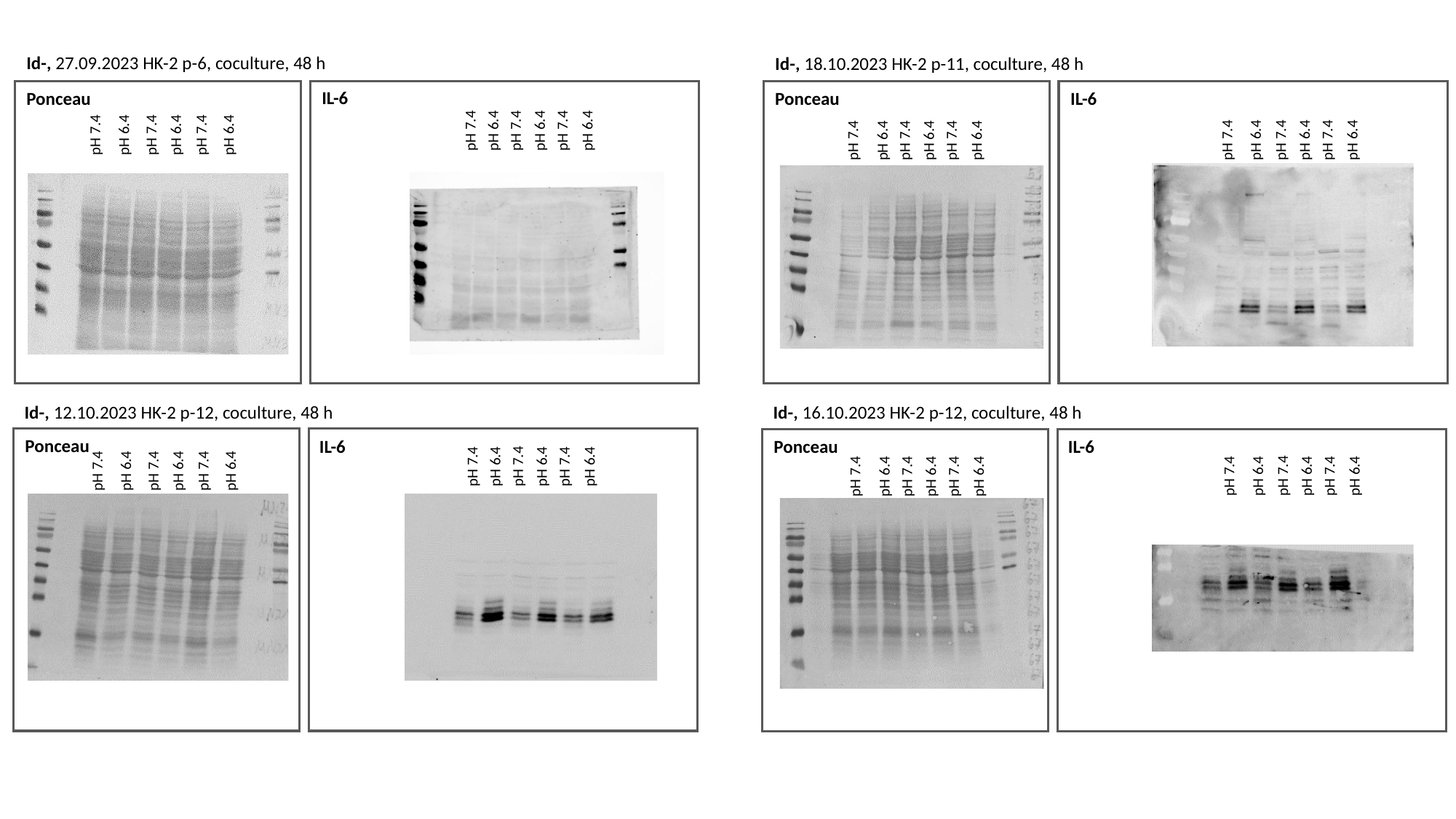

Id-, 27.09.2023 HK-2 p-6, coculture, 48 h
Id-, 18.10.2023 HK-2 p-11, coculture, 48 h
IL-6
Ponceau
IL-6
Ponceau
pH 6.4
pH 6.4
pH 6.4
pH 6.4
pH 6.4
pH 6.4
pH 7.4
pH 7.4
pH 7.4
pH 6.4
pH 6.4
pH 6.4
pH 6.4
pH 6.4
pH 6.4
pH 7.4
pH 7.4
pH 7.4
pH 7.4
pH 7.4
pH 7.4
pH 7.4
pH 7.4
pH 7.4
Id-, 12.10.2023 HK-2 p-12, coculture, 48 h
Id-, 16.10.2023 HK-2 p-12, coculture, 48 h
Ponceau
IL-6
Ponceau
IL-6
pH 6.4
pH 6.4
pH 6.4
pH 6.4
pH 6.4
pH 6.4
pH 7.4
pH 7.4
pH 7.4
pH 6.4
pH 6.4
pH 6.4
pH 6.4
pH 6.4
pH 6.4
pH 7.4
pH 7.4
pH 7.4
pH 7.4
pH 7.4
pH 7.4
pH 7.4
pH 7.4
pH 7.4

## Slide 7
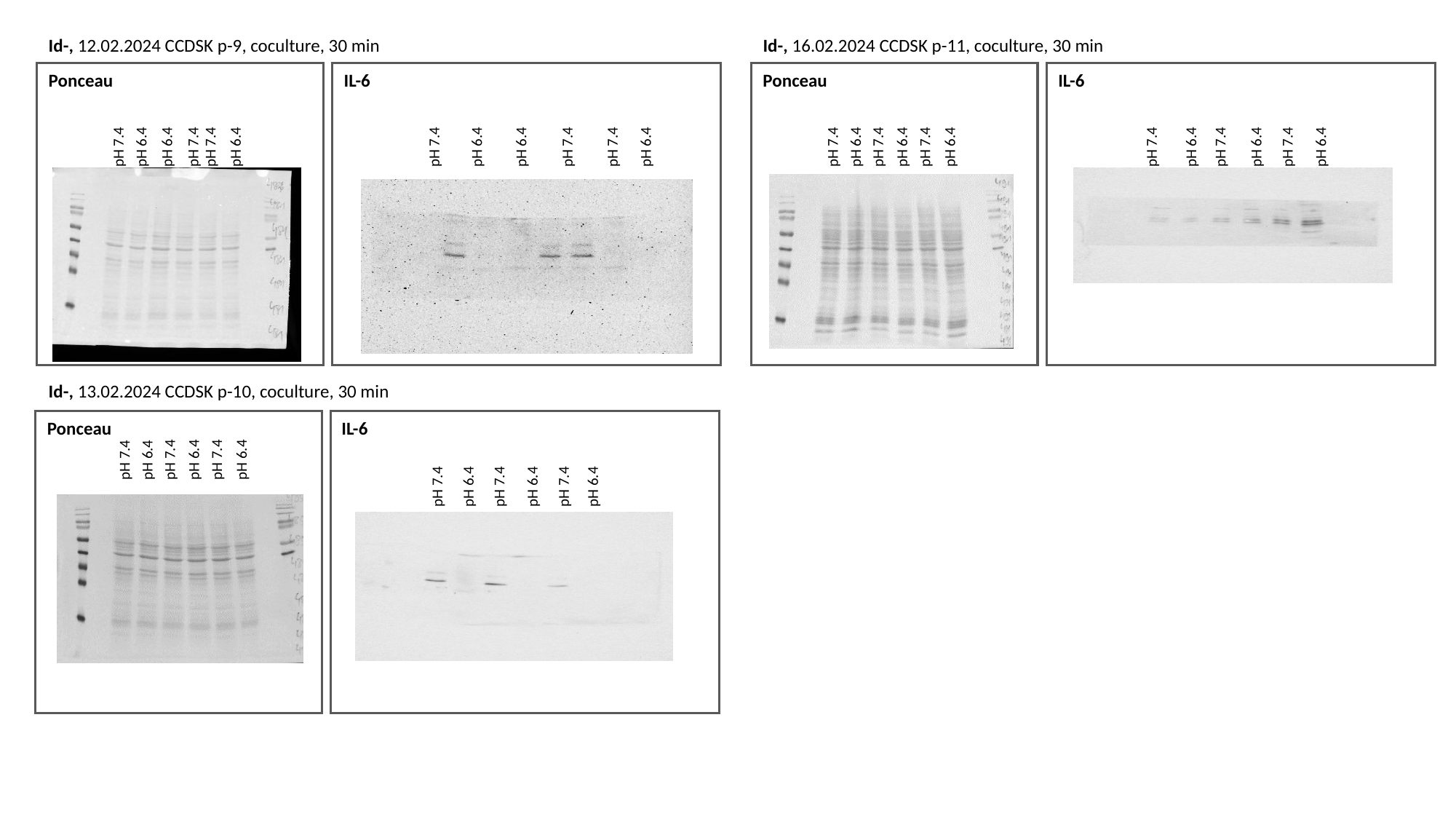

Id-, 12.02.2024 CCDSK p-9, coculture, 30 min
Id-, 16.02.2024 CCDSK p-11, coculture, 30 min
IL-6
IL-6
Ponceau
Ponceau
pH 6.4
pH 6.4
pH 6.4
pH 6.4
pH 6.4
pH 6.4
pH 6.4
pH 6.4
pH 6.4
pH 6.4
pH 6.4
pH 6.4
pH 7.4
pH 7.4
pH 7.4
pH 7.4
pH 7.4
pH 7.4
pH 7.4
pH 7.4
pH 7.4
pH 7.4
pH 7.4
pH 7.4
Id-, 13.02.2024 CCDSK p-10, coculture, 30 min
Ponceau
IL-6
pH 6.4
pH 6.4
pH 6.4
pH 7.4
pH 7.4
pH 7.4
pH 6.4
pH 6.4
pH 6.4
pH 7.4
pH 7.4
pH 7.4

## Slide 8
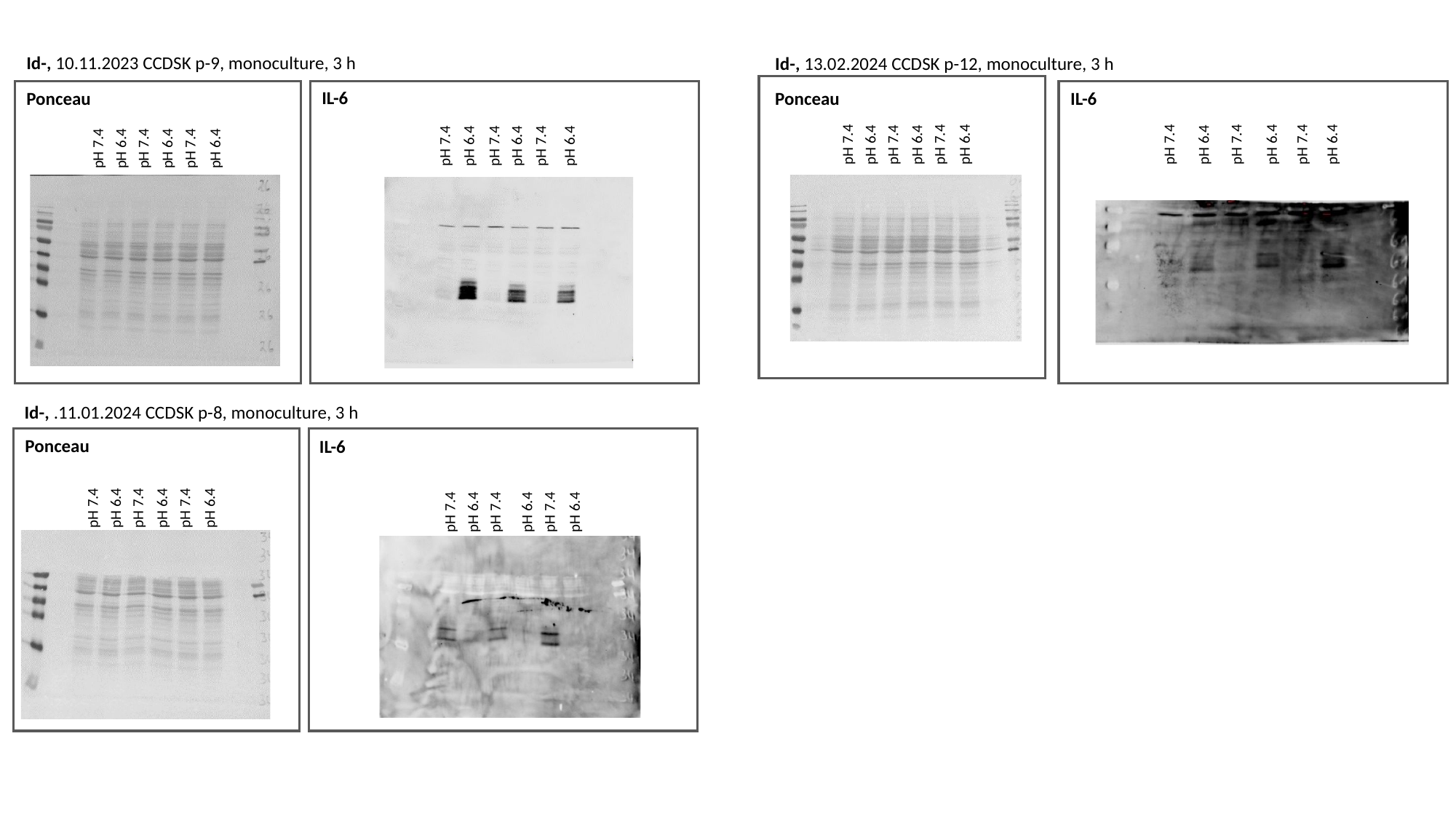

Id-, 10.11.2023 CCDSK p-9, monoculture, 3 h
Id-, 13.02.2024 CCDSK p-12, monoculture, 3 h
IL-6
Ponceau
IL-6
Ponceau
pH 6.4
pH 6.4
pH 6.4
pH 6.4
pH 6.4
pH 6.4
pH 6.4
pH 6.4
pH 6.4
pH 6.4
pH 6.4
pH 6.4
pH 7.4
pH 7.4
pH 7.4
pH 7.4
pH 7.4
pH 7.4
pH 7.4
pH 7.4
pH 7.4
pH 7.4
pH 7.4
pH 7.4
Id-, .11.01.2024 CCDSK p-8, monoculture, 3 h
Ponceau
IL-6
pH 6.4
pH 6.4
pH 6.4
pH 6.4
pH 6.4
pH 6.4
pH 7.4
pH 7.4
pH 7.4
pH 7.4
pH 7.4
pH 7.4

## Slide 9
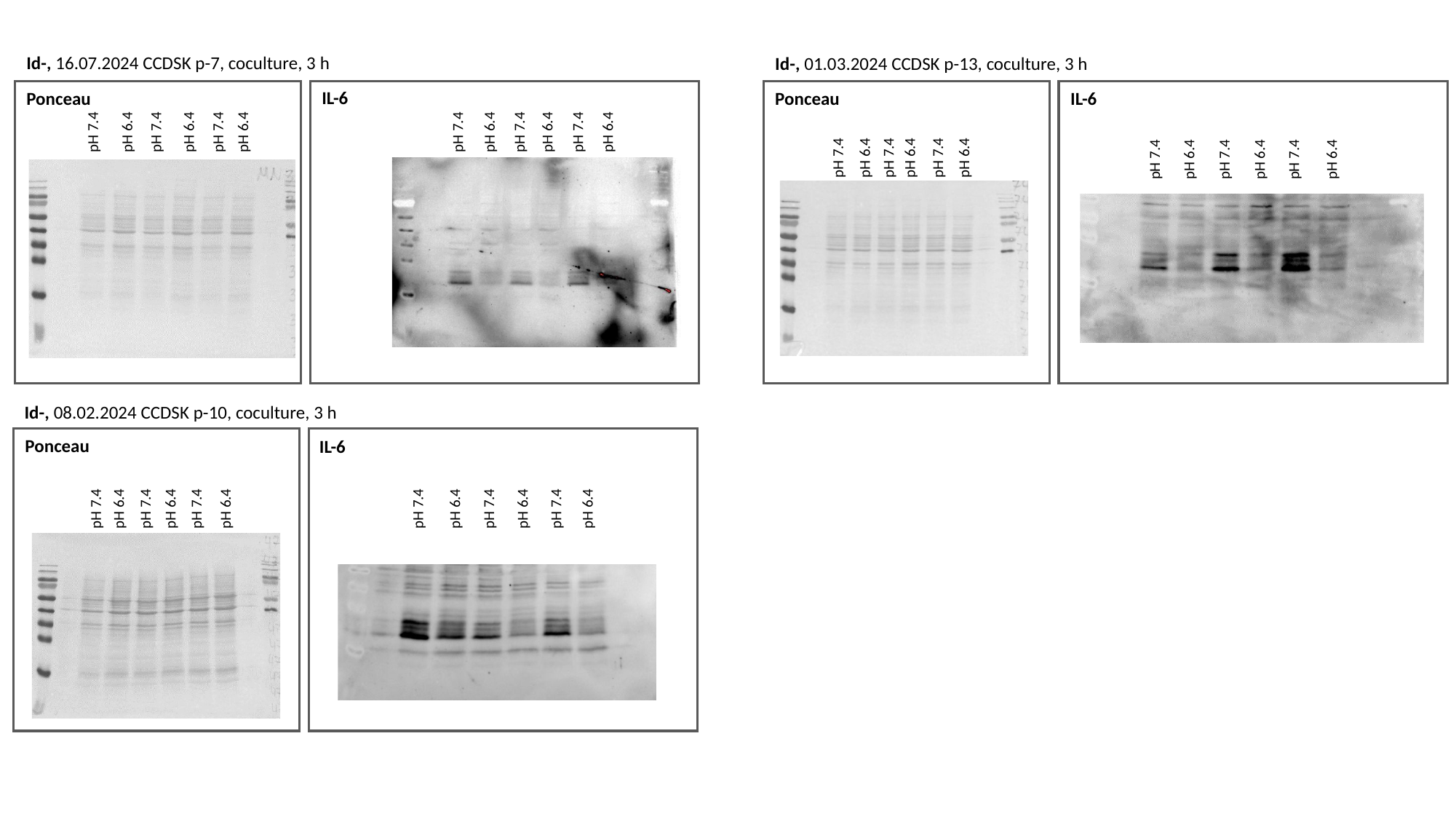

Id-, 16.07.2024 CCDSK p-7, coculture, 3 h
Id-, 01.03.2024 CCDSK p-13, coculture, 3 h
IL-6
Ponceau
IL-6
Ponceau
pH 6.4
pH 6.4
pH 6.4
pH 6.4
pH 6.4
pH 6.4
pH 7.4
pH 7.4
pH 7.4
pH 7.4
pH 7.4
pH 7.4
pH 6.4
pH 6.4
pH 6.4
pH 6.4
pH 6.4
pH 6.4
pH 7.4
pH 7.4
pH 7.4
pH 7.4
pH 7.4
pH 7.4
Id-, 08.02.2024 CCDSK p-10, coculture, 3 h
Ponceau
IL-6
pH 6.4
pH 6.4
pH 6.4
pH 6.4
pH 6.4
pH 6.4
pH 7.4
pH 7.4
pH 7.4
pH 7.4
pH 7.4
pH 7.4

## Slide 10
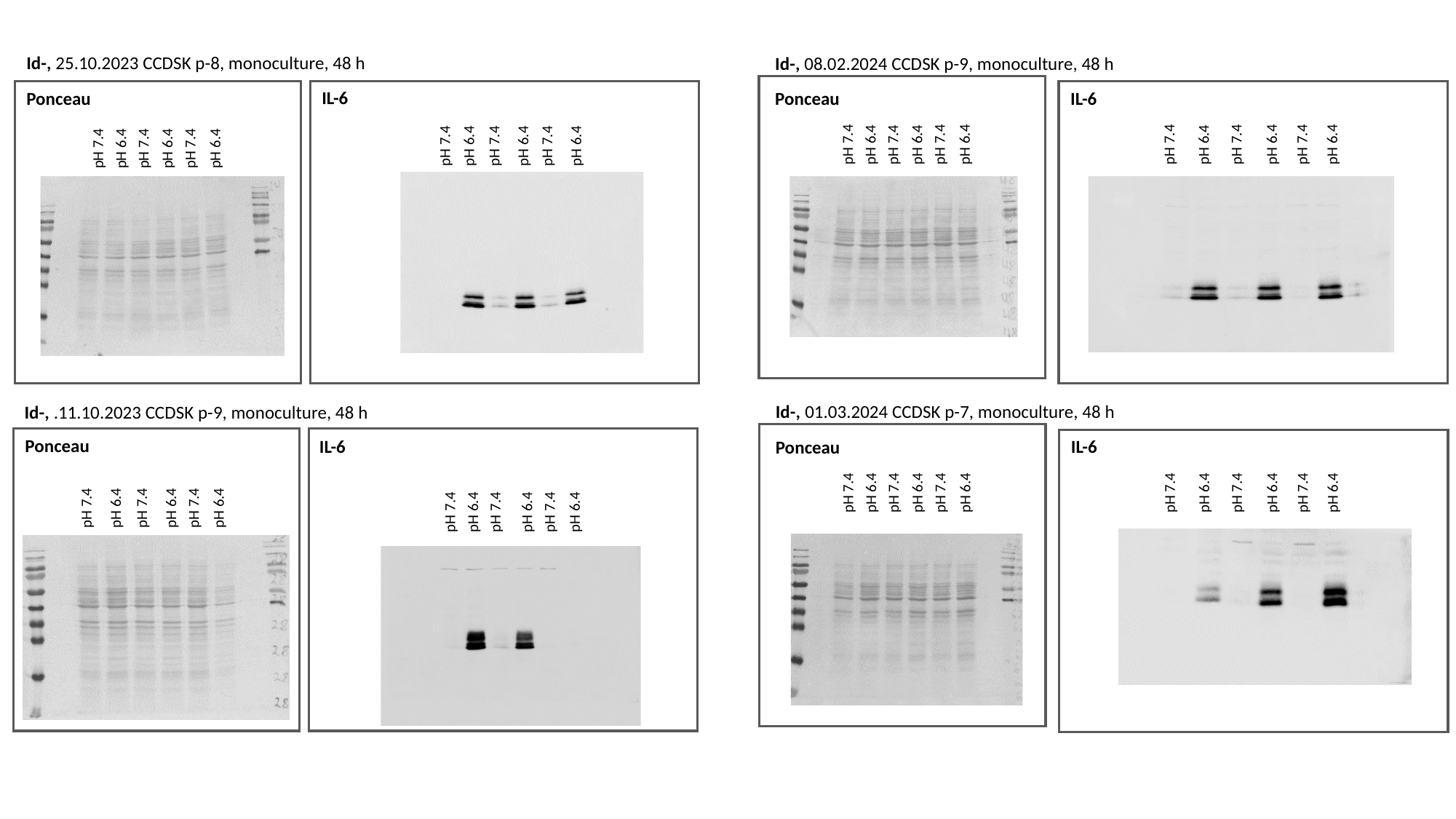

Id-, 25.10.2023 CCDSK p-8, monoculture, 48 h
Id-, 08.02.2024 CCDSK p-9, monoculture, 48 h
IL-6
Ponceau
IL-6
Ponceau
pH 6.4
pH 6.4
pH 6.4
pH 6.4
pH 6.4
pH 6.4
pH 6.4
pH 6.4
pH 6.4
pH 6.4
pH 6.4
pH 6.4
pH 7.4
pH 7.4
pH 7.4
pH 7.4
pH 7.4
pH 7.4
pH 7.4
pH 7.4
pH 7.4
pH 7.4
pH 7.4
pH 7.4
Id-, 01.03.2024 CCDSK p-7, monoculture, 48 h
Id-, .11.10.2023 CCDSK p-9, monoculture, 48 h
Ponceau
IL-6
IL-6
Ponceau
pH 6.4
pH 6.4
pH 6.4
pH 6.4
pH 6.4
pH 6.4
pH 7.4
pH 7.4
pH 7.4
pH 7.4
pH 7.4
pH 7.4
pH 6.4
pH 6.4
pH 6.4
pH 6.4
pH 6.4
pH 6.4
pH 7.4
pH 7.4
pH 7.4
pH 7.4
pH 7.4
pH 7.4

## Slide 11
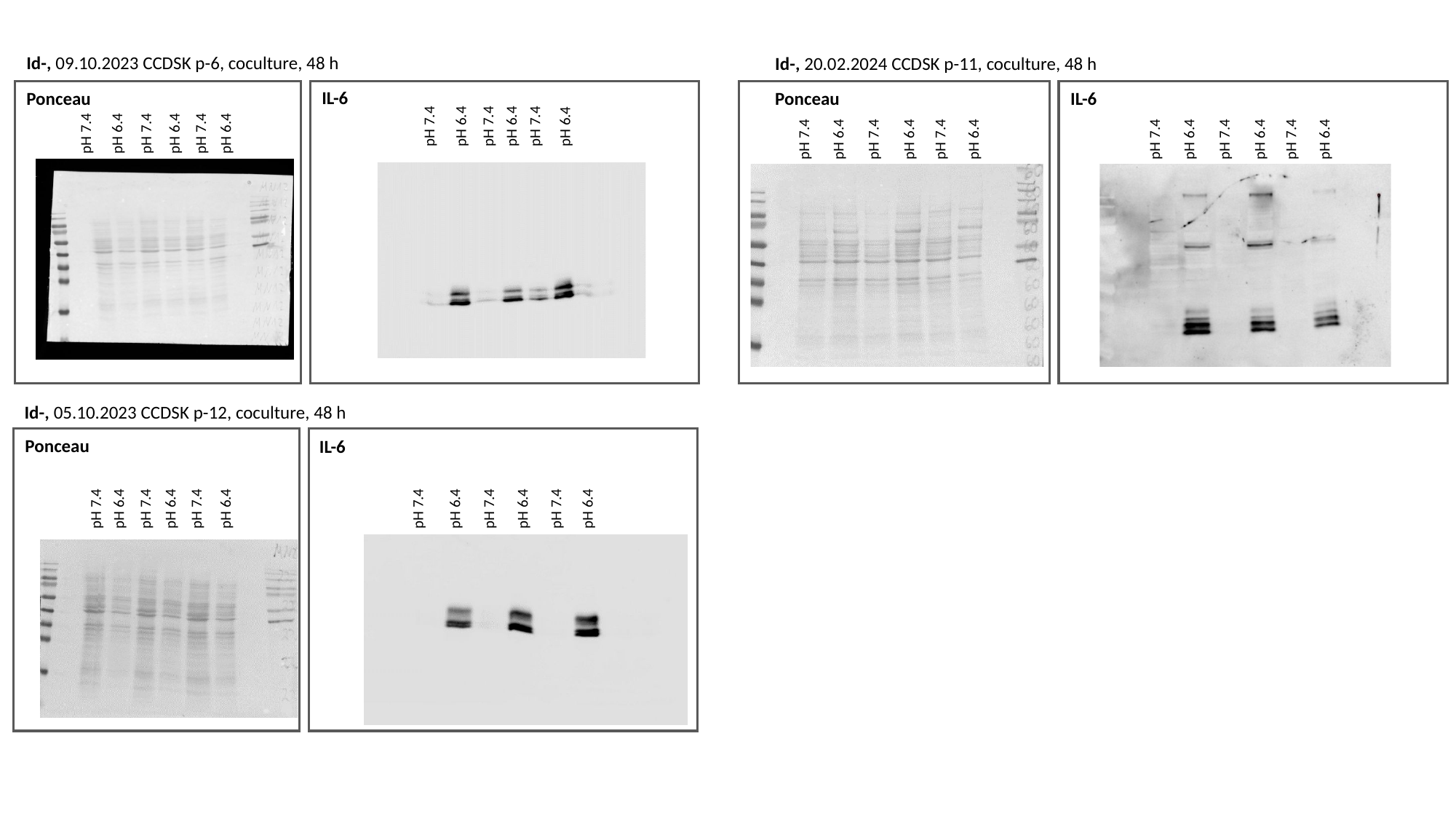

Id-, 09.10.2023 CCDSK p-6, coculture, 48 h
Id-, 20.02.2024 CCDSK p-11, coculture, 48 h
IL-6
Ponceau
IL-6
Ponceau
pH 6.4
pH 6.4
pH 6.4
pH 6.4
pH 6.4
pH 6.4
pH 7.4
pH 7.4
pH 7.4
pH 6.4
pH 6.4
pH 6.4
pH 6.4
pH 6.4
pH 6.4
pH 7.4
pH 7.4
pH 7.4
pH 7.4
pH 7.4
pH 7.4
pH 7.4
pH 7.4
pH 7.4
Id-, 05.10.2023 CCDSK p-12, coculture, 48 h
Ponceau
IL-6
pH 6.4
pH 6.4
pH 6.4
pH 6.4
pH 6.4
pH 6.4
pH 7.4
pH 7.4
pH 7.4
pH 7.4
pH 7.4
pH 7.4

## Slide 12
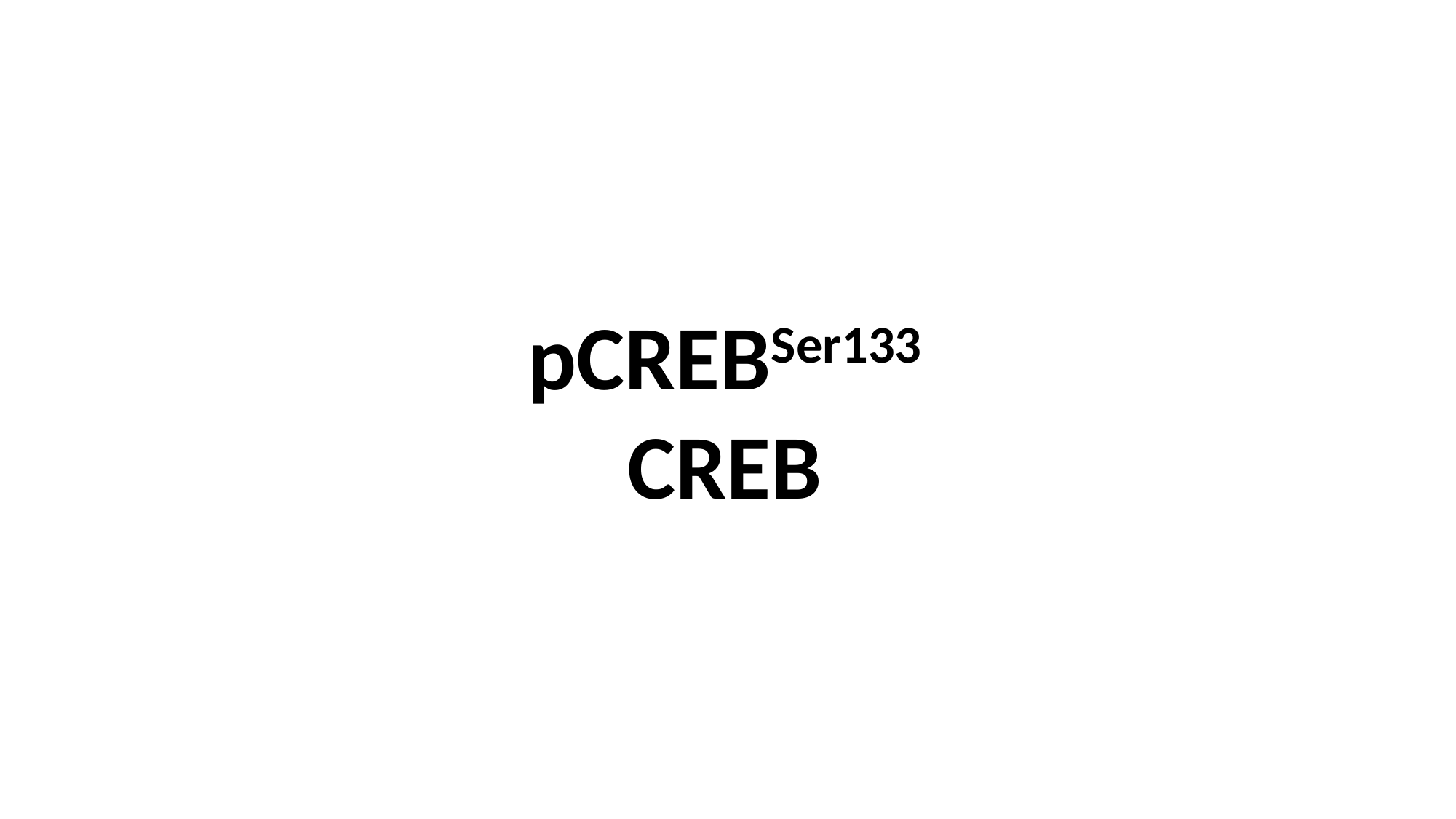

pCREBSer133
CREB

## Slide 13
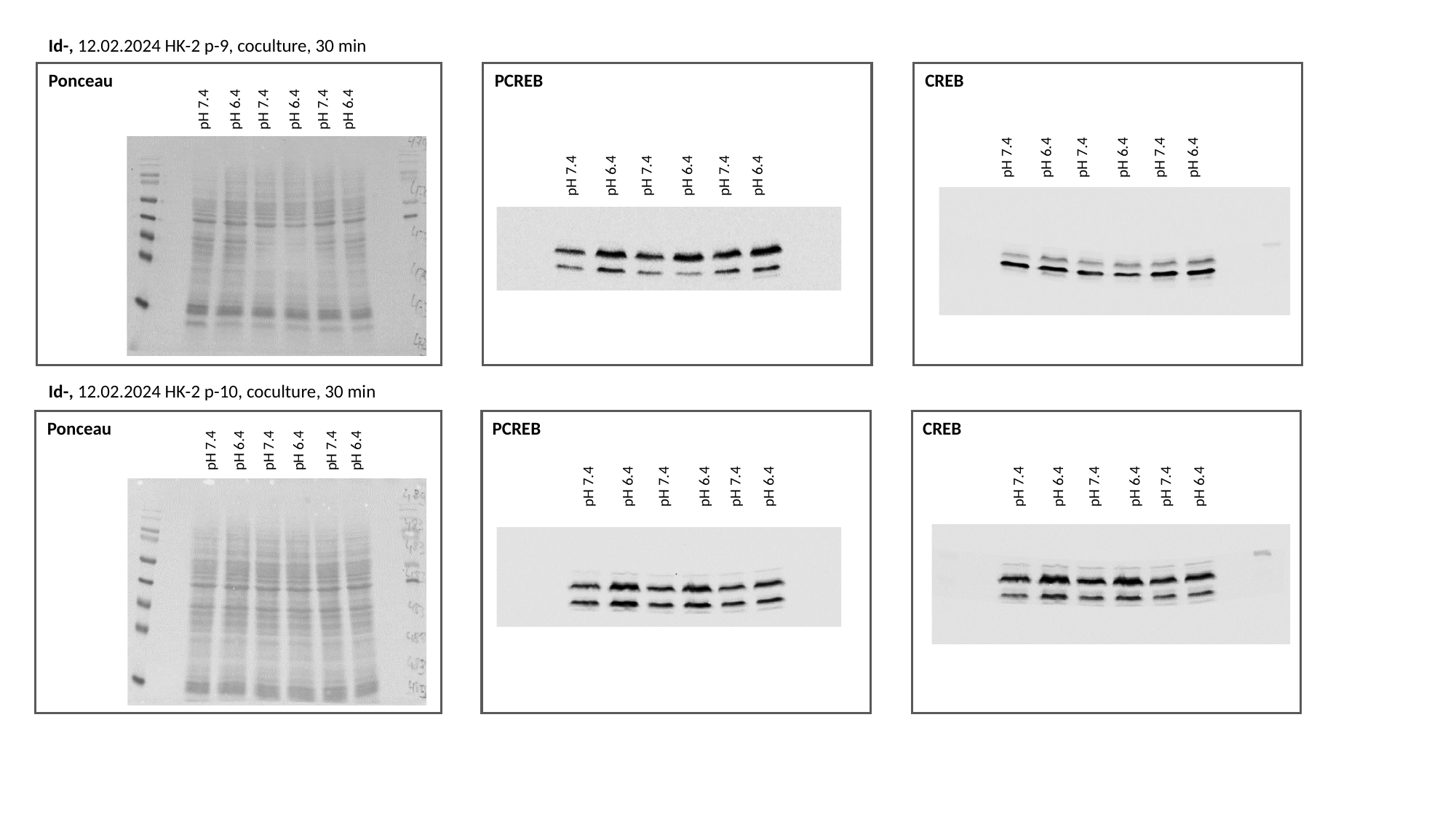

Id-, 12.02.2024 HK-2 p-9, coculture, 30 min
PCREB
CREB
Ponceau
pH 6.4
pH 6.4
pH 6.4
pH 7.4
pH 7.4
pH 7.4
pH 6.4
pH 6.4
pH 6.4
pH 7.4
pH 7.4
pH 7.4
pH 6.4
pH 6.4
pH 6.4
pH 7.4
pH 7.4
pH 7.4
Id-, 12.02.2024 HK-2 p-10, coculture, 30 min
Ponceau
PCREB
CREB
pH 6.4
pH 6.4
pH 6.4
pH 7.4
pH 7.4
pH 7.4
pH 6.4
pH 6.4
pH 6.4
pH 6.4
pH 6.4
pH 6.4
pH 7.4
pH 7.4
pH 7.4
pH 7.4
pH 7.4
pH 7.4

## Slide 14
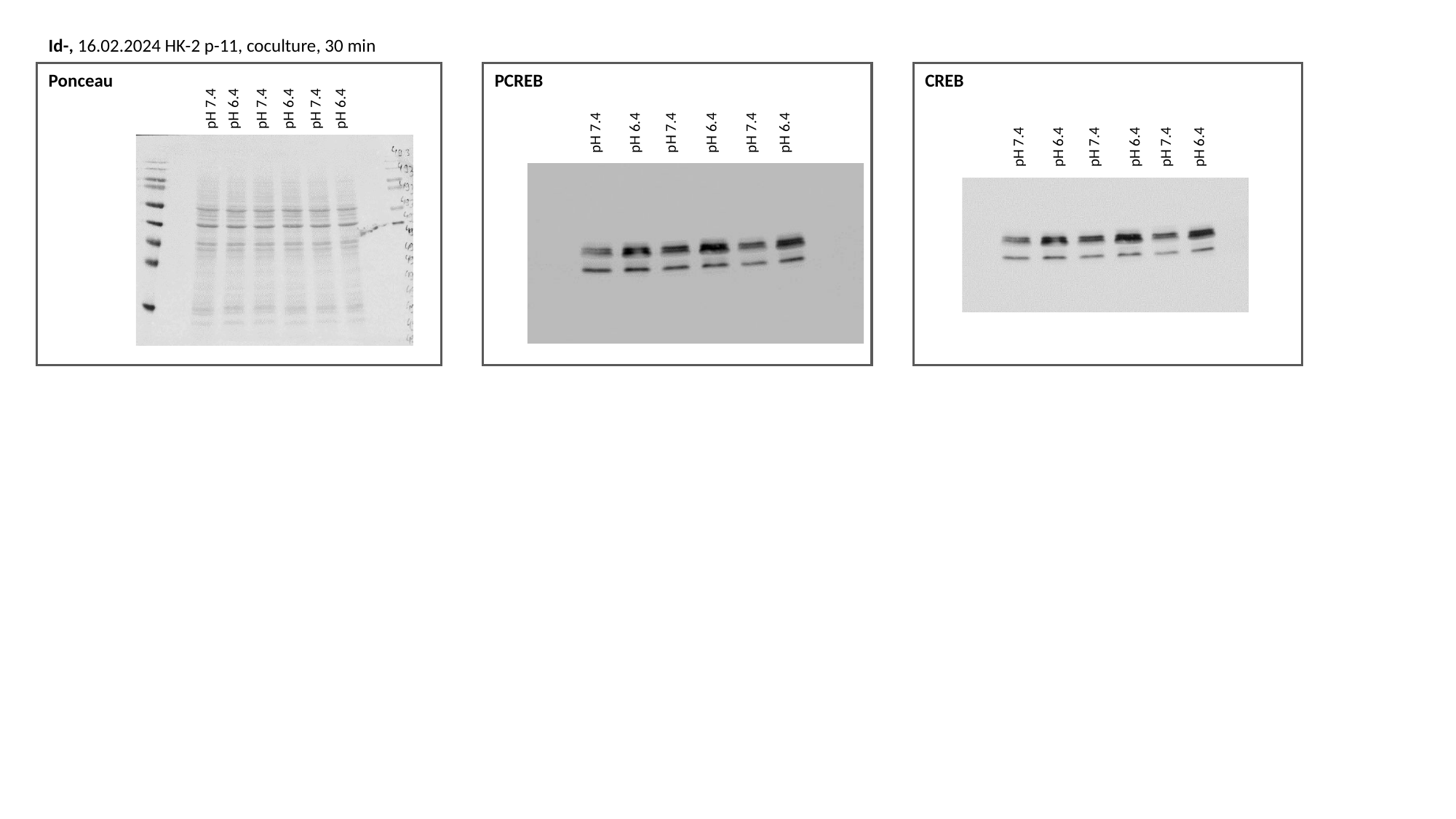

Id-, 16.02.2024 HK-2 p-11, coculture, 30 min
PCREB
CREB
Ponceau
pH 6.4
pH 6.4
pH 6.4
pH 7.4
pH 7.4
pH 7.4
pH 6.4
pH 6.4
pH 6.4
pH 7.4
pH 7.4
pH 7.4
pH 6.4
pH 6.4
pH 6.4
pH 7.4
pH 7.4
pH 7.4

## Slide 15
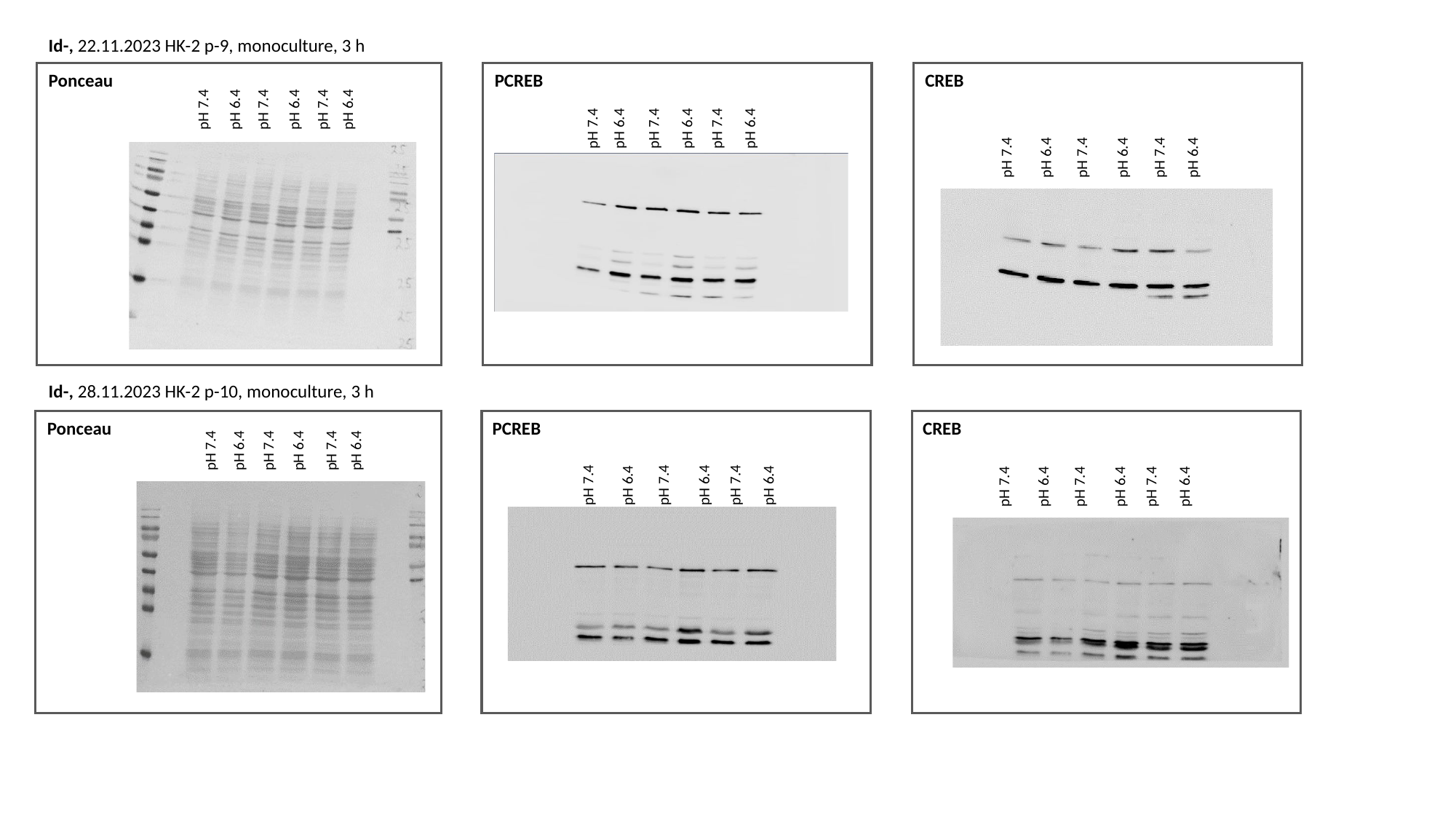

Id-, 22.11.2023 HK-2 p-9, monoculture, 3 h
PCREB
CREB
Ponceau
pH 6.4
pH 6.4
pH 6.4
pH 7.4
pH 7.4
pH 7.4
pH 6.4
pH 6.4
pH 6.4
pH 7.4
pH 7.4
pH 7.4
pH 6.4
pH 6.4
pH 6.4
pH 7.4
pH 7.4
pH 7.4
Id-, 28.11.2023 HK-2 p-10, monoculture, 3 h
Ponceau
PCREB
CREB
pH 6.4
pH 6.4
pH 6.4
pH 7.4
pH 7.4
pH 7.4
pH 6.4
pH 6.4
pH 6.4
pH 6.4
pH 6.4
pH 6.4
pH 7.4
pH 7.4
pH 7.4
pH 7.4
pH 7.4
pH 7.4

## Slide 16
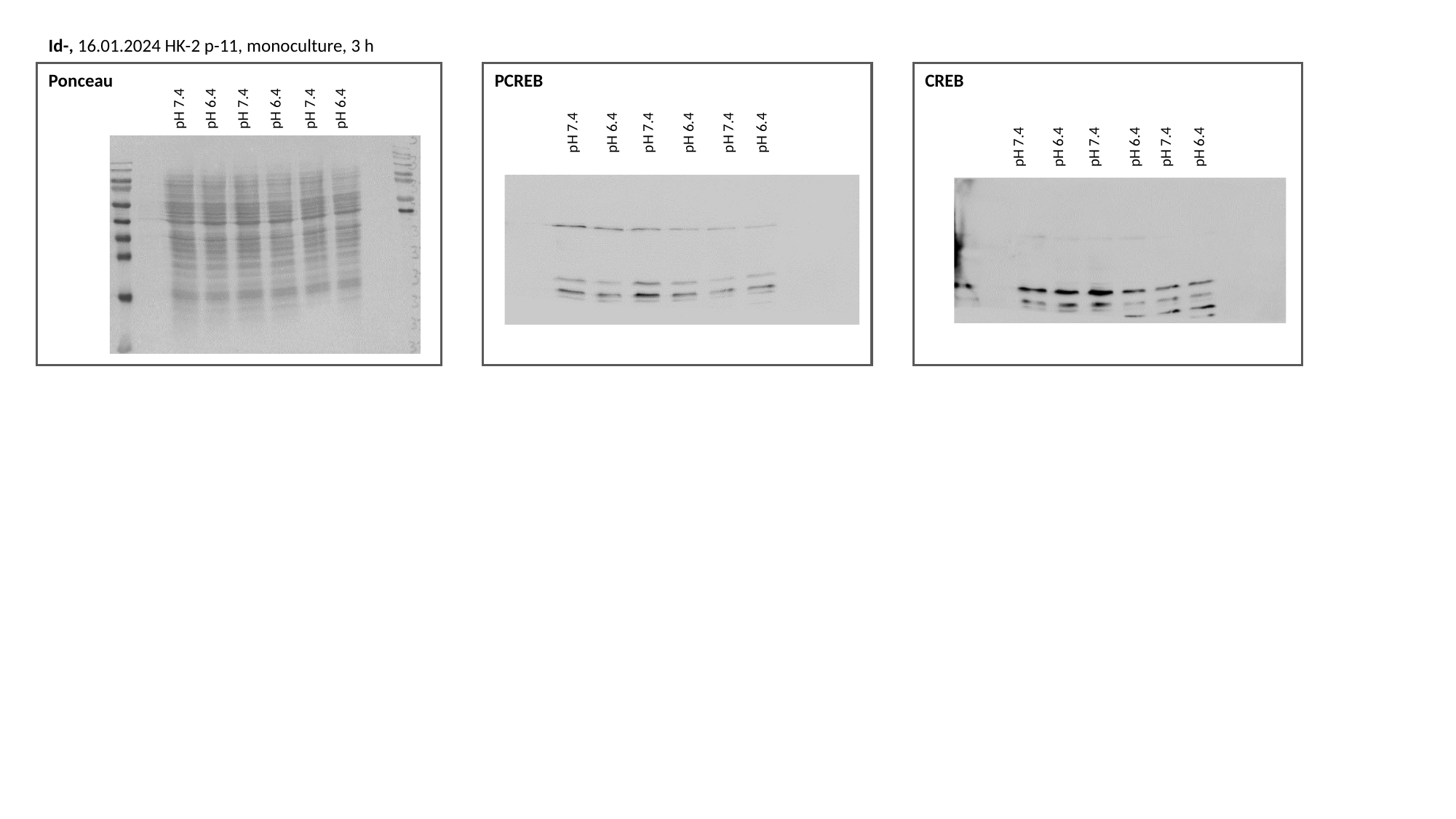

Id-, 16.01.2024 HK-2 p-11, monoculture, 3 h
PCREB
CREB
Ponceau
pH 6.4
pH 6.4
pH 6.4
pH 7.4
pH 7.4
pH 7.4
pH 6.4
pH 6.4
pH 6.4
pH 7.4
pH 7.4
pH 7.4
pH 6.4
pH 6.4
pH 6.4
pH 7.4
pH 7.4
pH 7.4

## Slide 17
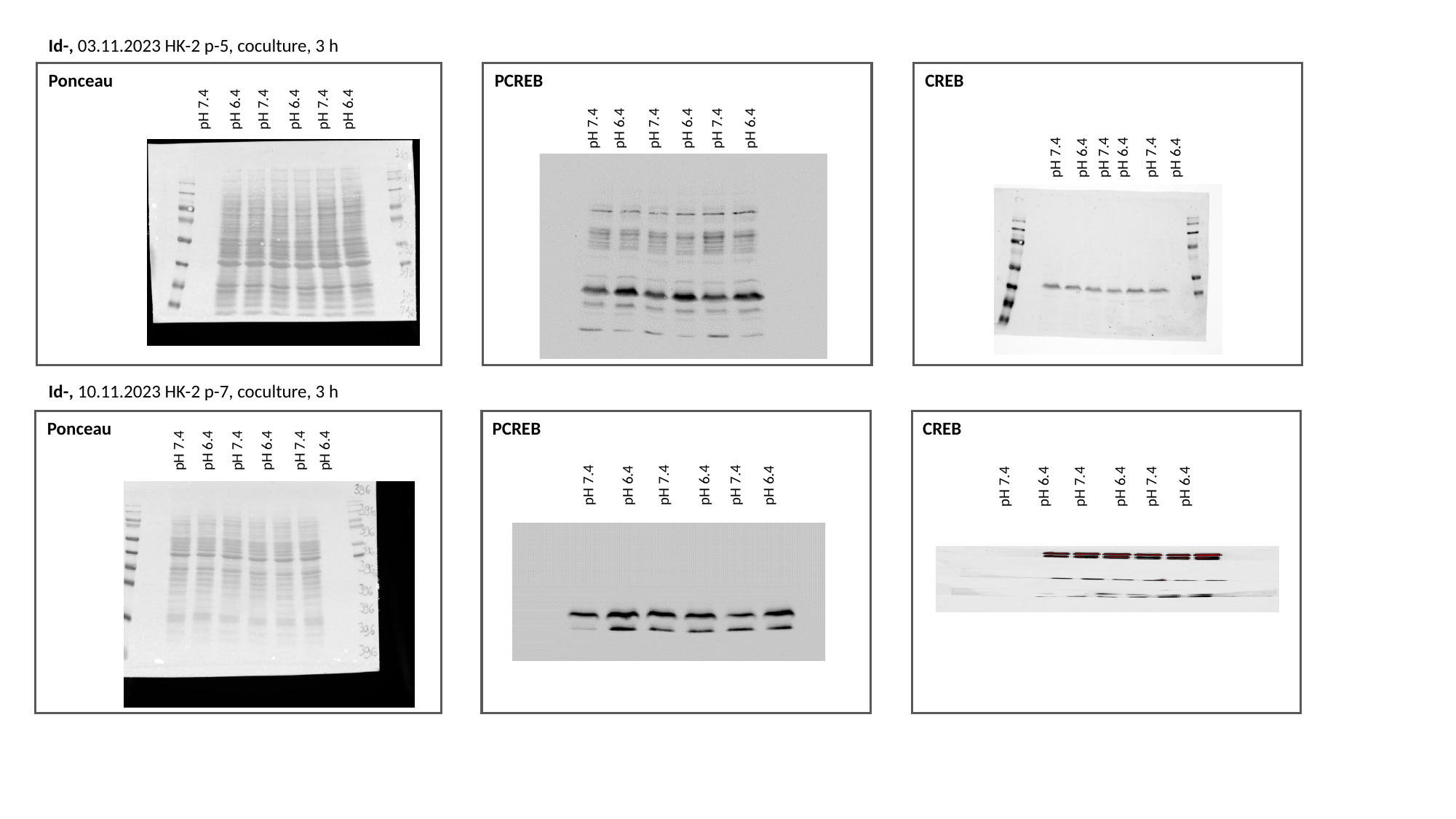

Id-, 03.11.2023 HK-2 p-5, coculture, 3 h
PCREB
CREB
Ponceau
pH 6.4
pH 6.4
pH 6.4
pH 7.4
pH 7.4
pH 7.4
pH 6.4
pH 6.4
pH 6.4
pH 7.4
pH 7.4
pH 7.4
pH 6.4
pH 6.4
pH 6.4
pH 7.4
pH 7.4
pH 7.4
Id-, 10.11.2023 HK-2 p-7, coculture, 3 h
Ponceau
PCREB
CREB
pH 6.4
pH 6.4
pH 6.4
pH 7.4
pH 7.4
pH 7.4
pH 6.4
pH 6.4
pH 6.4
pH 6.4
pH 6.4
pH 6.4
pH 7.4
pH 7.4
pH 7.4
pH 7.4
pH 7.4
pH 7.4

## Slide 18
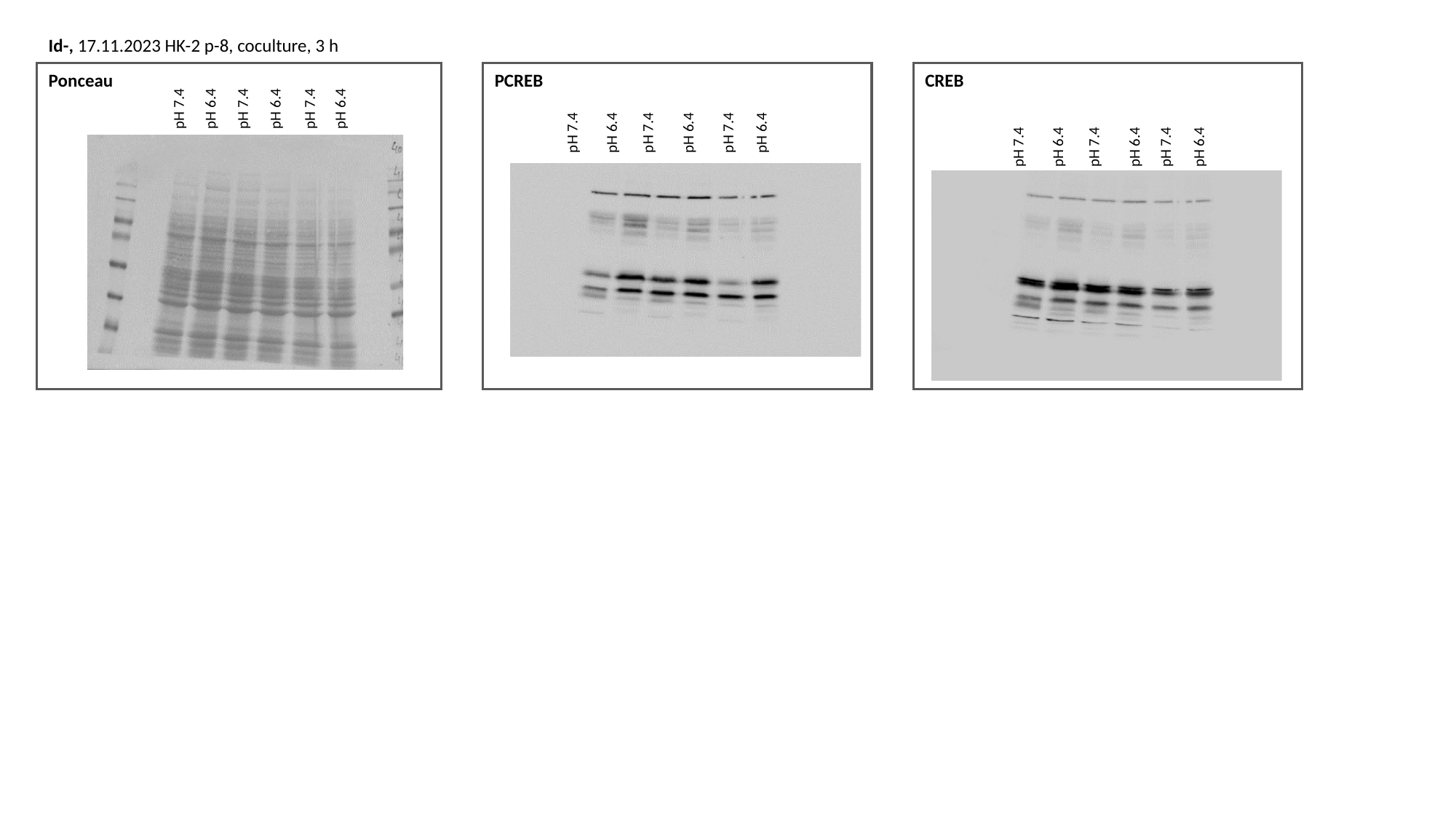

Id-, 17.11.2023 HK-2 p-8, coculture, 3 h
PCREB
CREB
Ponceau
pH 6.4
pH 6.4
pH 6.4
pH 7.4
pH 7.4
pH 7.4
pH 6.4
pH 6.4
pH 6.4
pH 7.4
pH 7.4
pH 7.4
pH 6.4
pH 6.4
pH 6.4
pH 7.4
pH 7.4
pH 7.4

## Slide 19
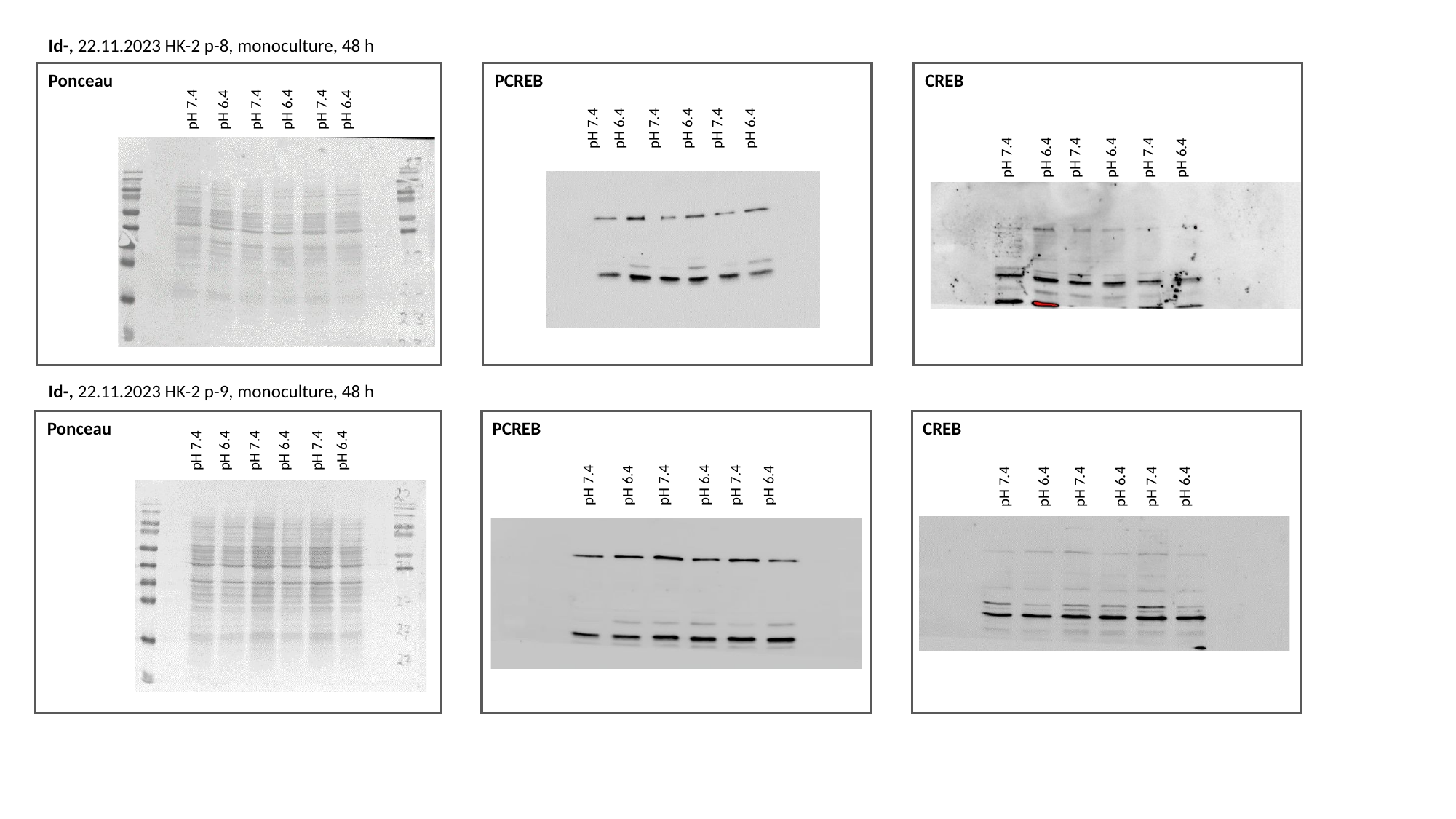

Id-, 22.11.2023 HK-2 p-8, monoculture, 48 h
PCREB
CREB
Ponceau
pH 6.4
pH 6.4
pH 6.4
pH 7.4
pH 7.4
pH 7.4
pH 6.4
pH 6.4
pH 6.4
pH 7.4
pH 7.4
pH 7.4
pH 6.4
pH 6.4
pH 6.4
pH 7.4
pH 7.4
pH 7.4
Id-, 22.11.2023 HK-2 p-9, monoculture, 48 h
Ponceau
PCREB
CREB
pH 6.4
pH 6.4
pH 6.4
pH 7.4
pH 7.4
pH 7.4
pH 6.4
pH 6.4
pH 6.4
pH 6.4
pH 6.4
pH 6.4
pH 7.4
pH 7.4
pH 7.4
pH 7.4
pH 7.4
pH 7.4

## Slide 20
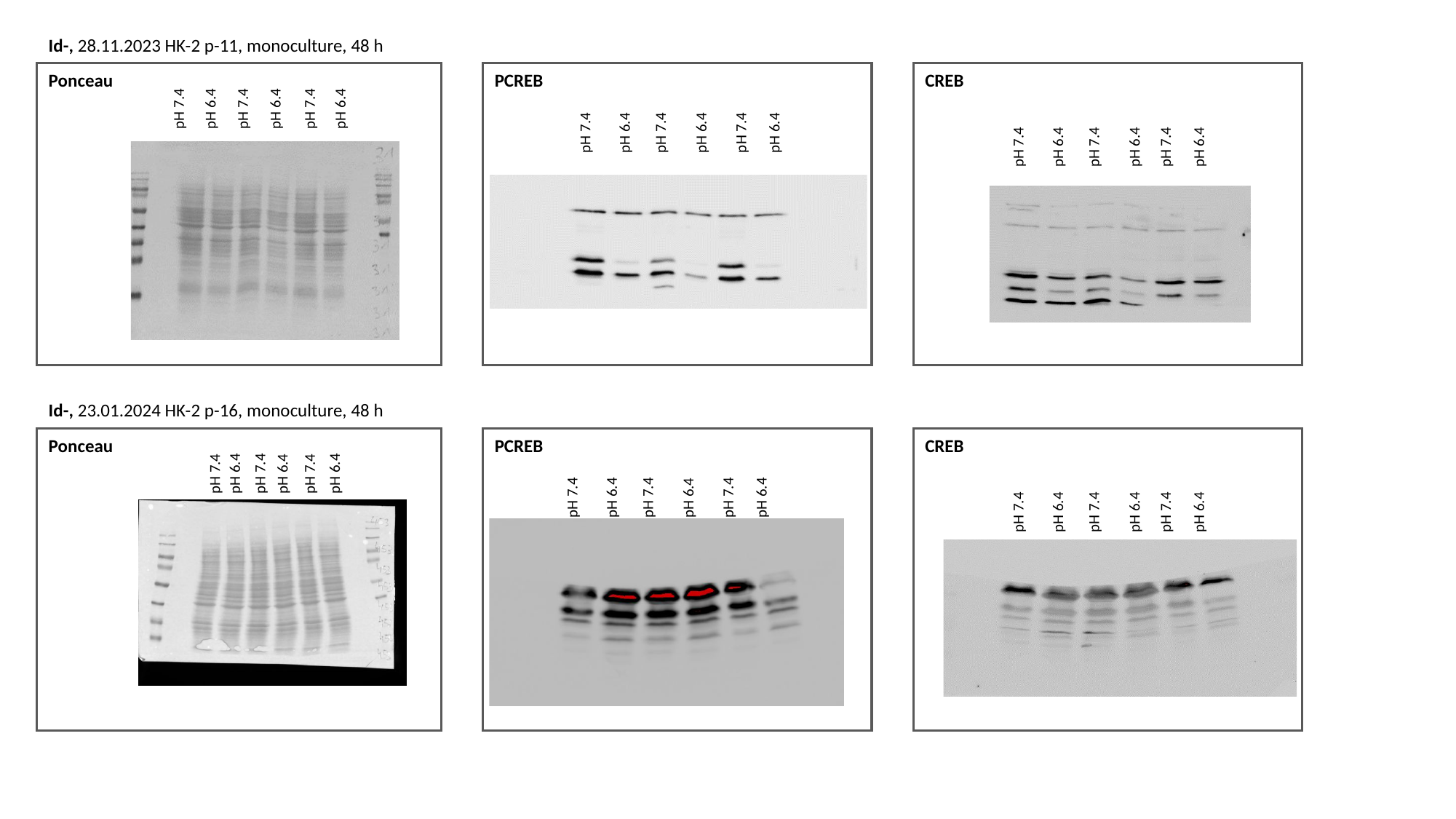

Id-, 28.11.2023 HK-2 p-11, monoculture, 48 h
PCREB
CREB
Ponceau
pH 6.4
pH 6.4
pH 6.4
pH 7.4
pH 7.4
pH 7.4
pH 6.4
pH 6.4
pH 6.4
pH 7.4
pH 7.4
pH 7.4
pH 6.4
pH 6.4
pH 6.4
pH 7.4
pH 7.4
pH 7.4
Id-, 23.01.2024 HK-2 p-16, monoculture, 48 h
PCREB
CREB
Ponceau
pH 6.4
pH 6.4
pH 6.4
pH 7.4
pH 7.4
pH 7.4
pH 6.4
pH 6.4
pH 6.4
pH 7.4
pH 7.4
pH 7.4
pH 6.4
pH 6.4
pH 6.4
pH 7.4
pH 7.4
pH 7.4

## Slide 21
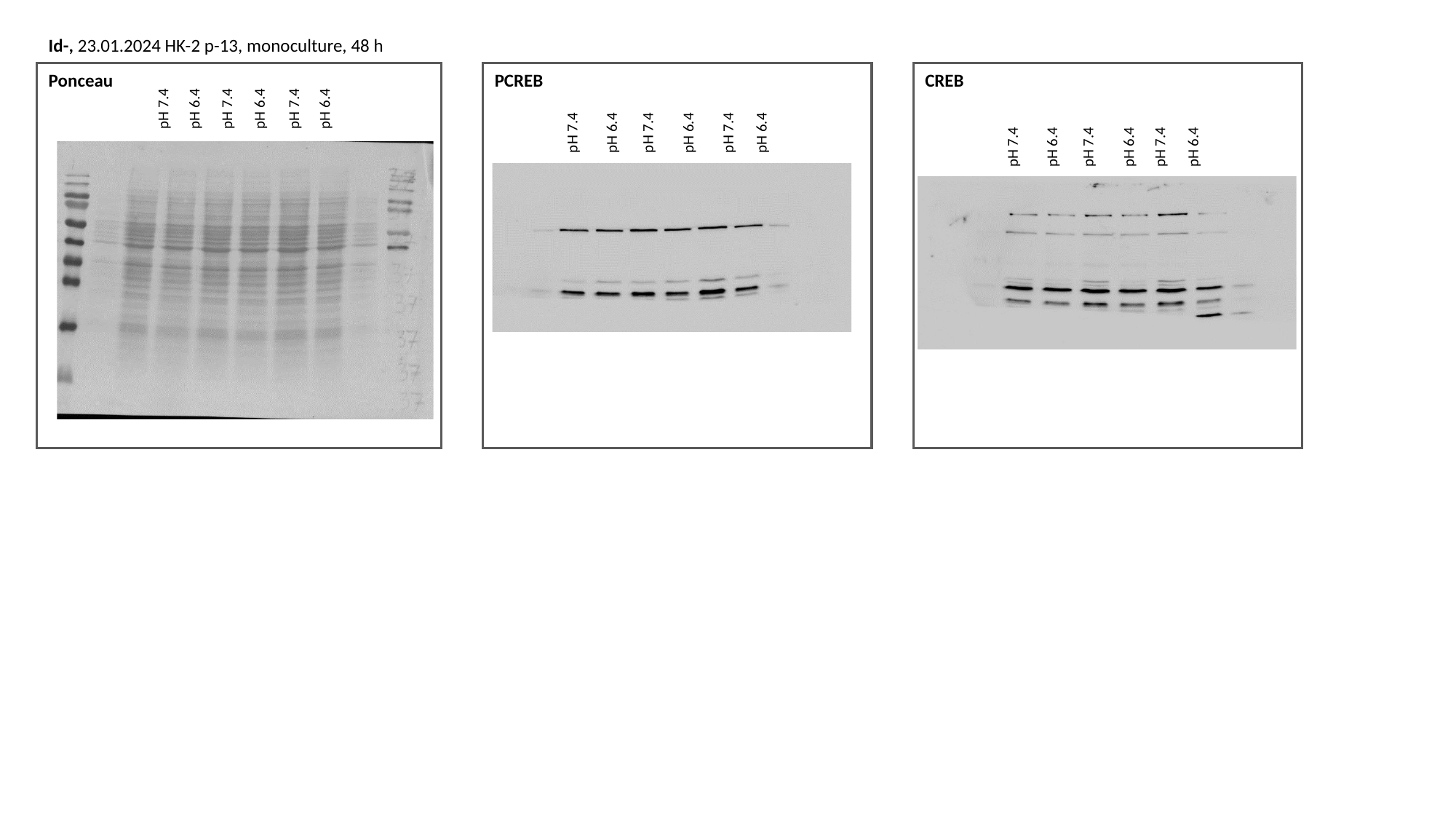

Id-, 23.01.2024 HK-2 p-13, monoculture, 48 h
PCREB
CREB
Ponceau
pH 6.4
pH 6.4
pH 6.4
pH 7.4
pH 7.4
pH 7.4
pH 6.4
pH 6.4
pH 6.4
pH 7.4
pH 7.4
pH 7.4
pH 6.4
pH 6.4
pH 6.4
pH 7.4
pH 7.4
pH 7.4

## Slide 22
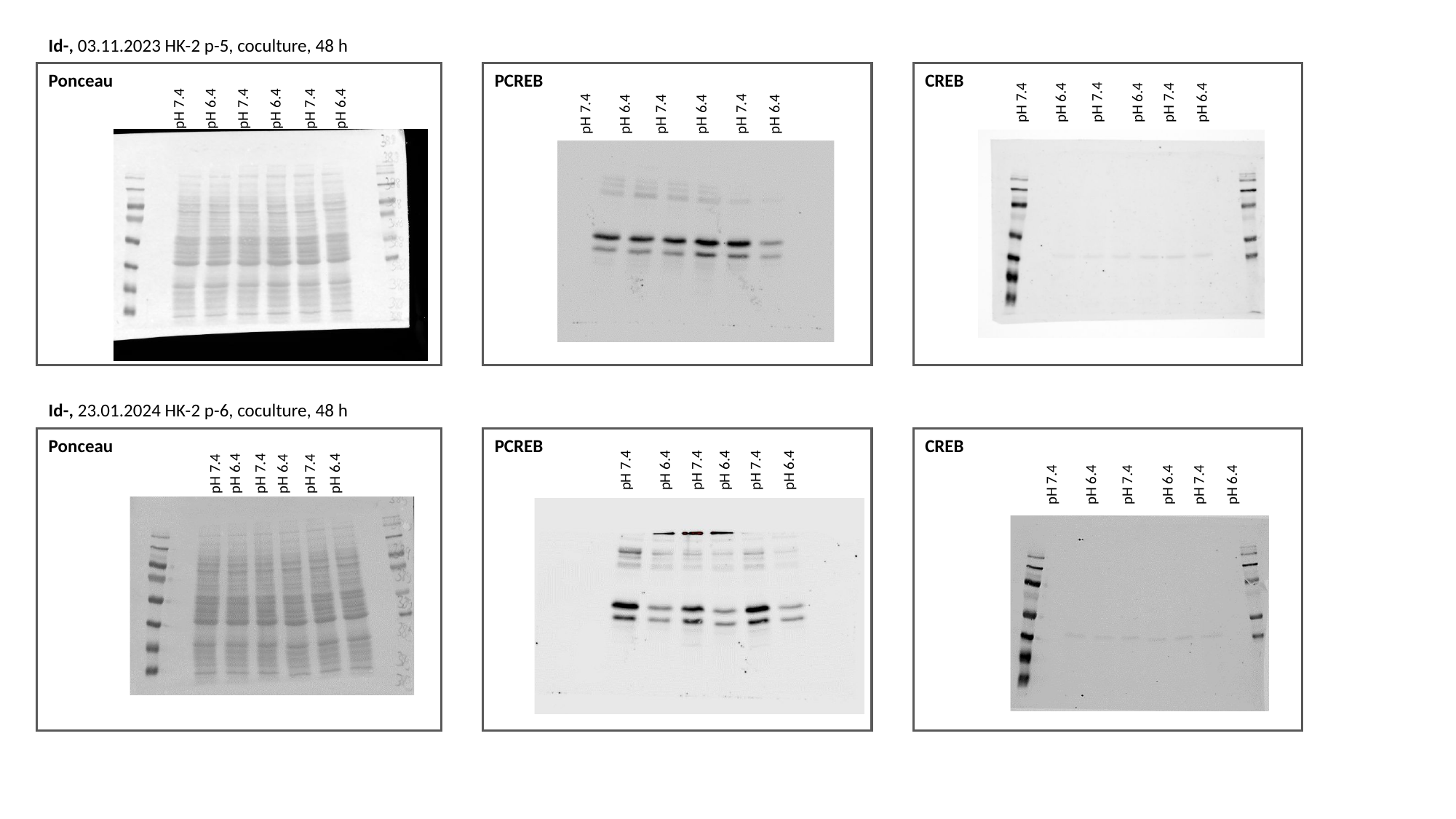

Id-, 03.11.2023 HK-2 p-5, coculture, 48 h
PCREB
CREB
Ponceau
pH 6.4
pH 6.4
pH 6.4
pH 6.4
pH 6.4
pH 6.4
pH 7.4
pH 7.4
pH 7.4
pH 6.4
pH 6.4
pH 6.4
pH 7.4
pH 7.4
pH 7.4
pH 7.4
pH 7.4
pH 7.4
Id-, 23.01.2024 HK-2 p-6, coculture, 48 h
PCREB
CREB
Ponceau
pH 6.4
pH 6.4
pH 6.4
pH 6.4
pH 6.4
pH 6.4
pH 7.4
pH 7.4
pH 7.4
pH 7.4
pH 7.4
pH 7.4
pH 6.4
pH 6.4
pH 6.4
pH 7.4
pH 7.4
pH 7.4

## Slide 23
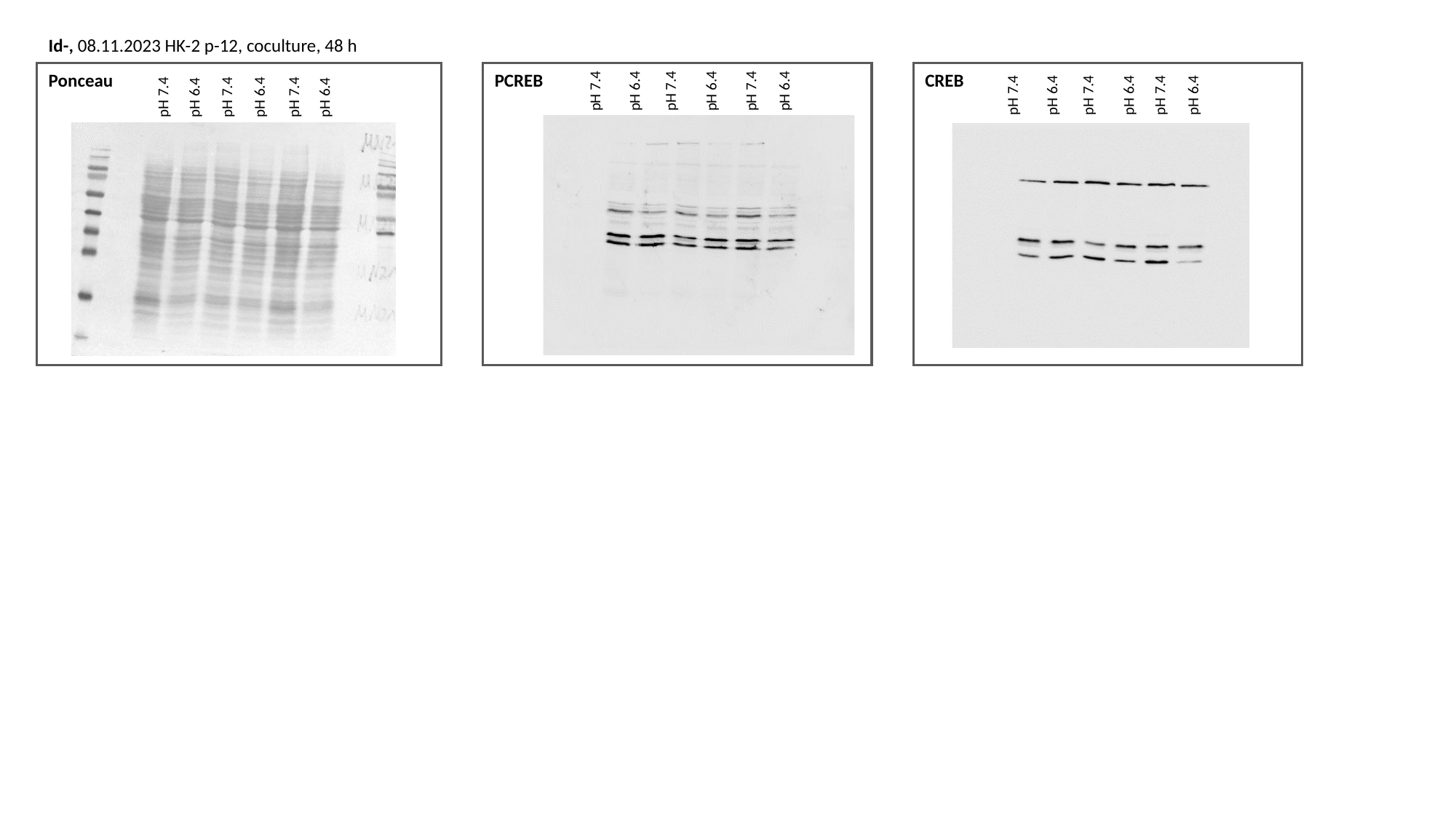

Id-, 08.11.2023 HK-2 p-12, coculture, 48 h
PCREB
CREB
Ponceau
pH 6.4
pH 6.4
pH 6.4
pH 6.4
pH 6.4
pH 6.4
pH 6.4
pH 6.4
pH 6.4
pH 7.4
pH 7.4
pH 7.4
pH 7.4
pH 7.4
pH 7.4
pH 7.4
pH 7.4
pH 7.4

## Slide 24
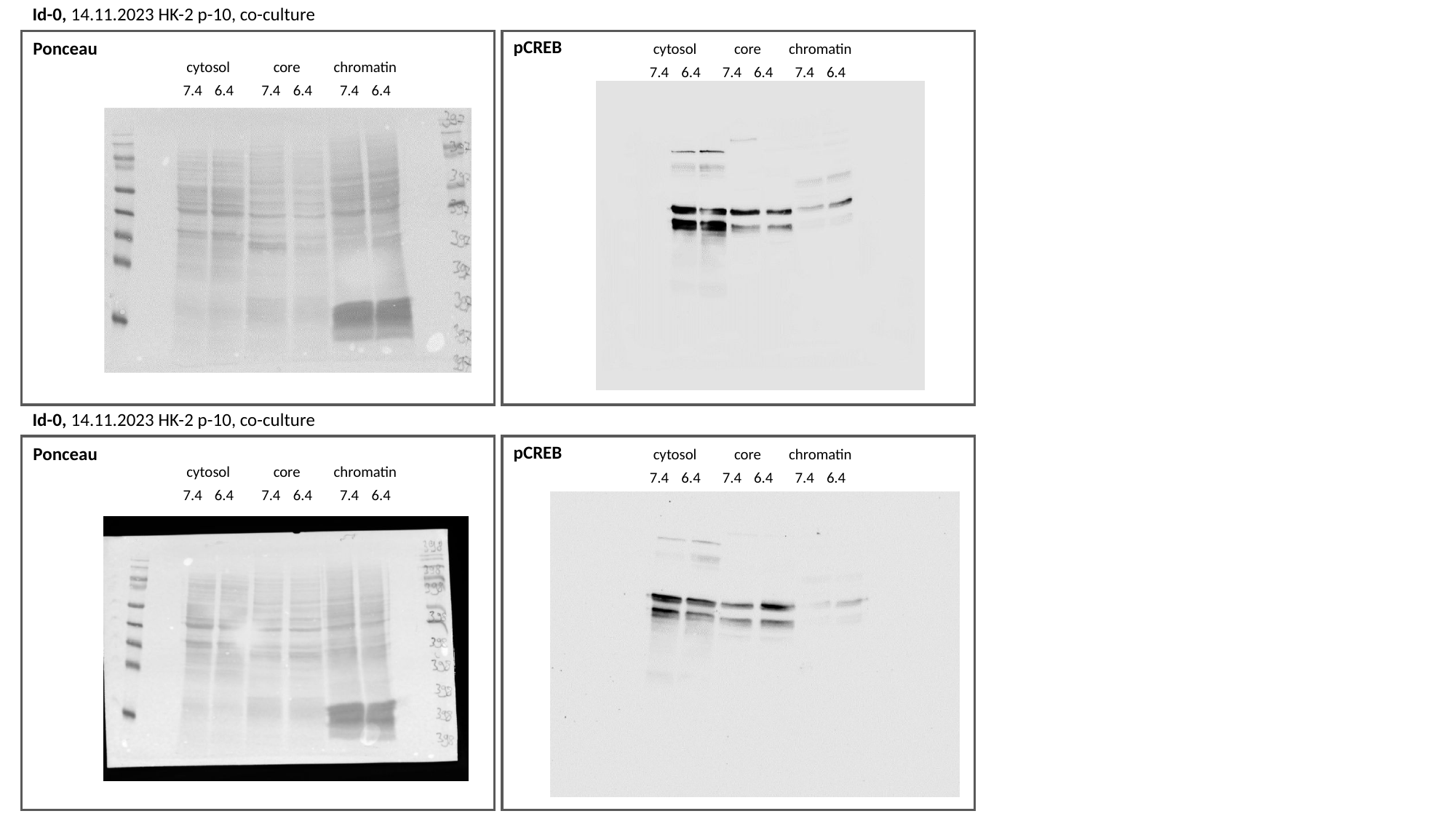

Id-0, 14.11.2023 HK-2 p-10, co-culture
pCREB
Ponceau
cytosol
7.4
6.4
core
7.4
6.4
chromatin
7.4
6.4
cytosol
7.4
6.4
core
7.4
6.4
chromatin
7.4
6.4
Id-0, 14.11.2023 HK-2 p-10, co-culture
pCREB
Ponceau
cytosol
7.4
6.4
core
7.4
6.4
chromatin
7.4
6.4
cytosol
7.4
6.4
core
7.4
6.4
chromatin
7.4
6.4

## Slide 25
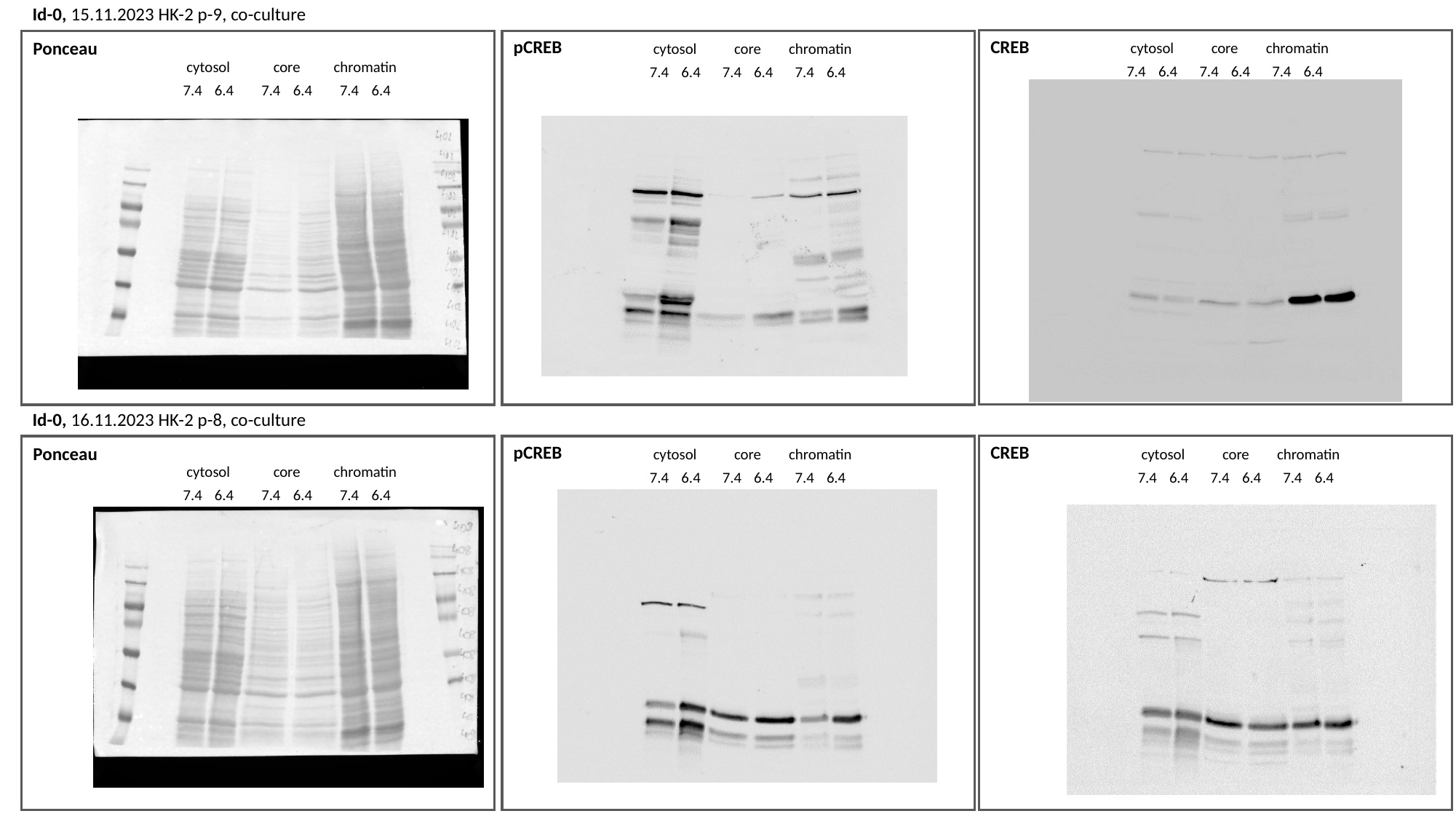

Id-0, 15.11.2023 HK-2 p-9, co-culture
CREB
pCREB
Ponceau
cytosol
7.4
6.4
core
7.4
6.4
chromatin
7.4
6.4
cytosol
7.4
6.4
core
7.4
6.4
chromatin
7.4
6.4
cytosol
7.4
6.4
core
7.4
6.4
chromatin
7.4
6.4
Id-0, 16.11.2023 HK-2 p-8, co-culture
CREB
pCREB
Ponceau
cytosol
7.4
6.4
core
7.4
6.4
chromatin
7.4
6.4
cytosol
7.4
6.4
core
7.4
6.4
chromatin
7.4
6.4
cytosol
7.4
6.4
core
7.4
6.4
chromatin
7.4
6.4

## Slide 26
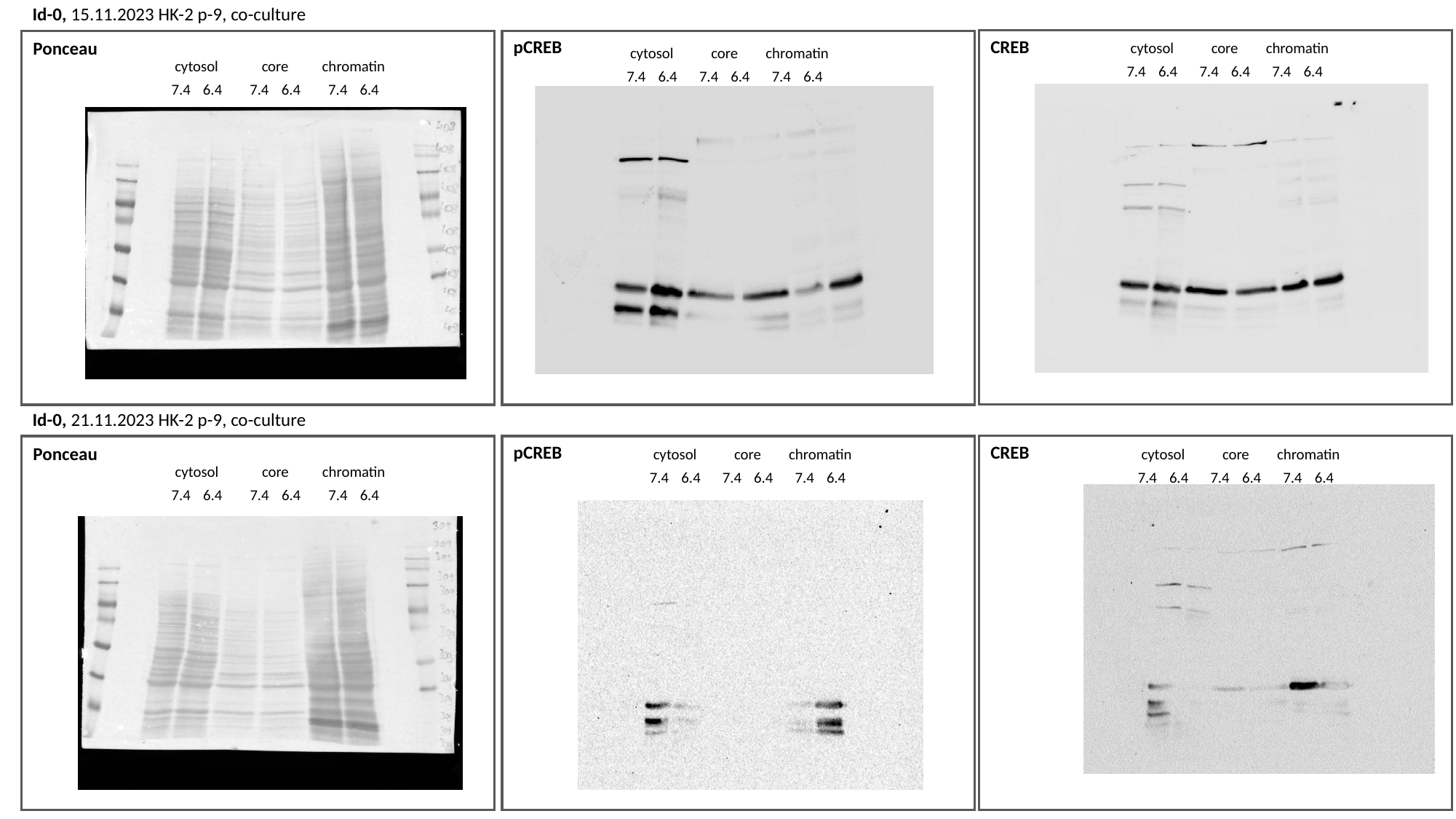

Id-0, 15.11.2023 HK-2 p-9, co-culture
CREB
pCREB
Ponceau
cytosol
7.4
6.4
core
7.4
6.4
chromatin
7.4
6.4
cytosol
7.4
6.4
core
7.4
6.4
chromatin
7.4
6.4
cytosol
7.4
6.4
core
7.4
6.4
chromatin
7.4
6.4
Id-0, 21.11.2023 HK-2 p-9, co-culture
CREB
pCREB
Ponceau
cytosol
7.4
6.4
core
7.4
6.4
chromatin
7.4
6.4
cytosol
7.4
6.4
core
7.4
6.4
chromatin
7.4
6.4
cytosol
7.4
6.4
core
7.4
6.4
chromatin
7.4
6.4

## Slide 27
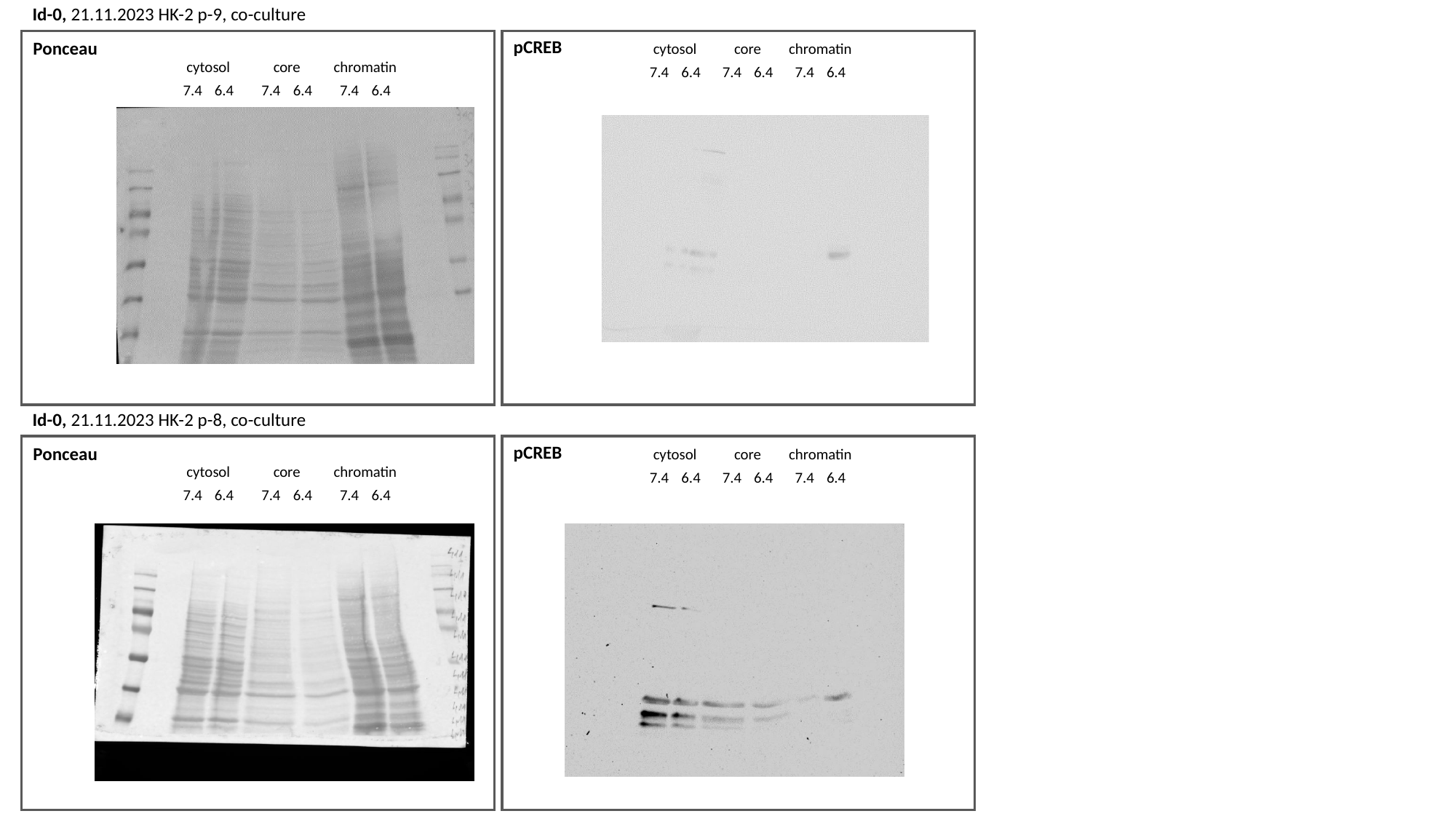

Id-0, 21.11.2023 HK-2 p-9, co-culture
pCREB
Ponceau
cytosol
7.4
6.4
core
7.4
6.4
chromatin
7.4
6.4
cytosol
7.4
6.4
core
7.4
6.4
chromatin
7.4
6.4
Id-0, 21.11.2023 HK-2 p-8, co-culture
pCREB
Ponceau
cytosol
7.4
6.4
core
7.4
6.4
chromatin
7.4
6.4
cytosol
7.4
6.4
core
7.4
6.4
chromatin
7.4
6.4

## Slide 28
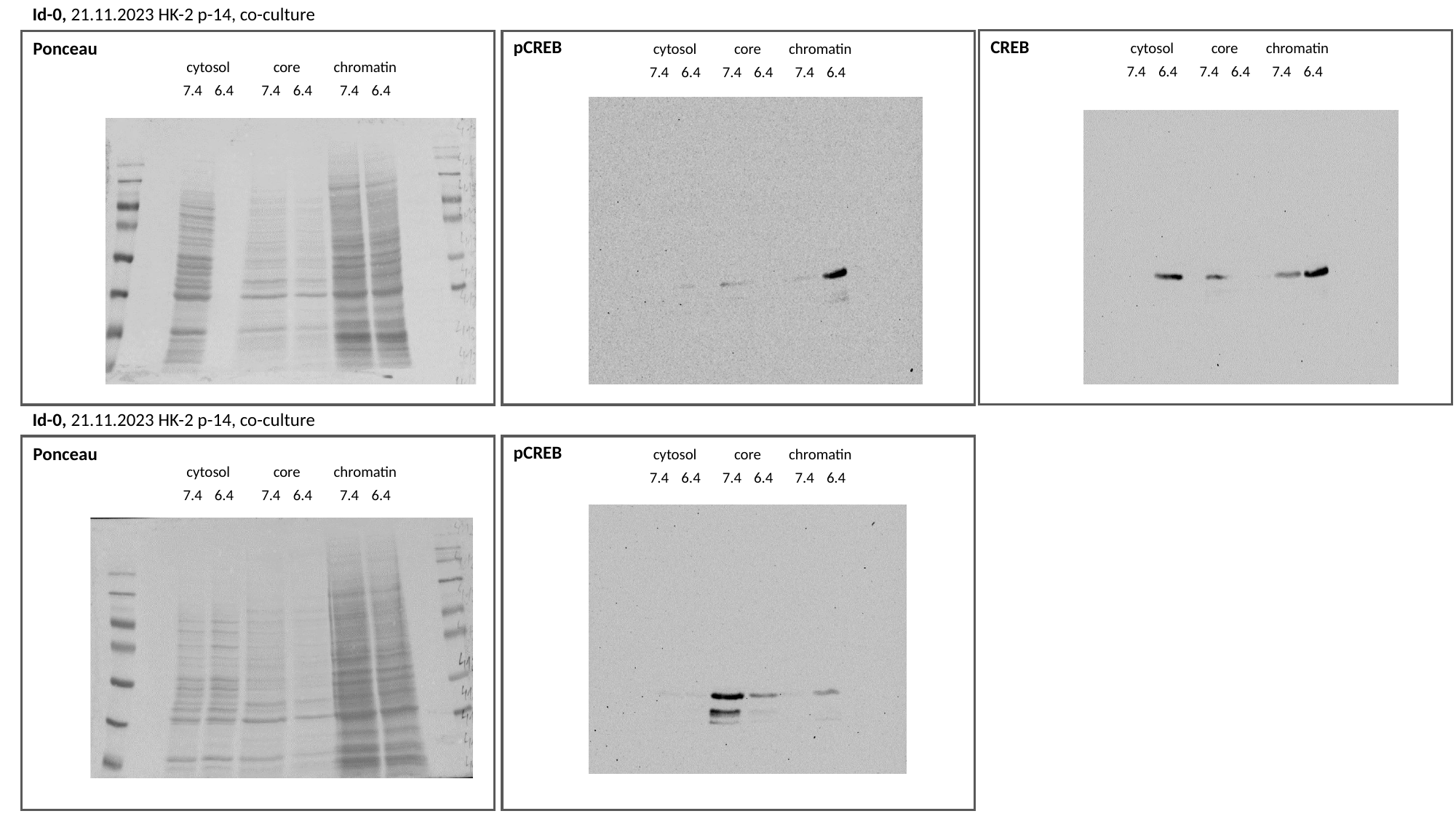

Id-0, 21.11.2023 HK-2 p-14, co-culture
CREB
pCREB
Ponceau
cytosol
7.4
6.4
core
7.4
6.4
chromatin
7.4
6.4
cytosol
7.4
6.4
core
7.4
6.4
chromatin
7.4
6.4
cytosol
7.4
6.4
core
7.4
6.4
chromatin
7.4
6.4
Id-0, 21.11.2023 HK-2 p-14, co-culture
pCREB
Ponceau
cytosol
7.4
6.4
core
7.4
6.4
chromatin
7.4
6.4
cytosol
7.4
6.4
core
7.4
6.4
chromatin
7.4
6.4

## Slide 29
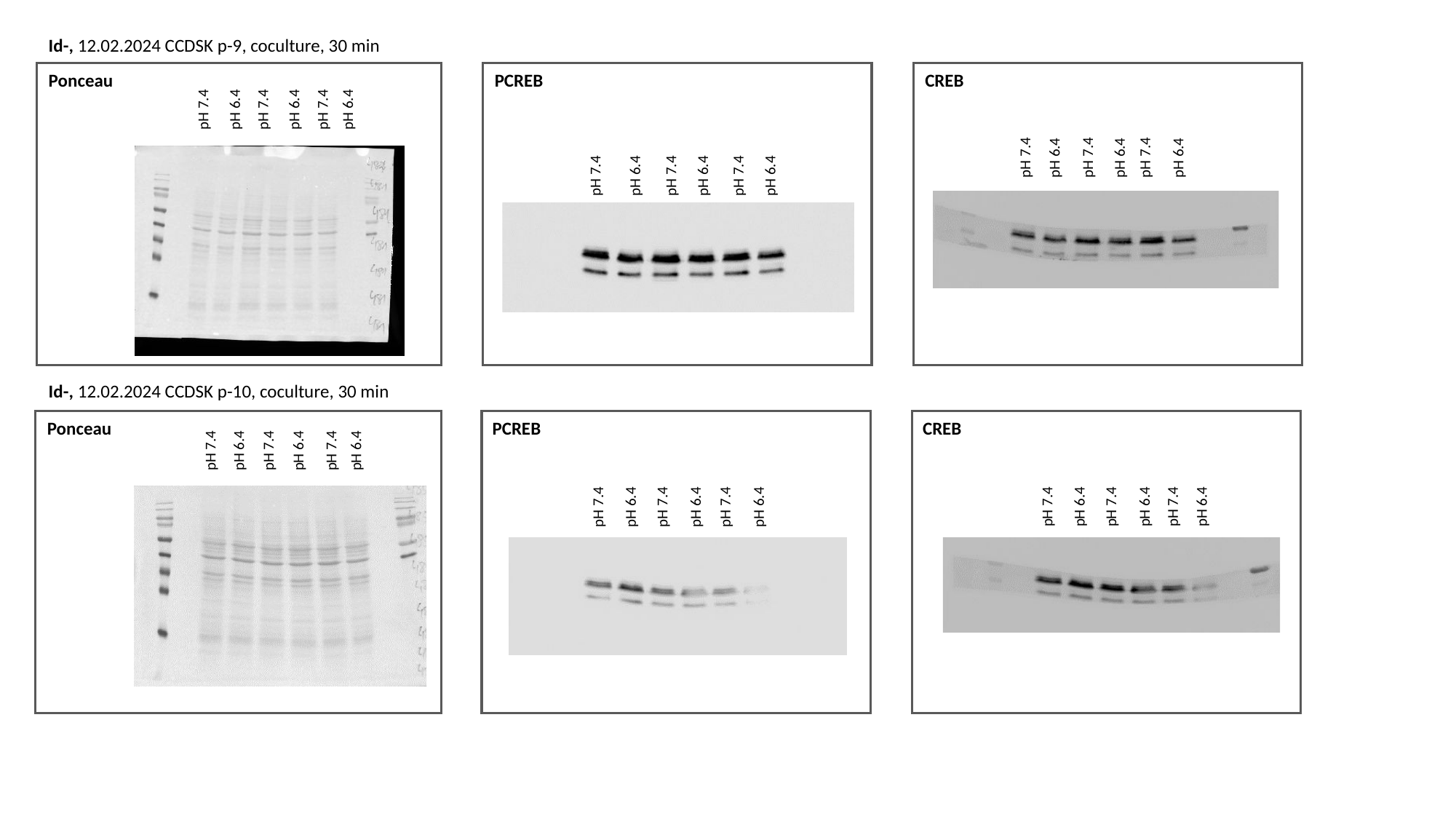

Id-, 12.02.2024 CCDSK p-9, coculture, 30 min
PCREB
CREB
Ponceau
pH 6.4
pH 6.4
pH 6.4
pH 7.4
pH 7.4
pH 7.4
pH 6.4
pH 6.4
pH 6.4
pH 7.4
pH 7.4
pH 7.4
pH 6.4
pH 6.4
pH 6.4
pH 7.4
pH 7.4
pH 7.4
Id-, 12.02.2024 CCDSK p-10, coculture, 30 min
Ponceau
PCREB
CREB
pH 6.4
pH 6.4
pH 6.4
pH 7.4
pH 7.4
pH 7.4
pH 6.4
pH 6.4
pH 6.4
pH 6.4
pH 6.4
pH 6.4
pH 7.4
pH 7.4
pH 7.4
pH 7.4
pH 7.4
pH 7.4

## Slide 30
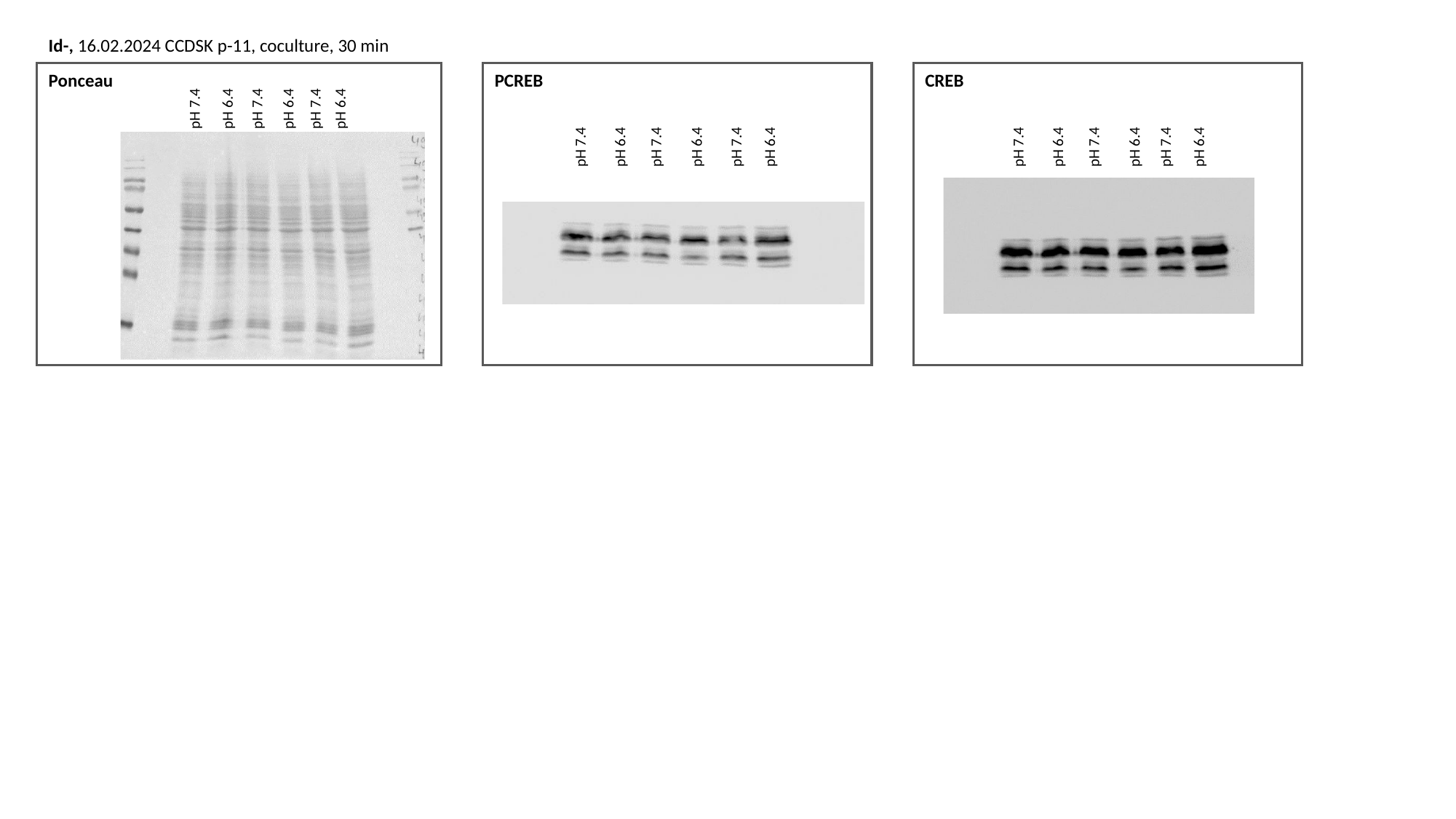

Id-, 16.02.2024 CCDSK p-11, coculture, 30 min
PCREB
CREB
Ponceau
pH 6.4
pH 6.4
pH 6.4
pH 7.4
pH 7.4
pH 7.4
pH 6.4
pH 6.4
pH 6.4
pH 6.4
pH 6.4
pH 6.4
pH 7.4
pH 7.4
pH 7.4
pH 7.4
pH 7.4
pH 7.4

## Slide 31
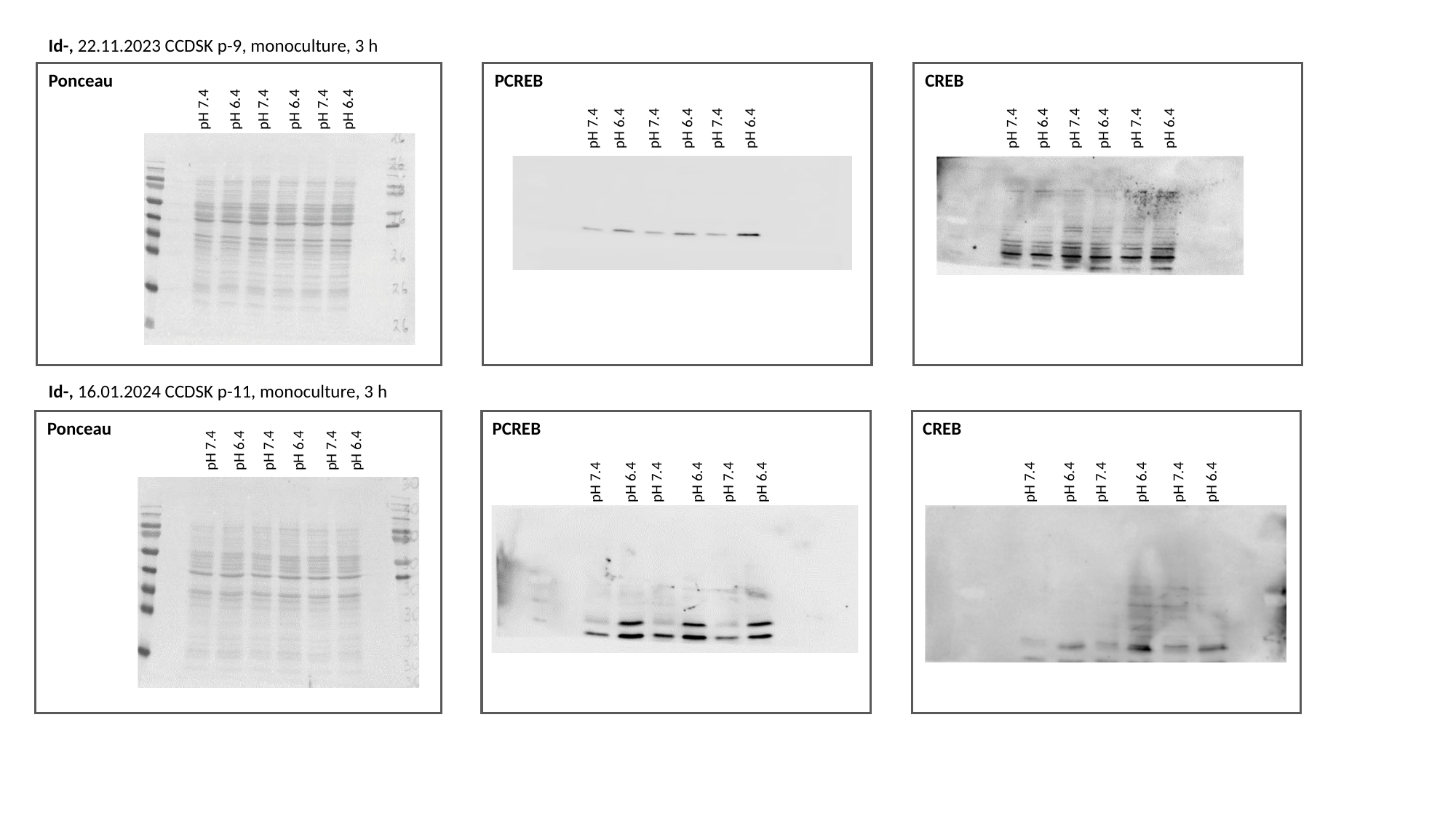

Id-, 22.11.2023 CCDSK p-9, monoculture, 3 h
PCREB
CREB
Ponceau
pH 6.4
pH 6.4
pH 6.4
pH 7.4
pH 7.4
pH 7.4
pH 6.4
pH 6.4
pH 6.4
pH 6.4
pH 6.4
pH 6.4
pH 7.4
pH 7.4
pH 7.4
pH 7.4
pH 7.4
pH 7.4
Id-, 16.01.2024 CCDSK p-11, monoculture, 3 h
Ponceau
PCREB
CREB
pH 6.4
pH 6.4
pH 6.4
pH 7.4
pH 7.4
pH 7.4
pH 6.4
pH 6.4
pH 6.4
pH 6.4
pH 6.4
pH 6.4
pH 7.4
pH 7.4
pH 7.4
pH 7.4
pH 7.4
pH 7.4

## Slide 32
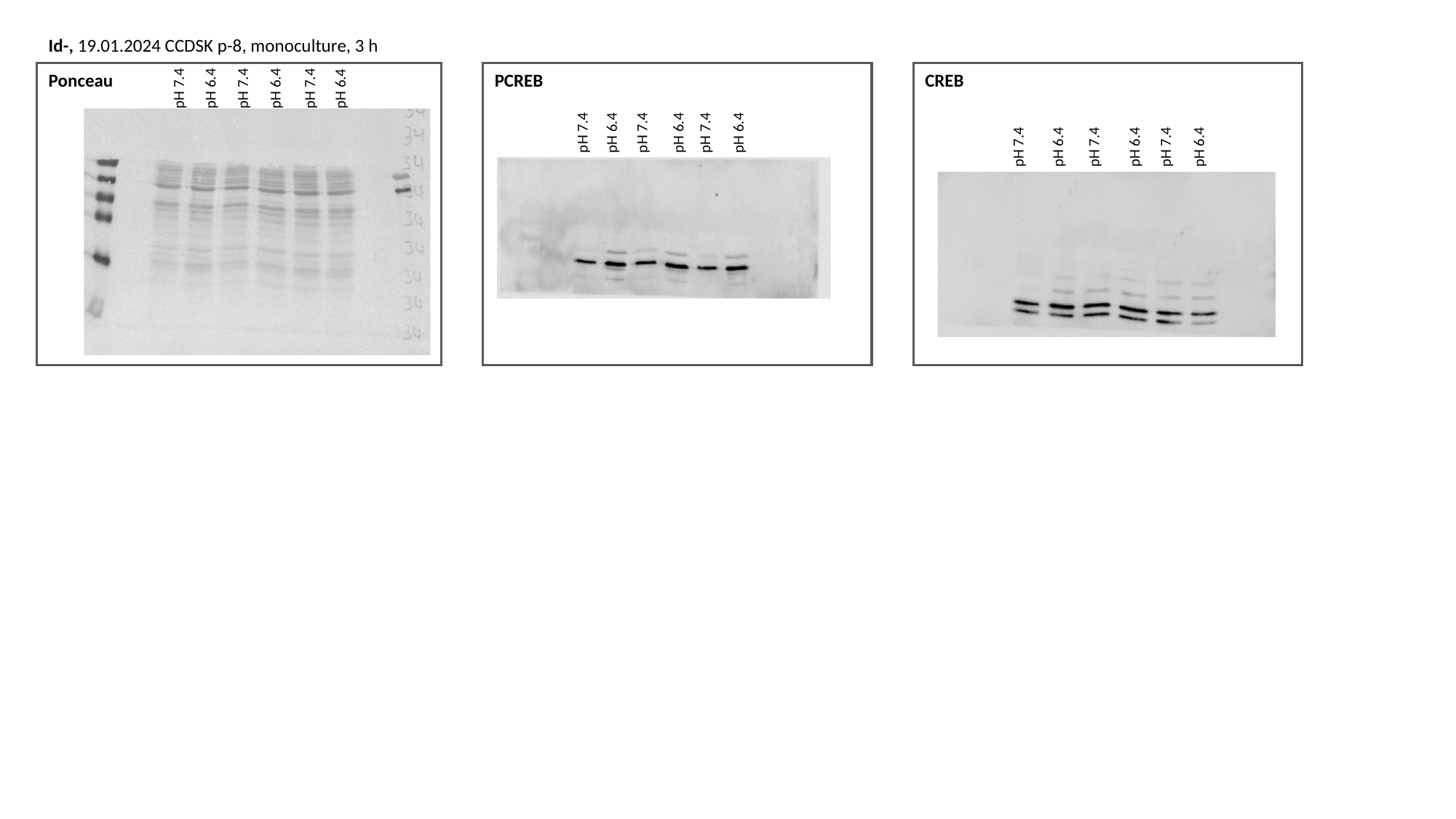

Id-, 19.01.2024 CCDSK p-8, monoculture, 3 h
pH 6.4
pH 6.4
pH 6.4
PCREB
CREB
Ponceau
pH 7.4
pH 7.4
pH 7.4
pH 6.4
pH 6.4
pH 6.4
pH 7.4
pH 7.4
pH 7.4
pH 6.4
pH 6.4
pH 6.4
pH 7.4
pH 7.4
pH 7.4

## Slide 33
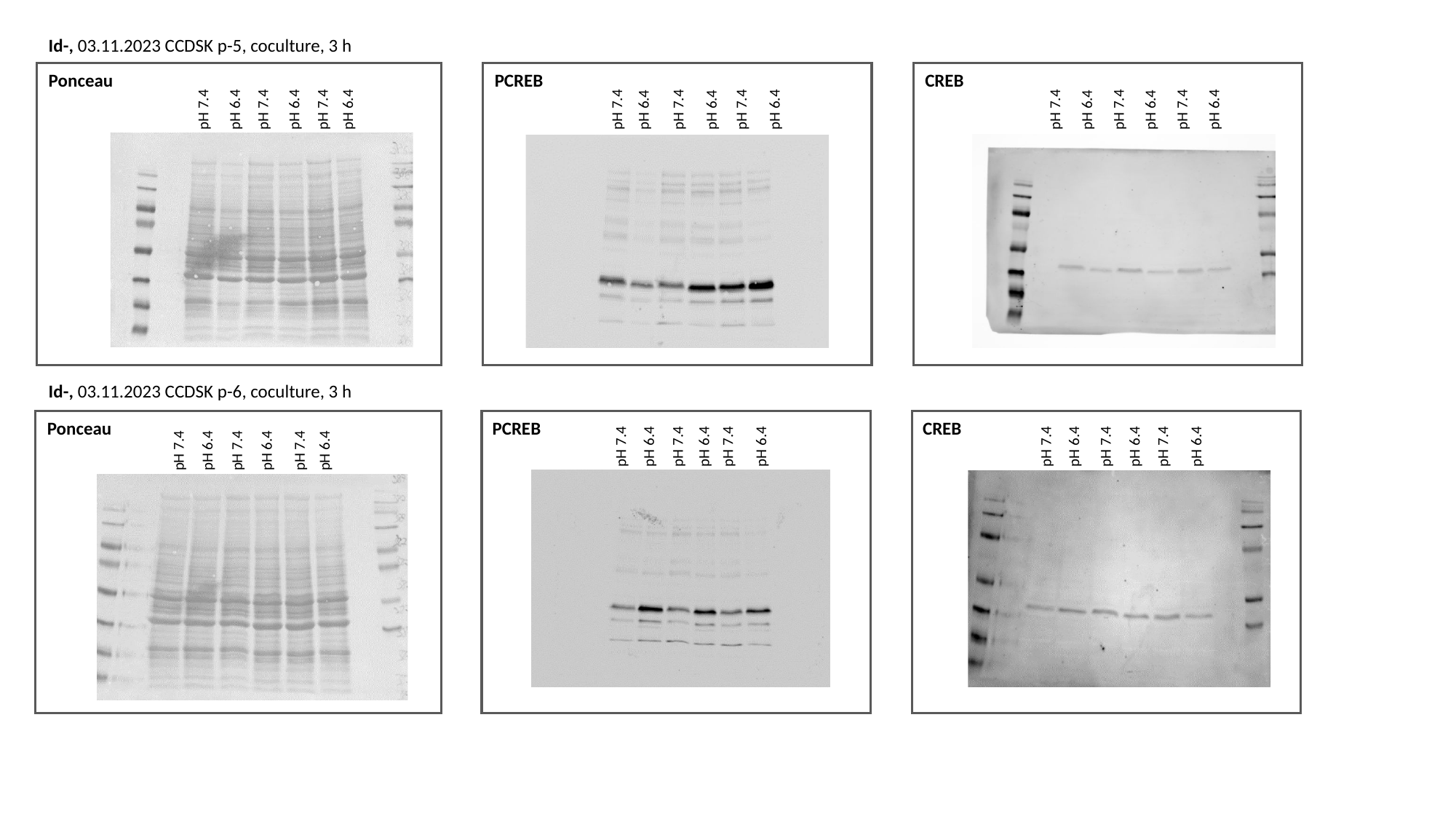

Id-, 03.11.2023 CCDSK p-5, coculture, 3 h
PCREB
CREB
Ponceau
pH 6.4
pH 6.4
pH 6.4
pH 6.4
pH 6.4
pH 6.4
pH 6.4
pH 6.4
pH 6.4
pH 7.4
pH 7.4
pH 7.4
pH 7.4
pH 7.4
pH 7.4
pH 7.4
pH 7.4
pH 7.4
Id-, 03.11.2023 CCDSK p-6, coculture, 3 h
Ponceau
PCREB
CREB
pH 6.4
pH 6.4
pH 6.4
pH 6.4
pH 6.4
pH 6.4
pH 6.4
pH 6.4
pH 6.4
pH 7.4
pH 7.4
pH 7.4
pH 7.4
pH 7.4
pH 7.4
pH 7.4
pH 7.4
pH 7.4

## Slide 34
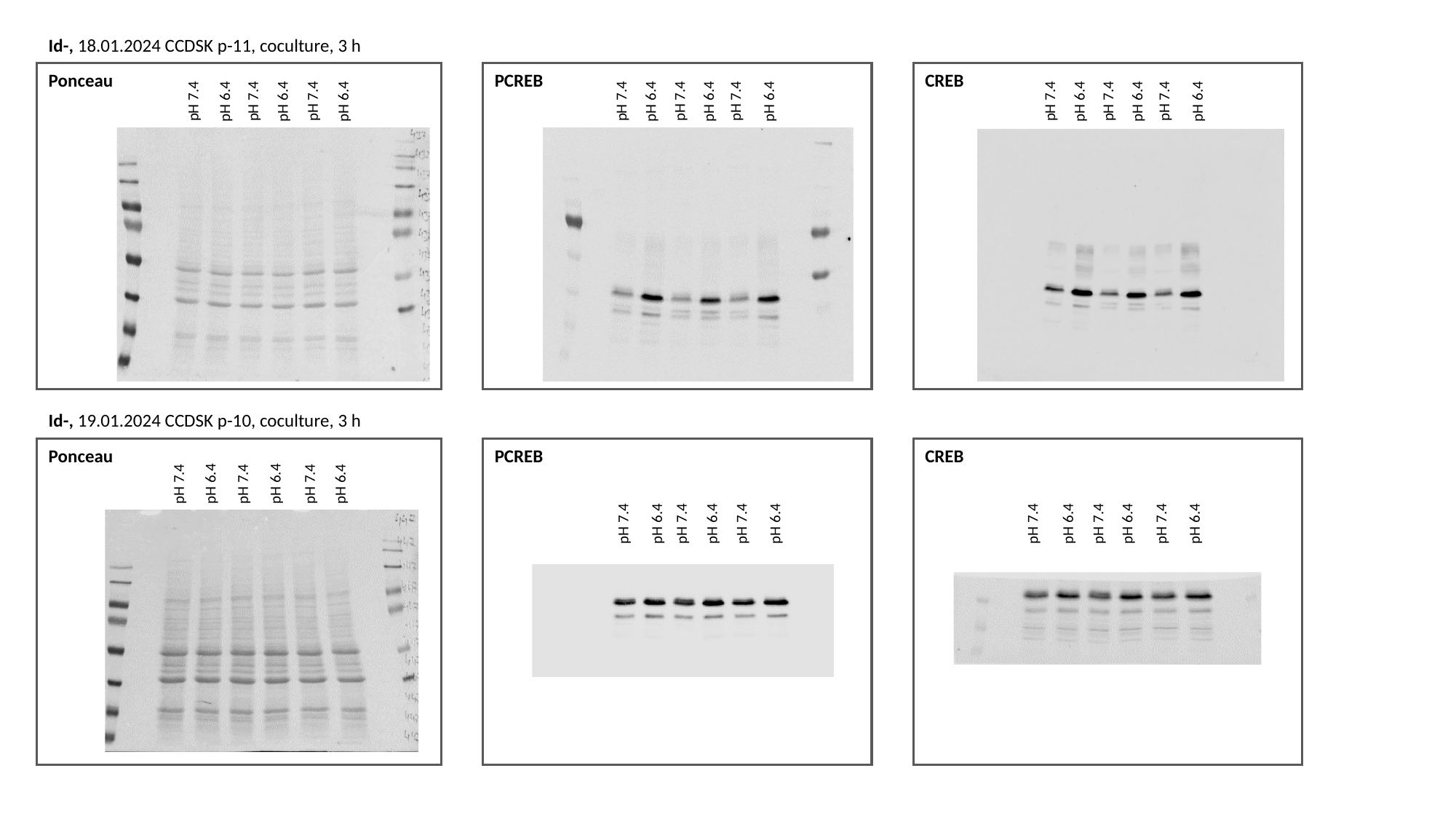

Id-, 18.01.2024 CCDSK p-11, coculture, 3 h
PCREB
CREB
Ponceau
pH 6.4
pH 6.4
pH 6.4
pH 6.4
pH 6.4
pH 6.4
pH 6.4
pH 6.4
pH 6.4
pH 7.4
pH 7.4
pH 7.4
pH 7.4
pH 7.4
pH 7.4
pH 7.4
pH 7.4
pH 7.4
Id-, 19.01.2024 CCDSK p-10, coculture, 3 h
PCREB
CREB
Ponceau
pH 6.4
pH 6.4
pH 6.4
pH 7.4
pH 7.4
pH 7.4
pH 6.4
pH 6.4
pH 6.4
pH 6.4
pH 6.4
pH 6.4
pH 7.4
pH 7.4
pH 7.4
pH 7.4
pH 7.4
pH 7.4

## Slide 35
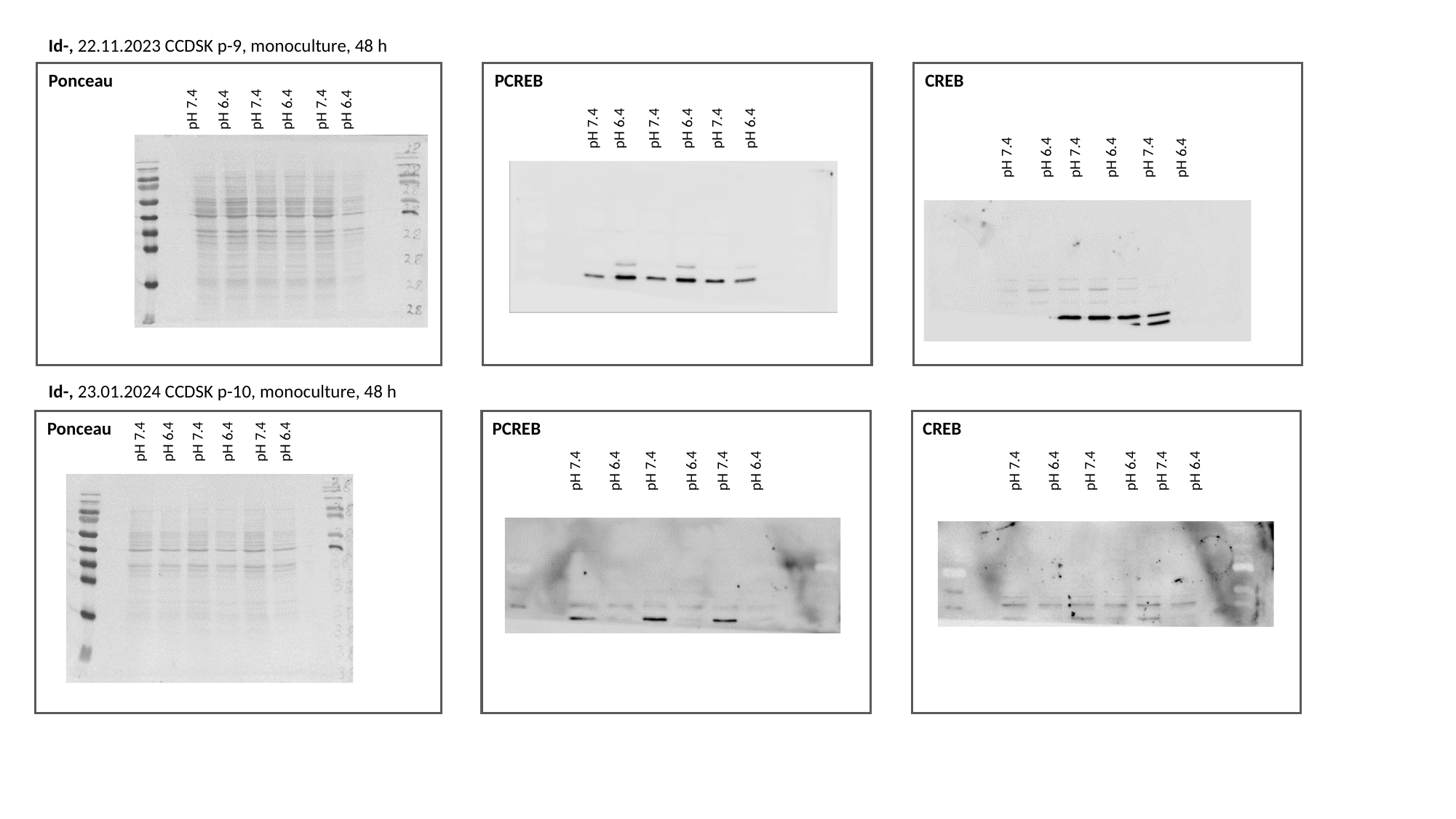

Id-, 22.11.2023 CCDSK p-9, monoculture, 48 h
PCREB
CREB
Ponceau
pH 6.4
pH 6.4
pH 6.4
pH 7.4
pH 7.4
pH 7.4
pH 6.4
pH 6.4
pH 6.4
pH 7.4
pH 7.4
pH 7.4
pH 6.4
pH 6.4
pH 6.4
pH 7.4
pH 7.4
pH 7.4
Id-, 23.01.2024 CCDSK p-10, monoculture, 48 h
Ponceau
PCREB
CREB
pH 6.4
pH 6.4
pH 6.4
pH 7.4
pH 7.4
pH 7.4
pH 6.4
pH 6.4
pH 6.4
pH 6.4
pH 6.4
pH 6.4
pH 7.4
pH 7.4
pH 7.4
pH 7.4
pH 7.4
pH 7.4

## Slide 36
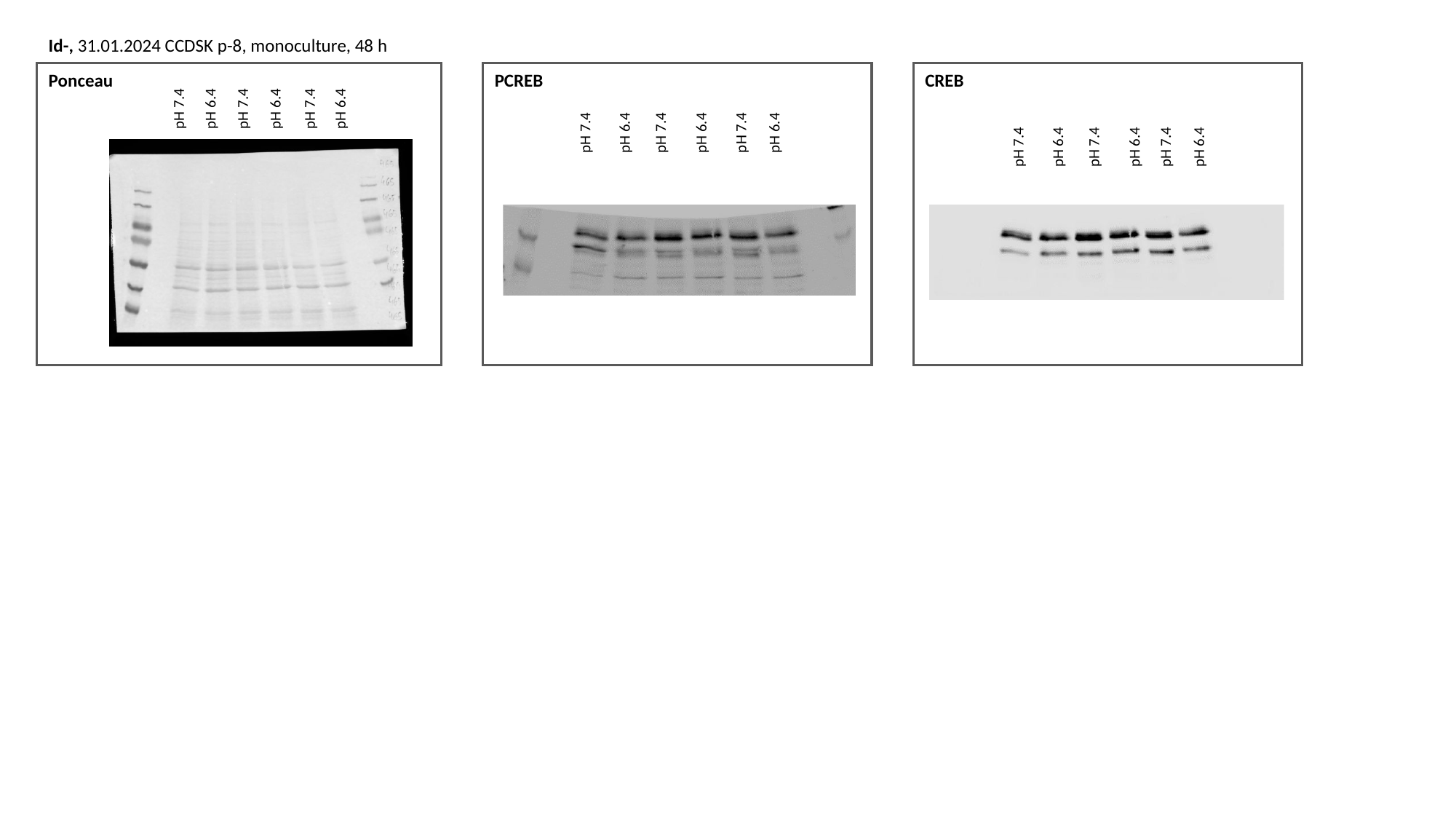

Id-, 31.01.2024 CCDSK p-8, monoculture, 48 h
PCREB
CREB
Ponceau
pH 6.4
pH 6.4
pH 6.4
pH 7.4
pH 7.4
pH 7.4
pH 6.4
pH 6.4
pH 6.4
pH 7.4
pH 7.4
pH 7.4
pH 6.4
pH 6.4
pH 6.4
pH 7.4
pH 7.4
pH 7.4

## Slide 37
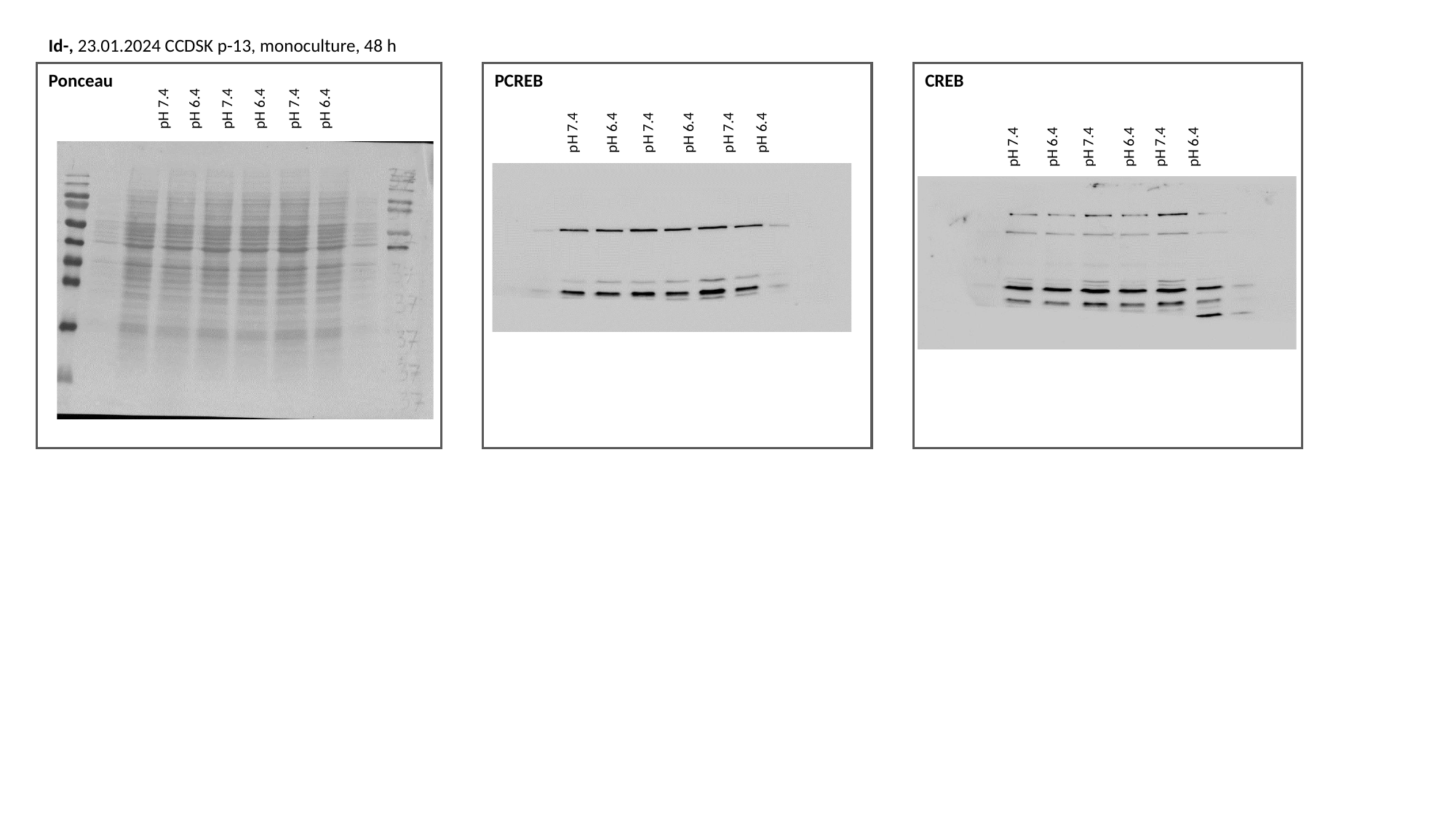

Id-, 23.01.2024 CCDSK p-13, monoculture, 48 h
PCREB
CREB
Ponceau
pH 6.4
pH 6.4
pH 6.4
pH 7.4
pH 7.4
pH 7.4
pH 6.4
pH 6.4
pH 6.4
pH 7.4
pH 7.4
pH 7.4
pH 6.4
pH 6.4
pH 6.4
pH 7.4
pH 7.4
pH 7.4

## Slide 38
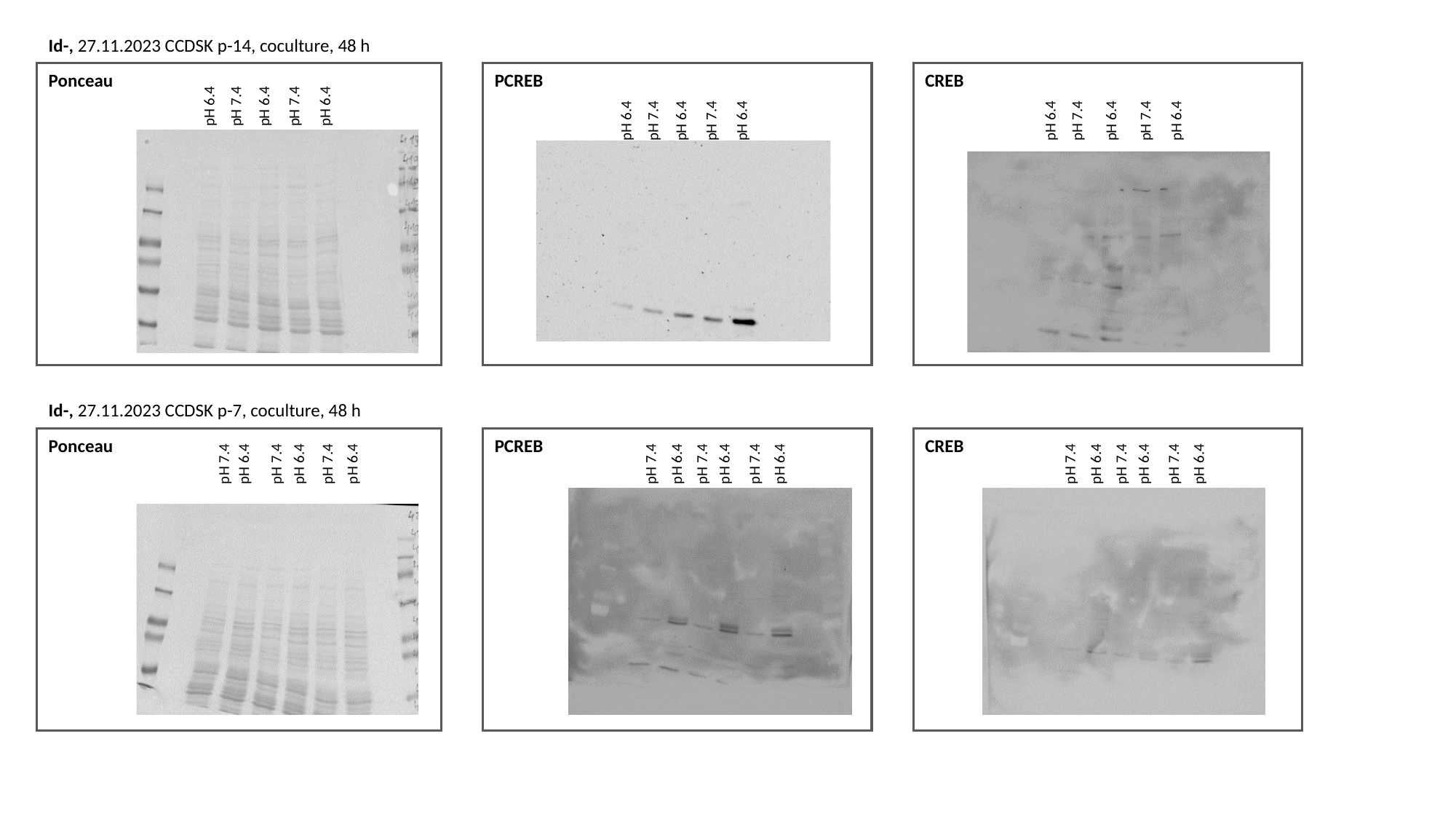

Id-, 27.11.2023 CCDSK p-14, coculture, 48 h
PCREB
CREB
Ponceau
pH 6.4
pH 6.4
pH 6.4
pH 7.4
pH 7.4
pH 6.4
pH 6.4
pH 6.4
pH 6.4
pH 6.4
pH 6.4
pH 7.4
pH 7.4
pH 7.4
pH 7.4
Id-, 27.11.2023 CCDSK p-7, coculture, 48 h
PCREB
CREB
Ponceau
pH 6.4
pH 6.4
pH 6.4
pH 6.4
pH 6.4
pH 6.4
pH 6.4
pH 6.4
pH 6.4
pH 7.4
pH 7.4
pH 7.4
pH 7.4
pH 7.4
pH 7.4
pH 7.4
pH 7.4
pH 7.4

## Slide 39
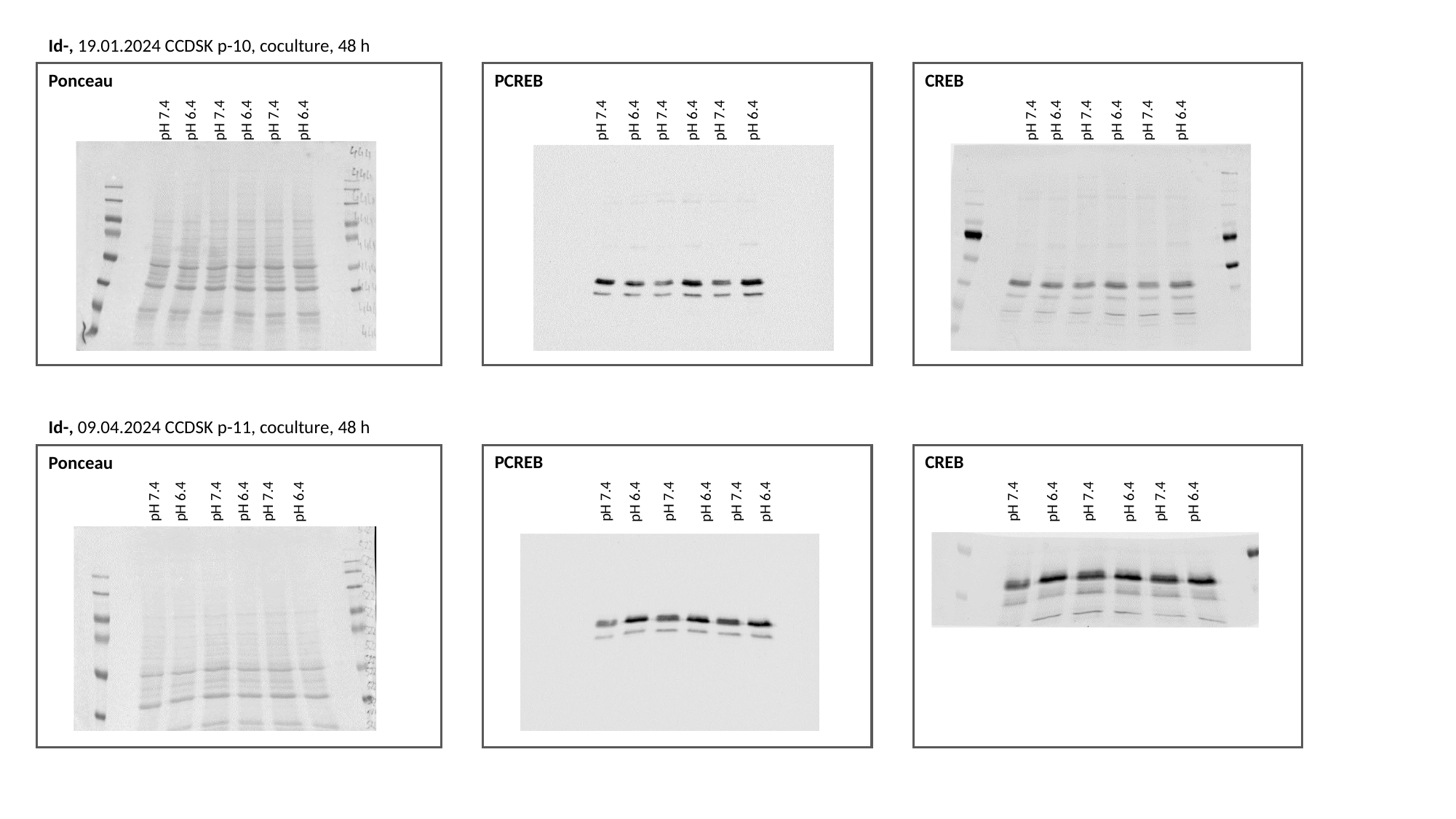

Id-, 19.01.2024 CCDSK p-10, coculture, 48 h
PCREB
CREB
Ponceau
pH 6.4
pH 6.4
pH 6.4
pH 6.4
pH 6.4
pH 6.4
pH 6.4
pH 6.4
pH 7.4
pH 7.4
pH 7.4
pH 7.4
pH 7.4
pH 7.4
pH 7.4
pH 7.4
pH 7.4
pH 6.4
Id-, 09.04.2024 CCDSK p-11, coculture, 48 h
PCREB
CREB
Ponceau
pH 6.4
pH 6.4
pH 6.4
pH 6.4
pH 6.4
pH 6.4
pH 6.4
pH 6.4
pH 7.4
pH 7.4
pH 7.4
pH 7.4
pH 7.4
pH 7.4
pH 7.4
pH 7.4
pH 7.4
pH 6.4

## Slide 40
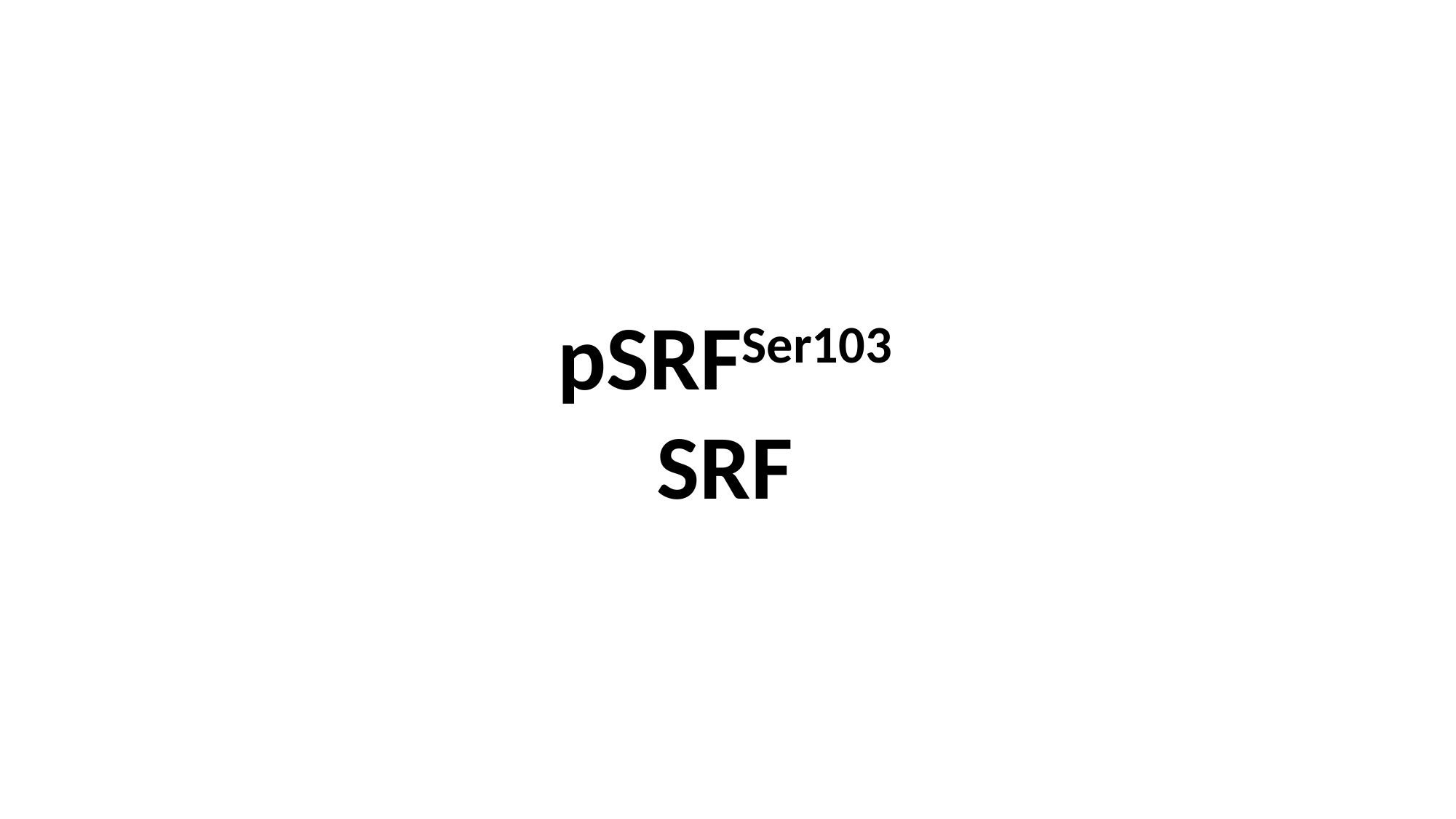

pSRFSer103
SRF

## Slide 41
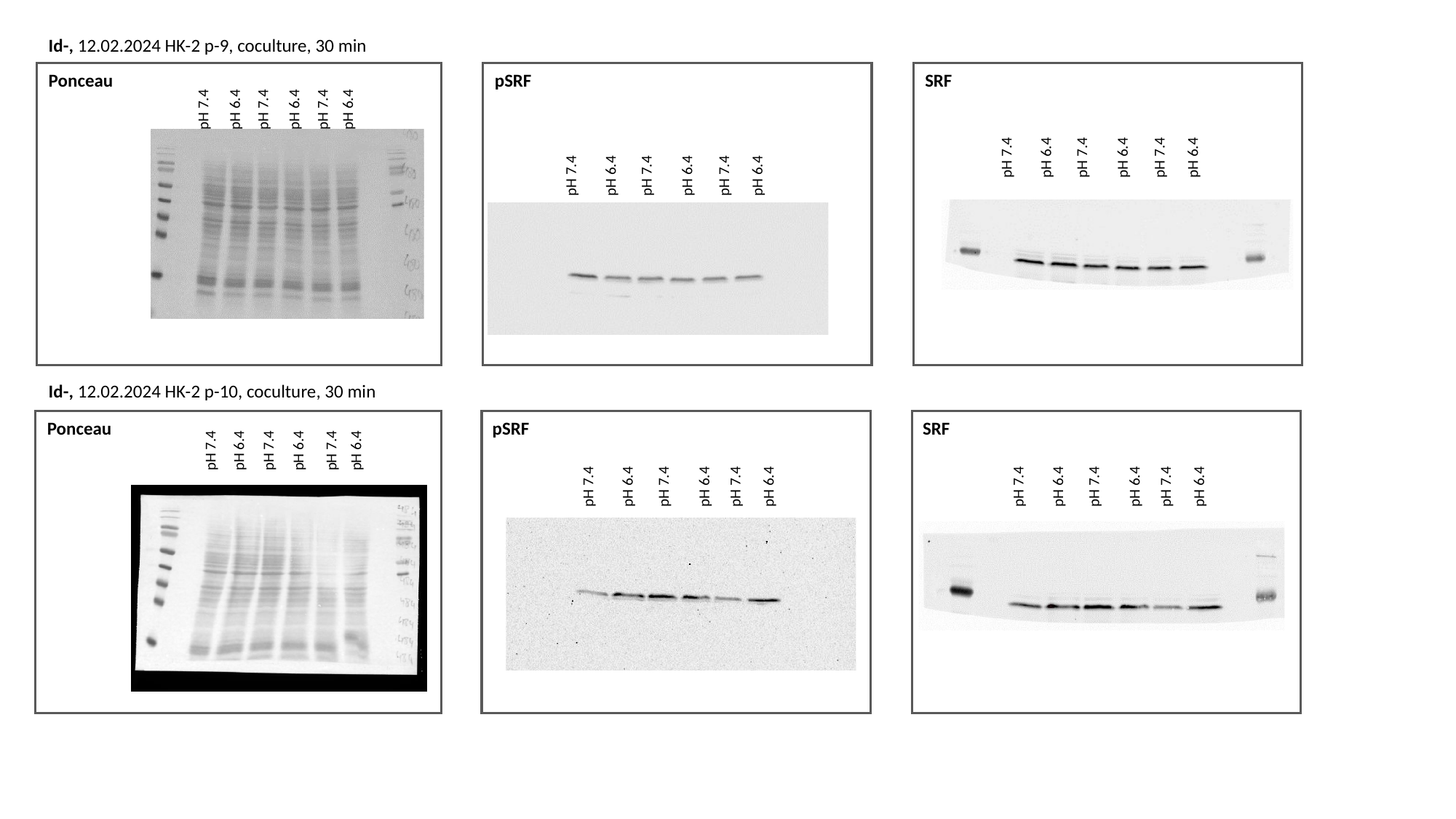

Id-, 12.02.2024 HK-2 p-9, coculture, 30 min
pSRF
SRF
Ponceau
pH 6.4
pH 6.4
pH 6.4
pH 7.4
pH 7.4
pH 7.4
pH 6.4
pH 6.4
pH 6.4
pH 7.4
pH 7.4
pH 7.4
pH 6.4
pH 6.4
pH 6.4
pH 7.4
pH 7.4
pH 7.4
Id-, 12.02.2024 HK-2 p-10, coculture, 30 min
Ponceau
pSRF
SRF
pH 6.4
pH 6.4
pH 6.4
pH 7.4
pH 7.4
pH 7.4
pH 6.4
pH 6.4
pH 6.4
pH 6.4
pH 6.4
pH 6.4
pH 7.4
pH 7.4
pH 7.4
pH 7.4
pH 7.4
pH 7.4

## Slide 42
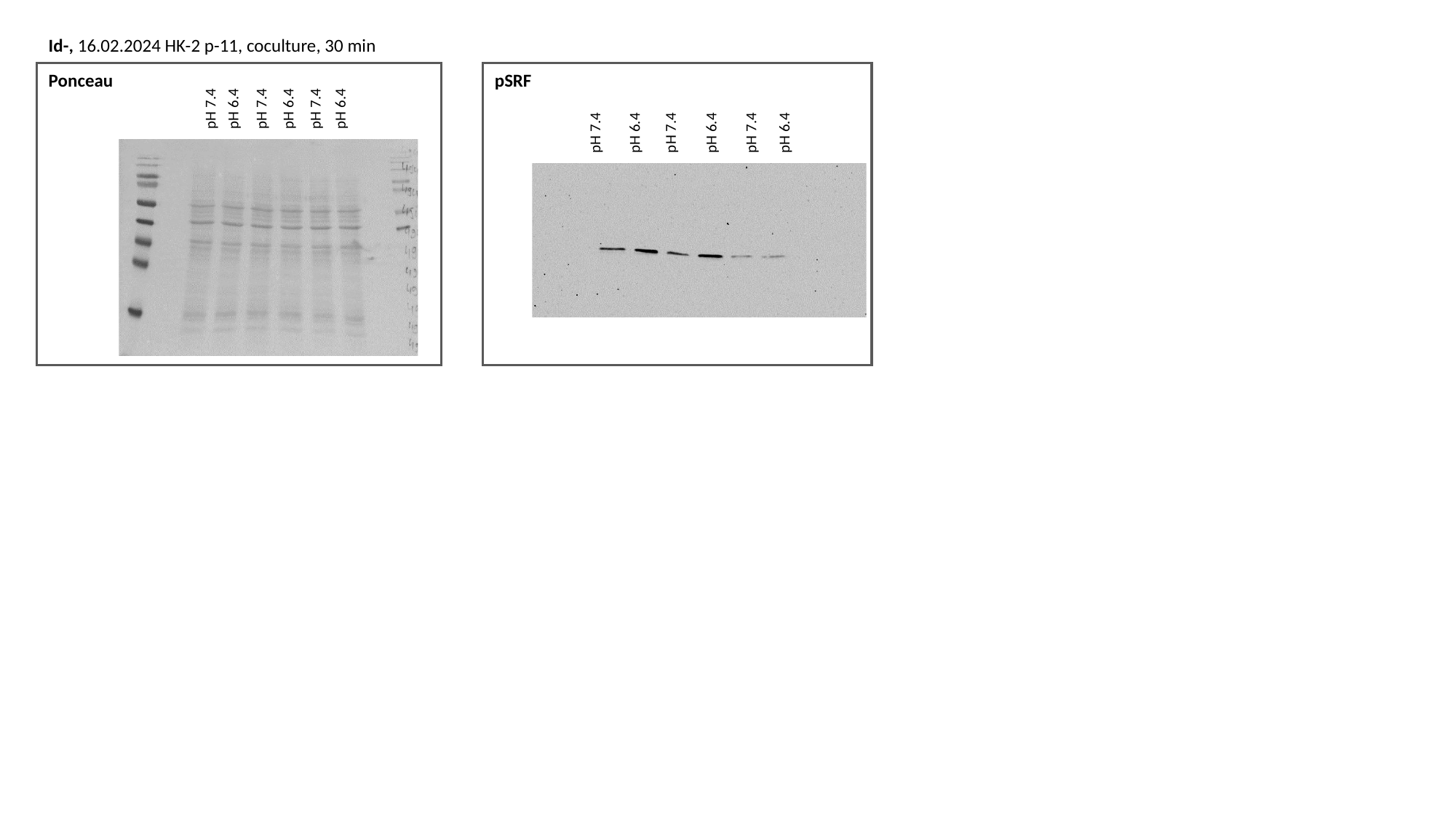

Id-, 16.02.2024 HK-2 p-11, coculture, 30 min
pSRF
Ponceau
pH 6.4
pH 6.4
pH 6.4
pH 7.4
pH 7.4
pH 7.4
pH 6.4
pH 6.4
pH 6.4
pH 7.4
pH 7.4
pH 7.4

## Slide 43
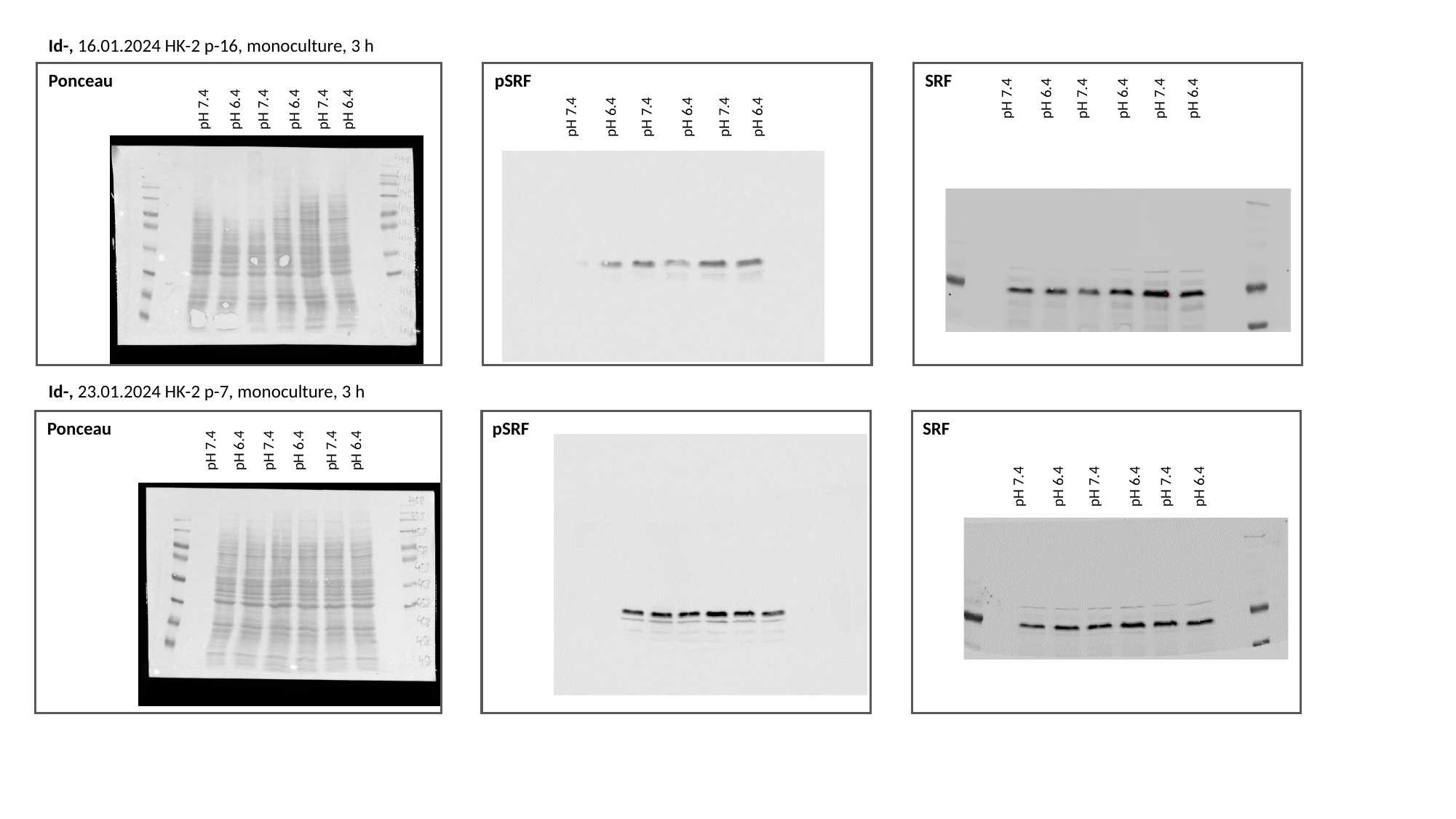

Id-, 16.01.2024 HK-2 p-16, monoculture, 3 h
pSRF
SRF
Ponceau
pH 6.4
pH 6.4
pH 6.4
pH 7.4
pH 7.4
pH 7.4
pH 6.4
pH 6.4
pH 6.4
pH 6.4
pH 6.4
pH 6.4
pH 7.4
pH 7.4
pH 7.4
pH 7.4
pH 7.4
pH 7.4
Id-, 23.01.2024 HK-2 p-7, monoculture, 3 h
Ponceau
pSRF
SRF
pH 6.4
pH 6.4
pH 6.4
pH 7.4
pH 7.4
pH 7.4
pH 6.4
pH 6.4
pH 6.4
pH 6.4
pH 6.4
pH 6.4
pH 7.4
pH 7.4
pH 7.4
pH 7.4
pH 7.4
pH 7.4

## Slide 44
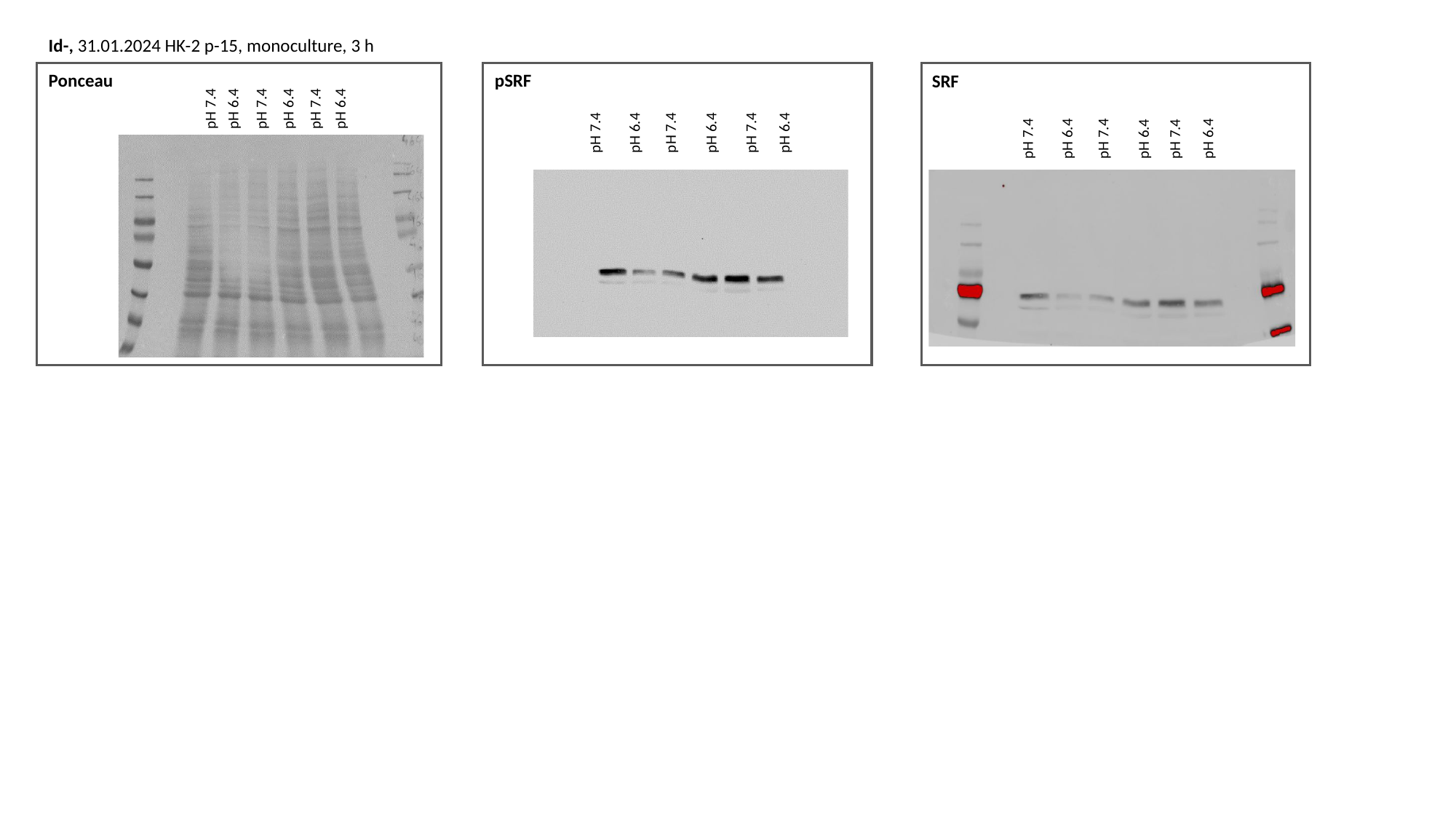

Id-, 31.01.2024 HK-2 p-15, monoculture, 3 h
pSRF
Ponceau
SRF
pH 6.4
pH 6.4
pH 6.4
pH 7.4
pH 7.4
pH 7.4
pH 6.4
pH 6.4
pH 6.4
pH 6.4
pH 6.4
pH 6.4
pH 7.4
pH 7.4
pH 7.4
pH 7.4
pH 7.4
pH 7.4

## Slide 45
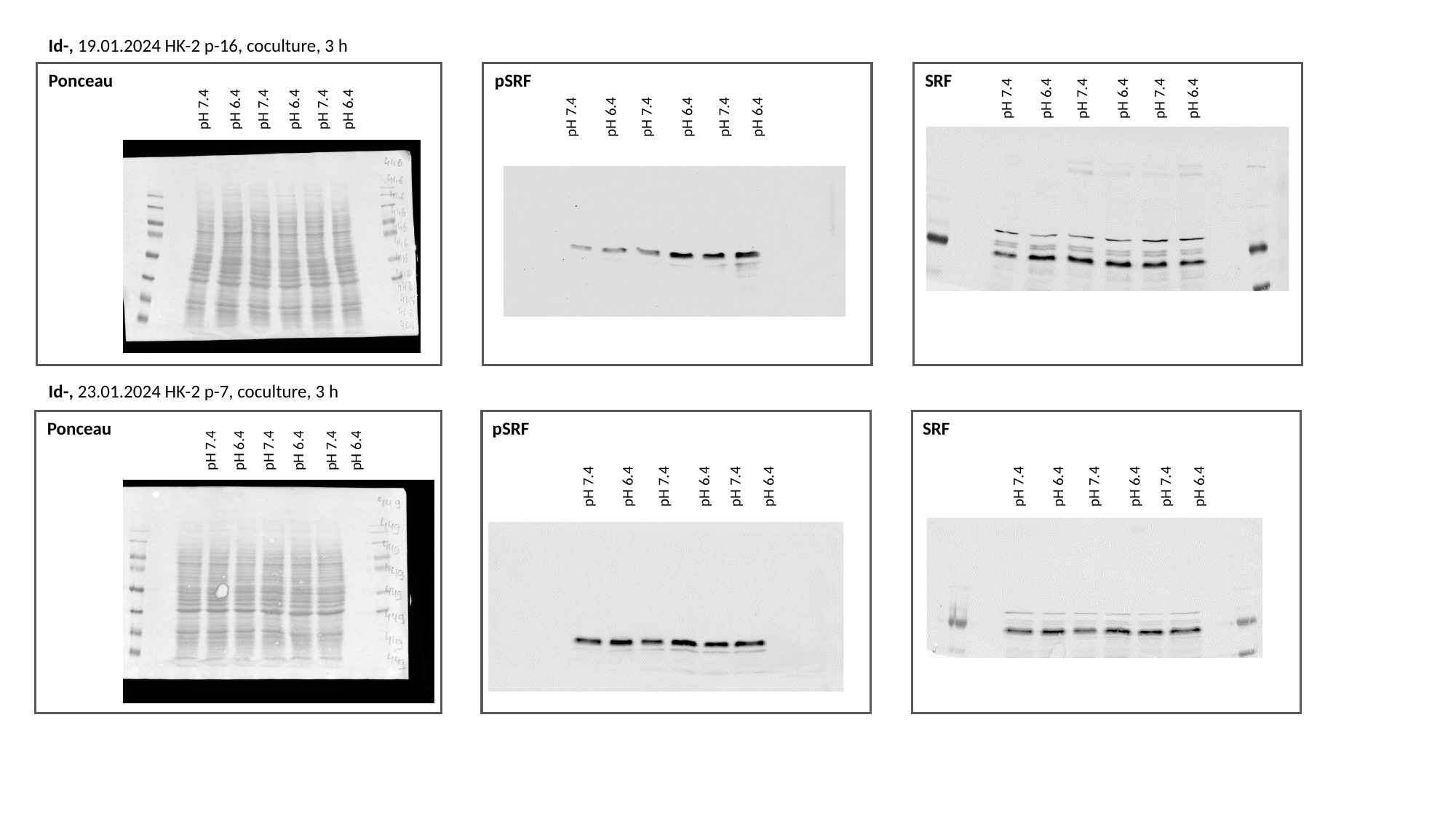

Id-, 19.01.2024 HK-2 p-16, coculture, 3 h
pSRF
SRF
Ponceau
pH 6.4
pH 6.4
pH 6.4
pH 7.4
pH 7.4
pH 7.4
pH 6.4
pH 6.4
pH 6.4
pH 6.4
pH 6.4
pH 6.4
pH 7.4
pH 7.4
pH 7.4
pH 7.4
pH 7.4
pH 7.4
Id-, 23.01.2024 HK-2 p-7, coculture, 3 h
Ponceau
pSRF
SRF
pH 6.4
pH 6.4
pH 6.4
pH 7.4
pH 7.4
pH 7.4
pH 6.4
pH 6.4
pH 6.4
pH 6.4
pH 6.4
pH 6.4
pH 7.4
pH 7.4
pH 7.4
pH 7.4
pH 7.4
pH 7.4

## Slide 46
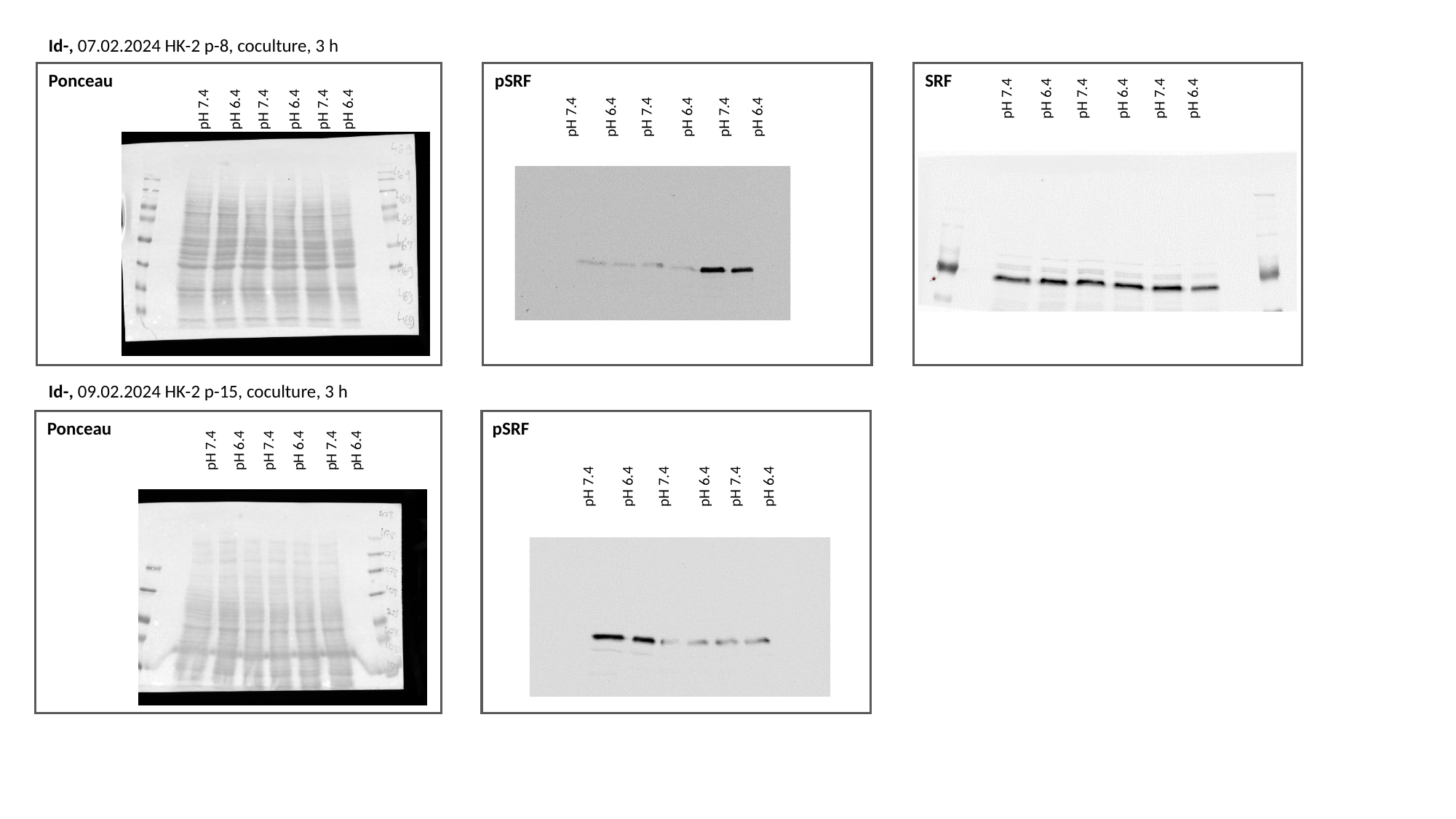

Id-, 07.02.2024 HK-2 p-8, coculture, 3 h
pSRF
SRF
Ponceau
pH 6.4
pH 6.4
pH 6.4
pH 7.4
pH 7.4
pH 7.4
pH 6.4
pH 6.4
pH 6.4
pH 6.4
pH 6.4
pH 6.4
pH 7.4
pH 7.4
pH 7.4
pH 7.4
pH 7.4
pH 7.4
Id-, 09.02.2024 HK-2 p-15, coculture, 3 h
Ponceau
pSRF
pH 6.4
pH 6.4
pH 6.4
pH 7.4
pH 7.4
pH 7.4
pH 6.4
pH 6.4
pH 6.4
pH 7.4
pH 7.4
pH 7.4

## Slide 47
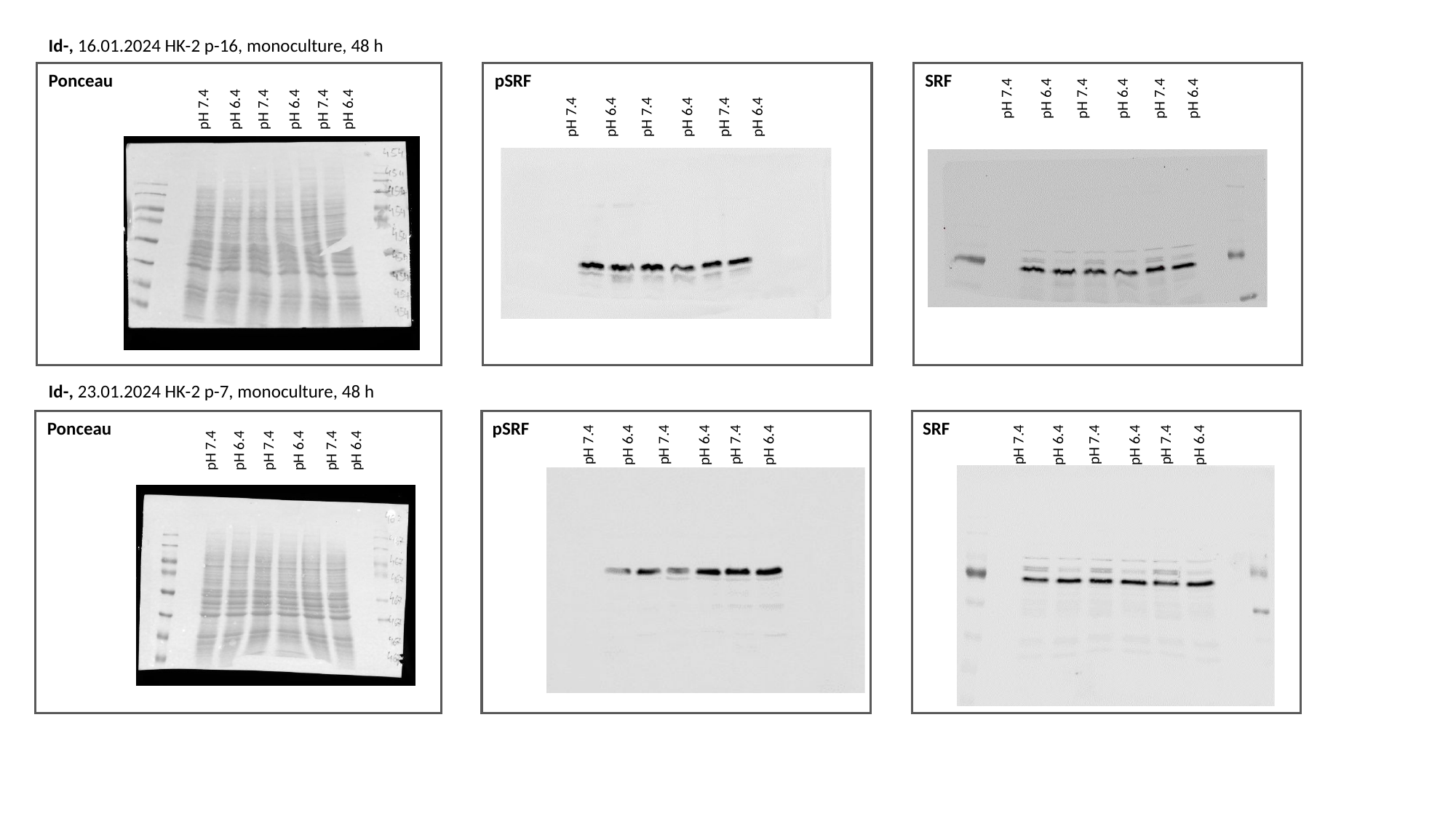

Id-, 16.01.2024 HK-2 p-16, monoculture, 48 h
pSRF
SRF
Ponceau
pH 6.4
pH 6.4
pH 6.4
pH 7.4
pH 7.4
pH 7.4
pH 6.4
pH 6.4
pH 6.4
pH 6.4
pH 6.4
pH 6.4
pH 7.4
pH 7.4
pH 7.4
pH 7.4
pH 7.4
pH 7.4
Id-, 23.01.2024 HK-2 p-7, monoculture, 48 h
Ponceau
pSRF
SRF
pH 6.4
pH 6.4
pH 6.4
pH 6.4
pH 6.4
pH 6.4
pH 6.4
pH 6.4
pH 6.4
pH 7.4
pH 7.4
pH 7.4
pH 7.4
pH 7.4
pH 7.4
pH 7.4
pH 7.4
pH 7.4

## Slide 48
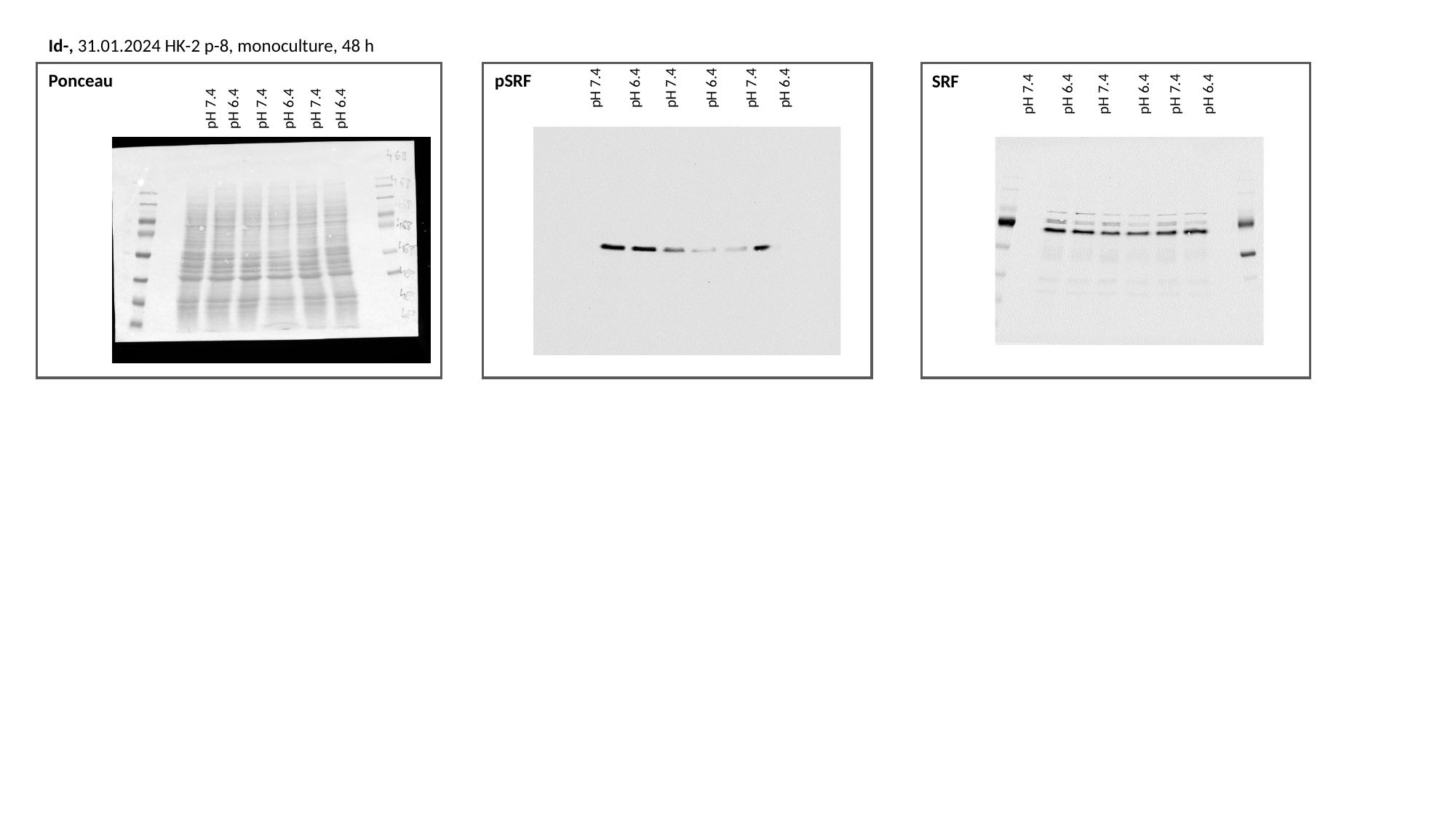

Id-, 31.01.2024 HK-2 p-8, monoculture, 48 h
pH 6.4
pH 6.4
pH 6.4
pSRF
Ponceau
SRF
pH 6.4
pH 6.4
pH 6.4
pH 7.4
pH 7.4
pH 7.4
pH 7.4
pH 7.4
pH 7.4
pH 6.4
pH 6.4
pH 6.4
pH 7.4
pH 7.4
pH 7.4

## Slide 49
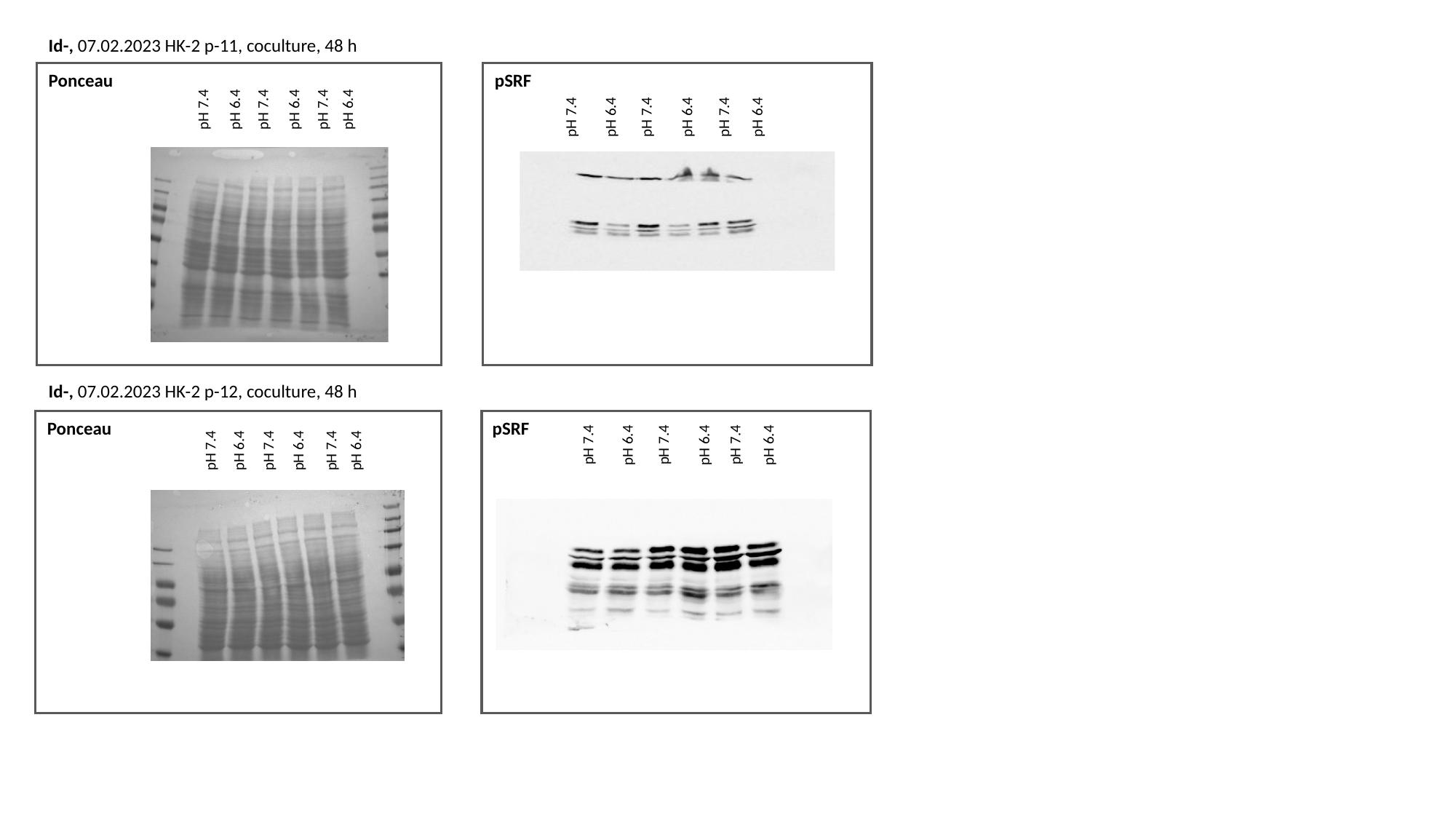

Id-, 07.02.2023 HK-2 p-11, coculture, 48 h
pSRF
Ponceau
pH 6.4
pH 6.4
pH 6.4
pH 6.4
pH 6.4
pH 6.4
pH 7.4
pH 7.4
pH 7.4
pH 7.4
pH 7.4
pH 7.4
Id-, 07.02.2023 HK-2 p-12, coculture, 48 h
Ponceau
pSRF
pH 6.4
pH 6.4
pH 6.4
pH 6.4
pH 6.4
pH 6.4
pH 7.4
pH 7.4
pH 7.4
pH 7.4
pH 7.4
pH 7.4

## Slide 50
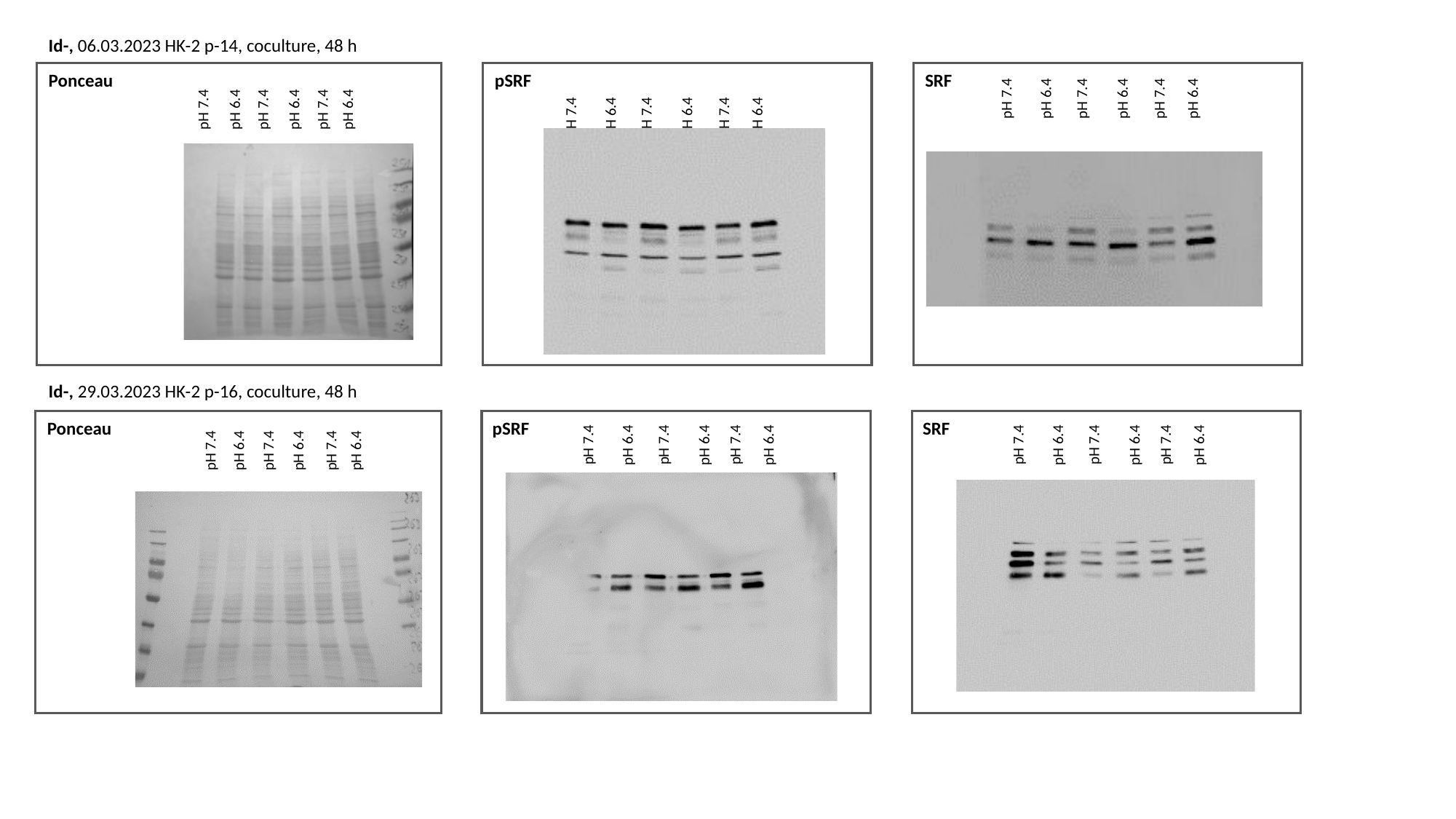

Id-, 06.03.2023 HK-2 p-14, coculture, 48 h
pSRF
SRF
Ponceau
pH 6.4
pH 6.4
pH 6.4
pH 7.4
pH 7.4
pH 7.4
pH 6.4
pH 6.4
pH 6.4
pH 6.4
pH 6.4
pH 6.4
pH 7.4
pH 7.4
pH 7.4
pH 7.4
pH 7.4
pH 7.4
Id-, 29.03.2023 HK-2 p-16, coculture, 48 h
Ponceau
pSRF
SRF
pH 6.4
pH 6.4
pH 6.4
pH 6.4
pH 6.4
pH 6.4
pH 6.4
pH 6.4
pH 6.4
pH 7.4
pH 7.4
pH 7.4
pH 7.4
pH 7.4
pH 7.4
pH 7.4
pH 7.4
pH 7.4

## Slide 51
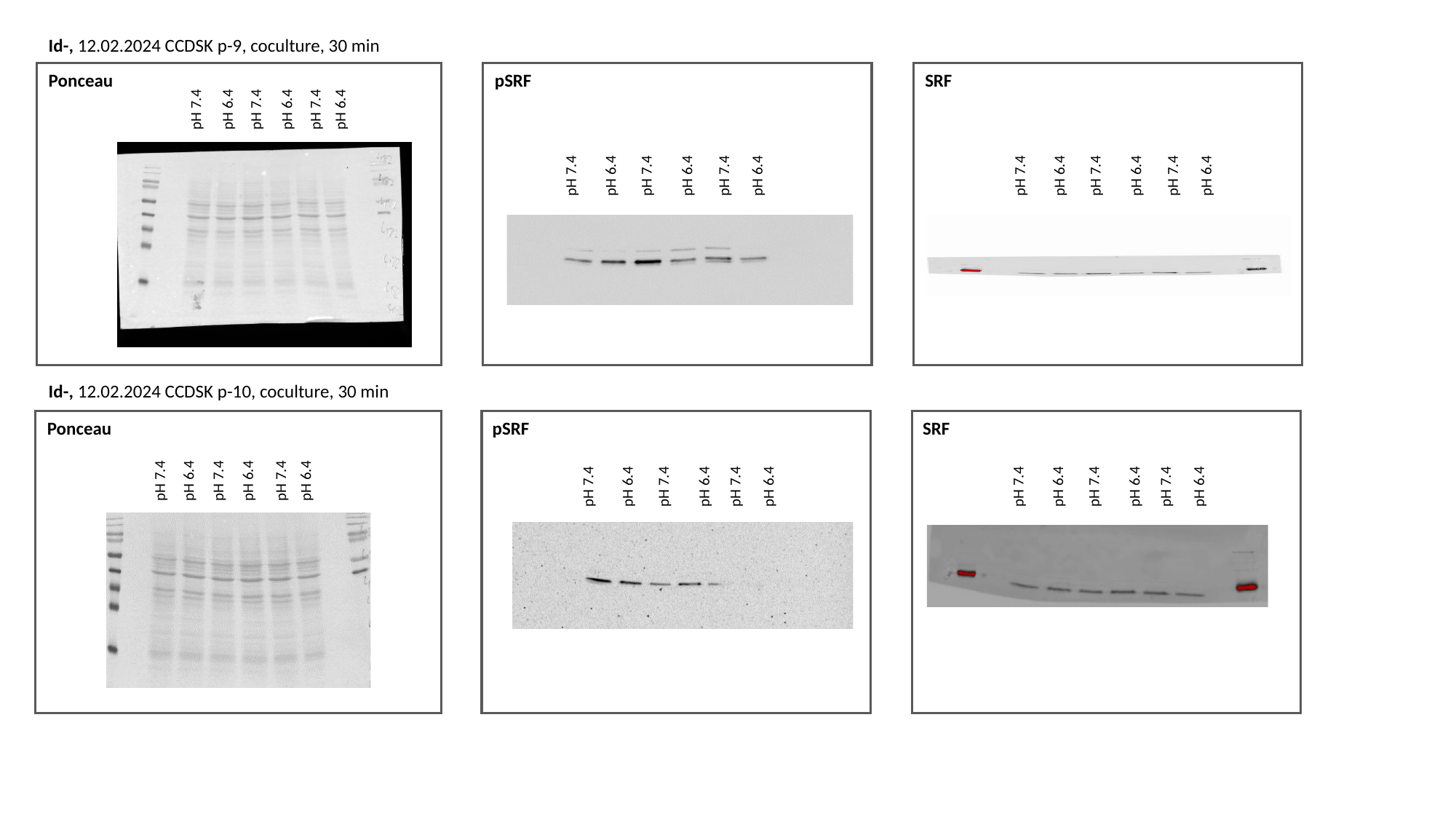

Id-, 12.02.2024 CCDSK p-9, coculture, 30 min
pSRF
SRF
Ponceau
pH 6.4
pH 6.4
pH 6.4
pH 7.4
pH 7.4
pH 7.4
pH 6.4
pH 6.4
pH 6.4
pH 6.4
pH 6.4
pH 6.4
pH 7.4
pH 7.4
pH 7.4
pH 7.4
pH 7.4
pH 7.4
Id-, 12.02.2024 CCDSK p-10, coculture, 30 min
Ponceau
pSRF
SRF
pH 6.4
pH 6.4
pH 6.4
pH 6.4
pH 6.4
pH 6.4
pH 6.4
pH 6.4
pH 6.4
pH 7.4
pH 7.4
pH 7.4
pH 7.4
pH 7.4
pH 7.4
pH 7.4
pH 7.4
pH 7.4

## Slide 52
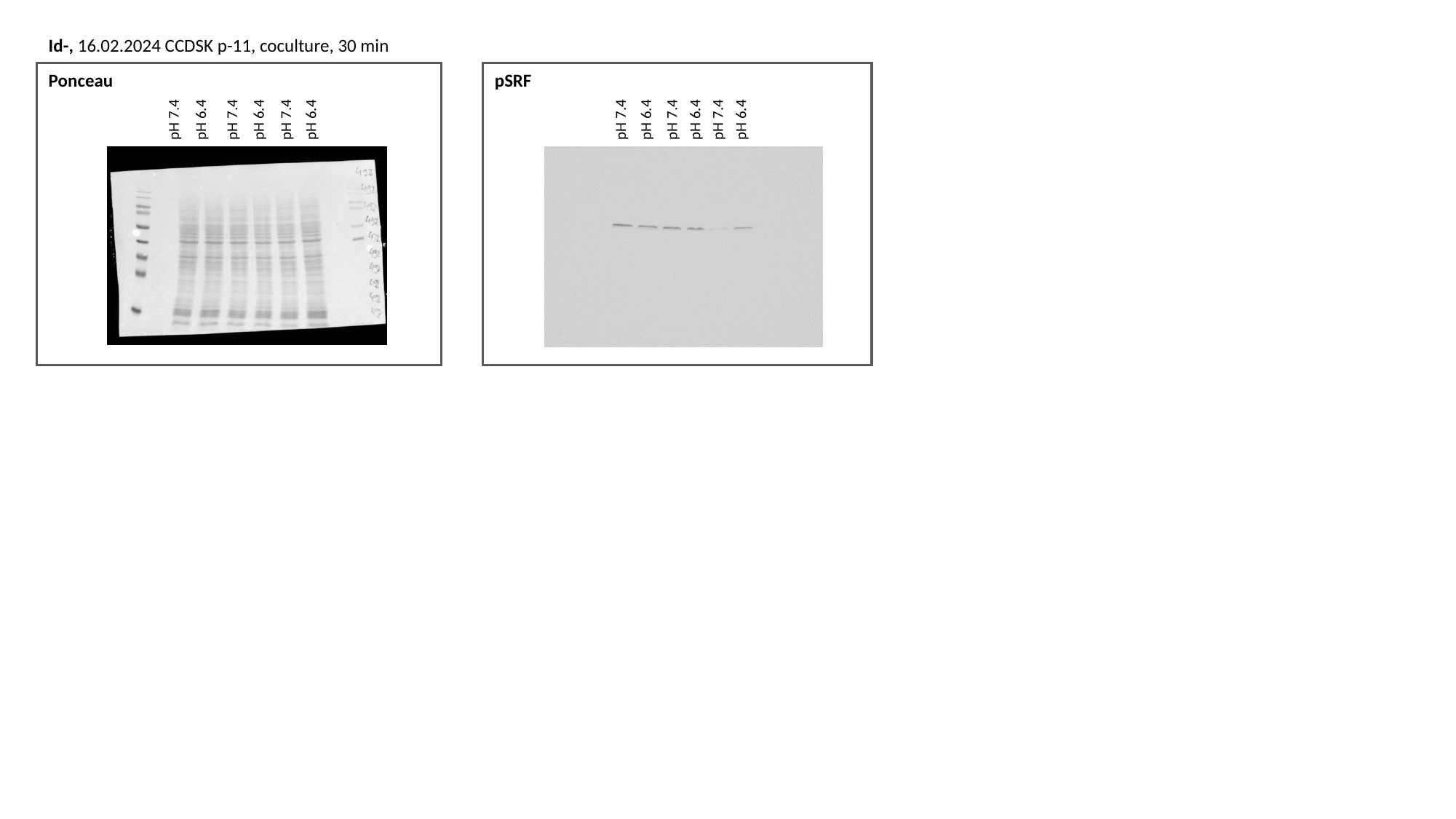

Id-, 16.02.2024 CCDSK p-11, coculture, 30 min
pSRF
Ponceau
pH 6.4
pH 6.4
pH 6.4
pH 6.4
pH 6.4
pH 6.4
pH 7.4
pH 7.4
pH 7.4
pH 7.4
pH 7.4
pH 7.4

## Slide 53
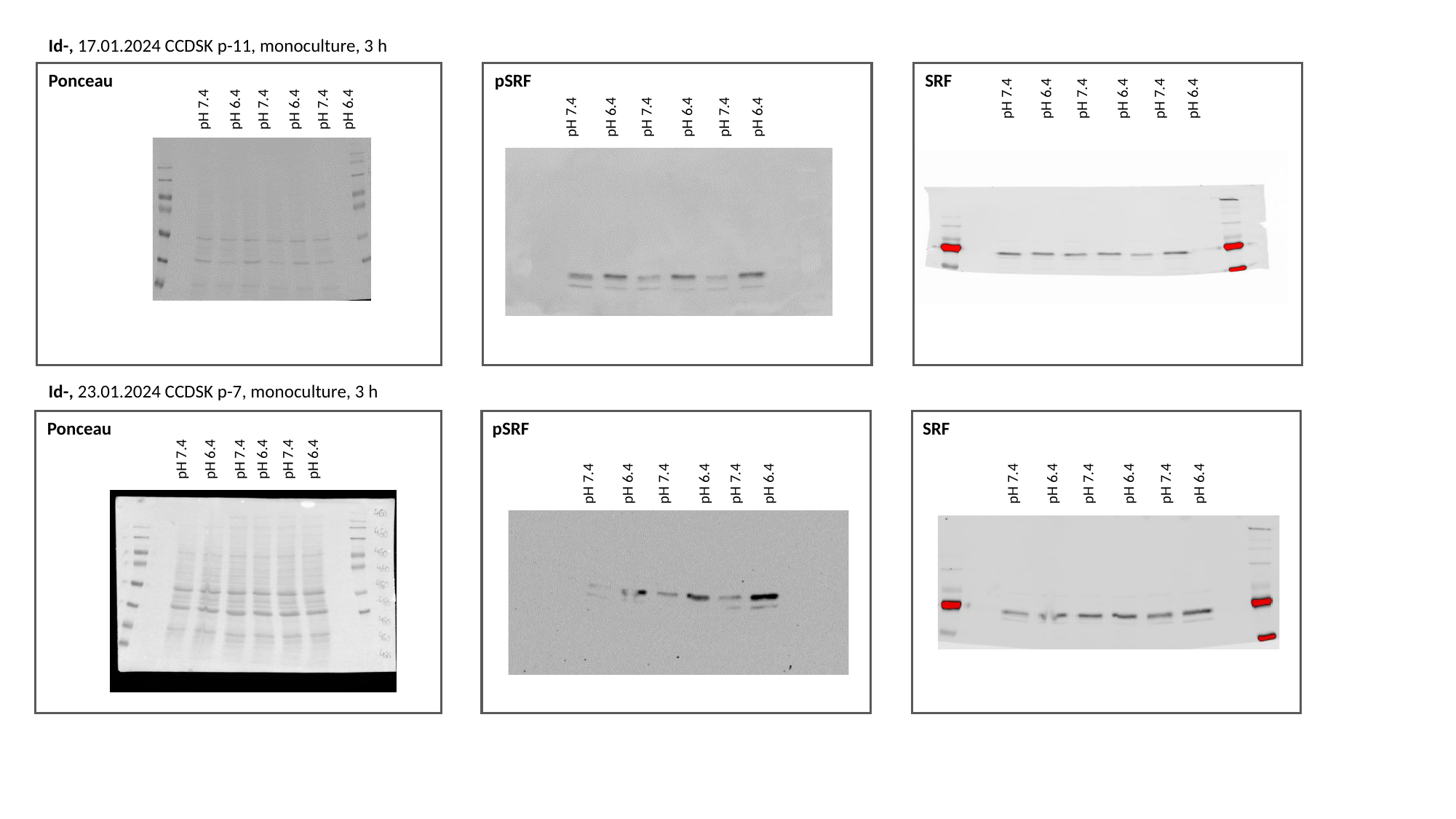

Id-, 17.01.2024 CCDSK p-11, monoculture, 3 h
pSRF
SRF
Ponceau
pH 6.4
pH 6.4
pH 6.4
pH 7.4
pH 7.4
pH 7.4
pH 6.4
pH 6.4
pH 6.4
pH 6.4
pH 6.4
pH 6.4
pH 7.4
pH 7.4
pH 7.4
pH 7.4
pH 7.4
pH 7.4
Id-, 23.01.2024 CCDSK p-7, monoculture, 3 h
Ponceau
pSRF
SRF
pH 6.4
pH 6.4
pH 6.4
pH 7.4
pH 7.4
pH 7.4
pH 6.4
pH 6.4
pH 6.4
pH 6.4
pH 6.4
pH 6.4
pH 7.4
pH 7.4
pH 7.4
pH 7.4
pH 7.4
pH 7.4

## Slide 54
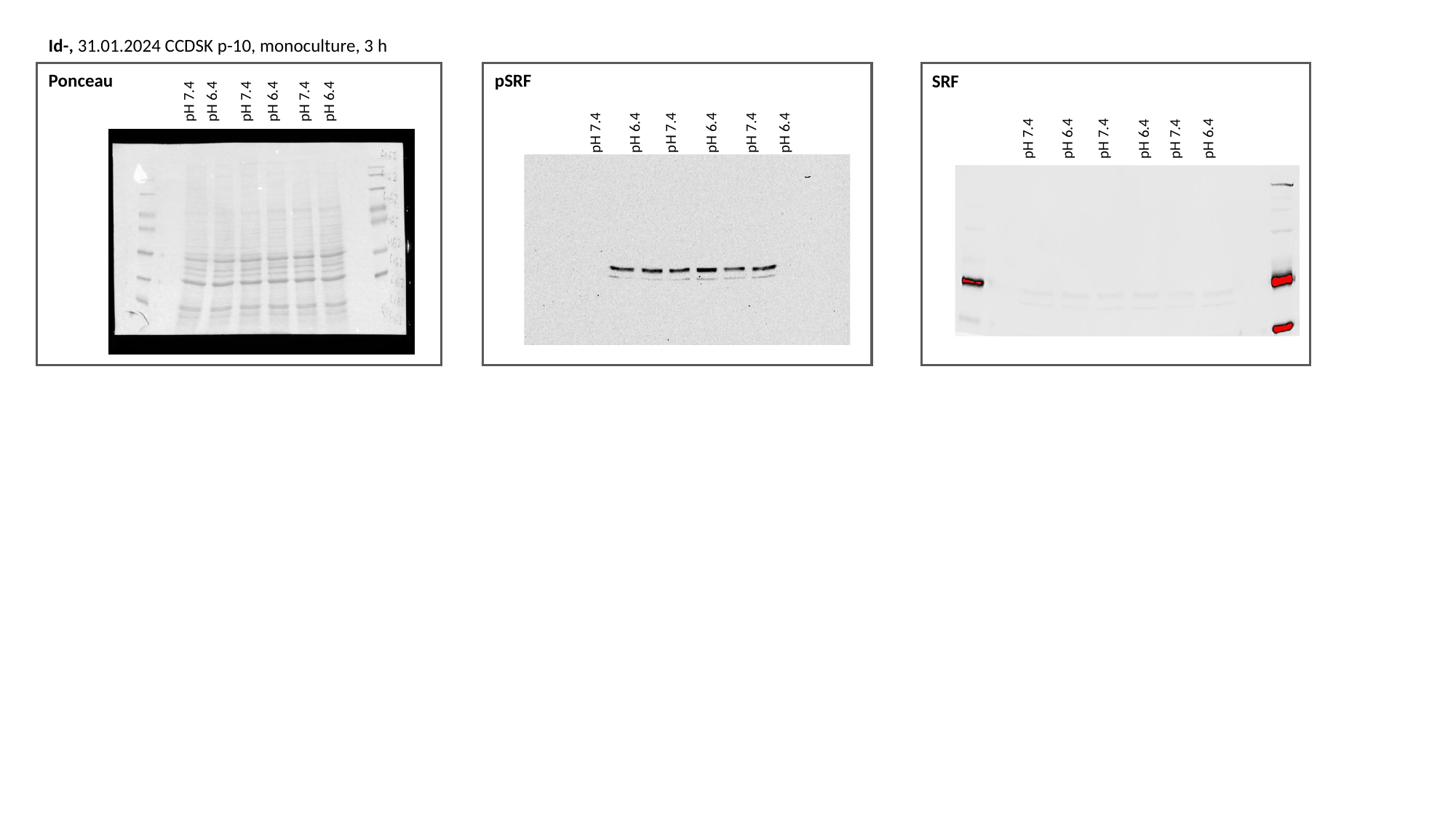

Id-, 31.01.2024 CCDSK p-10, monoculture, 3 h
pSRF
Ponceau
SRF
pH 6.4
pH 6.4
pH 6.4
pH 7.4
pH 7.4
pH 7.4
pH 6.4
pH 6.4
pH 6.4
pH 6.4
pH 6.4
pH 6.4
pH 7.4
pH 7.4
pH 7.4
pH 7.4
pH 7.4
pH 7.4

## Slide 55
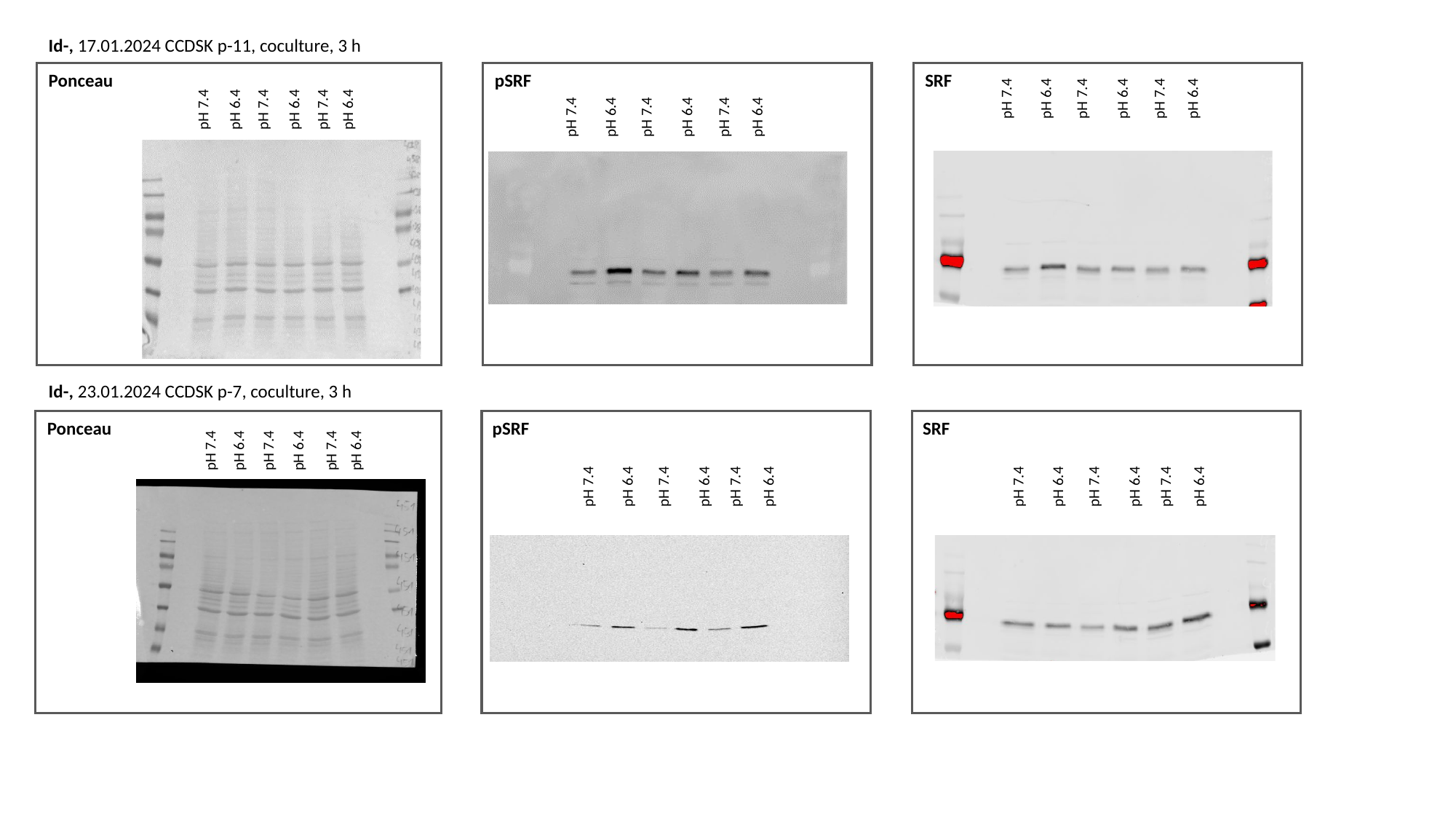

Id-, 17.01.2024 CCDSK p-11, coculture, 3 h
pSRF
SRF
Ponceau
pH 6.4
pH 6.4
pH 6.4
pH 7.4
pH 7.4
pH 7.4
pH 6.4
pH 6.4
pH 6.4
pH 6.4
pH 6.4
pH 6.4
pH 7.4
pH 7.4
pH 7.4
pH 7.4
pH 7.4
pH 7.4
Id-, 23.01.2024 CCDSK p-7, coculture, 3 h
Ponceau
pSRF
SRF
pH 6.4
pH 6.4
pH 6.4
pH 7.4
pH 7.4
pH 7.4
pH 6.4
pH 6.4
pH 6.4
pH 6.4
pH 6.4
pH 6.4
pH 7.4
pH 7.4
pH 7.4
pH 7.4
pH 7.4
pH 7.4

## Slide 56
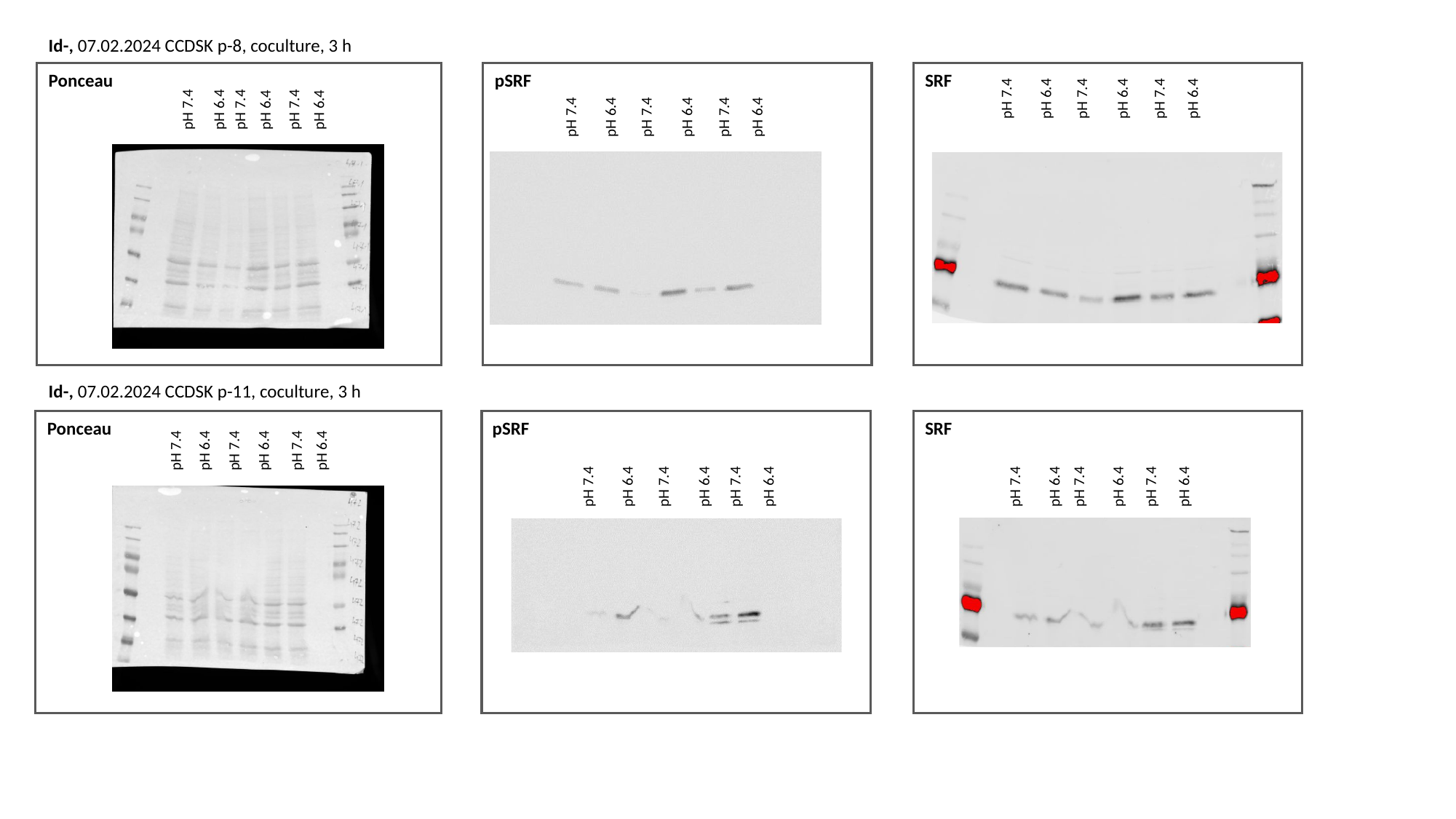

Id-, 07.02.2024 CCDSK p-8, coculture, 3 h
pSRF
SRF
Ponceau
pH 6.4
pH 6.4
pH 6.4
pH 7.4
pH 7.4
pH 7.4
pH 6.4
pH 6.4
pH 6.4
pH 6.4
pH 6.4
pH 6.4
pH 7.4
pH 7.4
pH 7.4
pH 7.4
pH 7.4
pH 7.4
Id-, 07.02.2024 CCDSK p-11, coculture, 3 h
SRF
Ponceau
pSRF
pH 6.4
pH 6.4
pH 6.4
pH 7.4
pH 7.4
pH 7.4
pH 6.4
pH 6.4
pH 6.4
pH 6.4
pH 6.4
pH 6.4
pH 7.4
pH 7.4
pH 7.4
pH 7.4
pH 7.4
pH 7.4

## Slide 57
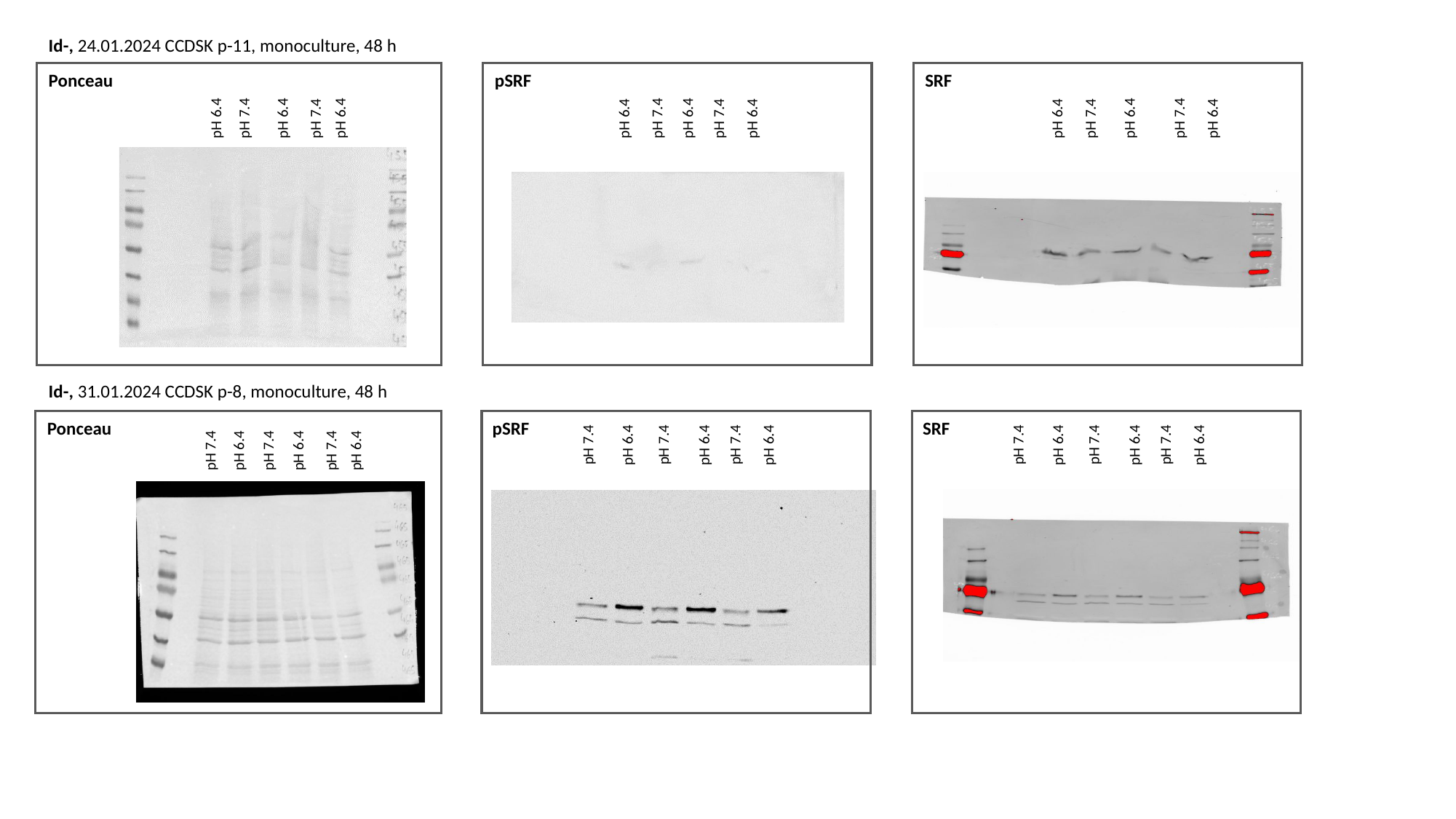

Id-, 24.01.2024 CCDSK p-11, monoculture, 48 h
pSRF
SRF
Ponceau
pH 6.4
pH 6.4
pH 6.4
pH 6.4
pH 6.4
pH 6.4
pH 6.4
pH 6.4
pH 6.4
pH 7.4
pH 7.4
pH 7.4
pH 7.4
pH 7.4
pH 7.4
Id-, 31.01.2024 CCDSK p-8, monoculture, 48 h
Ponceau
pSRF
SRF
pH 6.4
pH 6.4
pH 6.4
pH 6.4
pH 6.4
pH 6.4
pH 6.4
pH 6.4
pH 6.4
pH 7.4
pH 7.4
pH 7.4
pH 7.4
pH 7.4
pH 7.4
pH 7.4
pH 7.4
pH 7.4

## Slide 58
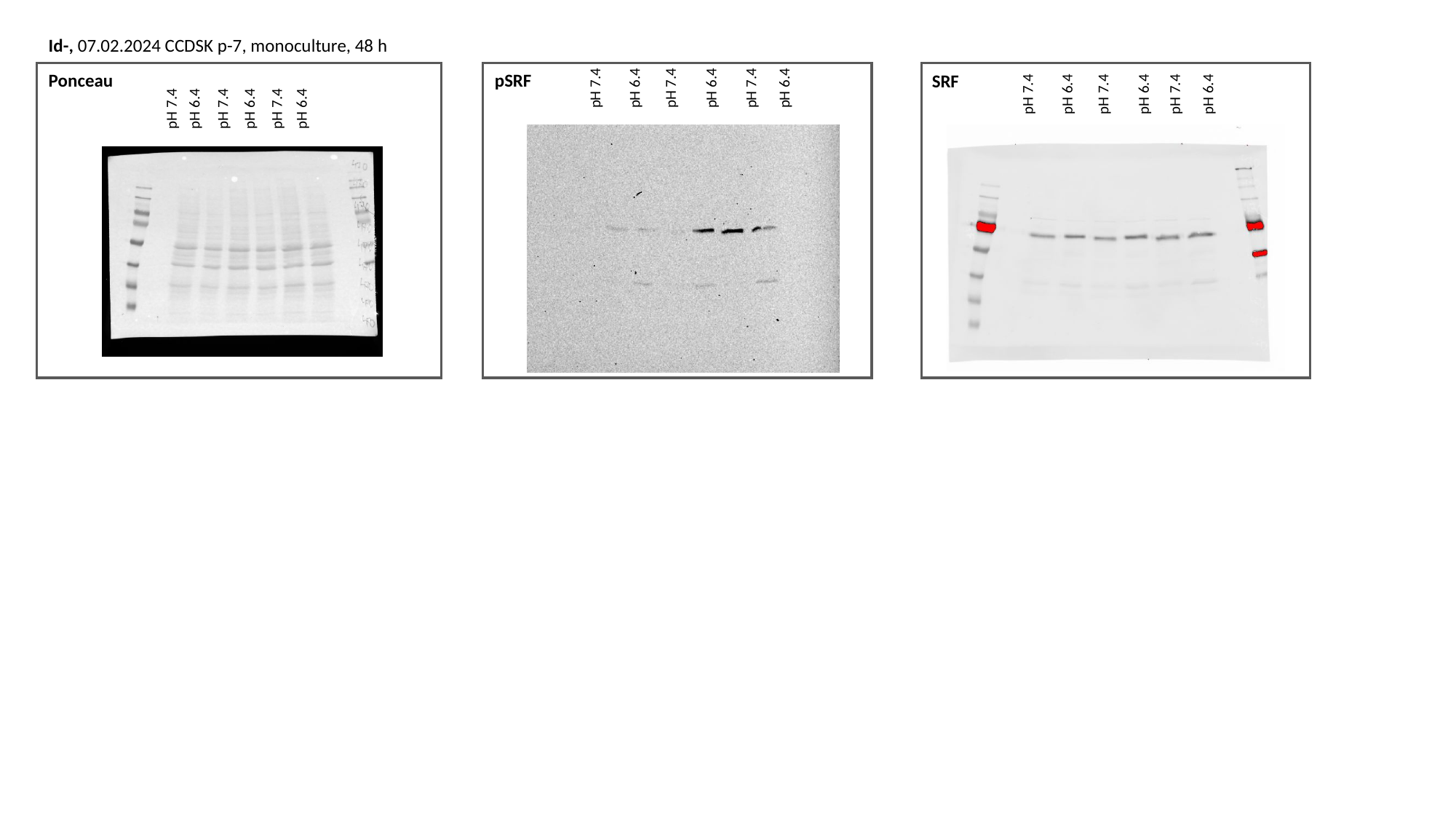

Id-, 07.02.2024 CCDSK p-7, monoculture, 48 h
pH 6.4
pH 6.4
pH 6.4
pSRF
Ponceau
SRF
pH 6.4
pH 6.4
pH 6.4
pH 7.4
pH 7.4
pH 7.4
pH 7.4
pH 7.4
pH 7.4
pH 6.4
pH 6.4
pH 6.4
pH 7.4
pH 7.4
pH 7.4

## Slide 59
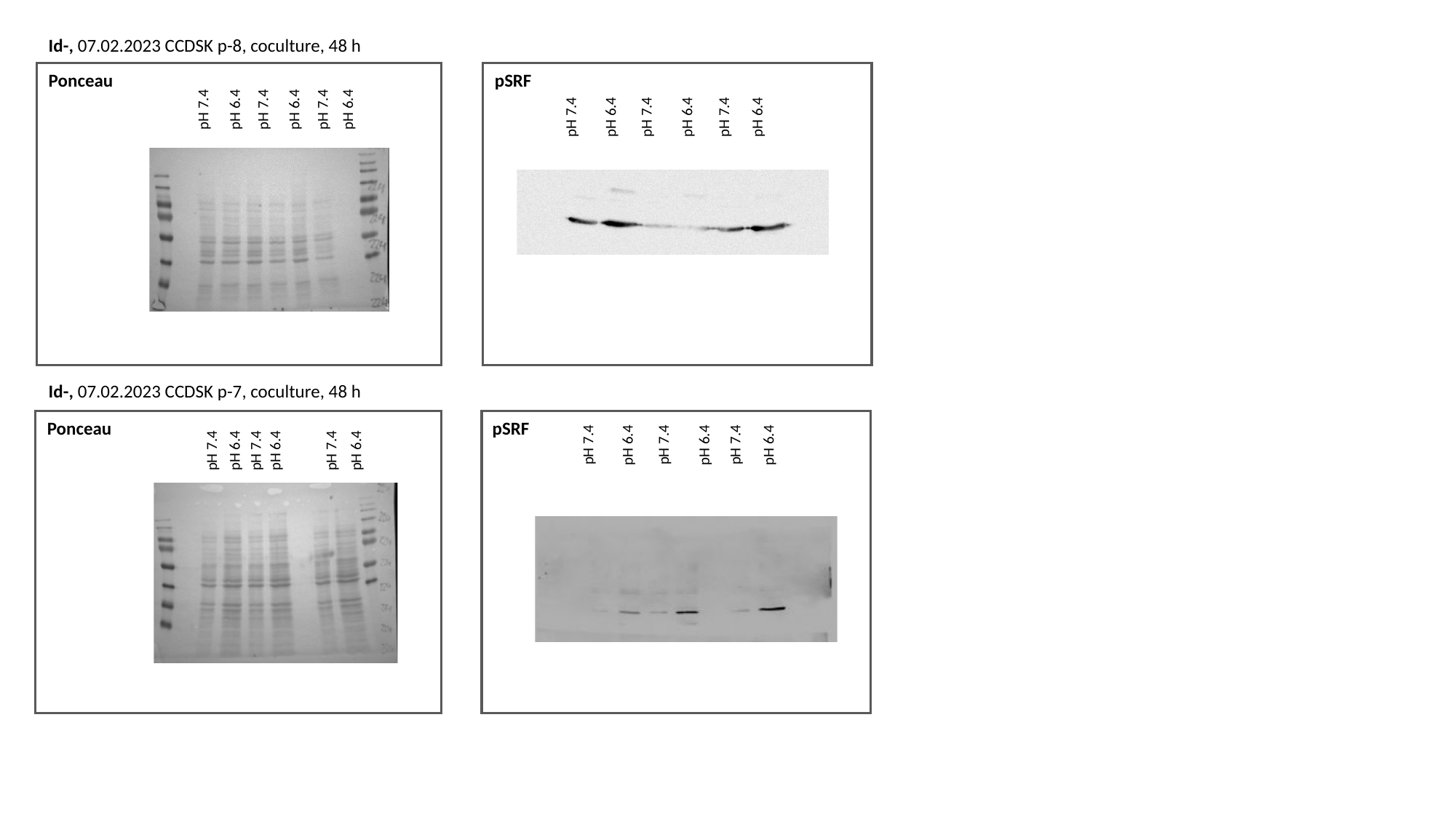

Id-, 07.02.2023 CCDSK p-8, coculture, 48 h
pSRF
Ponceau
pH 6.4
pH 6.4
pH 6.4
pH 6.4
pH 6.4
pH 6.4
pH 7.4
pH 7.4
pH 7.4
pH 7.4
pH 7.4
pH 7.4
Id-, 07.02.2023 CCDSK p-7, coculture, 48 h
Ponceau
pSRF
pH 6.4
pH 6.4
pH 6.4
pH 6.4
pH 6.4
pH 6.4
pH 7.4
pH 7.4
pH 7.4
pH 7.4
pH 7.4
pH 7.4

## Slide 60
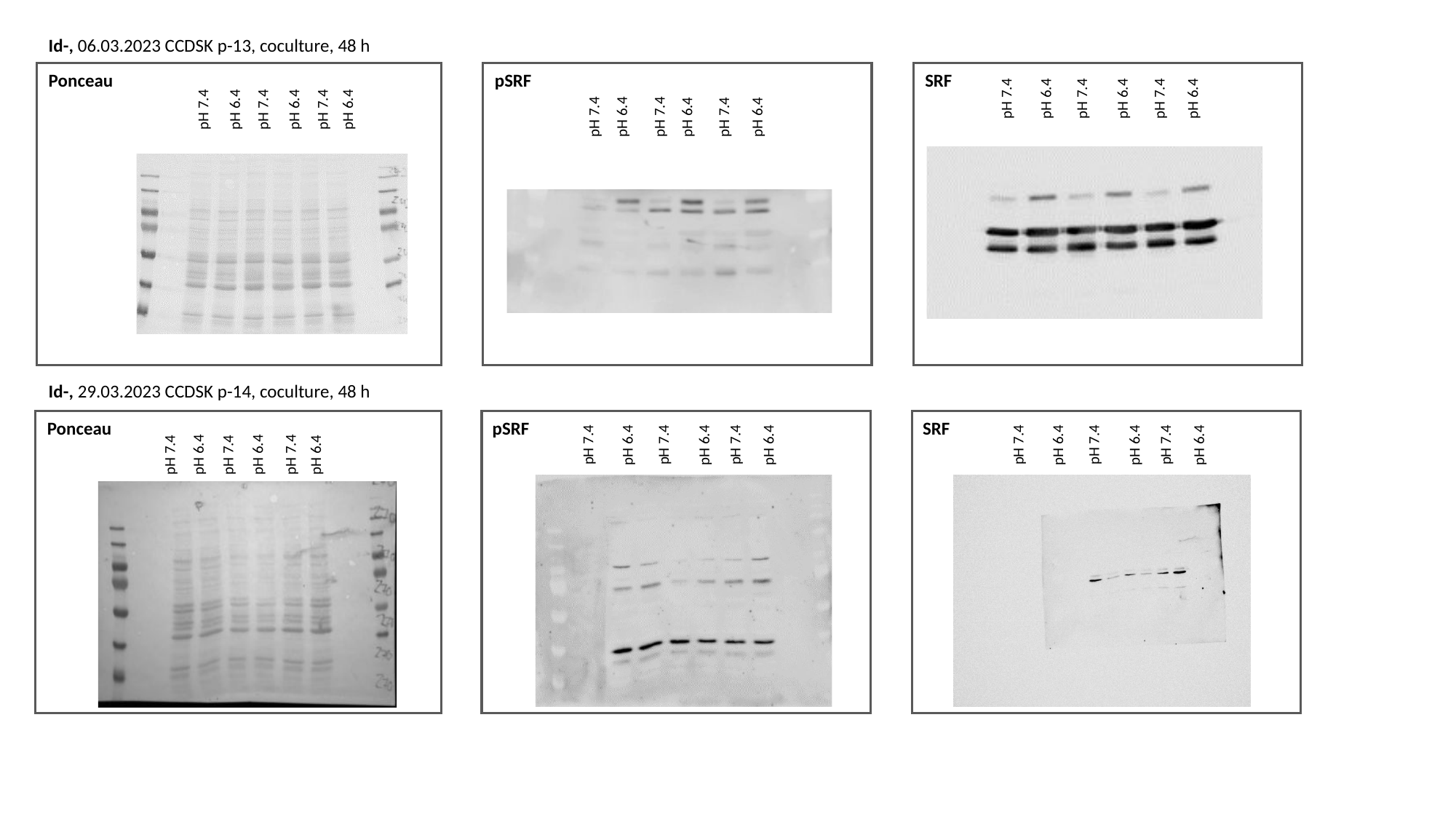

Id-, 06.03.2023 CCDSK p-13, coculture, 48 h
pSRF
SRF
Ponceau
pH 6.4
pH 6.4
pH 6.4
pH 7.4
pH 7.4
pH 7.4
pH 6.4
pH 6.4
pH 6.4
pH 6.4
pH 6.4
pH 6.4
pH 7.4
pH 7.4
pH 7.4
pH 7.4
pH 7.4
pH 7.4
Id-, 29.03.2023 CCDSK p-14, coculture, 48 h
Ponceau
pSRF
SRF
pH 6.4
pH 6.4
pH 6.4
pH 6.4
pH 6.4
pH 6.4
pH 7.4
pH 7.4
pH 7.4
pH 7.4
pH 7.4
pH 7.4
pH 6.4
pH 6.4
pH 6.4
pH 7.4
pH 7.4
pH 7.4

## Slide 61
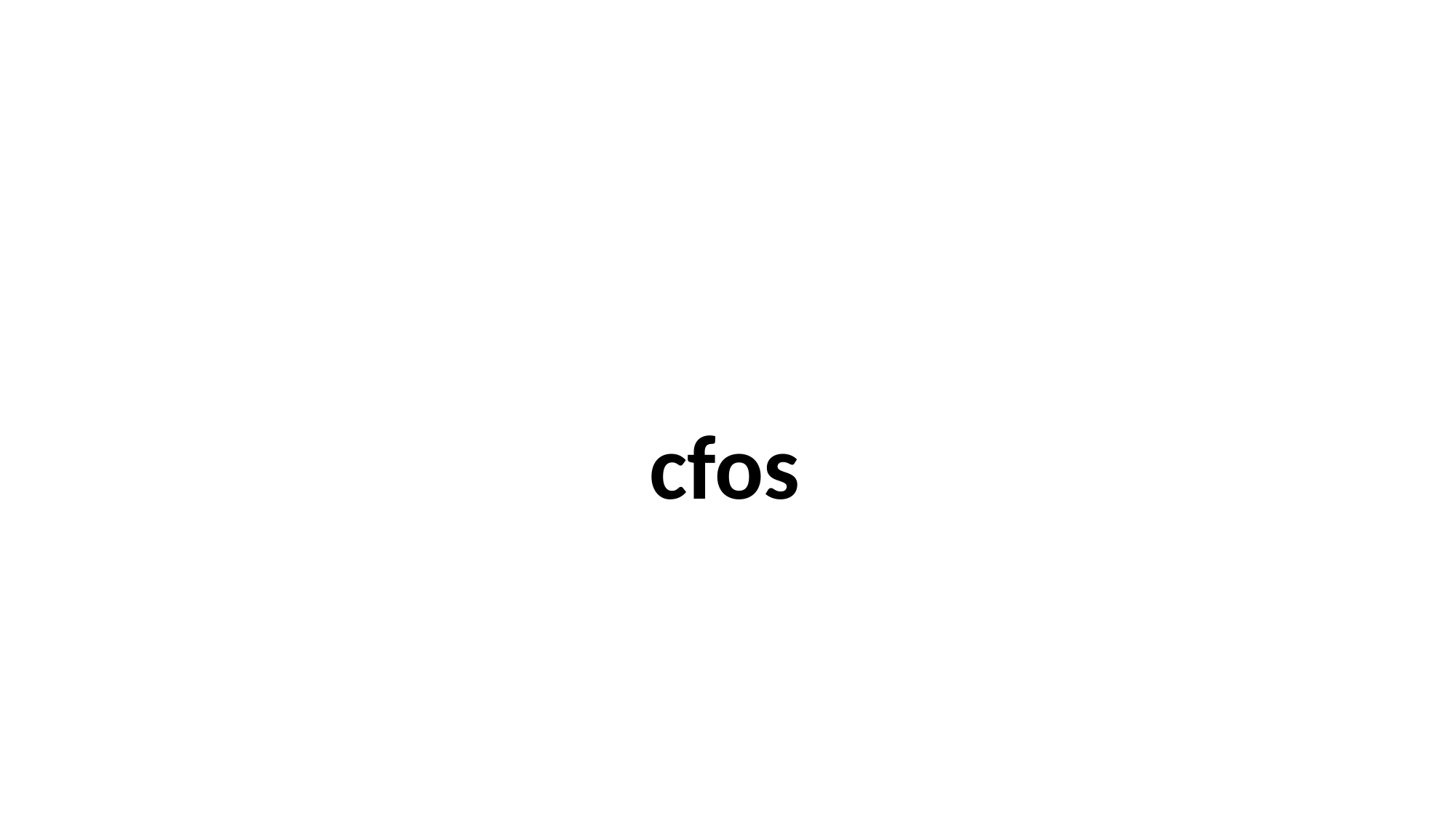

cfos

## Slide 62
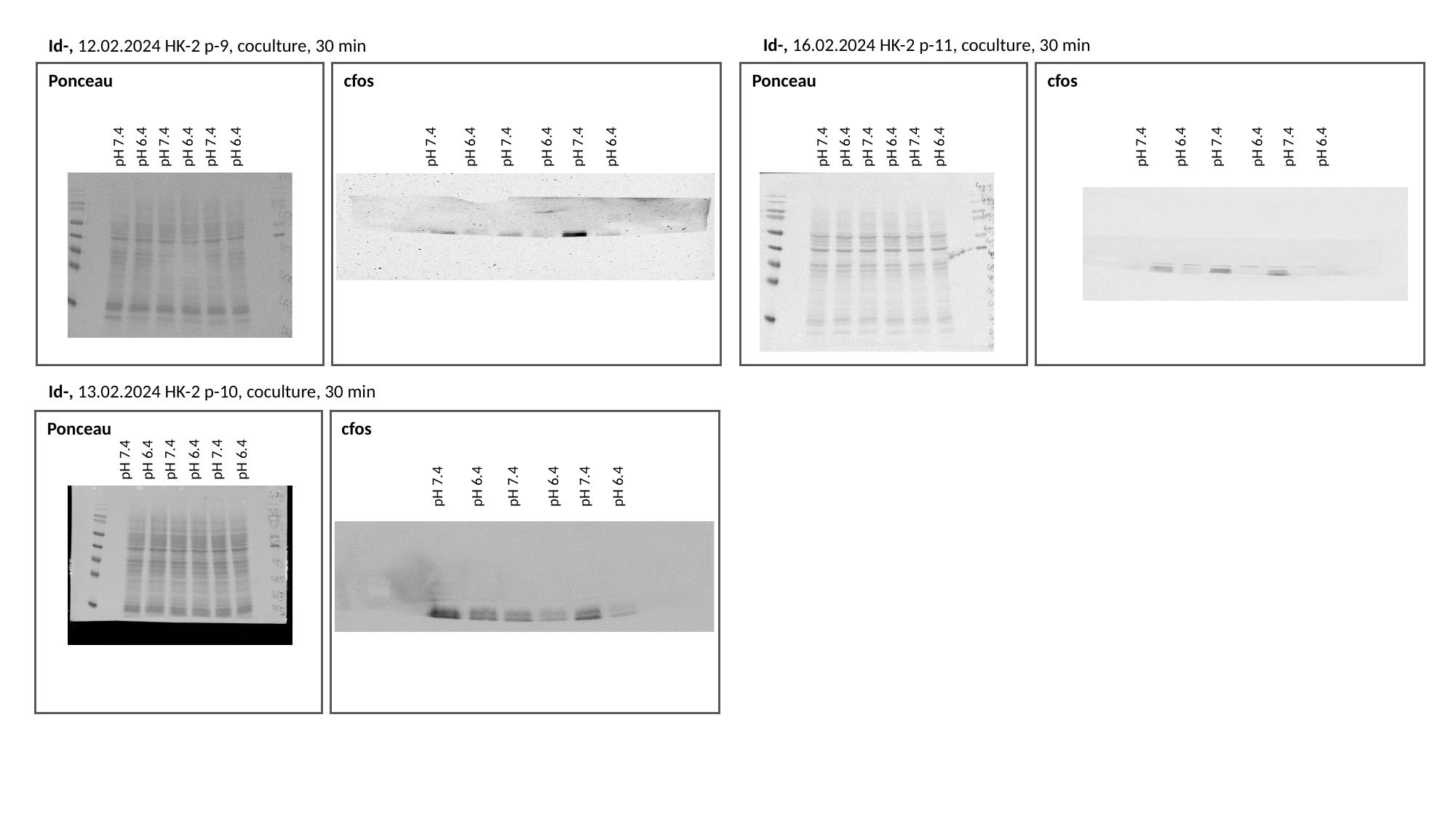

Id-, 16.02.2024 HK-2 p-11, coculture, 30 min
Id-, 12.02.2024 HK-2 p-9, coculture, 30 min
cfos
cfos
Ponceau
Ponceau
pH 6.4
pH 6.4
pH 6.4
pH 6.4
pH 6.4
pH 6.4
pH 6.4
pH 6.4
pH 6.4
pH 6.4
pH 6.4
pH 6.4
pH 7.4
pH 7.4
pH 7.4
pH 7.4
pH 7.4
pH 7.4
pH 7.4
pH 7.4
pH 7.4
pH 7.4
pH 7.4
pH 7.4
Id-, 13.02.2024 HK-2 p-10, coculture, 30 min
Ponceau
cfos
pH 6.4
pH 6.4
pH 6.4
pH 7.4
pH 7.4
pH 7.4
pH 6.4
pH 6.4
pH 6.4
pH 7.4
pH 7.4
pH 7.4

## Slide 63
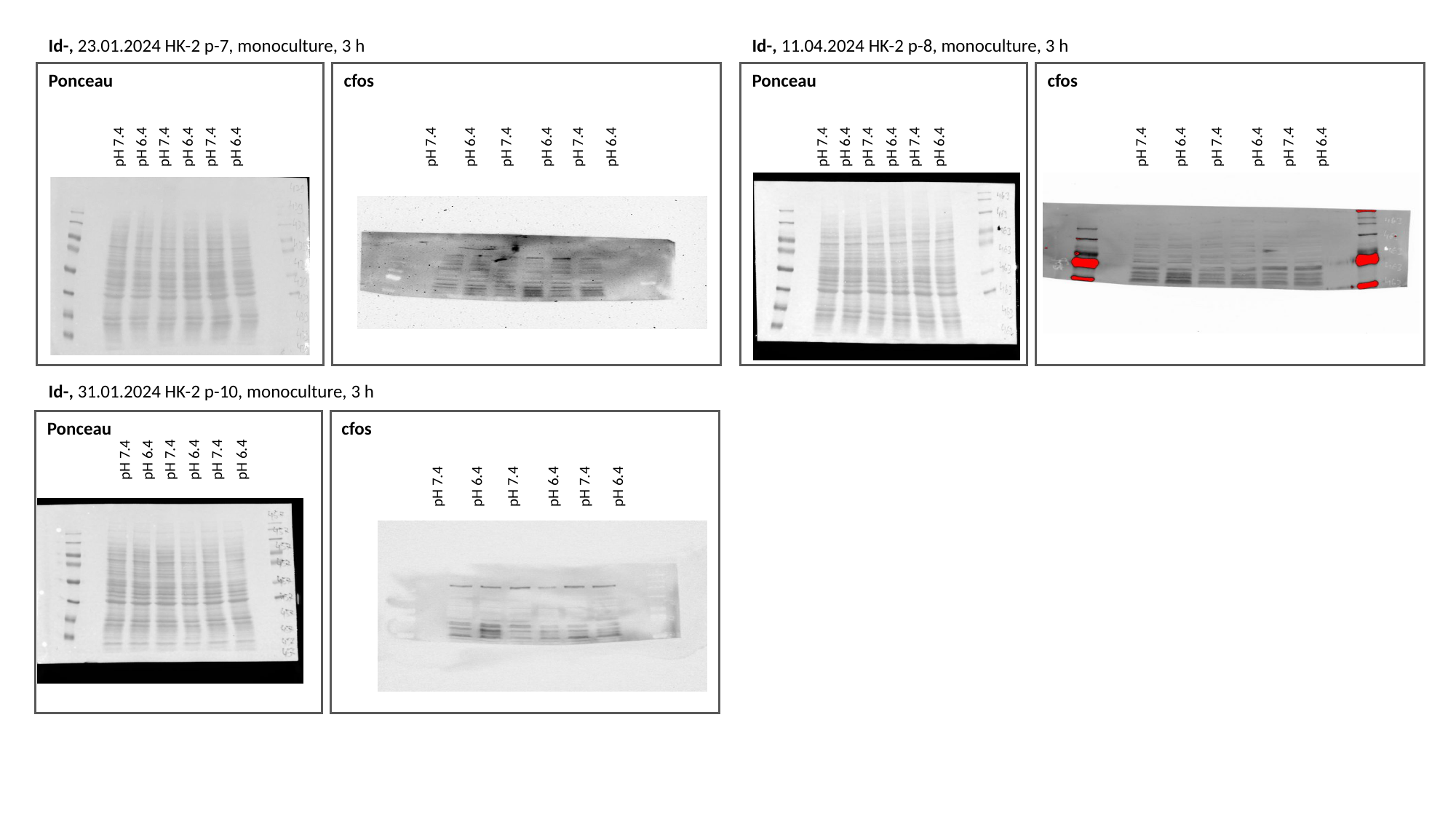

Id-, 23.01.2024 HK-2 p-7, monoculture, 3 h
Id-, 11.04.2024 HK-2 p-8, monoculture, 3 h
cfos
cfos
Ponceau
Ponceau
pH 6.4
pH 6.4
pH 6.4
pH 6.4
pH 6.4
pH 6.4
pH 6.4
pH 6.4
pH 6.4
pH 6.4
pH 6.4
pH 6.4
pH 7.4
pH 7.4
pH 7.4
pH 7.4
pH 7.4
pH 7.4
pH 7.4
pH 7.4
pH 7.4
pH 7.4
pH 7.4
pH 7.4
Id-, 31.01.2024 HK-2 p-10, monoculture, 3 h
Ponceau
cfos
pH 6.4
pH 6.4
pH 6.4
pH 7.4
pH 7.4
pH 7.4
pH 6.4
pH 6.4
pH 6.4
pH 7.4
pH 7.4
pH 7.4

## Slide 64
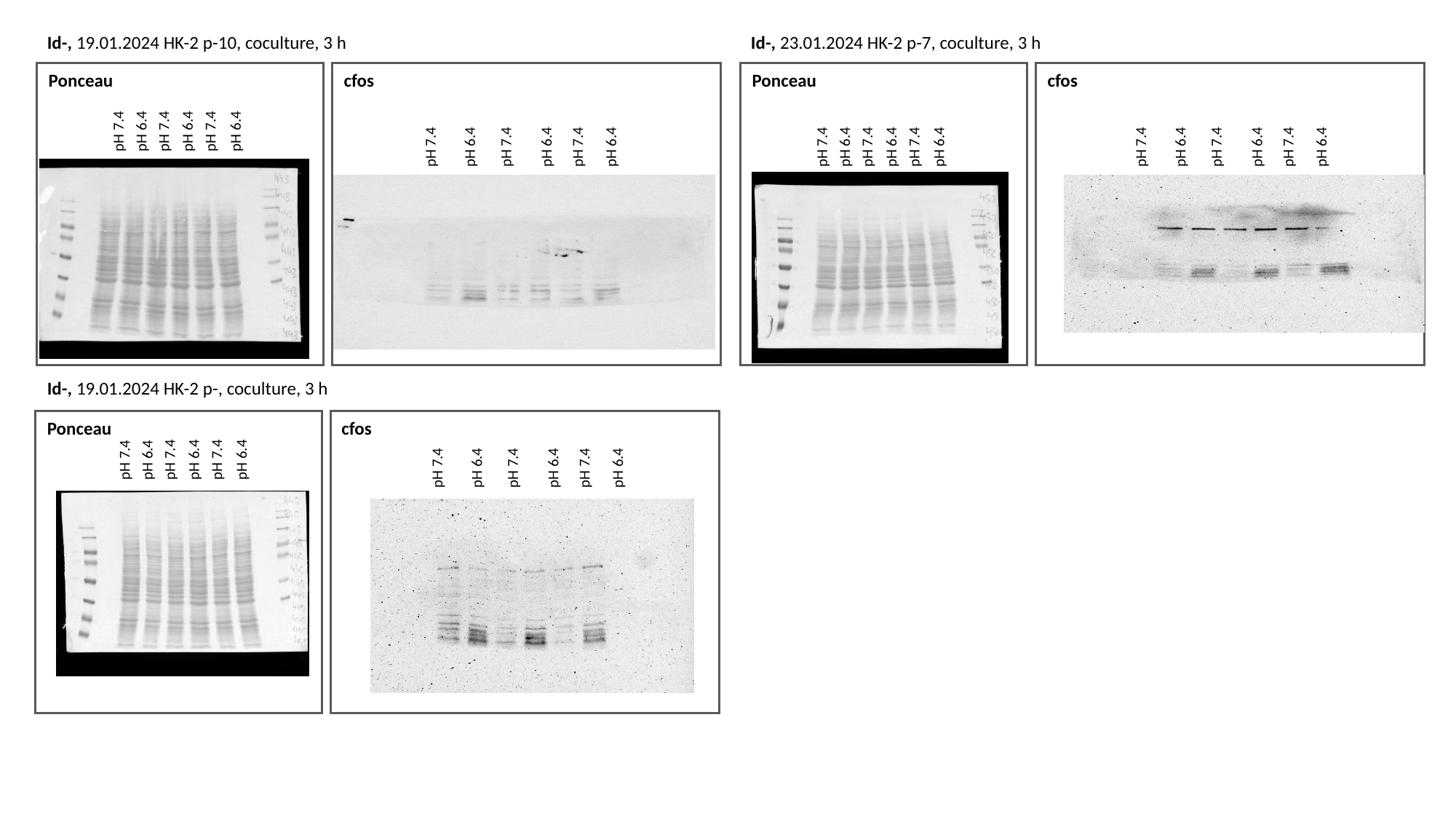

Id-, 19.01.2024 HK-2 p-10, coculture, 3 h
Id-, 23.01.2024 HK-2 p-7, coculture, 3 h
cfos
cfos
Ponceau
Ponceau
pH 6.4
pH 6.4
pH 6.4
pH 7.4
pH 7.4
pH 7.4
pH 6.4
pH 6.4
pH 6.4
pH 6.4
pH 6.4
pH 6.4
pH 6.4
pH 6.4
pH 6.4
pH 7.4
pH 7.4
pH 7.4
pH 7.4
pH 7.4
pH 7.4
pH 7.4
pH 7.4
pH 7.4
Id-, 19.01.2024 HK-2 p-, coculture, 3 h
Ponceau
cfos
pH 6.4
pH 6.4
pH 6.4
pH 6.4
pH 6.4
pH 6.4
pH 7.4
pH 7.4
pH 7.4
pH 7.4
pH 7.4
pH 7.4

## Slide 65
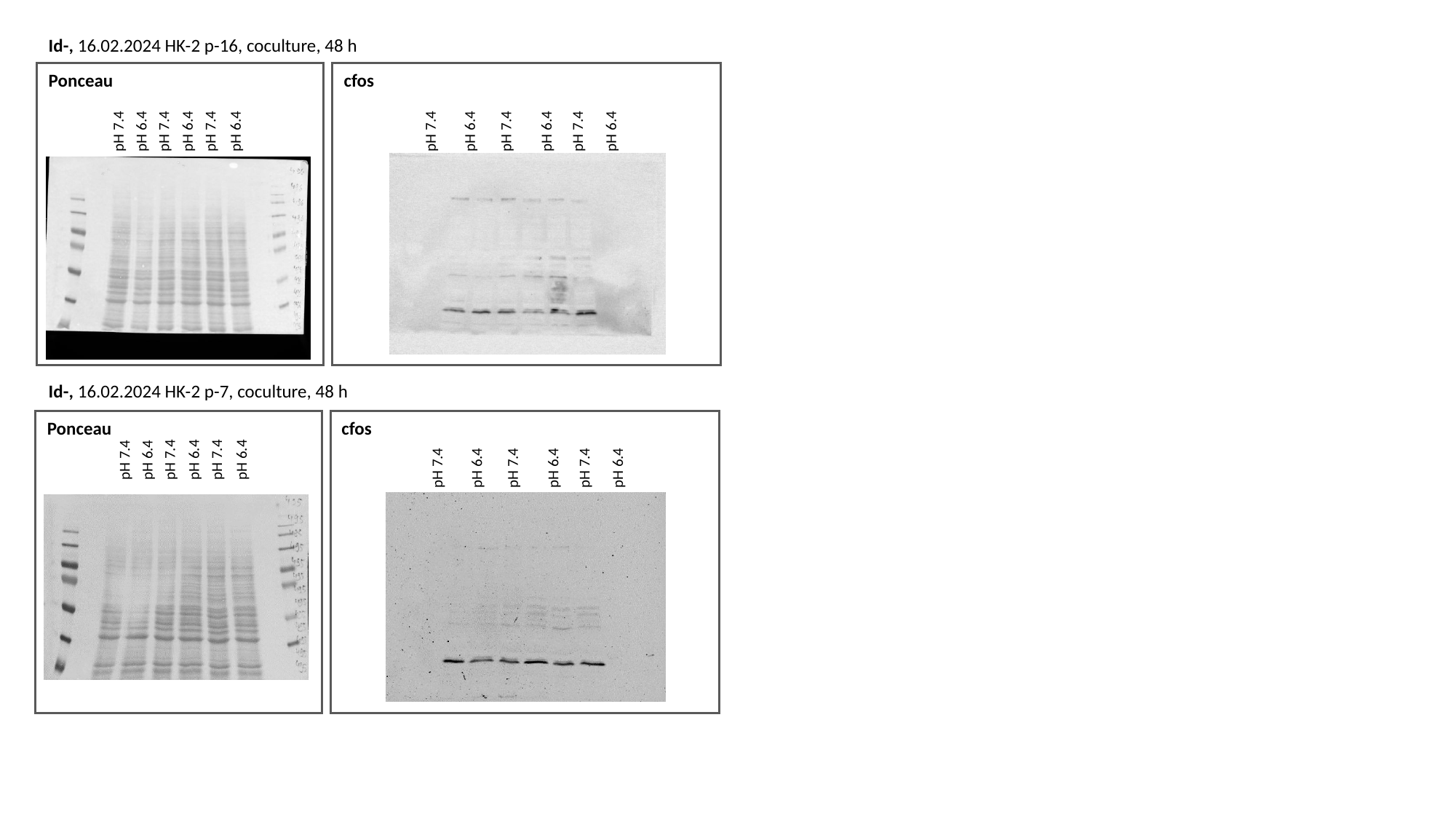

Id-, 16.02.2024 HK-2 p-16, coculture, 48 h
Ponceau
cfos
pH 6.4
pH 6.4
pH 6.4
pH 6.4
pH 6.4
pH 6.4
pH 7.4
pH 7.4
pH 7.4
pH 7.4
pH 7.4
pH 7.4
Id-, 16.02.2024 HK-2 p-7, coculture, 48 h
Ponceau
cfos
pH 6.4
pH 6.4
pH 6.4
pH 6.4
pH 6.4
pH 6.4
pH 7.4
pH 7.4
pH 7.4
pH 7.4
pH 7.4
pH 7.4

## Slide 66
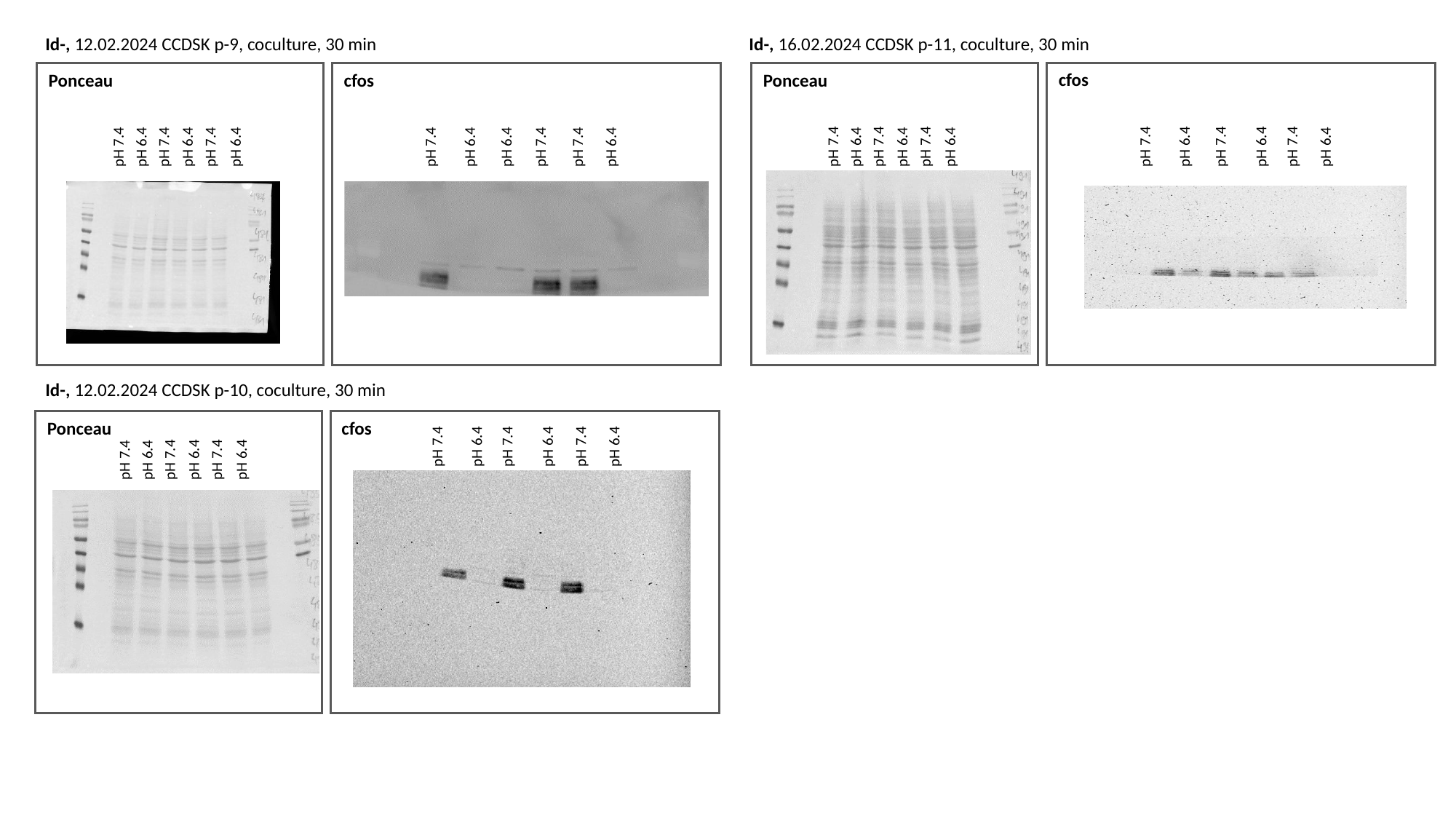

Id-, 12.02.2024 CCDSK p-9, coculture, 30 min
Id-, 16.02.2024 CCDSK p-11, coculture, 30 min
cfos
Ponceau
cfos
Ponceau
pH 6.4
pH 6.4
pH 6.4
pH 6.4
pH 6.4
pH 6.4
pH 6.4
pH 6.4
pH 6.4
pH 6.4
pH 6.4
pH 6.4
pH 7.4
pH 7.4
pH 7.4
pH 7.4
pH 7.4
pH 7.4
pH 7.4
pH 7.4
pH 7.4
pH 7.4
pH 7.4
pH 7.4
Id-, 12.02.2024 CCDSK p-10, coculture, 30 min
Ponceau
cfos
pH 6.4
pH 6.4
pH 6.4
pH 7.4
pH 7.4
pH 7.4
pH 6.4
pH 6.4
pH 6.4
pH 7.4
pH 7.4
pH 7.4

## Slide 67
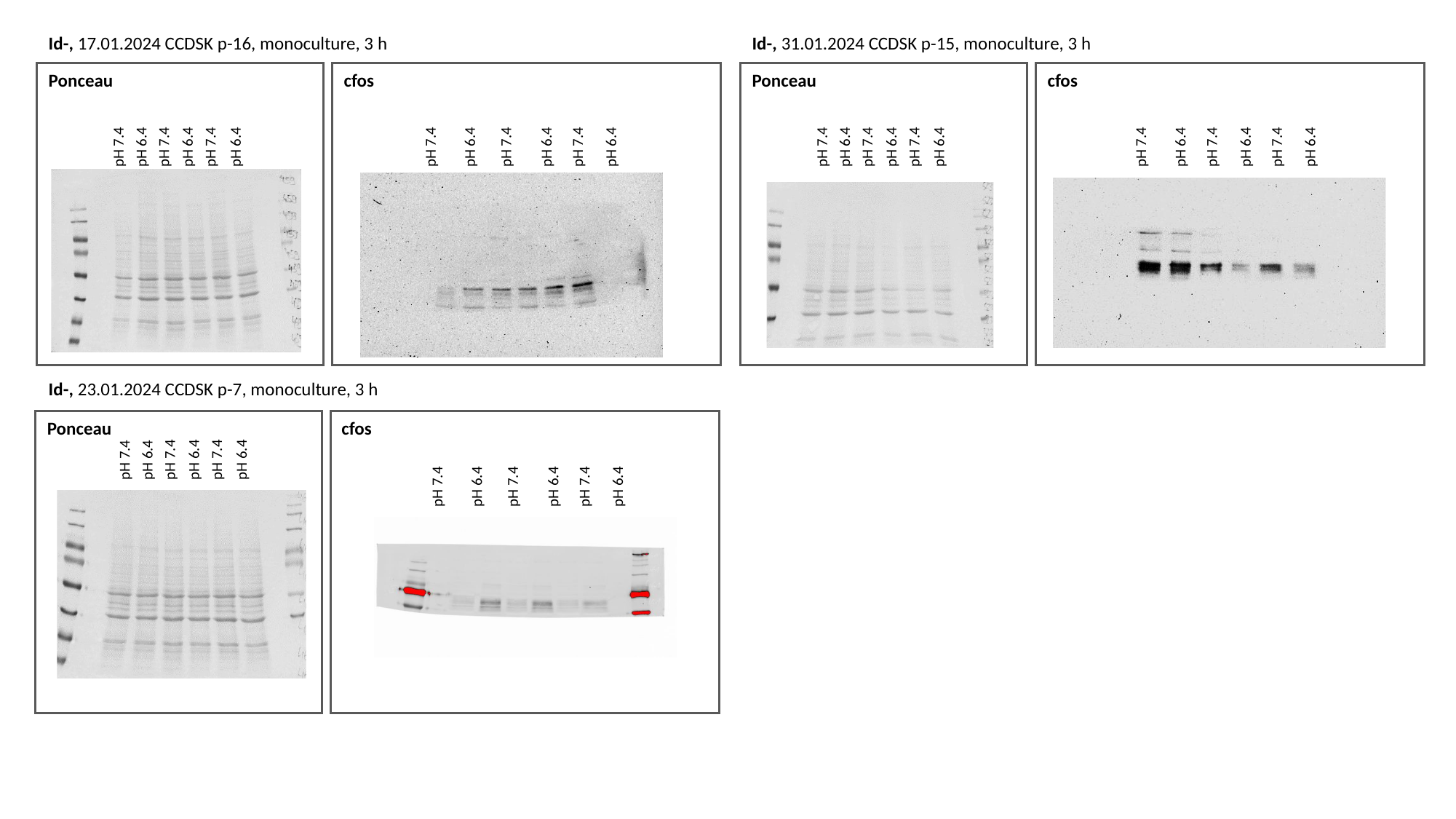

Id-, 17.01.2024 CCDSK p-16, monoculture, 3 h
Id-, 31.01.2024 CCDSK p-15, monoculture, 3 h
cfos
cfos
Ponceau
Ponceau
pH 6.4
pH 6.4
pH 6.4
pH 6.4
pH 6.4
pH 6.4
pH 6.4
pH 6.4
pH 6.4
pH 6.4
pH 6.4
pH 6.4
pH 7.4
pH 7.4
pH 7.4
pH 7.4
pH 7.4
pH 7.4
pH 7.4
pH 7.4
pH 7.4
pH 7.4
pH 7.4
pH 7.4
Id-, 23.01.2024 CCDSK p-7, monoculture, 3 h
Ponceau
cfos
pH 6.4
pH 6.4
pH 6.4
pH 7.4
pH 7.4
pH 7.4
pH 6.4
pH 6.4
pH 6.4
pH 7.4
pH 7.4
pH 7.4

## Slide 68
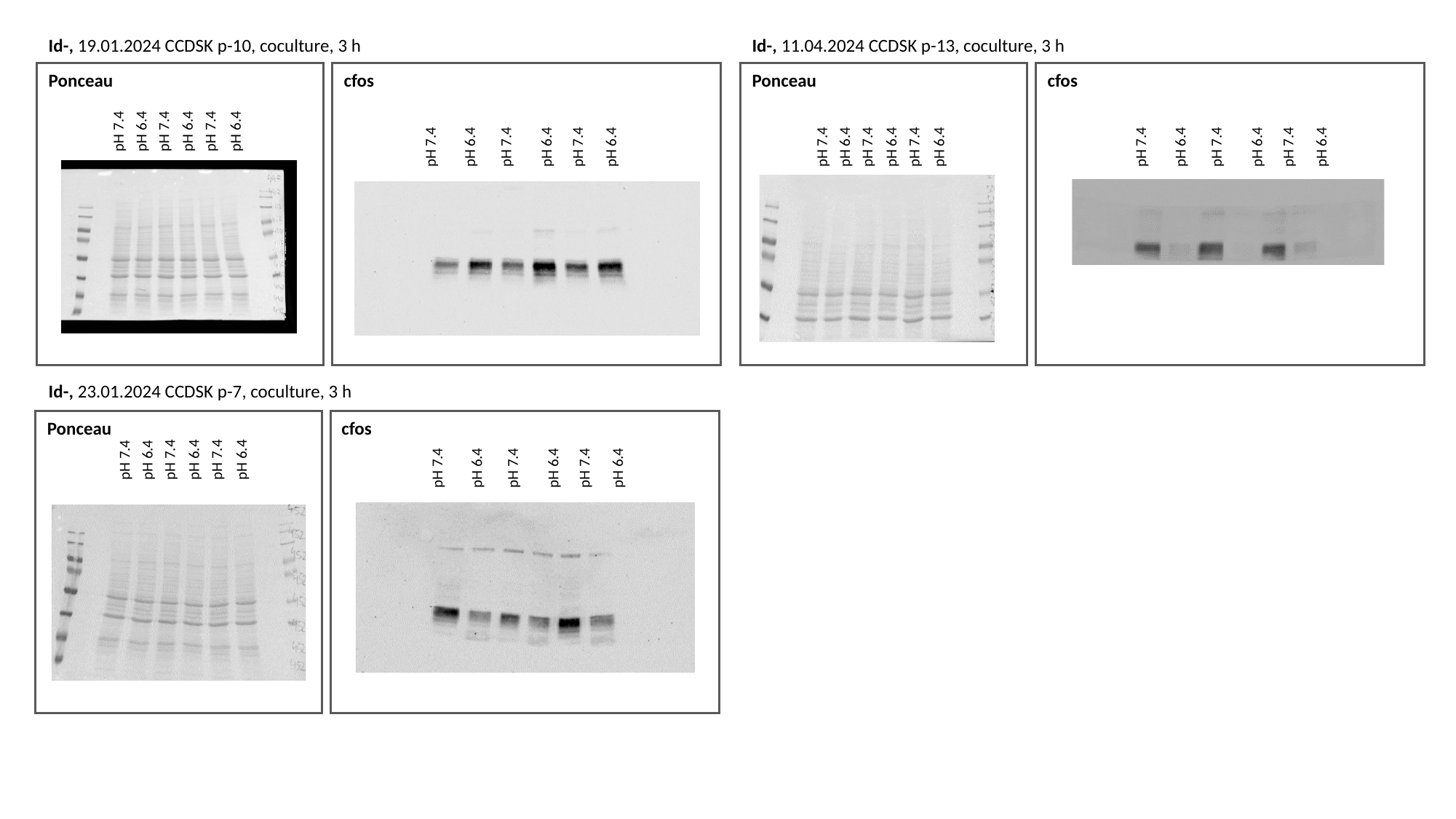

Id-, 19.01.2024 CCDSK p-10, coculture, 3 h
Id-, 11.04.2024 CCDSK p-13, coculture, 3 h
cfos
cfos
Ponceau
Ponceau
pH 6.4
pH 6.4
pH 6.4
pH 7.4
pH 7.4
pH 7.4
pH 6.4
pH 6.4
pH 6.4
pH 6.4
pH 6.4
pH 6.4
pH 6.4
pH 6.4
pH 6.4
pH 7.4
pH 7.4
pH 7.4
pH 7.4
pH 7.4
pH 7.4
pH 7.4
pH 7.4
pH 7.4
Id-, 23.01.2024 CCDSK p-7, coculture, 3 h
Ponceau
cfos
pH 6.4
pH 6.4
pH 6.4
pH 6.4
pH 6.4
pH 6.4
pH 7.4
pH 7.4
pH 7.4
pH 7.4
pH 7.4
pH 7.4

## Slide 69
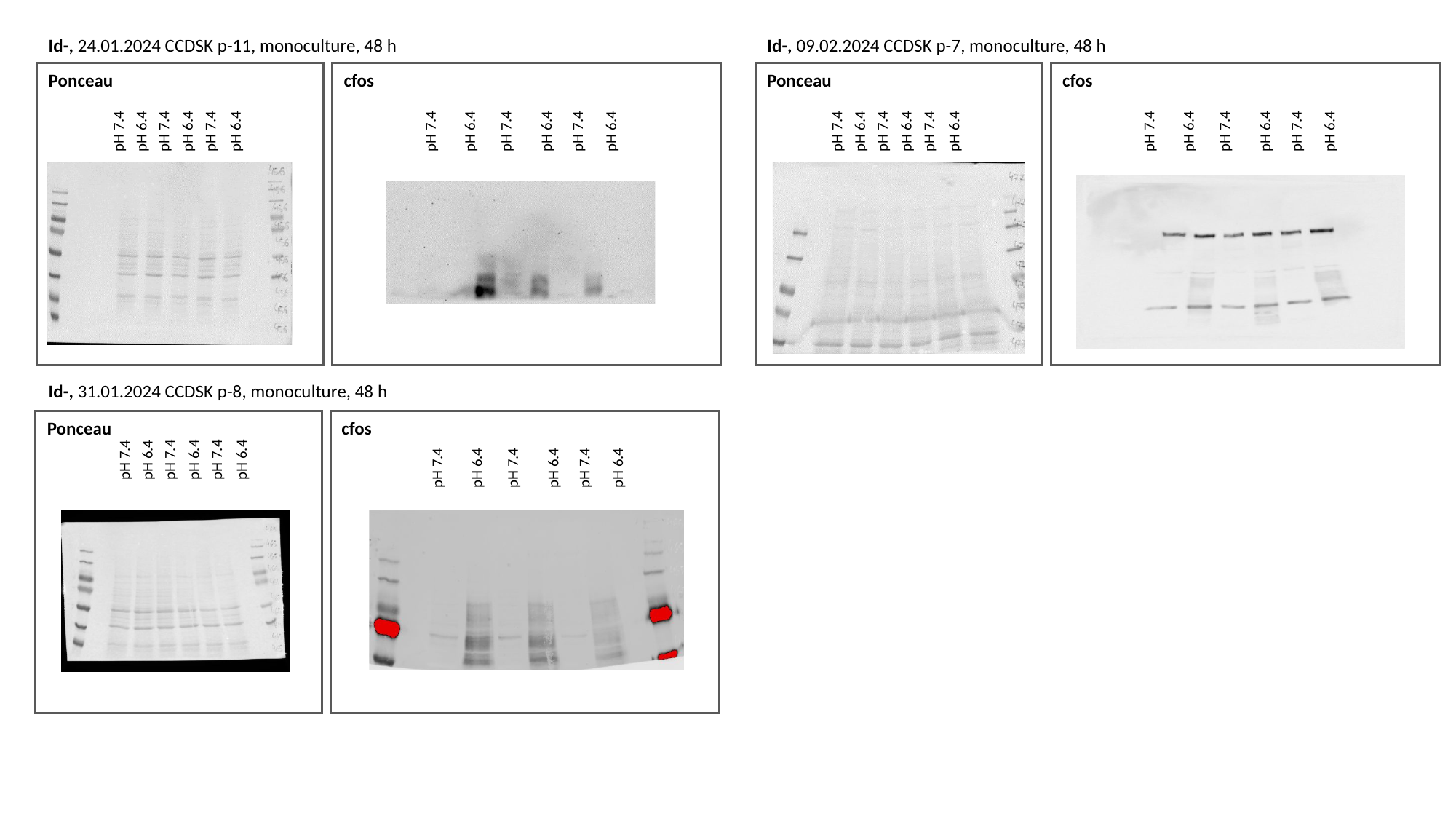

Id-, 24.01.2024 CCDSK p-11, monoculture, 48 h
Id-, 09.02.2024 CCDSK p-7, monoculture, 48 h
Ponceau
cfos
Ponceau
cfos
pH 6.4
pH 6.4
pH 6.4
pH 6.4
pH 6.4
pH 6.4
pH 6.4
pH 6.4
pH 6.4
pH 6.4
pH 6.4
pH 6.4
pH 7.4
pH 7.4
pH 7.4
pH 7.4
pH 7.4
pH 7.4
pH 7.4
pH 7.4
pH 7.4
pH 7.4
pH 7.4
pH 7.4
Id-, 31.01.2024 CCDSK p-8, monoculture, 48 h
Ponceau
cfos
pH 6.4
pH 6.4
pH 6.4
pH 6.4
pH 6.4
pH 6.4
pH 7.4
pH 7.4
pH 7.4
pH 7.4
pH 7.4
pH 7.4

## Slide 70
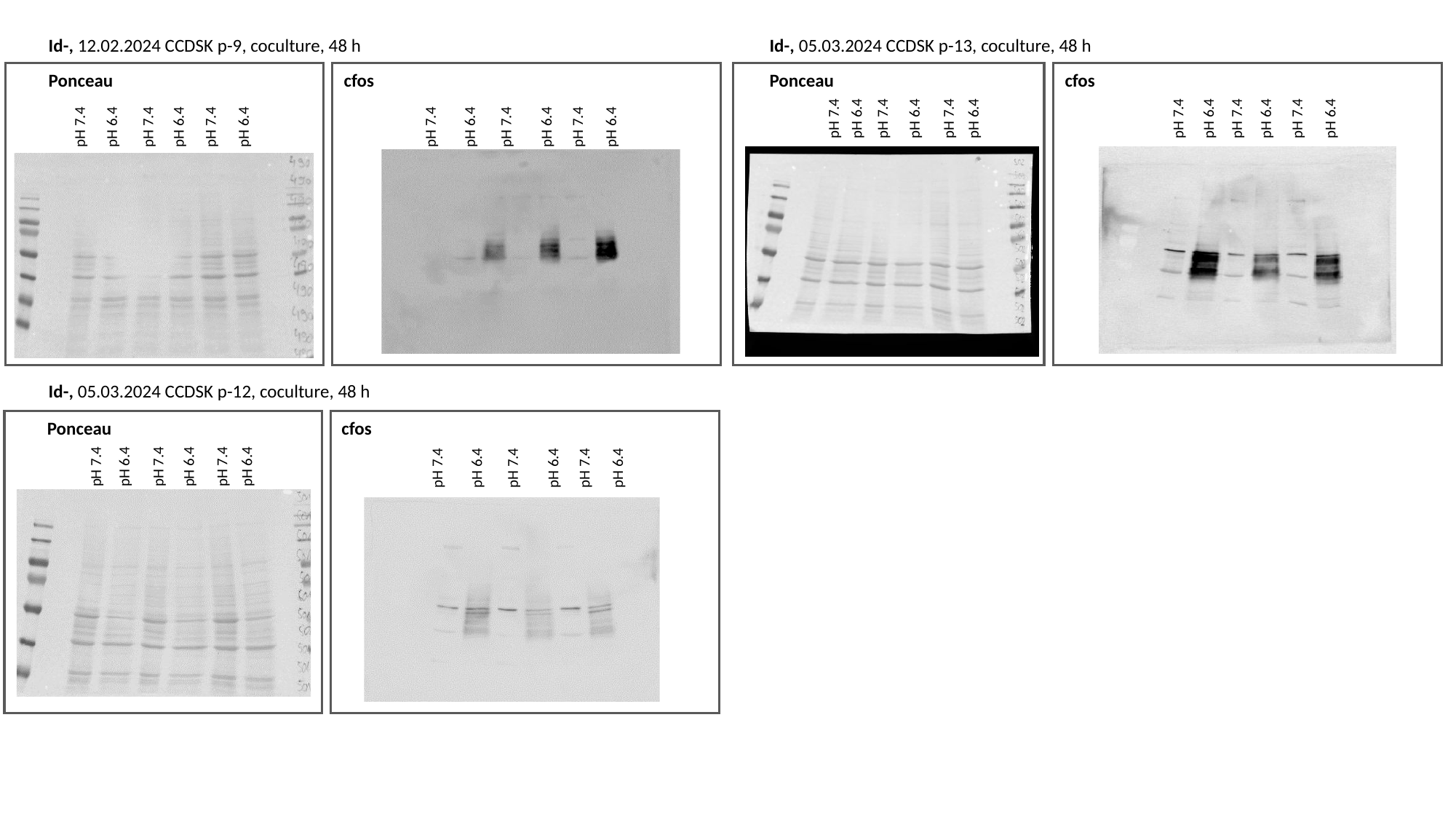

Id-, 12.02.2024 CCDSK p-9, coculture, 48 h
Id-, 05.03.2024 CCDSK p-13, coculture, 48 h
Ponceau
cfos
Ponceau
cfos
pH 6.4
pH 6.4
pH 6.4
pH 6.4
pH 6.4
pH 6.4
pH 6.4
pH 6.4
pH 6.4
pH 6.4
pH 6.4
pH 6.4
pH 7.4
pH 7.4
pH 7.4
pH 7.4
pH 7.4
pH 7.4
pH 7.4
pH 7.4
pH 7.4
pH 7.4
pH 7.4
pH 7.4
Id-, 05.03.2024 CCDSK p-12, coculture, 48 h
Ponceau
cfos
pH 6.4
pH 6.4
pH 6.4
pH 6.4
pH 6.4
pH 6.4
pH 7.4
pH 7.4
pH 7.4
pH 7.4
pH 7.4
pH 7.4

## Slide 71
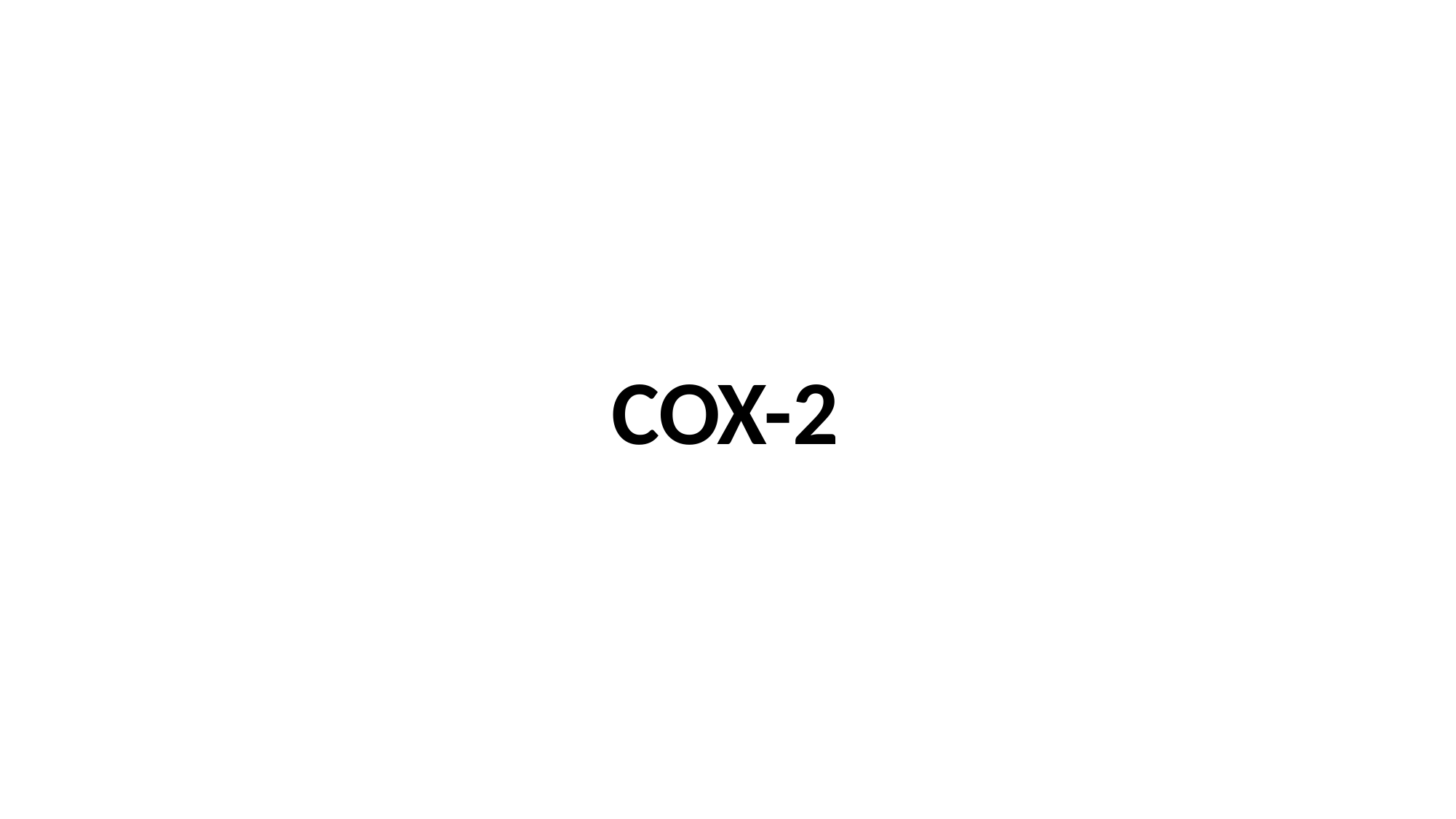

COX-2

## Slide 72
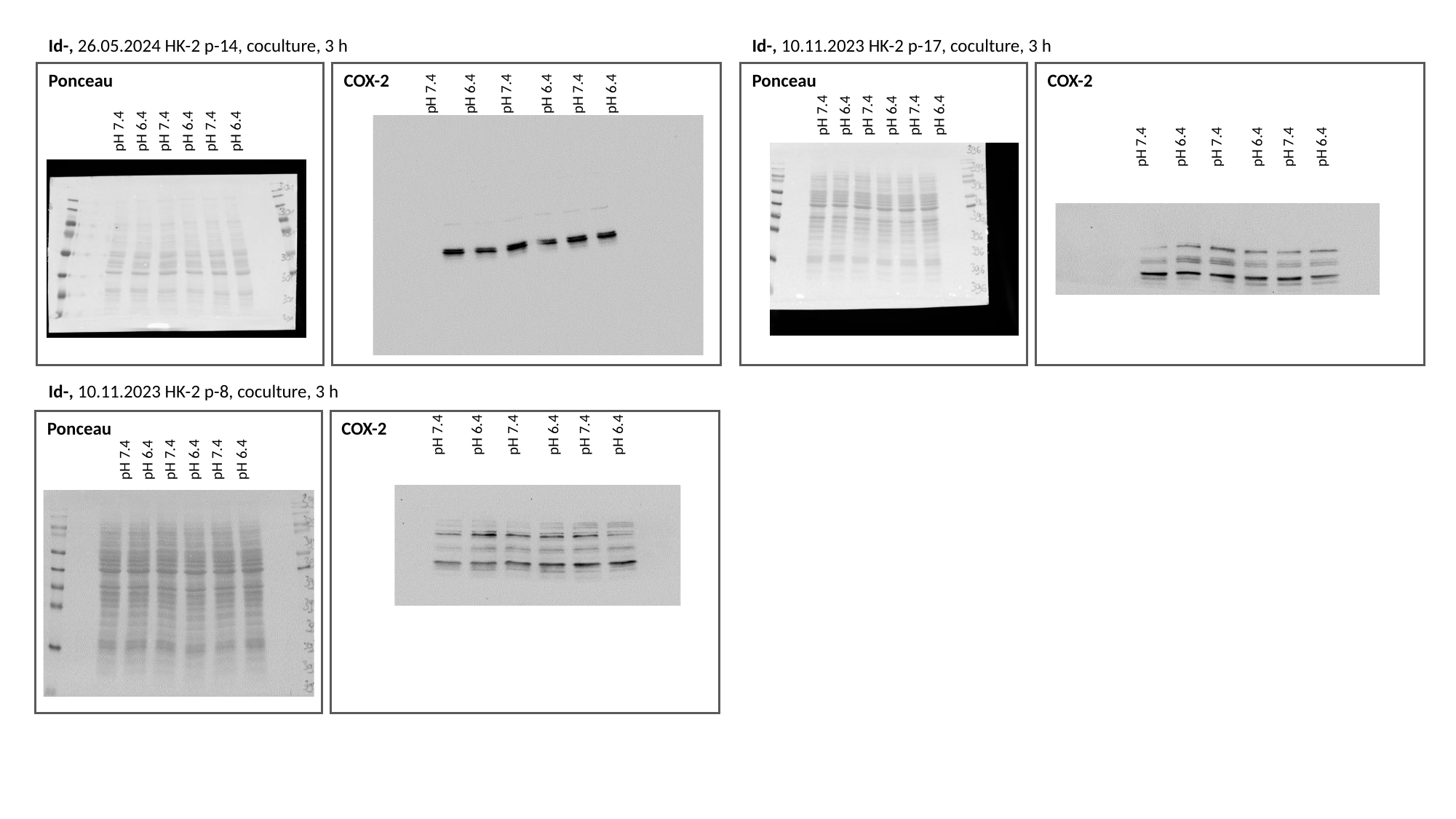

Id-, 26.05.2024 HK-2 p-14, coculture, 3 h
Id-, 10.11.2023 HK-2 p-17, coculture, 3 h
COX-2
COX-2
Ponceau
Ponceau
pH 6.4
pH 6.4
pH 6.4
pH 7.4
pH 7.4
pH 7.4
pH 6.4
pH 6.4
pH 6.4
pH 7.4
pH 7.4
pH 7.4
pH 6.4
pH 6.4
pH 6.4
pH 7.4
pH 7.4
pH 7.4
pH 6.4
pH 6.4
pH 6.4
pH 7.4
pH 7.4
pH 7.4
Id-, 10.11.2023 HK-2 p-8, coculture, 3 h
pH 6.4
pH 6.4
pH 6.4
Ponceau
COX-2
pH 7.4
pH 7.4
pH 7.4
pH 6.4
pH 6.4
pH 6.4
pH 7.4
pH 7.4
pH 7.4

## Slide 73
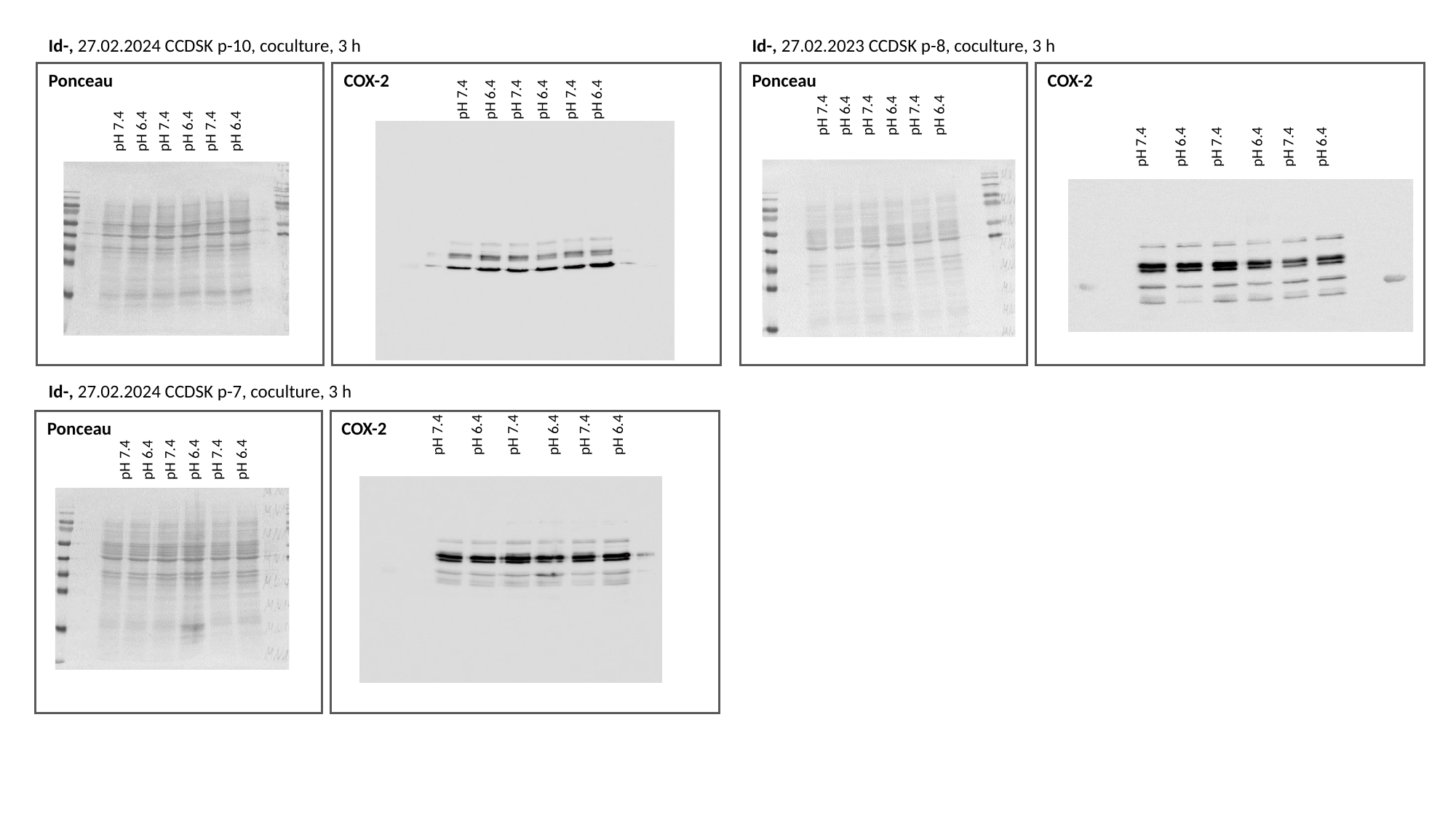

Id-, 27.02.2024 CCDSK p-10, coculture, 3 h
Id-, 27.02.2023 CCDSK p-8, coculture, 3 h
COX-2
COX-2
Ponceau
Ponceau
pH 6.4
pH 6.4
pH 6.4
pH 7.4
pH 7.4
pH 7.4
pH 6.4
pH 6.4
pH 6.4
pH 7.4
pH 7.4
pH 7.4
pH 6.4
pH 6.4
pH 6.4
pH 7.4
pH 7.4
pH 7.4
pH 6.4
pH 6.4
pH 6.4
pH 7.4
pH 7.4
pH 7.4
Id-, 27.02.2024 CCDSK p-7, coculture, 3 h
pH 6.4
pH 6.4
pH 6.4
Ponceau
COX-2
pH 7.4
pH 7.4
pH 7.4
pH 6.4
pH 6.4
pH 6.4
pH 7.4
pH 7.4
pH 7.4

## Slide 74
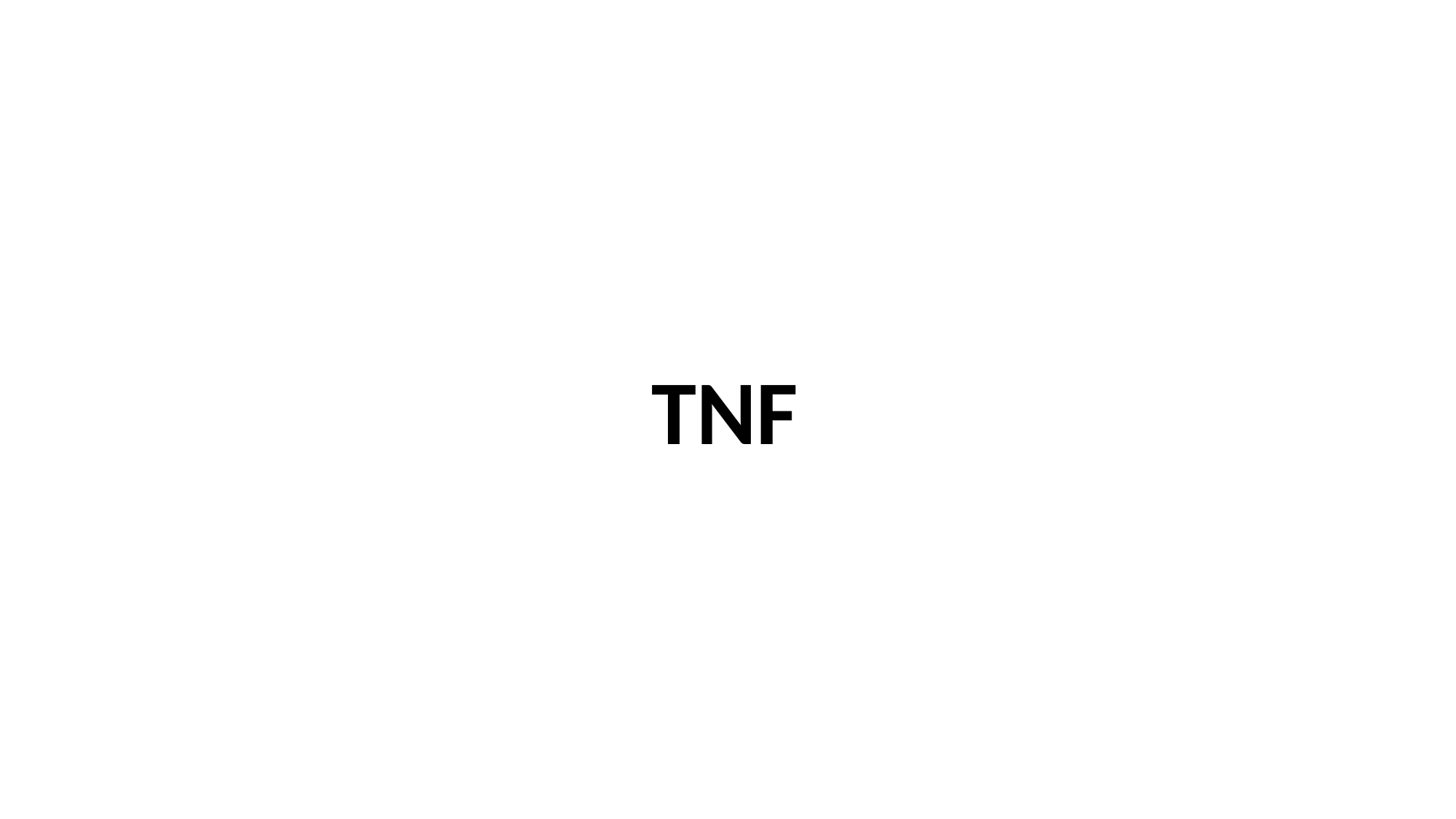

TNF

## Slide 75
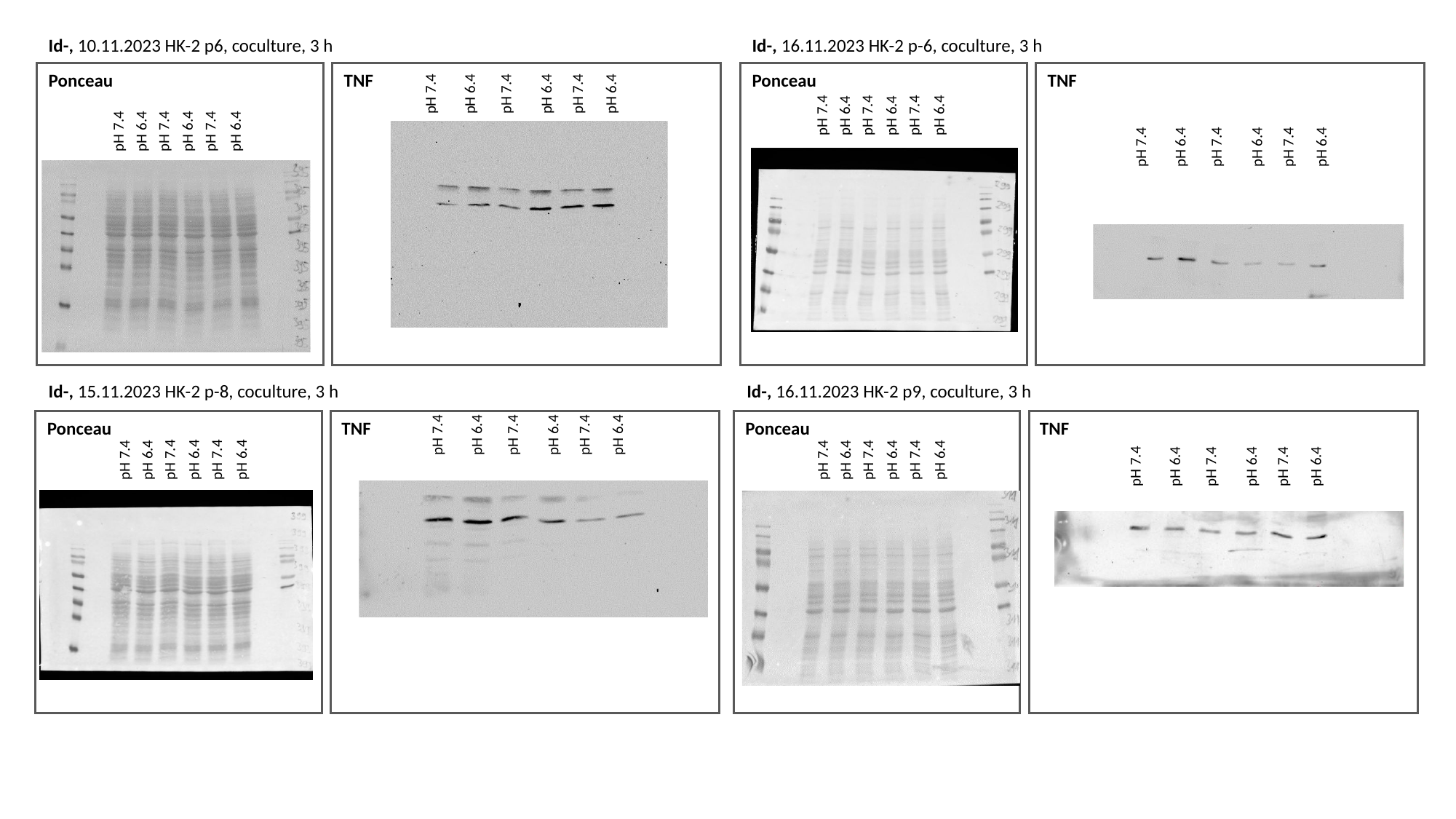

Id-, 10.11.2023 HK-2 p6, coculture, 3 h
Id-, 16.11.2023 HK-2 p-6, coculture, 3 h
TNF
TNF
Ponceau
Ponceau
pH 6.4
pH 6.4
pH 6.4
pH 7.4
pH 7.4
pH 7.4
pH 6.4
pH 6.4
pH 6.4
pH 7.4
pH 7.4
pH 7.4
pH 6.4
pH 6.4
pH 6.4
pH 7.4
pH 7.4
pH 7.4
pH 6.4
pH 6.4
pH 6.4
pH 7.4
pH 7.4
pH 7.4
Id-, 15.11.2023 HK-2 p-8, coculture, 3 h
Id-, 16.11.2023 HK-2 p9, coculture, 3 h
pH 6.4
pH 6.4
pH 6.4
Ponceau
Ponceau
TNF
TNF
pH 7.4
pH 7.4
pH 7.4
pH 6.4
pH 6.4
pH 6.4
pH 6.4
pH 6.4
pH 6.4
pH 6.4
pH 6.4
pH 6.4
pH 7.4
pH 7.4
pH 7.4
pH 7.4
pH 7.4
pH 7.4
pH 7.4
pH 7.4
pH 7.4

## Slide 76
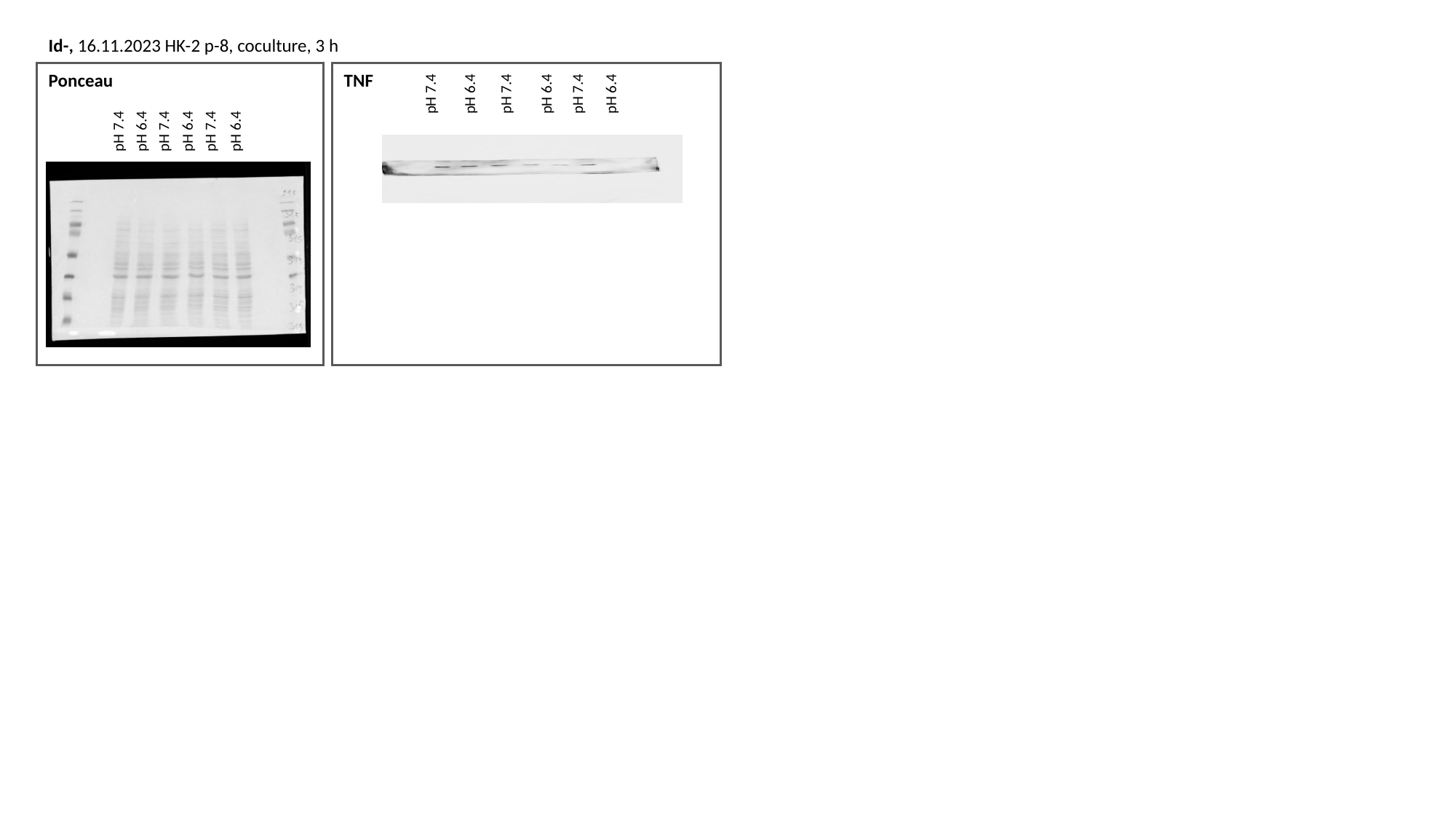

Id-, 16.11.2023 HK-2 p-8, coculture, 3 h
TNF
Ponceau
pH 6.4
pH 6.4
pH 6.4
pH 7.4
pH 7.4
pH 7.4
pH 6.4
pH 6.4
pH 6.4
pH 7.4
pH 7.4
pH 7.4

## Slide 77
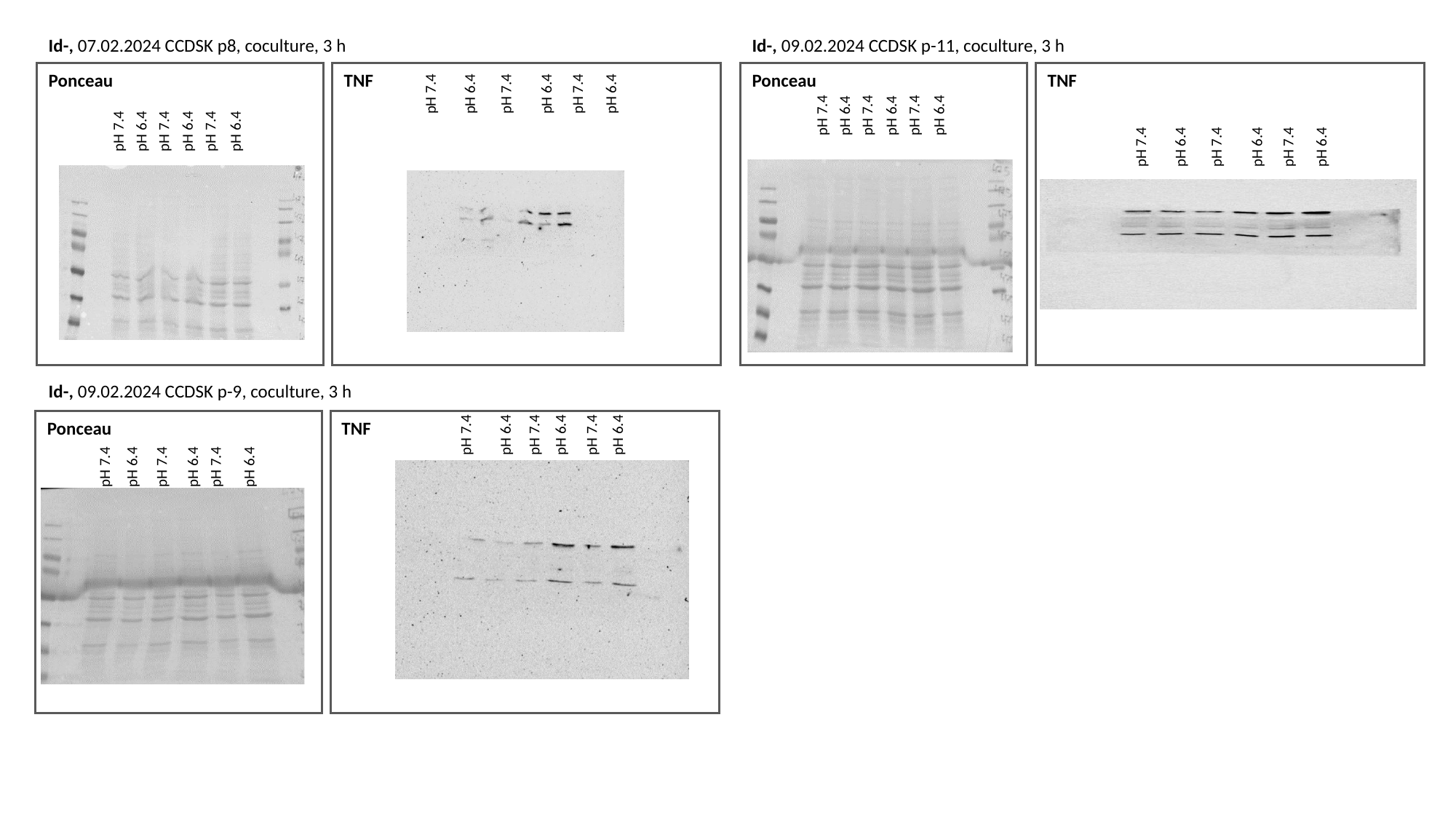

Id-, 07.02.2024 CCDSK p8, coculture, 3 h
Id-, 09.02.2024 CCDSK p-11, coculture, 3 h
TNF
TNF
Ponceau
Ponceau
pH 6.4
pH 6.4
pH 6.4
pH 7.4
pH 7.4
pH 7.4
pH 6.4
pH 6.4
pH 6.4
pH 7.4
pH 7.4
pH 7.4
pH 6.4
pH 6.4
pH 6.4
pH 7.4
pH 7.4
pH 7.4
pH 6.4
pH 6.4
pH 6.4
pH 7.4
pH 7.4
pH 7.4
Id-, 09.02.2024 CCDSK p-9, coculture, 3 h
pH 6.4
pH 6.4
pH 6.4
Ponceau
TNF
pH 7.4
pH 7.4
pH 7.4
pH 6.4
pH 6.4
pH 6.4
pH 7.4
pH 7.4
pH 7.4

## Slide 78
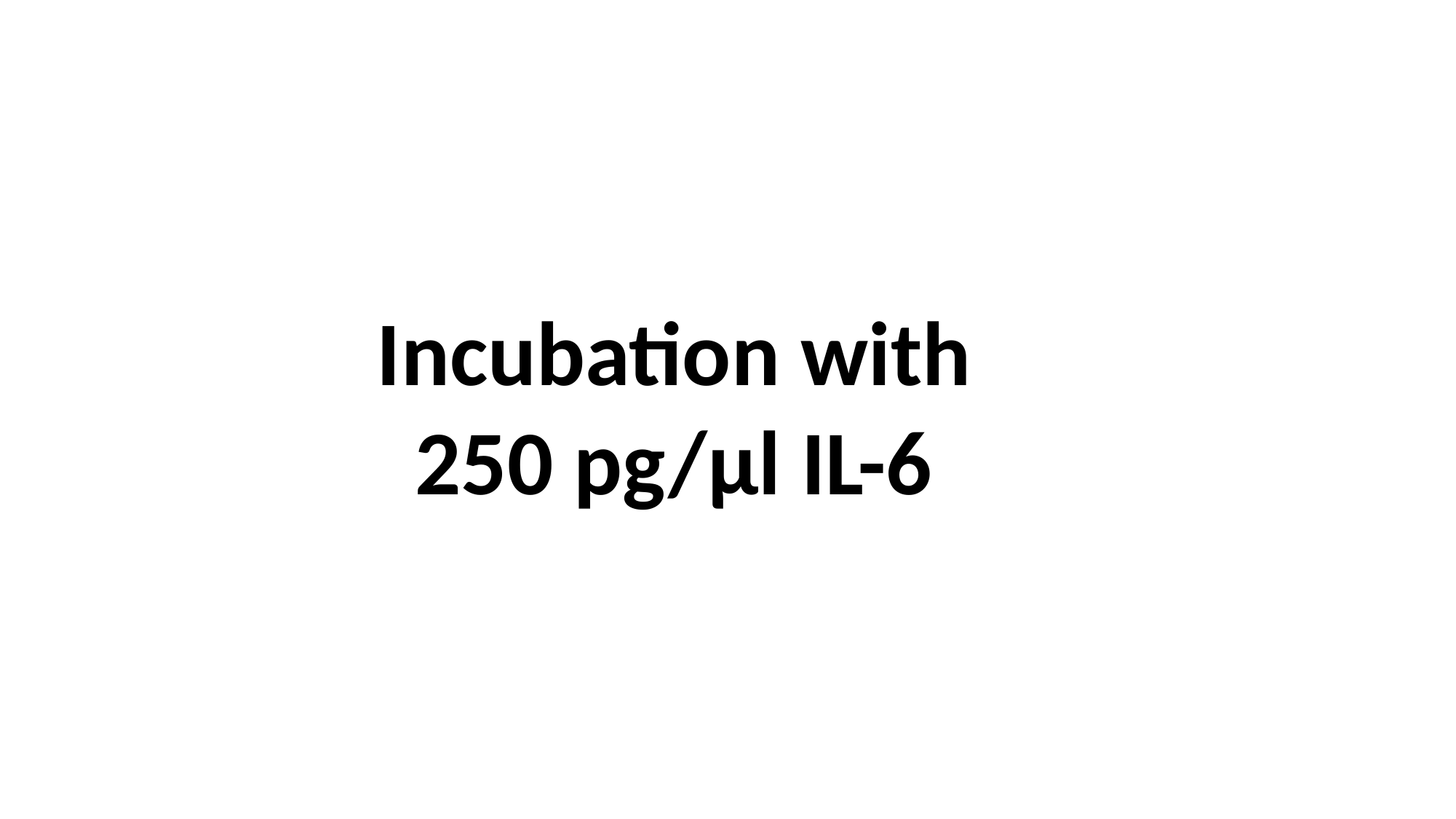

Incubation with 250 pg/µl IL-6

## Slide 79
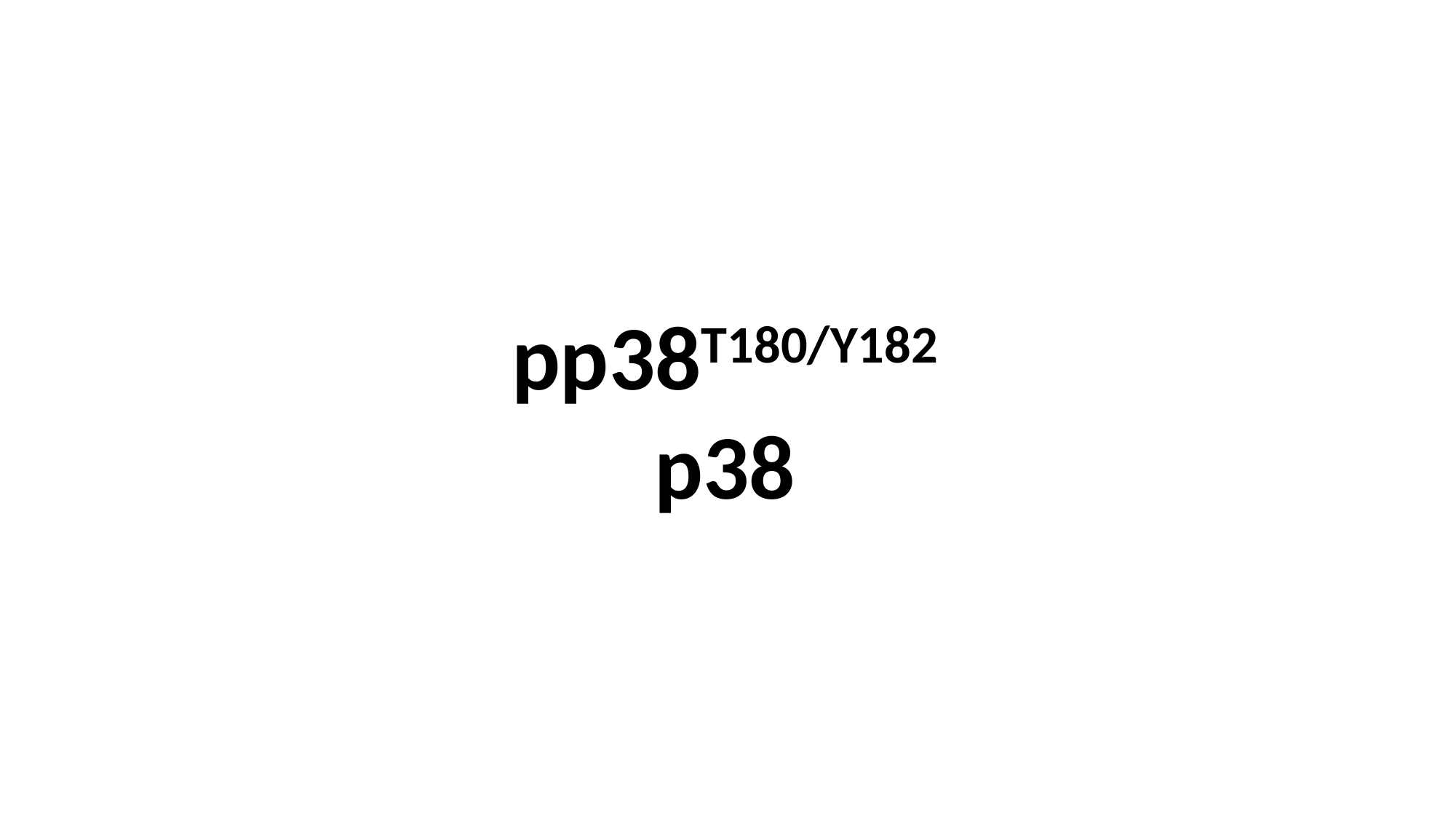

pp38T180/Y182
p38

## Slide 80
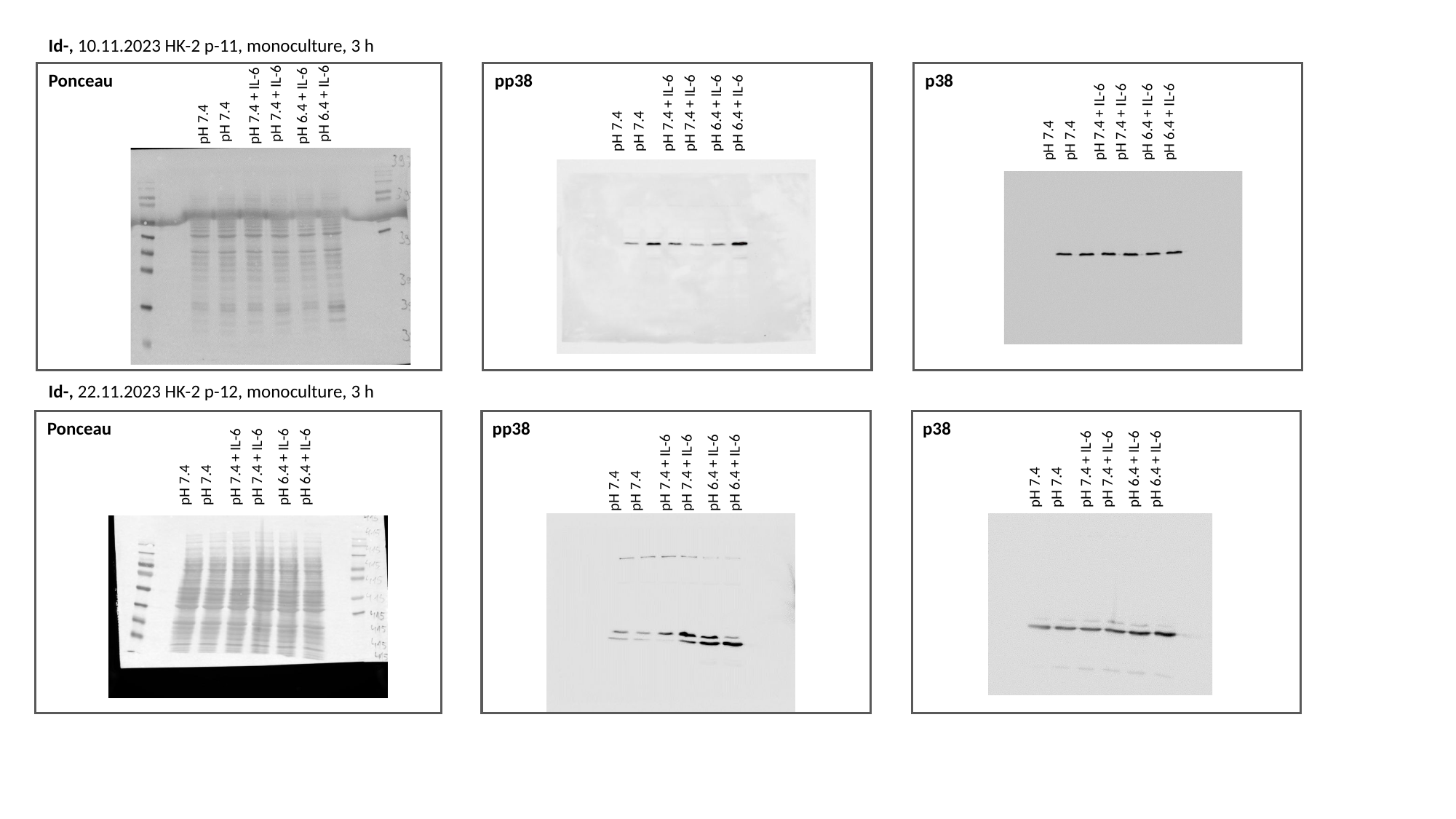

Id-, 10.11.2023 HK-2 p-11, monoculture, 3 h
pp38
p38
Ponceau
pH 6.4 + IL-6
pH 6.4 + IL-6
pH 7.4 + IL-6
pH 7.4 + IL-6
pH 6.4 + IL-6
pH 6.4 + IL-6
pH 7.4 + IL-6
pH 7.4 + IL-6
pH 6.4 + IL-6
pH 6.4 + IL-6
pH 7.4
pH 7.4
pH 7.4 + IL-6
pH 7.4 + IL-6
pH 7.4
pH 7.4
pH 7.4
pH 7.4
Id-, 22.11.2023 HK-2 p-12, monoculture, 3 h
Ponceau
pp38
p38
pH 6.4 + IL-6
pH 6.4 + IL-6
pH 6.4 + IL-6
pH 6.4 + IL-6
pH 7.4 + IL-6
pH 7.4 + IL-6
pH 6.4 + IL-6
pH 6.4 + IL-6
pH 7.4 + IL-6
pH 7.4 + IL-6
pH 7.4 + IL-6
pH 7.4 + IL-6
pH 7.4
pH 7.4
pH 7.4
pH 7.4
pH 7.4
pH 7.4

## Slide 81
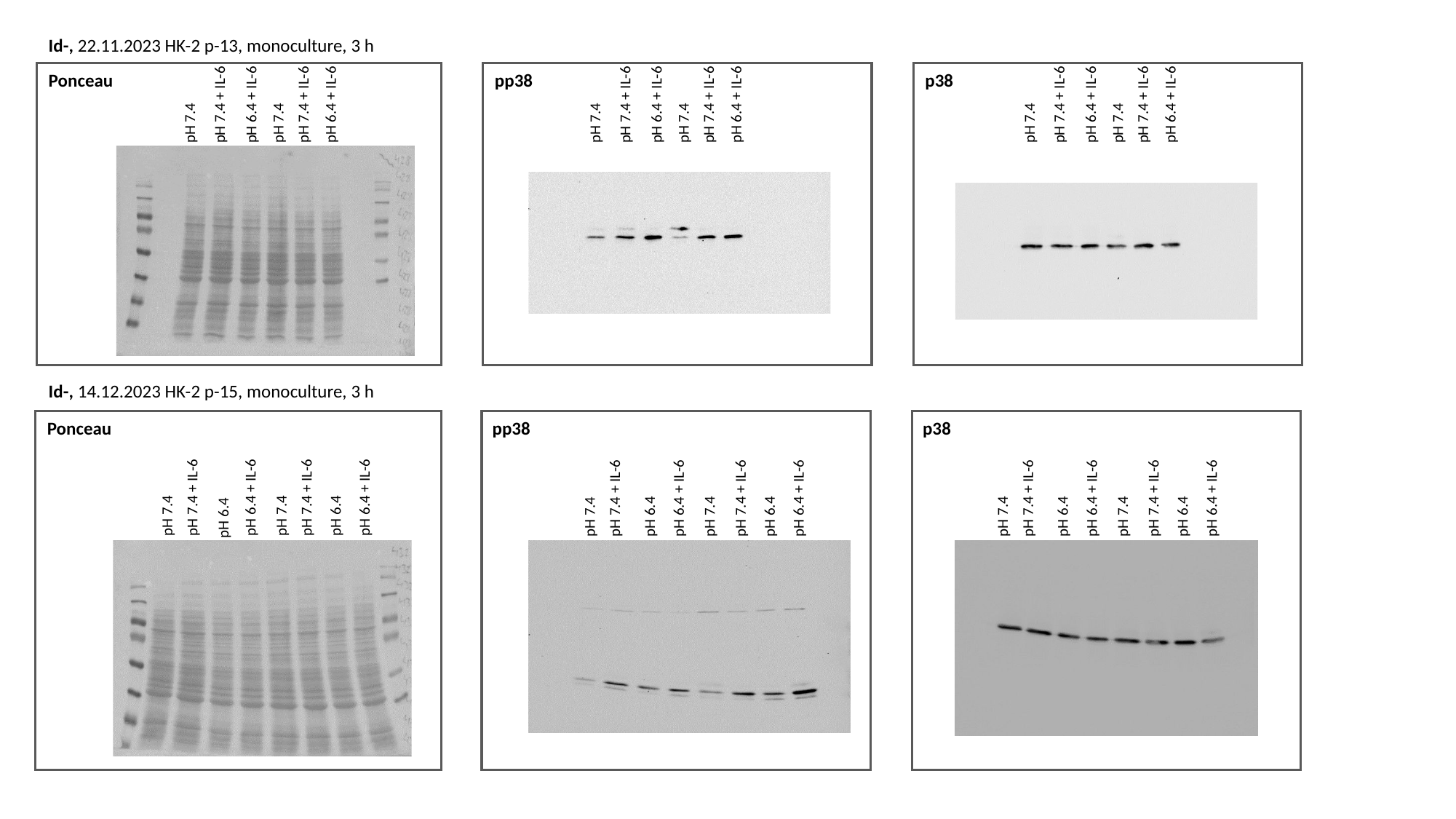

Id-, 22.11.2023 HK-2 p-13, monoculture, 3 h
pp38
p38
Ponceau
pH 6.4 + IL-6
pH 6.4 + IL-6
pH 6.4 + IL-6
pH 6.4 + IL-6
pH 6.4 + IL-6
pH 6.4 + IL-6
pH 7.4 + IL-6
pH 7.4 + IL-6
pH 7.4 + IL-6
pH 7.4 + IL-6
pH 7.4 + IL-6
pH 7.4 + IL-6
pH 7.4
pH 7.4
pH 7.4
pH 7.4
pH 7.4
pH 7.4
Id-, 14.12.2023 HK-2 p-15, monoculture, 3 h
Ponceau
pp38
p38
pH 6.4 + IL-6
pH 6.4
pH 6.4 + IL-6
pH 6.4
pH 6.4 + IL-6
pH 6.4
pH 6.4 + IL-6
pH 6.4
pH 6.4 + IL-6
pH 6.4
pH 6.4 + IL-6
pH 6.4
pH 7.4 + IL-6
pH 7.4 + IL-6
pH 7.4 + IL-6
pH 7.4 + IL-6
pH 7.4 + IL-6
pH 7.4 + IL-6
pH 7.4
pH 7.4
pH 7.4
pH 7.4
pH 7.4
pH 7.4

## Slide 82
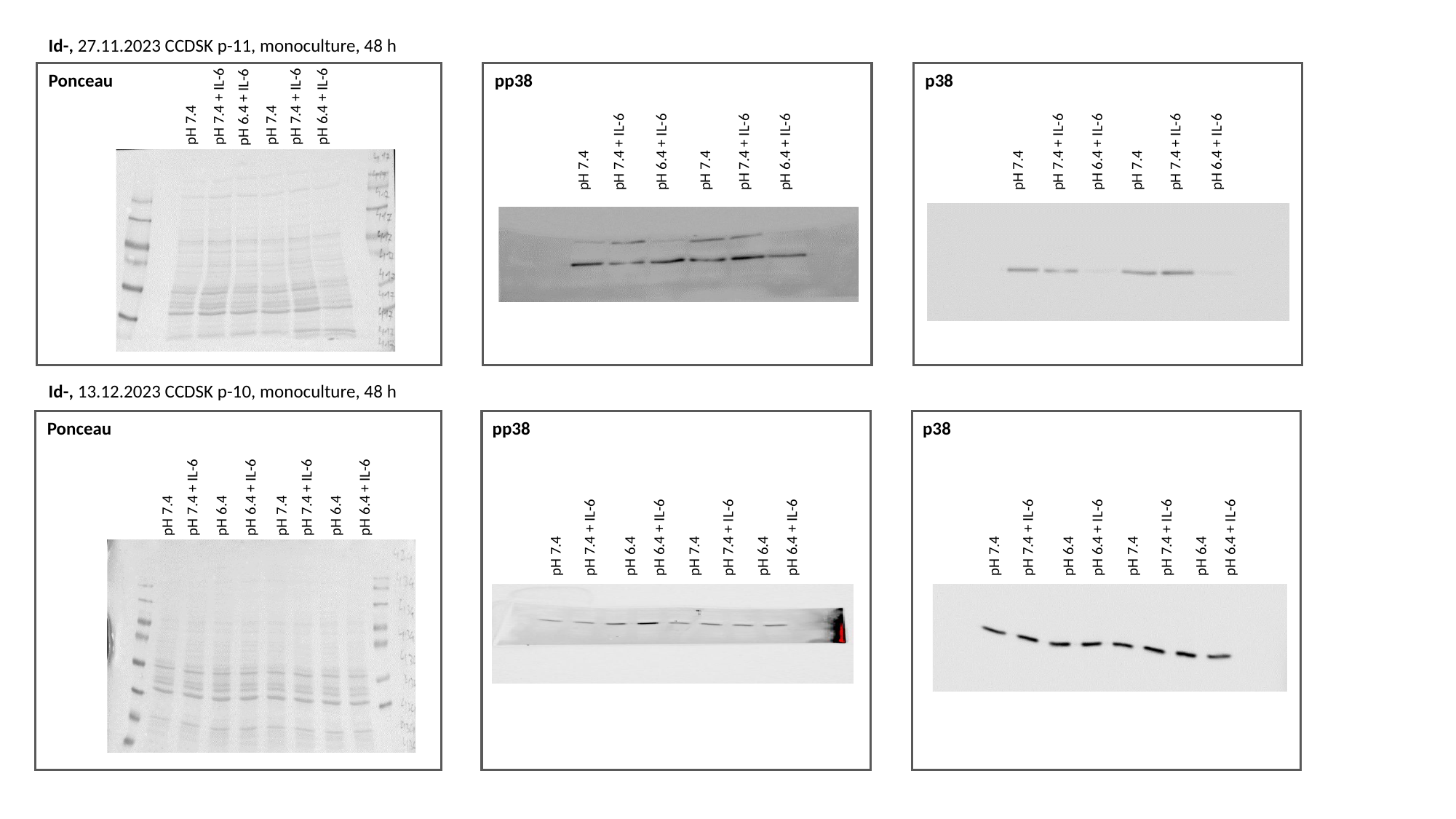

Id-, 27.11.2023 CCDSK p-11, monoculture, 48 h
pp38
p38
Ponceau
pH 6.4 + IL-6
pH 6.4 + IL-6
pH 7.4 + IL-6
pH 7.4 + IL-6
pH 7.4
pH 7.4
pH 6.4 + IL-6
pH 6.4 + IL-6
pH 6.4 + IL-6
pH 6.4 + IL-6
pH 7.4 + IL-6
pH 7.4 + IL-6
pH 7.4 + IL-6
pH 7.4 + IL-6
pH 7.4
pH 7.4
pH 7.4
pH 7.4
Id-, 13.12.2023 CCDSK p-10, monoculture, 48 h
Ponceau
pp38
p38
pH 6.4
pH 6.4 + IL-6
pH 6.4
pH 6.4 + IL-6
pH 7.4 + IL-6
pH 7.4 + IL-6
pH 7.4
pH 7.4
pH 6.4
pH 6.4 + IL-6
pH 6.4
pH 6.4 + IL-6
pH 6.4
pH 6.4 + IL-6
pH 6.4
pH 6.4 + IL-6
pH 7.4 + IL-6
pH 7.4 + IL-6
pH 7.4 + IL-6
pH 7.4 + IL-6
pH 7.4
pH 7.4
pH 7.4
pH 7.4

## Slide 83
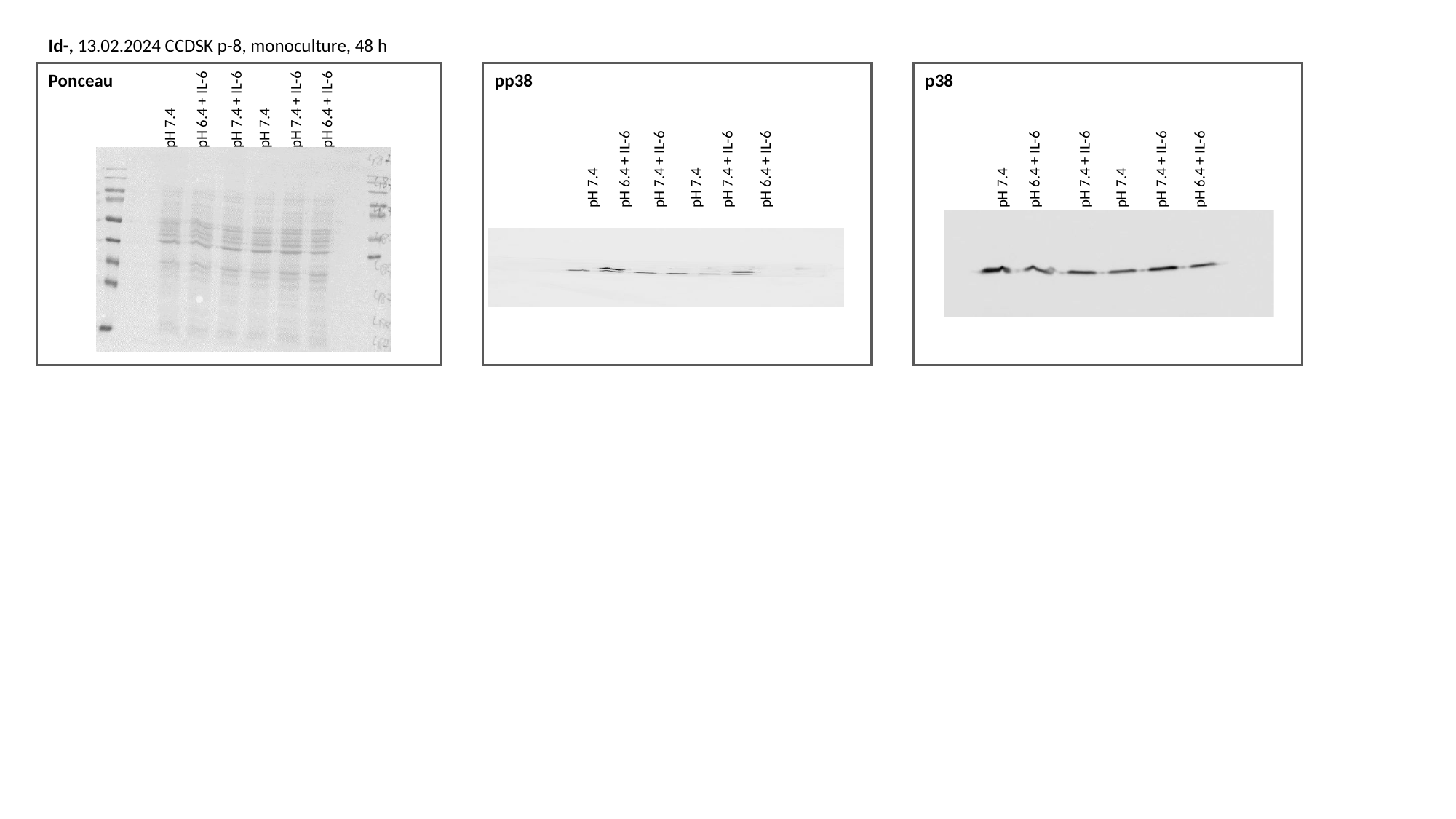

Id-, 13.02.2024 CCDSK p-8, monoculture, 48 h
pp38
p38
Ponceau
pH 6.4 + IL-6
pH 6.4 + IL-6
pH 7.4 + IL-6
pH 7.4 + IL-6
pH 7.4
pH 7.4
pH 6.4 + IL-6
pH 6.4 + IL-6
pH 6.4 + IL-6
pH 6.4 + IL-6
pH 7.4 + IL-6
pH 7.4 + IL-6
pH 7.4 + IL-6
pH 7.4 + IL-6
pH 7.4
pH 7.4
pH 7.4
pH 7.4

## Slide 84
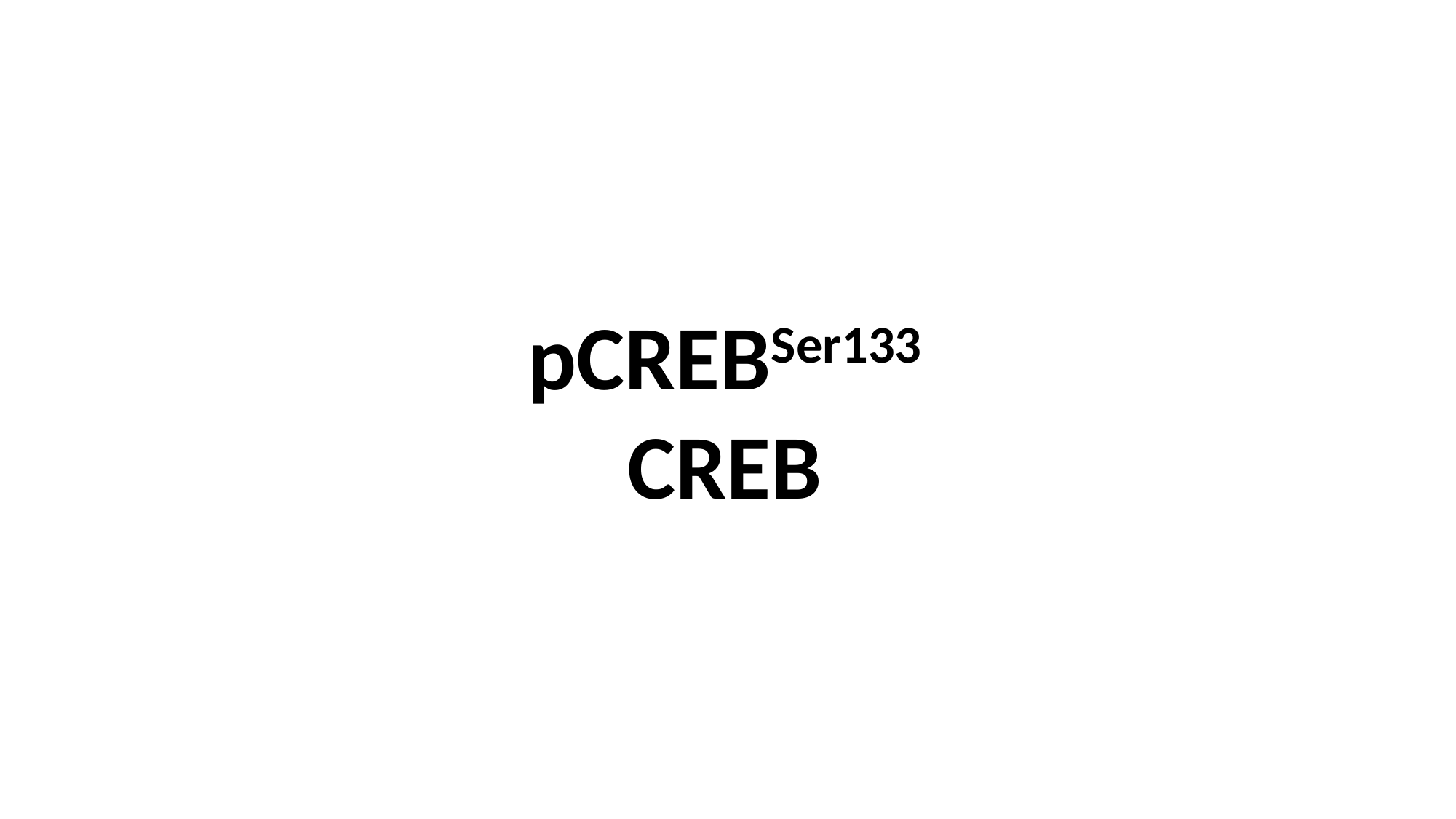

pCREBSer133
CREB

## Slide 85
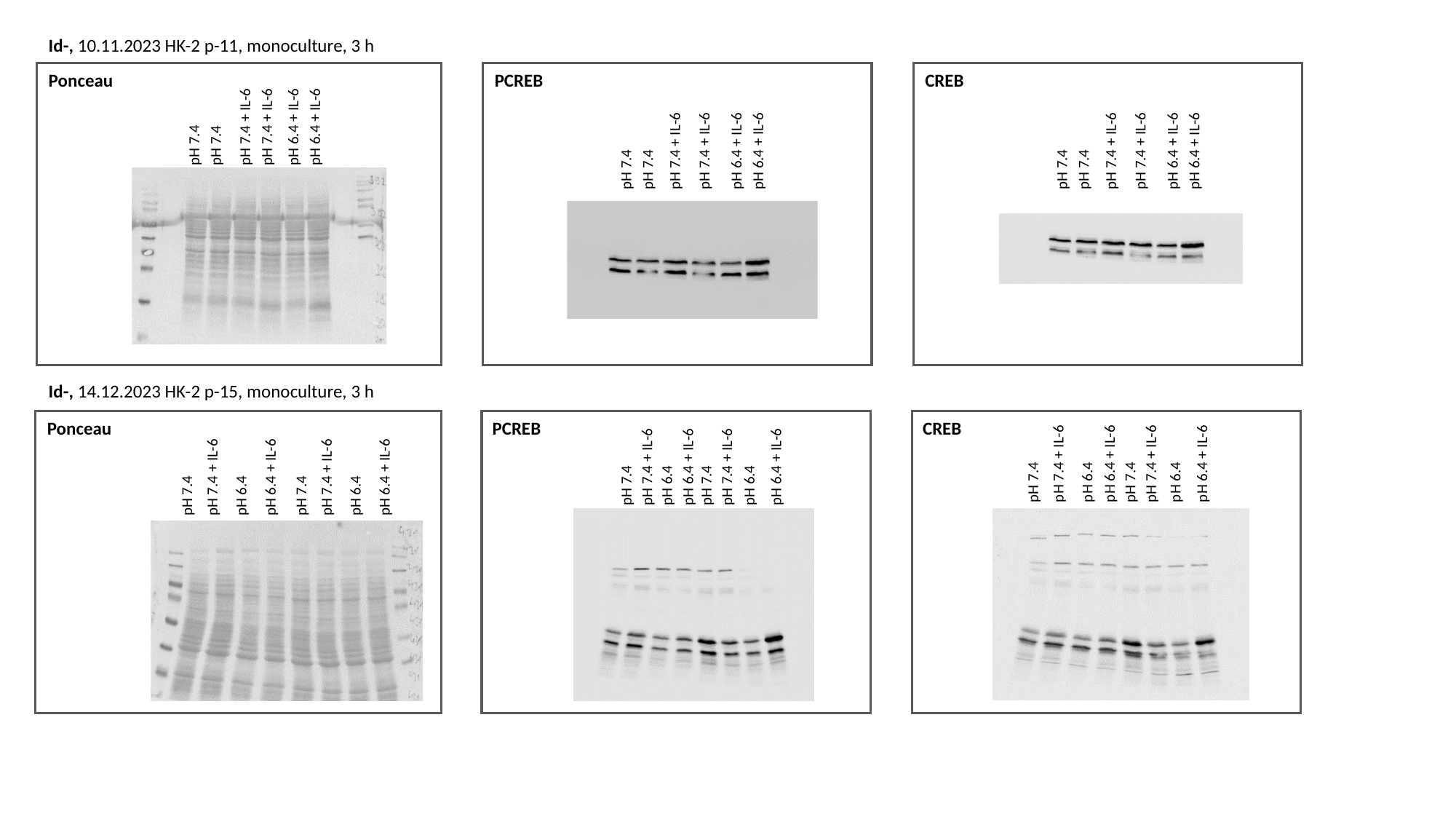

Id-, 10.11.2023 HK-2 p-11, monoculture, 3 h
PCREB
CREB
Ponceau
pH 6.4 + IL-6
pH 6.4 + IL-6
pH 7.4 + IL-6
pH 7.4 + IL-6
pH 7.4
pH 7.4
pH 6.4 + IL-6
pH 6.4 + IL-6
pH 6.4 + IL-6
pH 6.4 + IL-6
pH 7.4 + IL-6
pH 7.4 + IL-6
pH 7.4 + IL-6
pH 7.4 + IL-6
pH 7.4
pH 7.4
pH 7.4
pH 7.4
Id-, 14.12.2023 HK-2 p-15, monoculture, 3 h
Ponceau
PCREB
CREB
pH 6.4
pH 6.4 + IL-6
pH 6.4
pH 6.4 + IL-6
pH 6.4
pH 6.4 + IL-6
pH 6.4
pH 6.4 + IL-6
pH 7.4 + IL-6
pH 7.4 + IL-6
pH 7.4 + IL-6
pH 7.4 + IL-6
pH 6.4
pH 6.4 + IL-6
pH 6.4
pH 6.4 + IL-6
pH 7.4 + IL-6
pH 7.4 + IL-6
pH 7.4
pH 7.4
pH 7.4
pH 7.4
pH 7.4
pH 7.4

## Slide 86
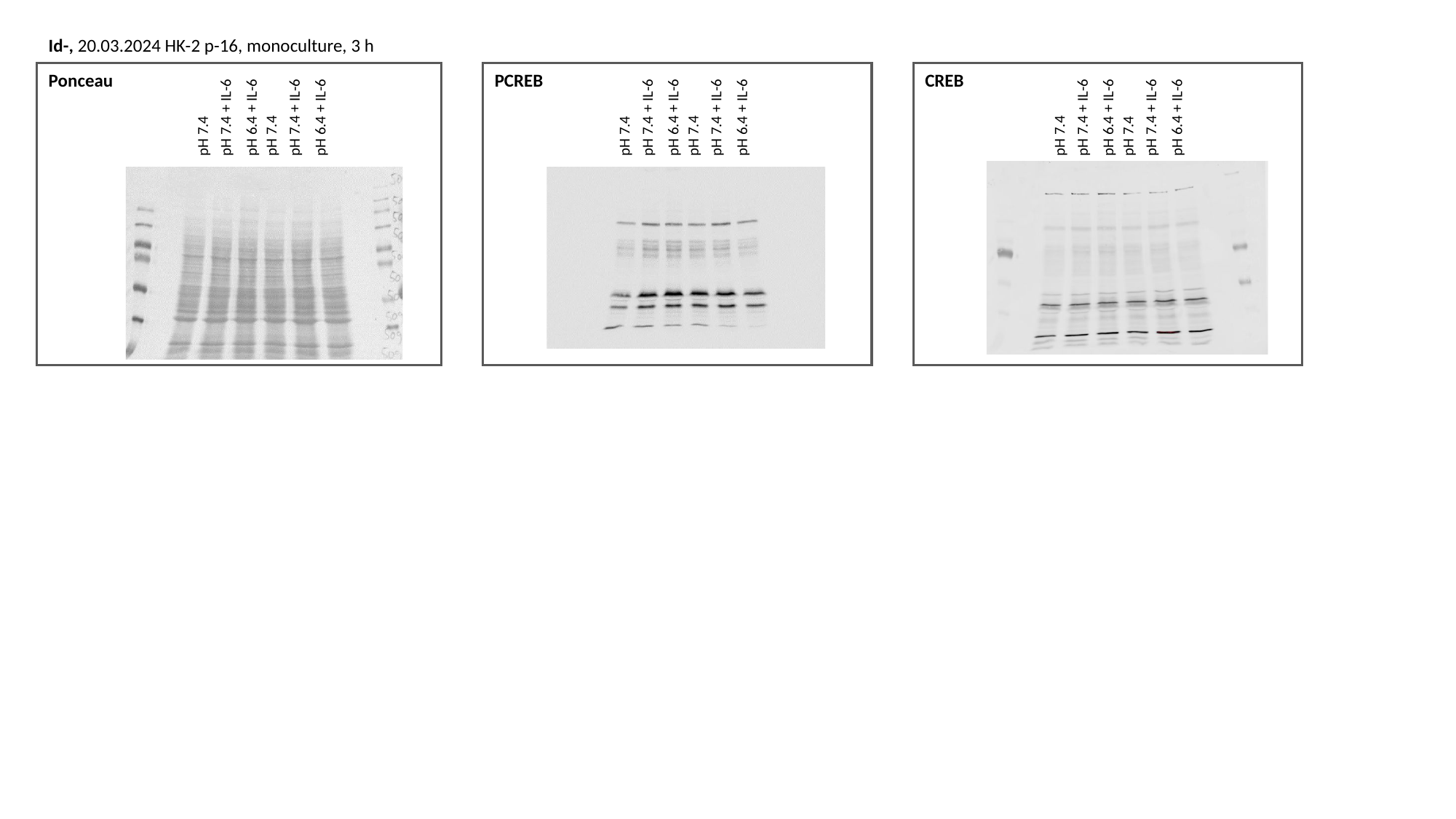

Id-, 20.03.2024 HK-2 p-16, monoculture, 3 h
PCREB
CREB
Ponceau
pH 6.4 + IL-6
pH 6.4 + IL-6
pH 6.4 + IL-6
pH 6.4 + IL-6
pH 6.4 + IL-6
pH 6.4 + IL-6
pH 7.4 + IL-6
pH 7.4 + IL-6
pH 7.4 + IL-6
pH 7.4 + IL-6
pH 7.4 + IL-6
pH 7.4 + IL-6
pH 7.4
pH 7.4
pH 7.4
pH 7.4
pH 7.4
pH 7.4

## Slide 87
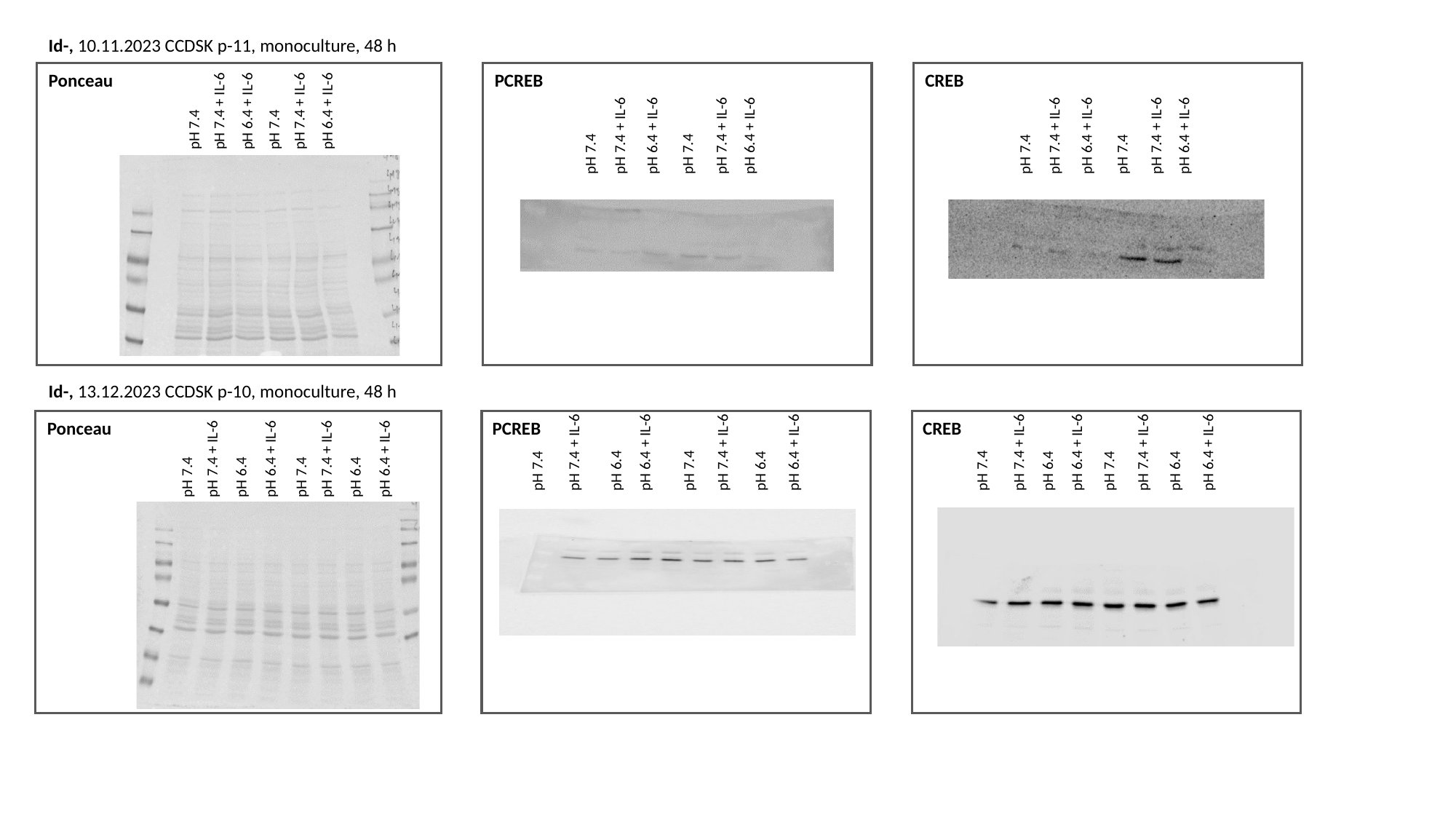

Id-, 10.11.2023 CCDSK p-11, monoculture, 48 h
PCREB
CREB
Ponceau
pH 6.4 + IL-6
pH 6.4 + IL-6
pH 7.4 + IL-6
pH 7.4 + IL-6
pH 7.4
pH 7.4
pH 6.4 + IL-6
pH 6.4 + IL-6
pH 6.4 + IL-6
pH 6.4 + IL-6
pH 7.4 + IL-6
pH 7.4 + IL-6
pH 7.4 + IL-6
pH 7.4 + IL-6
pH 7.4
pH 7.4
pH 7.4
pH 7.4
Id-, 13.12.2023 CCDSK p-10, monoculture, 48 h
Ponceau
PCREB
CREB
pH 6.4
pH 6.4 + IL-6
pH 6.4
pH 6.4 + IL-6
pH 6.4
pH 6.4 + IL-6
pH 6.4
pH 6.4 + IL-6
pH 7.4 + IL-6
pH 7.4 + IL-6
pH 7.4 + IL-6
pH 7.4 + IL-6
pH 6.4
pH 6.4 + IL-6
pH 6.4
pH 6.4 + IL-6
pH 7.4 + IL-6
pH 7.4 + IL-6
pH 7.4
pH 7.4
pH 7.4
pH 7.4
pH 7.4
pH 7.4

## Slide 88
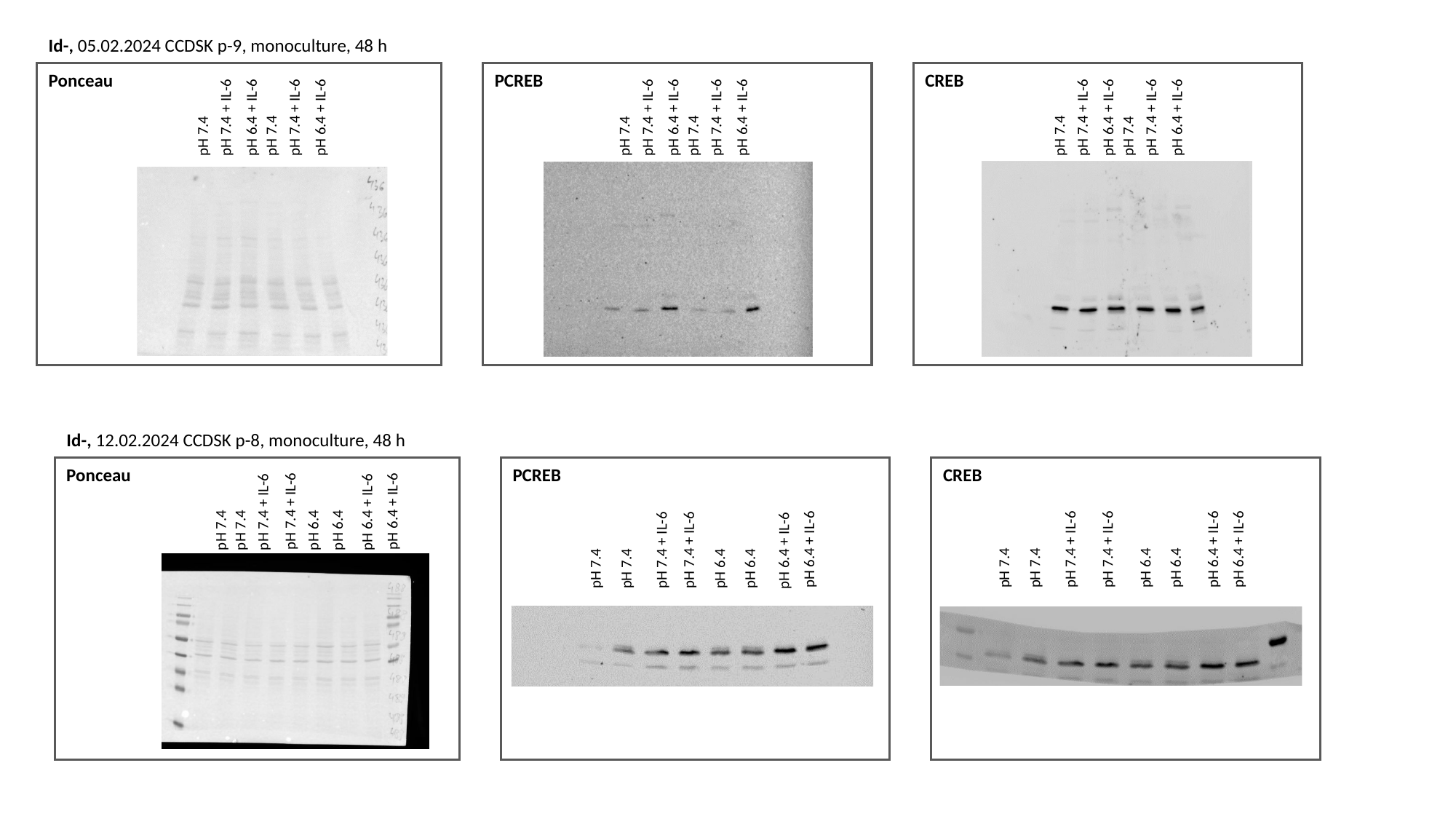

Id-, 05.02.2024 CCDSK p-9, monoculture, 48 h
PCREB
CREB
Ponceau
pH 6.4 + IL-6
pH 6.4 + IL-6
pH 6.4 + IL-6
pH 6.4 + IL-6
pH 6.4 + IL-6
pH 6.4 + IL-6
pH 7.4 + IL-6
pH 7.4 + IL-6
pH 7.4 + IL-6
pH 7.4 + IL-6
pH 7.4 + IL-6
pH 7.4 + IL-6
pH 7.4
pH 7.4
pH 7.4
pH 7.4
pH 7.4
pH 7.4
Id-, 12.02.2024 CCDSK p-8, monoculture, 48 h
PCREB
CREB
Ponceau
pH 6.4 + IL-6
pH 7.4 + IL-6
pH 7.4 + IL-6
pH 6.4 + IL-6
pH 6.4
pH 6.4
pH 7.4
pH 7.4
pH 6.4 + IL-6
pH 6.4 + IL-6
pH 7.4 + IL-6
pH 7.4 + IL-6
pH 6.4 + IL-6
pH 7.4 + IL-6
pH 7.4 + IL-6
pH 6.4 + IL-6
pH 6.4
pH 6.4
pH 6.4
pH 6.4
pH 7.4
pH 7.4
pH 7.4
pH 7.4

## Slide 89
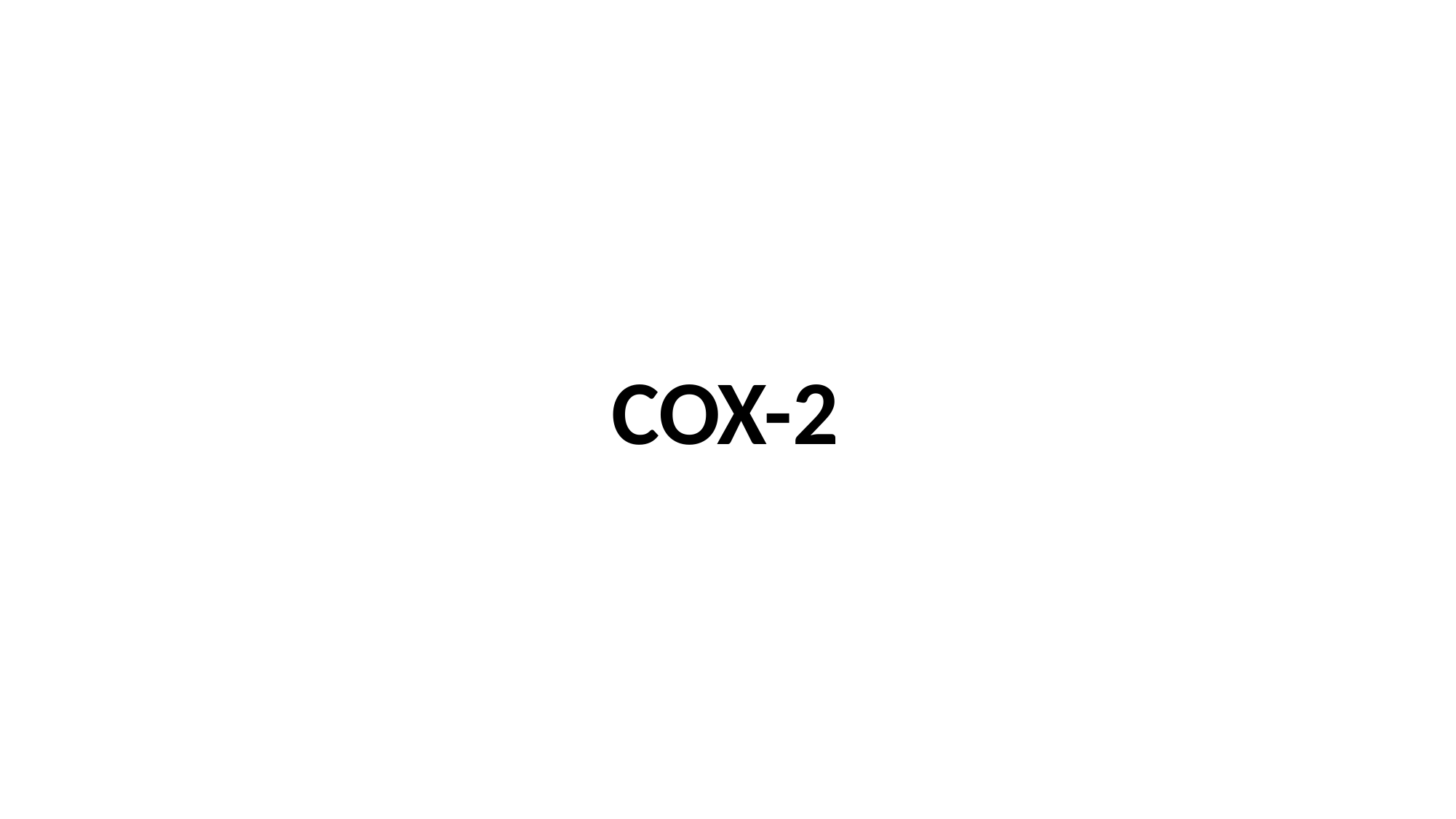

COX-2

## Slide 90
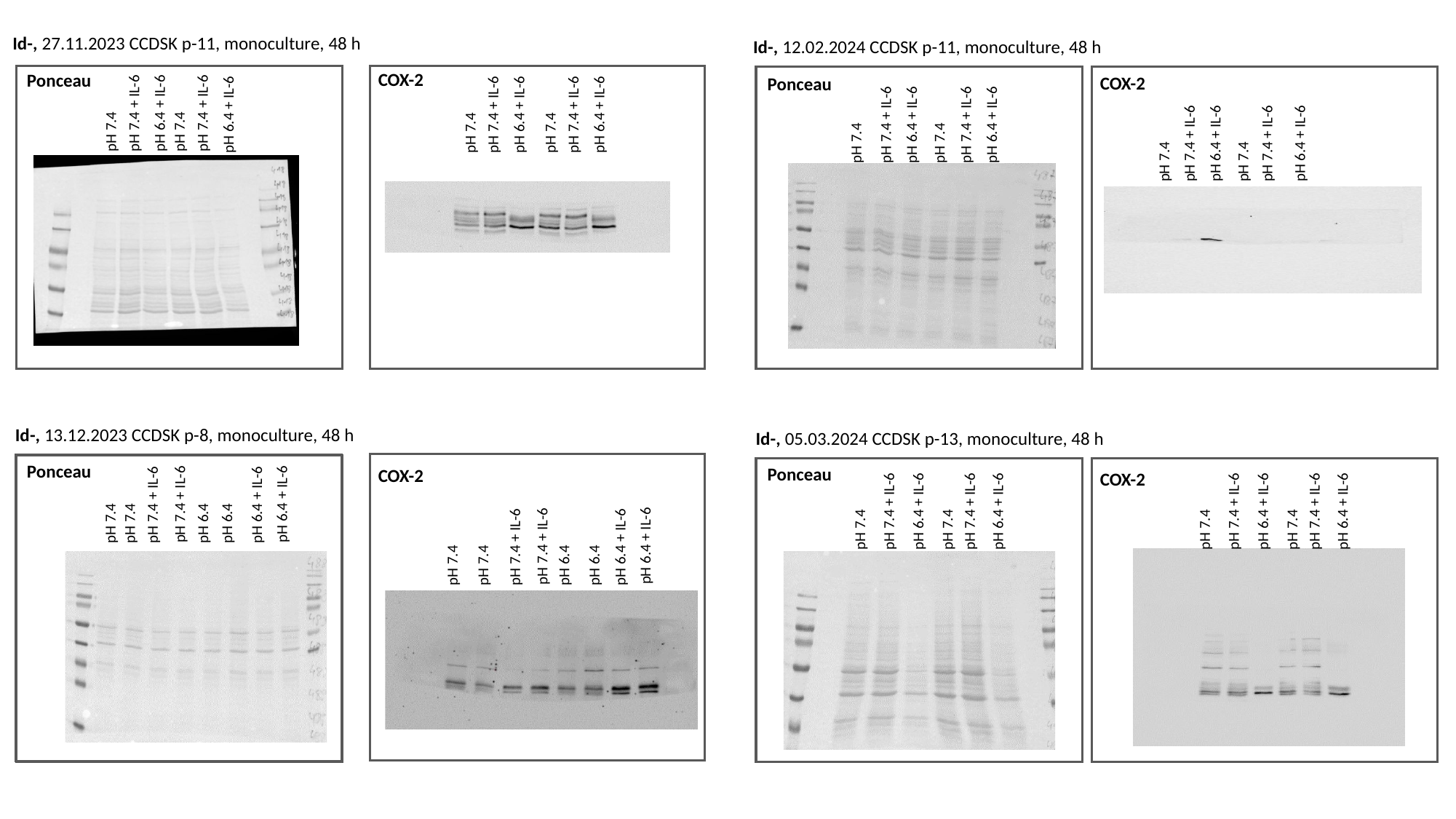

Id-, 27.11.2023 CCDSK p-11, monoculture, 48 h
Id-, 12.02.2024 CCDSK p-11, monoculture, 48 h
COX-2
Ponceau
COX-2
Ponceau
pH 6.4 + IL-6
pH 6.4 + IL-6
pH 6.4 + IL-6
pH 6.4 + IL-6
pH 7.4 + IL-6
pH 7.4 + IL-6
pH 7.4 + IL-6
pH 7.4 + IL-6
pH 6.4 + IL-6
pH 6.4 + IL-6
pH 7.4 + IL-6
pH 7.4 + IL-6
pH 7.4
pH 7.4
pH 7.4
pH 7.4
pH 6.4 + IL-6
pH 6.4 + IL-6
pH 7.4
pH 7.4
pH 7.4 + IL-6
pH 7.4 + IL-6
pH 7.4
pH 7.4
Id-, 13.12.2023 CCDSK p-8, monoculture, 48 h
Id-, 05.03.2024 CCDSK p-13, monoculture, 48 h
Ponceau
Ponceau
COX-2
COX-2
pH 6.4 + IL-6
pH 7.4 + IL-6
pH 7.4 + IL-6
pH 6.4 + IL-6
pH 7.4 + IL-6
pH 7.4 + IL-6
pH 7.4 + IL-6
pH 7.4 + IL-6
pH 6.4 + IL-6
pH 6.4 + IL-6
pH 6.4 + IL-6
pH 6.4 + IL-6
pH 6.4
pH 6.4
pH 7.4
pH 7.4
pH 7.4
pH 7.4
pH 7.4
pH 7.4
pH 6.4 + IL-6
pH 7.4 + IL-6
pH 7.4 + IL-6
pH 6.4 + IL-6
pH 6.4
pH 6.4
pH 7.4
pH 7.4

## Slide 91
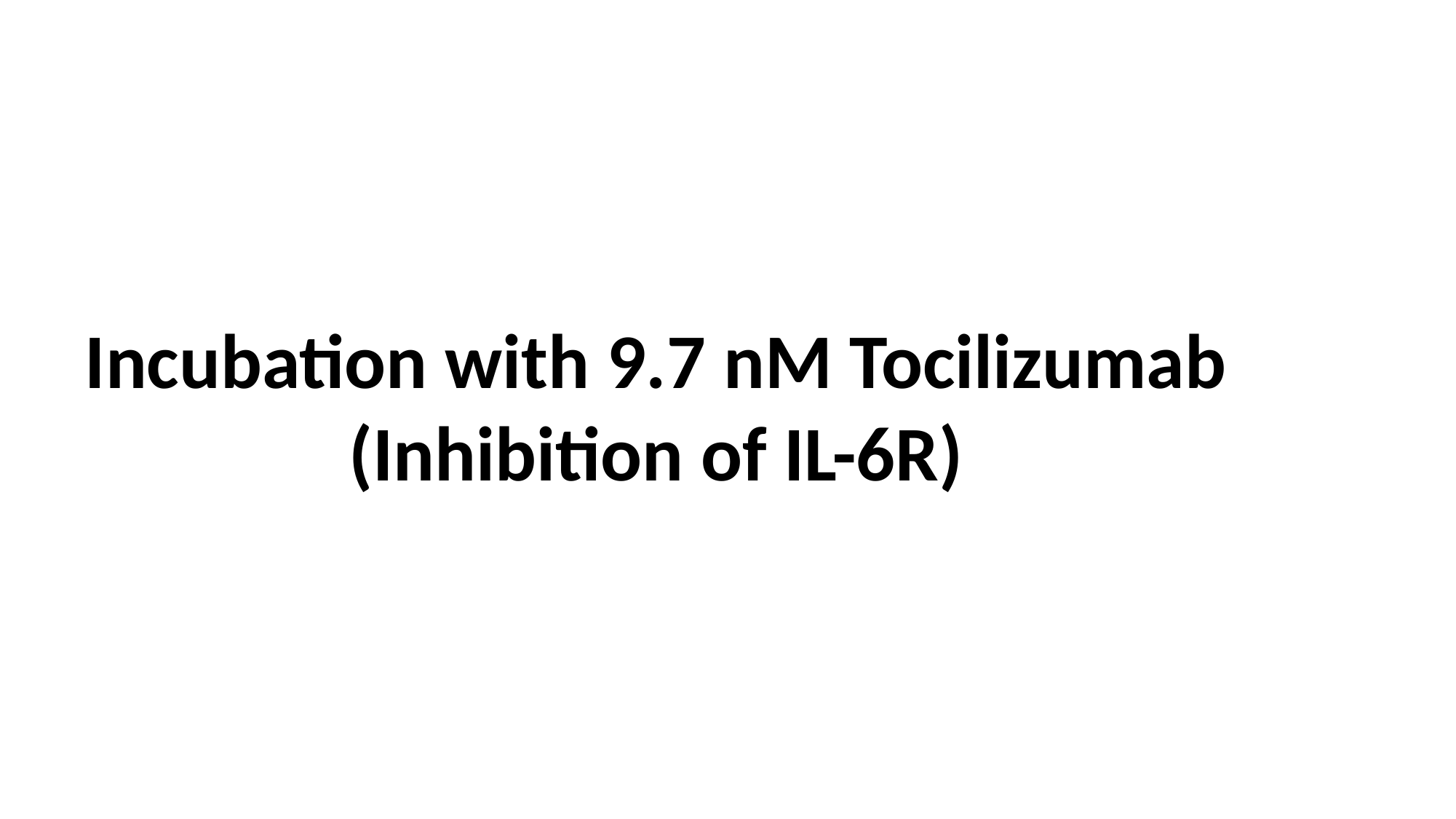

Incubation with 9.7 nM Tocilizumab
(Inhibition of IL-6R)

## Slide 92
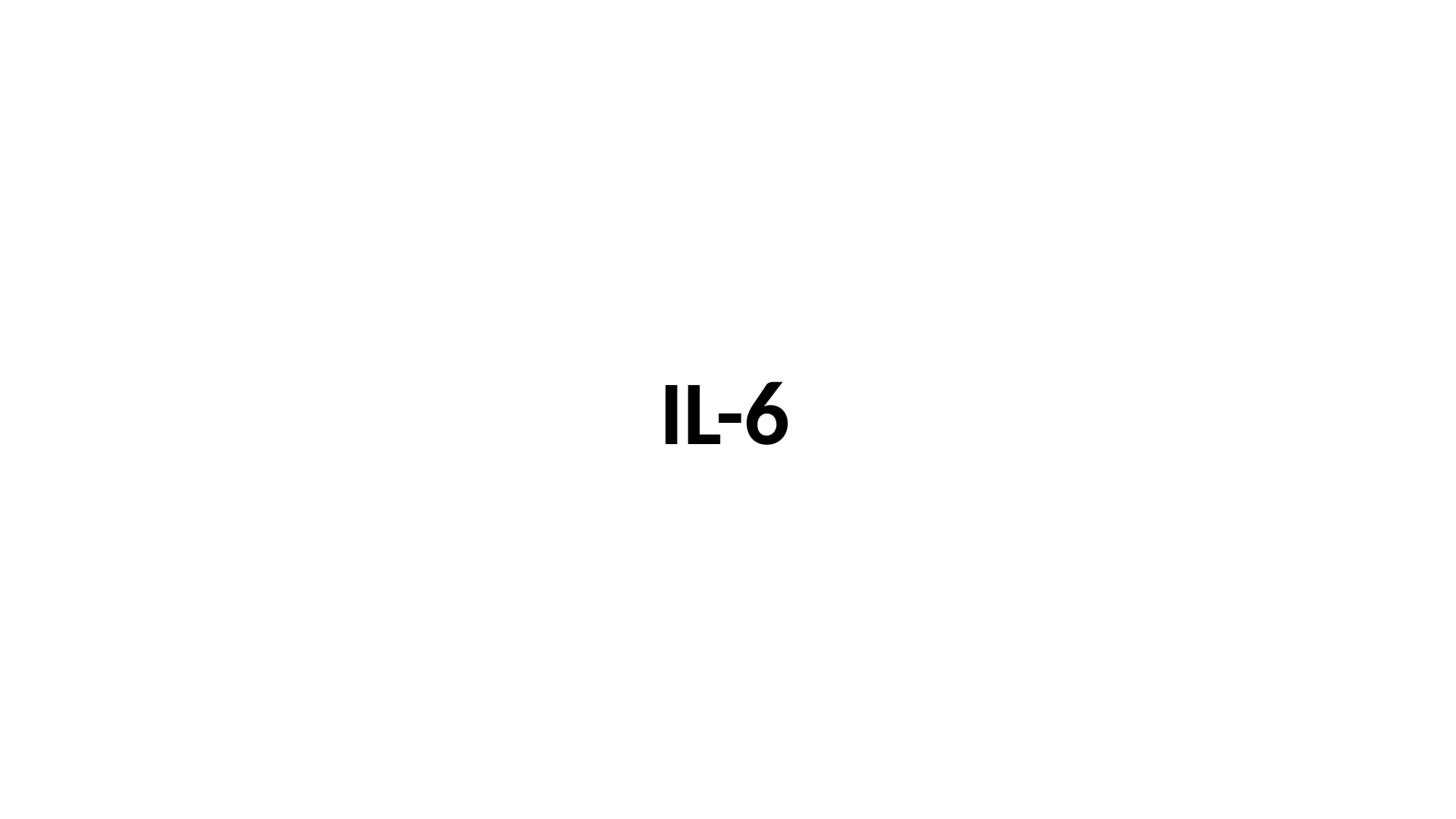

IL-6

## Slide 93
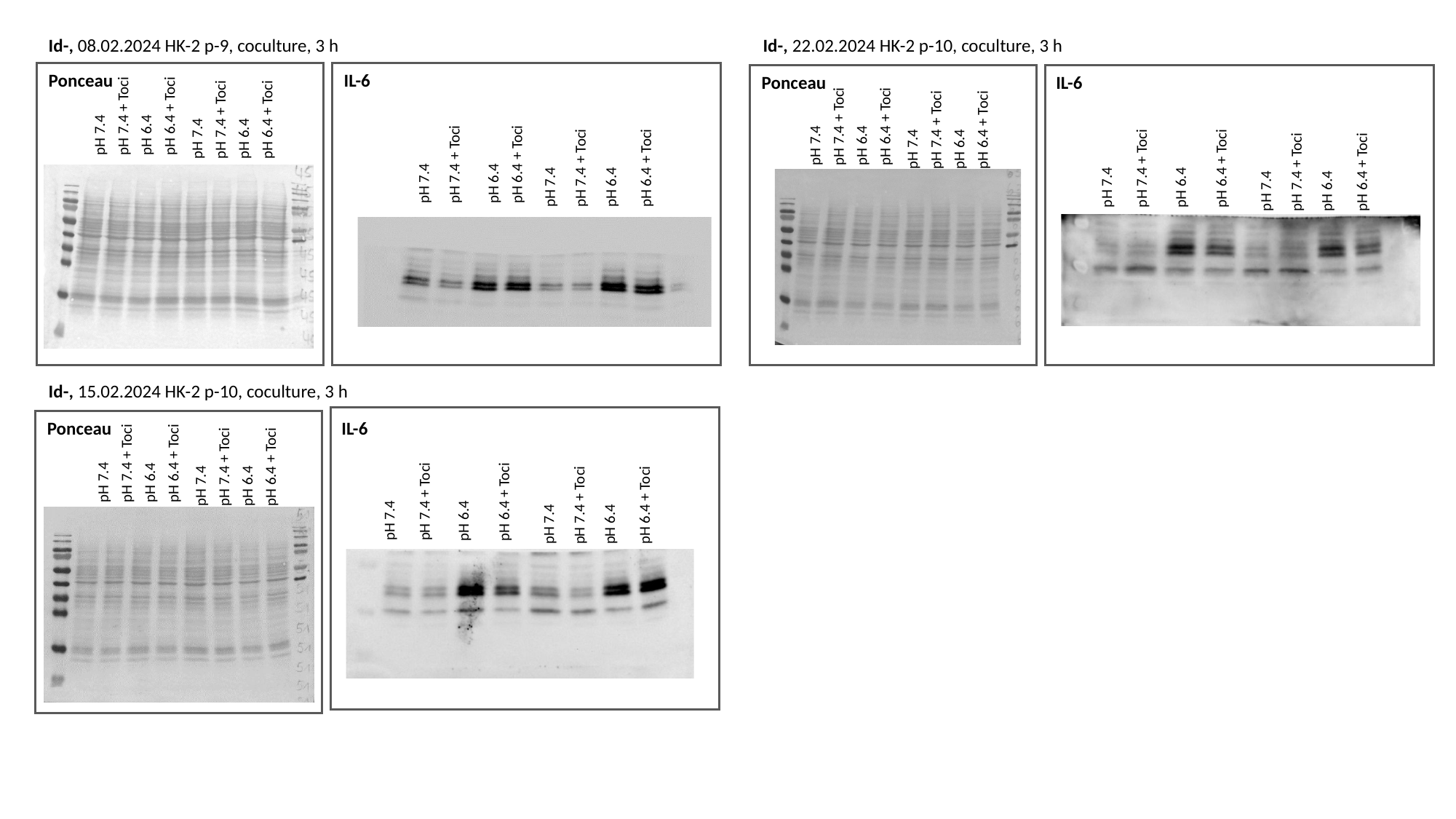

Id-, 08.02.2024 HK-2 p-9, coculture, 3 h
Id-, 22.02.2024 HK-2 p-10, coculture, 3 h
IL-6
Ponceau
Ponceau
IL-6
pH 7.4 + Toci
pH 7.4 + Toci
pH 6.4 + Toci
pH 6.4 + Toci
pH 7.4 + Toci
pH 7.4 + Toci
pH 6.4
pH 6.4 + Toci
pH 6.4
pH 6.4 + Toci
pH 7.4
pH 6.4
pH 7.4
pH 6.4
pH 7.4
pH 7.4
pH 7.4 + Toci
pH 7.4 + Toci
pH 7.4 + Toci
pH 6.4 + Toci
pH 7.4 + Toci
pH 6.4 + Toci
pH 6.4 + Toci
pH 6.4 + Toci
pH 6.4
pH 6.4
pH 6.4
pH 6.4
pH 7.4
pH 7.4
pH 7.4
pH 7.4
Id-, 15.02.2024 HK-2 p-10, coculture, 3 h
Ponceau
IL-6
pH 7.4 + Toci
pH 7.4 + Toci
pH 6.4 + Toci
pH 6.4 + Toci
pH 6.4
pH 6.4
pH 7.4
pH 7.4
pH 7.4 + Toci
pH 7.4 + Toci
pH 6.4 + Toci
pH 6.4 + Toci
pH 6.4
pH 6.4
pH 7.4
pH 7.4

## Slide 94
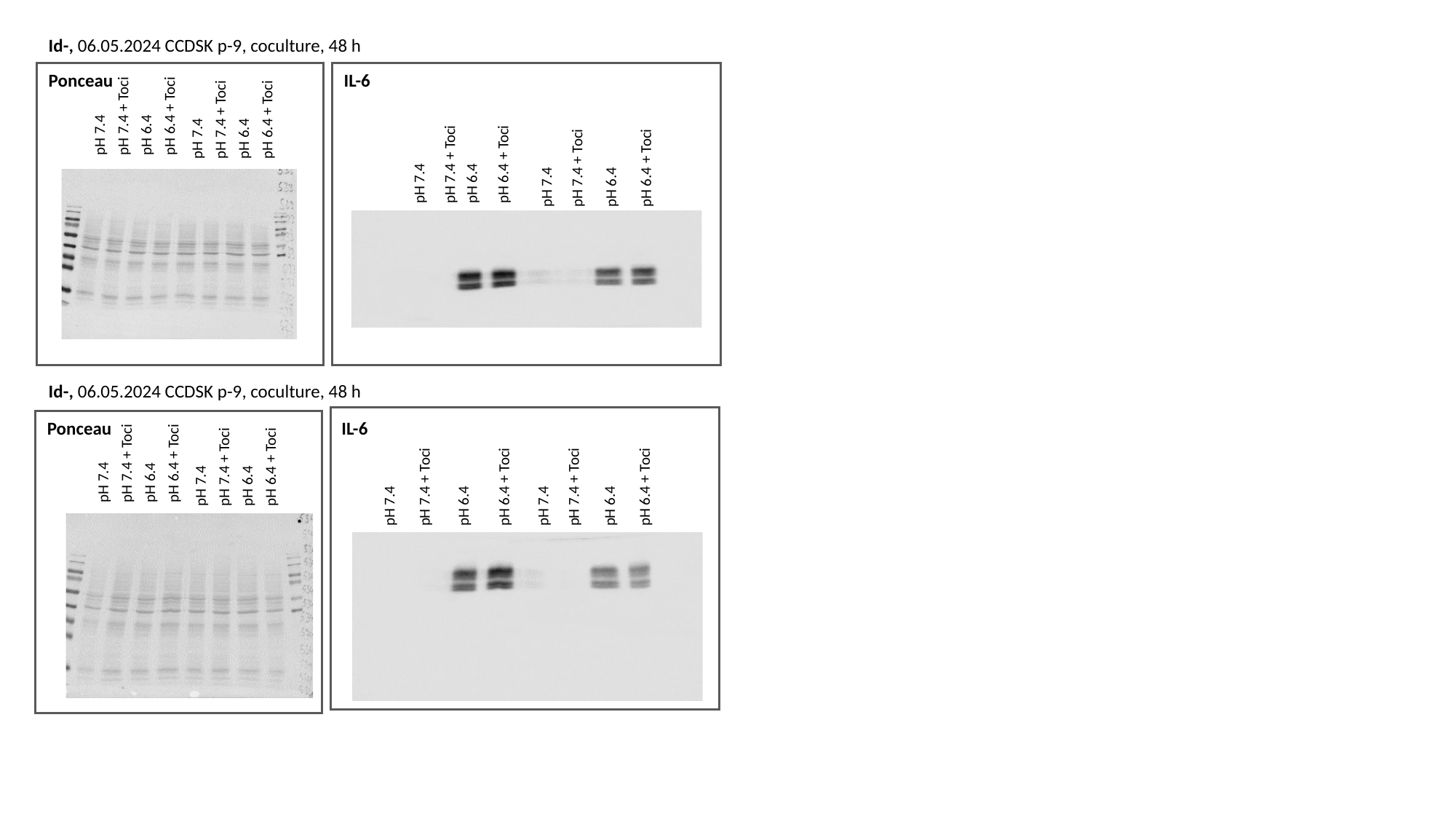

Id-, 06.05.2024 CCDSK p-9, coculture, 48 h
IL-6
Ponceau
pH 7.4 + Toci
pH 7.4 + Toci
pH 6.4 + Toci
pH 6.4 + Toci
pH 6.4
pH 6.4
pH 7.4
pH 7.4
pH 7.4 + Toci
pH 7.4 + Toci
pH 6.4 + Toci
pH 6.4 + Toci
pH 6.4
pH 6.4
pH 7.4
pH 7.4
Id-, 06.05.2024 CCDSK p-9, coculture, 48 h
Ponceau
IL-6
pH 7.4 + Toci
pH 7.4 + Toci
pH 6.4 + Toci
pH 6.4 + Toci
pH 6.4
pH 6.4
pH 7.4 + Toci
pH 7.4
pH 7.4 + Toci
pH 7.4
pH 6.4 + Toci
pH 6.4 + Toci
pH 6.4
pH 6.4
pH 7.4
pH 7.4

## Slide 95
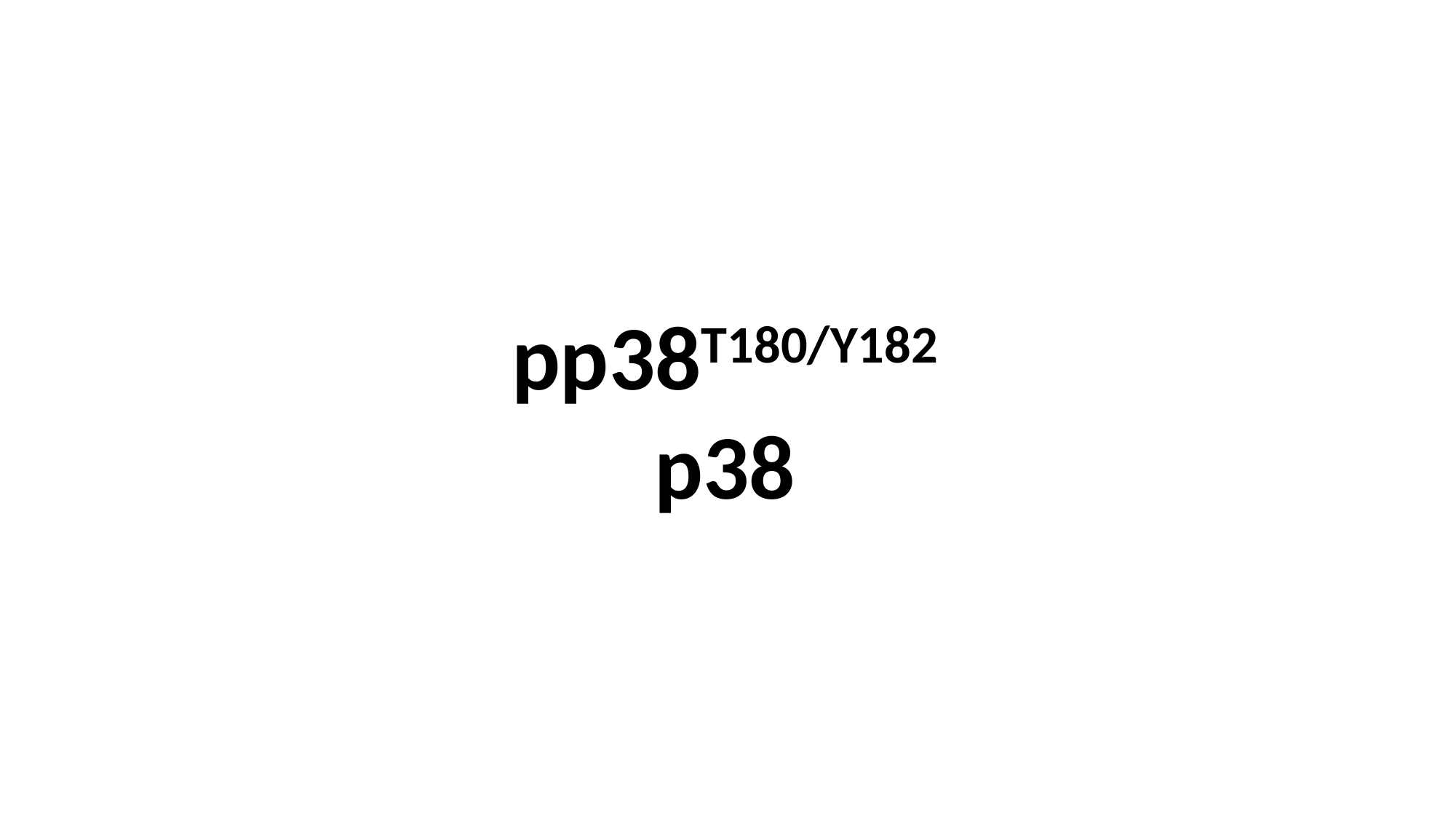

pp38T180/Y182
p38

## Slide 96
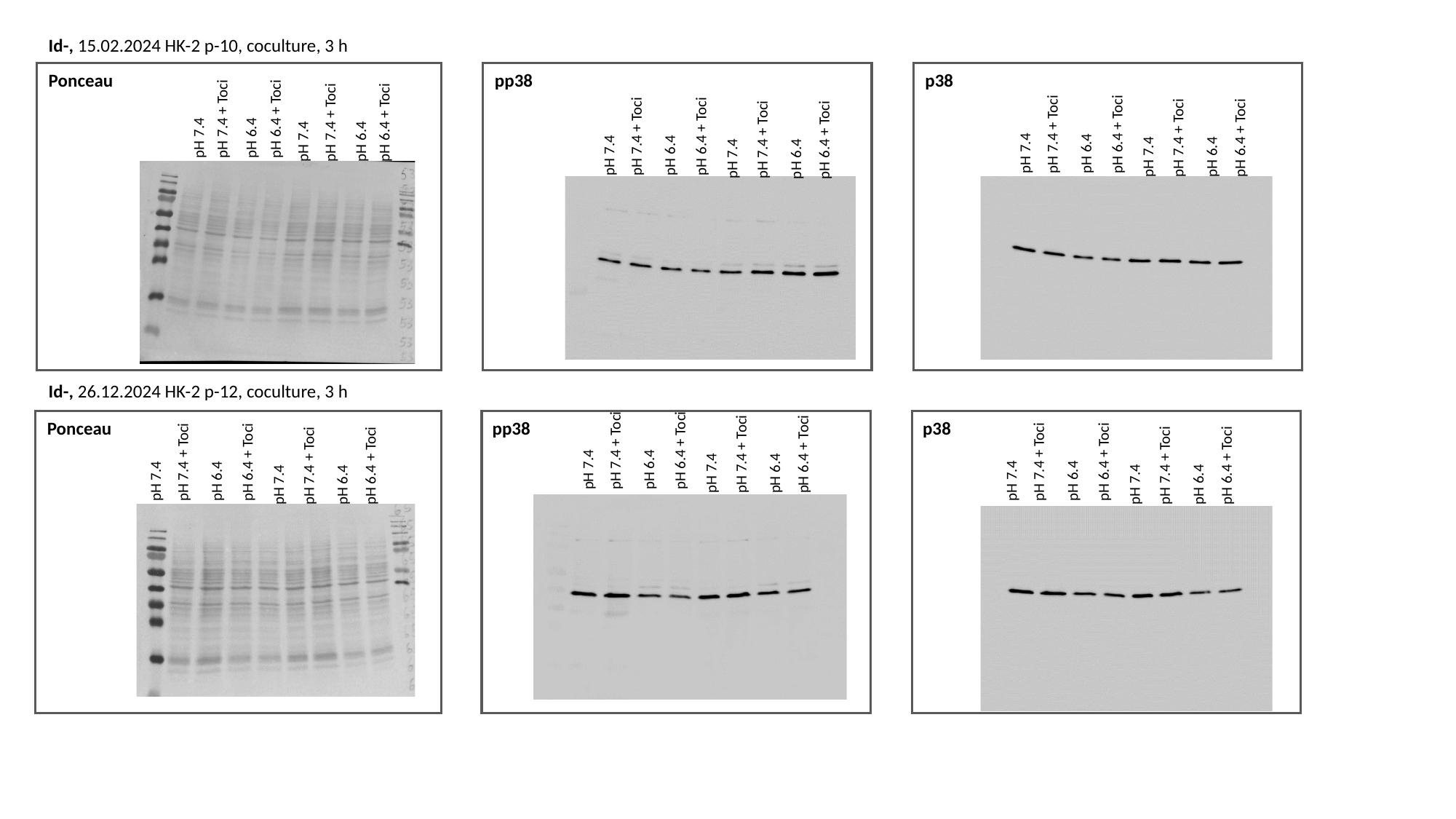

Id-, 15.02.2024 HK-2 p-10, coculture, 3 h
pp38
p38
Ponceau
pH 7.4 + Toci
pH 7.4 + Toci
pH 6.4 + Toci
pH 6.4 + Toci
pH 6.4
pH 7.4 + Toci
pH 7.4 + Toci
pH 7.4 + Toci
pH 6.4
pH 7.4 + Toci
pH 6.4 + Toci
pH 6.4 + Toci
pH 7.4
pH 6.4 + Toci
pH 6.4 + Toci
pH 7.4
pH 6.4
pH 6.4
pH 6.4
pH 6.4
pH 7.4
pH 7.4
pH 7.4
pH 7.4
Id-, 26.12.2024 HK-2 p-12, coculture, 3 h
Ponceau
pp38
p38
pH 7.4 + Toci
pH 7.4 + Toci
pH 6.4 + Toci
pH 6.4 + Toci
pH 7.4 + Toci
pH 7.4 + Toci
pH 7.4 + Toci
pH 7.4 + Toci
pH 6.4
pH 6.4 + Toci
pH 6.4 + Toci
pH 6.4
pH 6.4 + Toci
pH 6.4 + Toci
pH 7.4
pH 6.4
pH 6.4
pH 7.4
pH 6.4
pH 6.4
pH 7.4
pH 7.4
pH 7.4
pH 7.4

## Slide 97
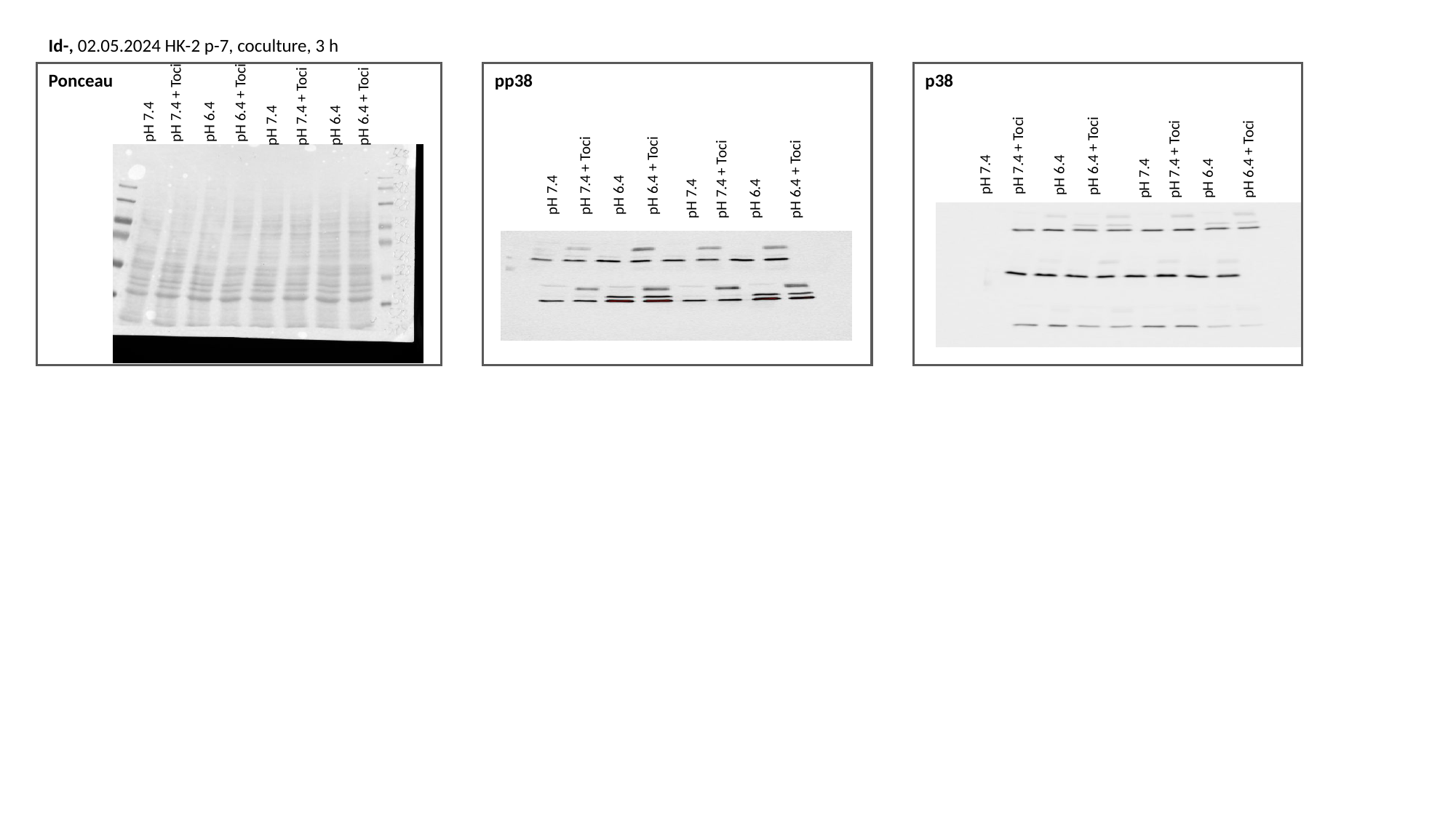

Id-, 02.05.2024 HK-2 p-7, coculture, 3 h
pp38
p38
Ponceau
pH 7.4 + Toci
pH 7.4 + Toci
pH 6.4 + Toci
pH 6.4 + Toci
pH 6.4
pH 6.4
pH 7.4
pH 7.4
pH 7.4 + Toci
pH 7.4 + Toci
pH 6.4 + Toci
pH 6.4 + Toci
pH 6.4
pH 6.4
pH 7.4 + Toci
pH 7.4 + Toci
pH 7.4
pH 6.4 + Toci
pH 7.4
pH 6.4 + Toci
pH 6.4
pH 6.4
pH 7.4
pH 7.4

## Slide 98
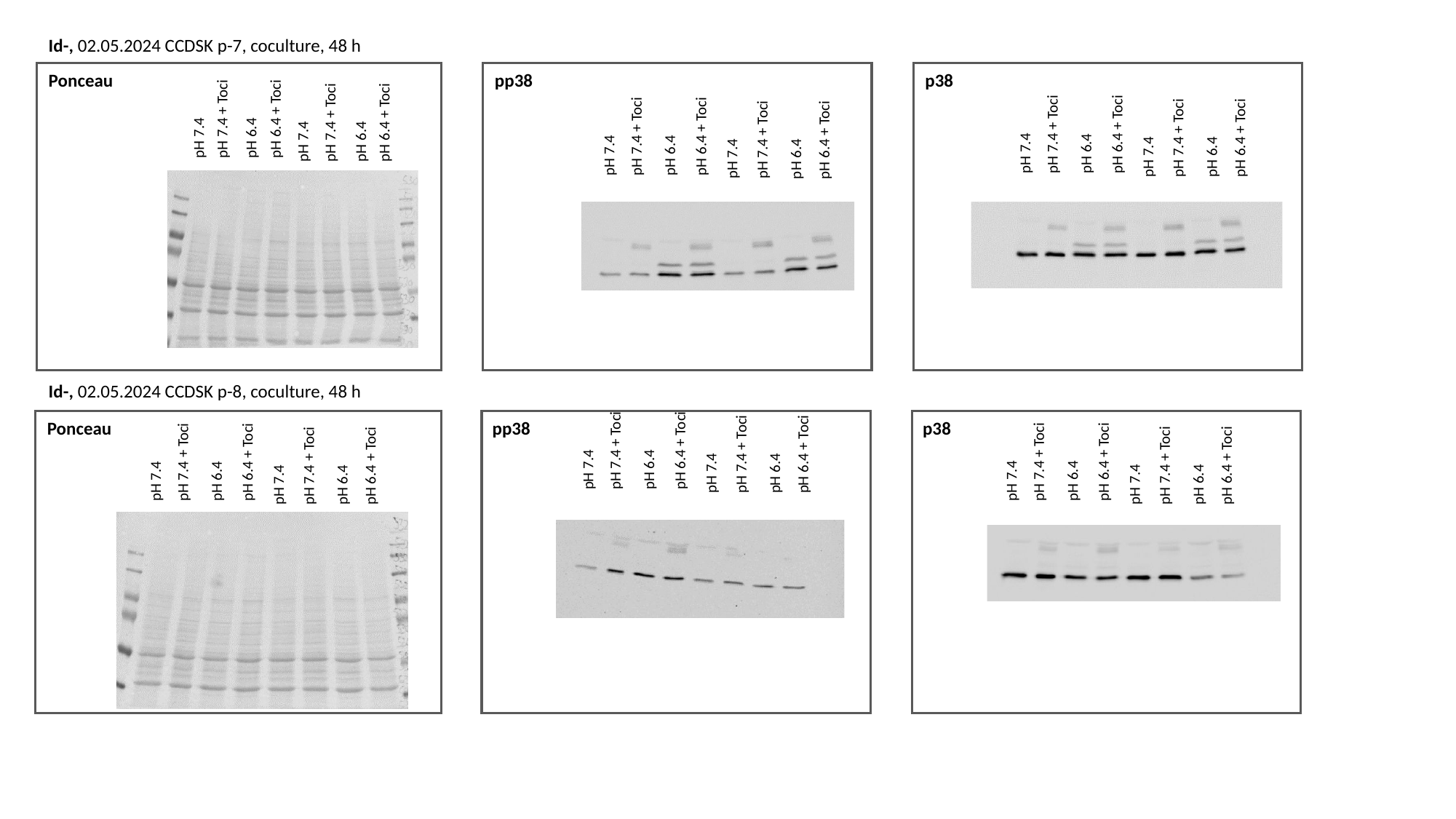

Id-, 02.05.2024 CCDSK p-7, coculture, 48 h
pp38
p38
Ponceau
pH 7.4 + Toci
pH 7.4 + Toci
pH 6.4 + Toci
pH 6.4 + Toci
pH 6.4
pH 7.4 + Toci
pH 7.4 + Toci
pH 7.4 + Toci
pH 6.4
pH 7.4 + Toci
pH 6.4 + Toci
pH 6.4 + Toci
pH 7.4
pH 6.4 + Toci
pH 6.4 + Toci
pH 7.4
pH 6.4
pH 6.4
pH 6.4
pH 6.4
pH 7.4
pH 7.4
pH 7.4
pH 7.4
Id-, 02.05.2024 CCDSK p-8, coculture, 48 h
Ponceau
pp38
p38
pH 7.4 + Toci
pH 7.4 + Toci
pH 6.4 + Toci
pH 6.4 + Toci
pH 7.4 + Toci
pH 7.4 + Toci
pH 7.4 + Toci
pH 7.4 + Toci
pH 6.4
pH 6.4 + Toci
pH 6.4 + Toci
pH 6.4
pH 6.4 + Toci
pH 6.4 + Toci
pH 7.4
pH 6.4
pH 6.4
pH 7.4
pH 6.4
pH 6.4
pH 7.4
pH 7.4
pH 7.4
pH 7.4

## Slide 99
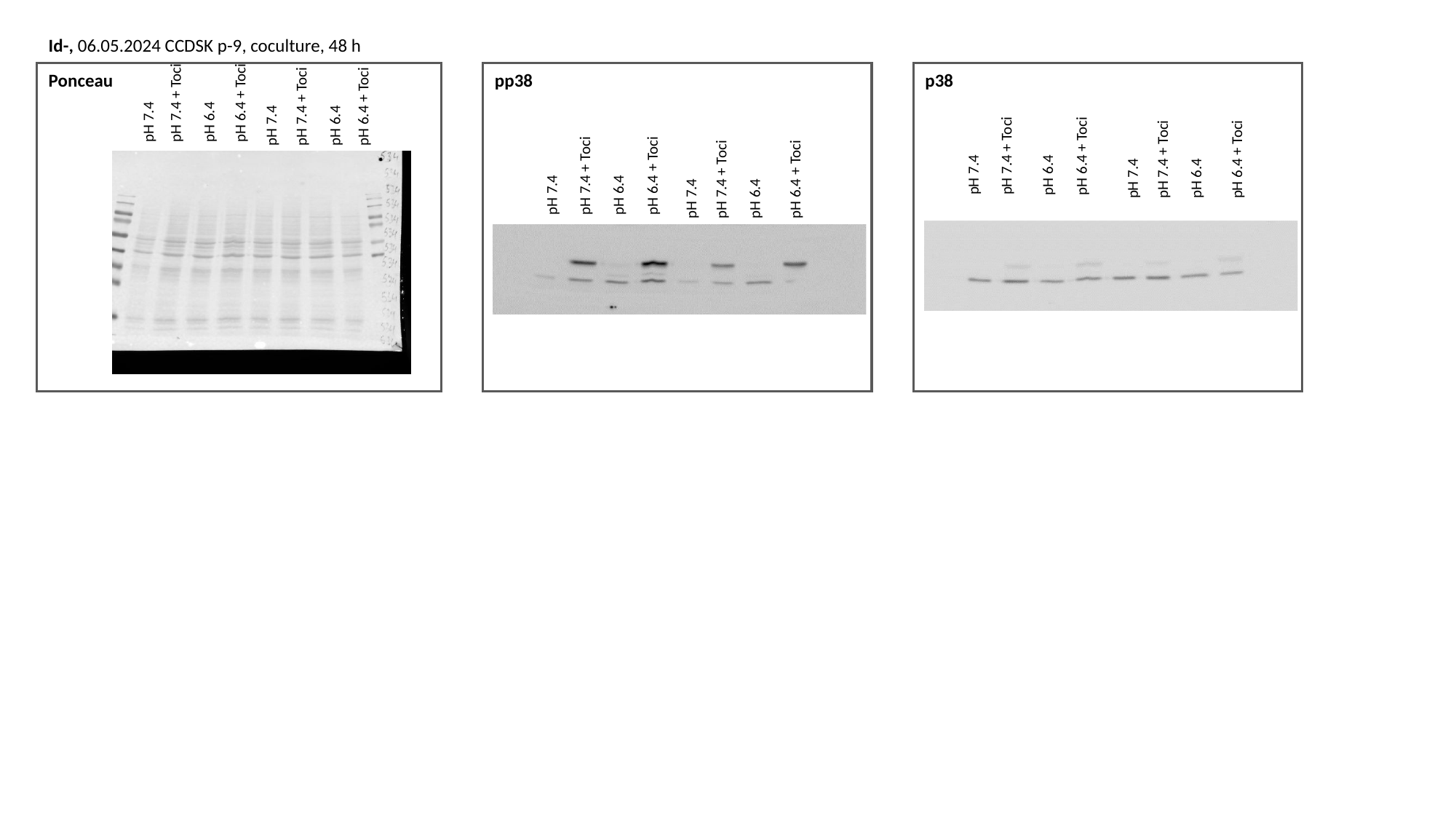

Id-, 06.05.2024 CCDSK p-9, coculture, 48 h
pp38
p38
Ponceau
pH 7.4 + Toci
pH 7.4 + Toci
pH 6.4 + Toci
pH 6.4 + Toci
pH 6.4
pH 6.4
pH 7.4
pH 7.4
pH 7.4 + Toci
pH 7.4 + Toci
pH 6.4 + Toci
pH 6.4 + Toci
pH 6.4
pH 6.4
pH 7.4 + Toci
pH 7.4 + Toci
pH 7.4
pH 6.4 + Toci
pH 7.4
pH 6.4 + Toci
pH 6.4
pH 6.4
pH 7.4
pH 7.4

## Slide 100
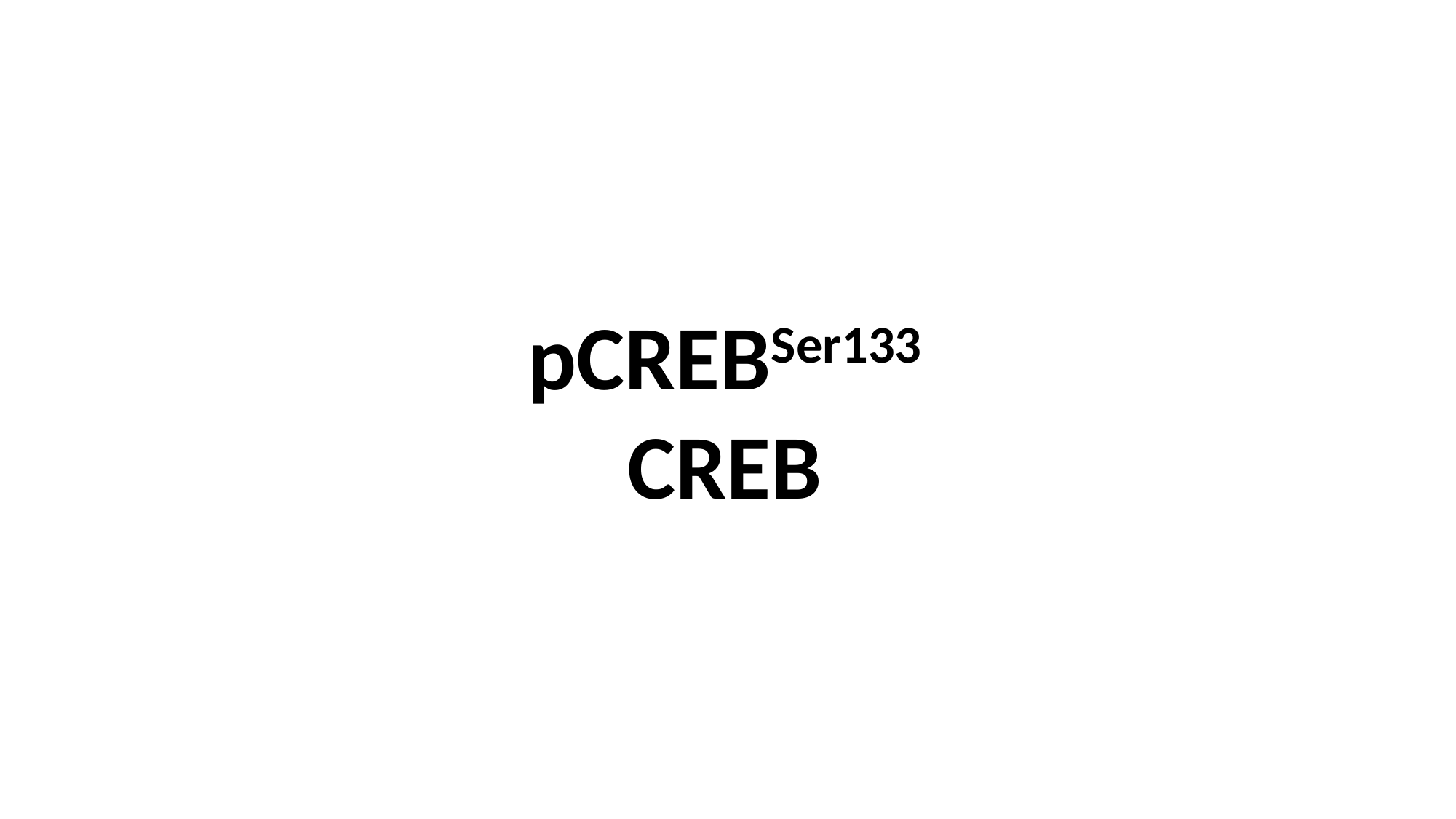

pCREBSer133
CREB

## Slide 101
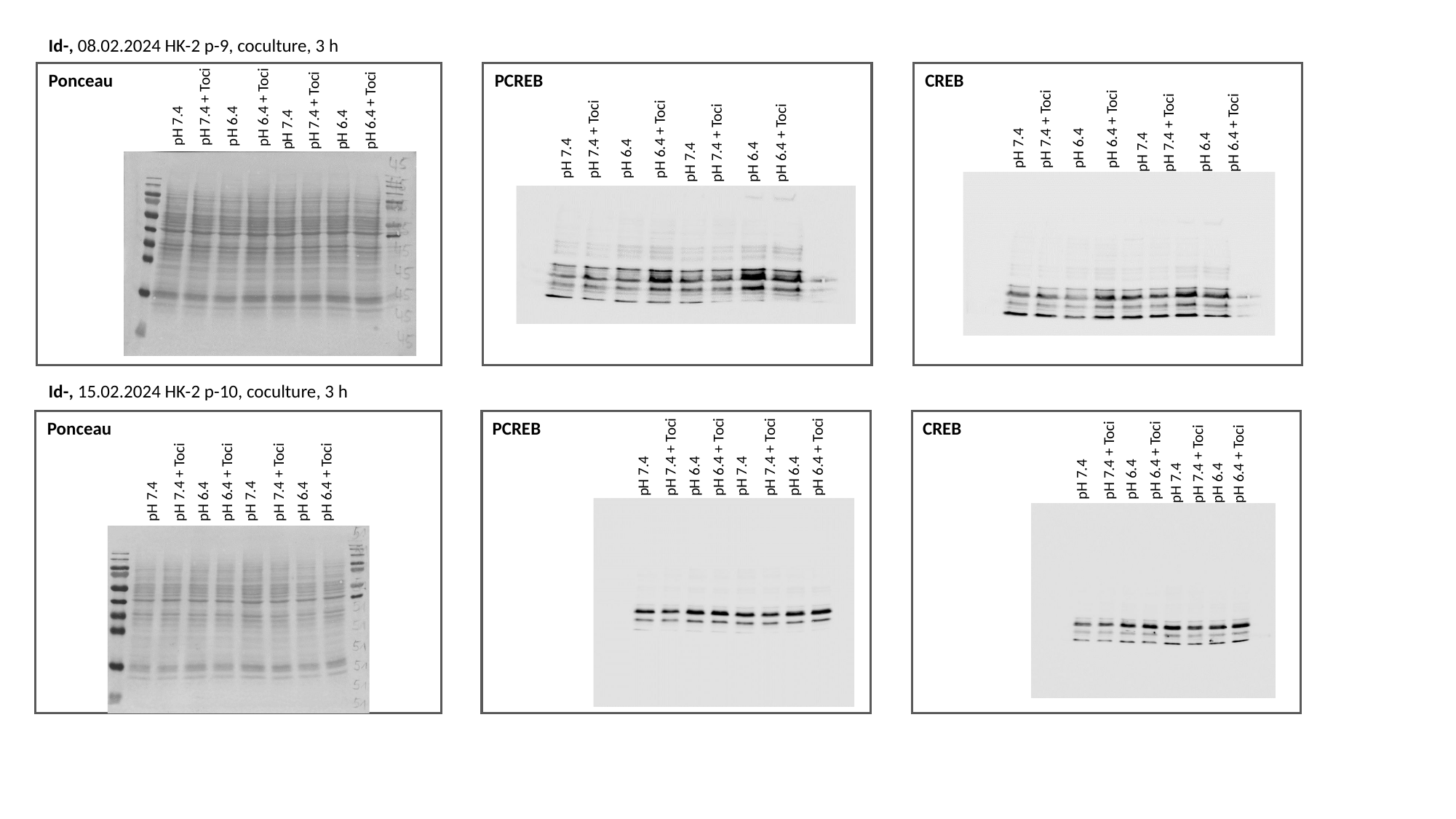

Id-, 08.02.2024 HK-2 p-9, coculture, 3 h
PCREB
CREB
Ponceau
pH 7.4 + Toci
pH 7.4 + Toci
pH 6.4 + Toci
pH 6.4 + Toci
pH 6.4
pH 6.4
pH 7.4 + Toci
pH 7.4
pH 7.4 + Toci
pH 7.4
pH 6.4 + Toci
pH 6.4 + Toci
pH 7.4 + Toci
pH 7.4 + Toci
pH 6.4
pH 6.4 + Toci
pH 6.4
pH 6.4 + Toci
pH 7.4
pH 6.4
pH 7.4
pH 6.4
pH 7.4
pH 7.4
Id-, 15.02.2024 HK-2 p-10, coculture, 3 h
Ponceau
PCREB
CREB
pH 7.4 + Toci
pH 7.4 + Toci
pH 7.4 + Toci
pH 6.4 + Toci
pH 6.4 + Toci
pH 7.4 + Toci
pH 6.4 + Toci
pH 6.4 + Toci
pH 6.4
pH 6.4
pH 6.4
pH 6.4
pH 7.4
pH 7.4
pH 7.4 + Toci
pH 7.4 + Toci
pH 7.4
pH 7.4
pH 6.4 + Toci
pH 6.4 + Toci
pH 6.4
pH 6.4
pH 7.4
pH 7.4

## Slide 102
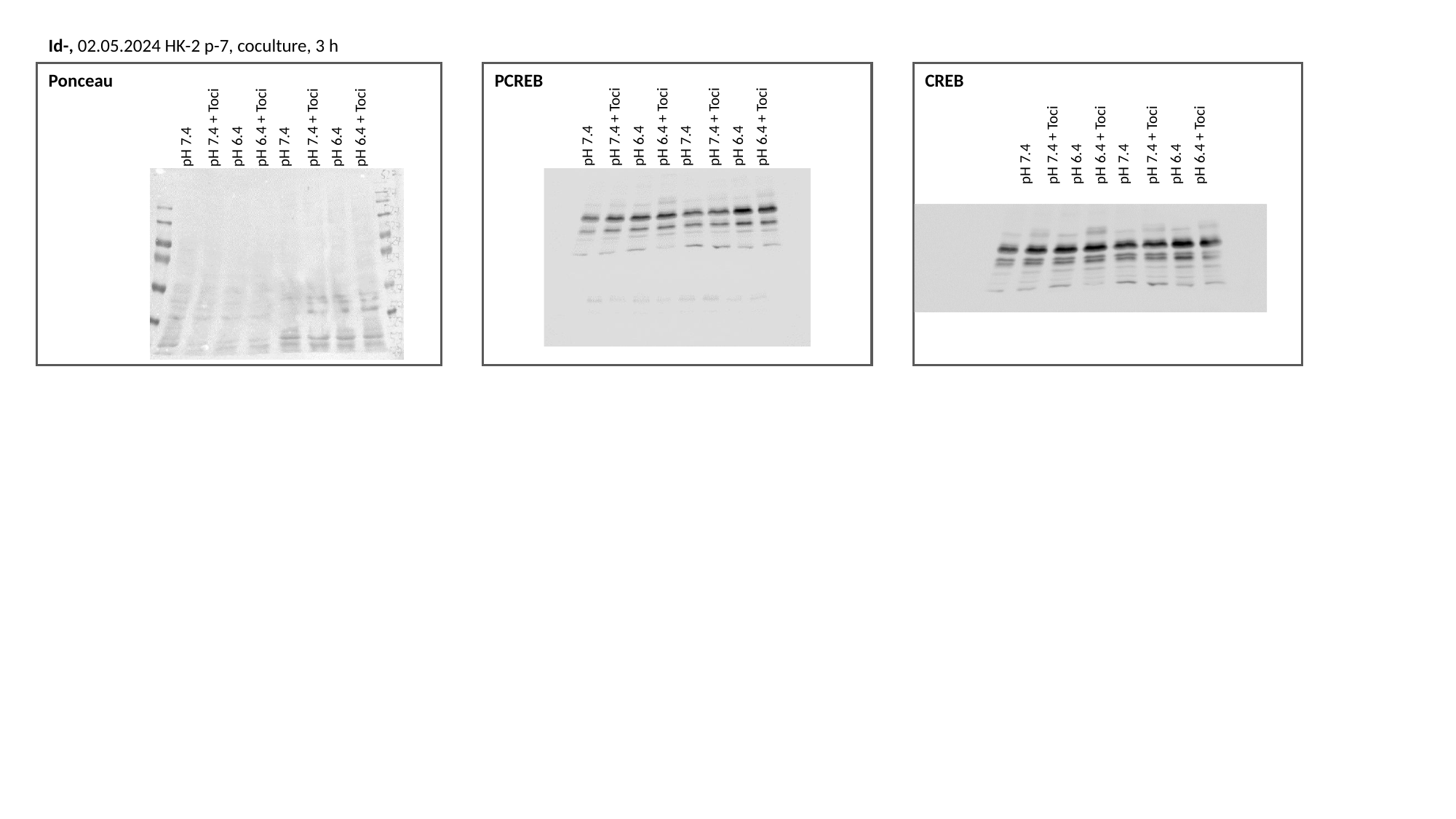

Id-, 02.05.2024 HK-2 p-7, coculture, 3 h
PCREB
CREB
Ponceau
pH 7.4 + Toci
pH 7.4 + Toci
pH 7.4 + Toci
pH 7.4 + Toci
pH 6.4 + Toci
pH 6.4 + Toci
pH 6.4 + Toci
pH 6.4 + Toci
pH 6.4
pH 6.4
pH 6.4
pH 6.4
pH 7.4 + Toci
pH 7.4 + Toci
pH 7.4
pH 7.4
pH 6.4 + Toci
pH 6.4 + Toci
pH 7.4
pH 7.4
pH 6.4
pH 6.4
pH 7.4
pH 7.4

## Slide 103
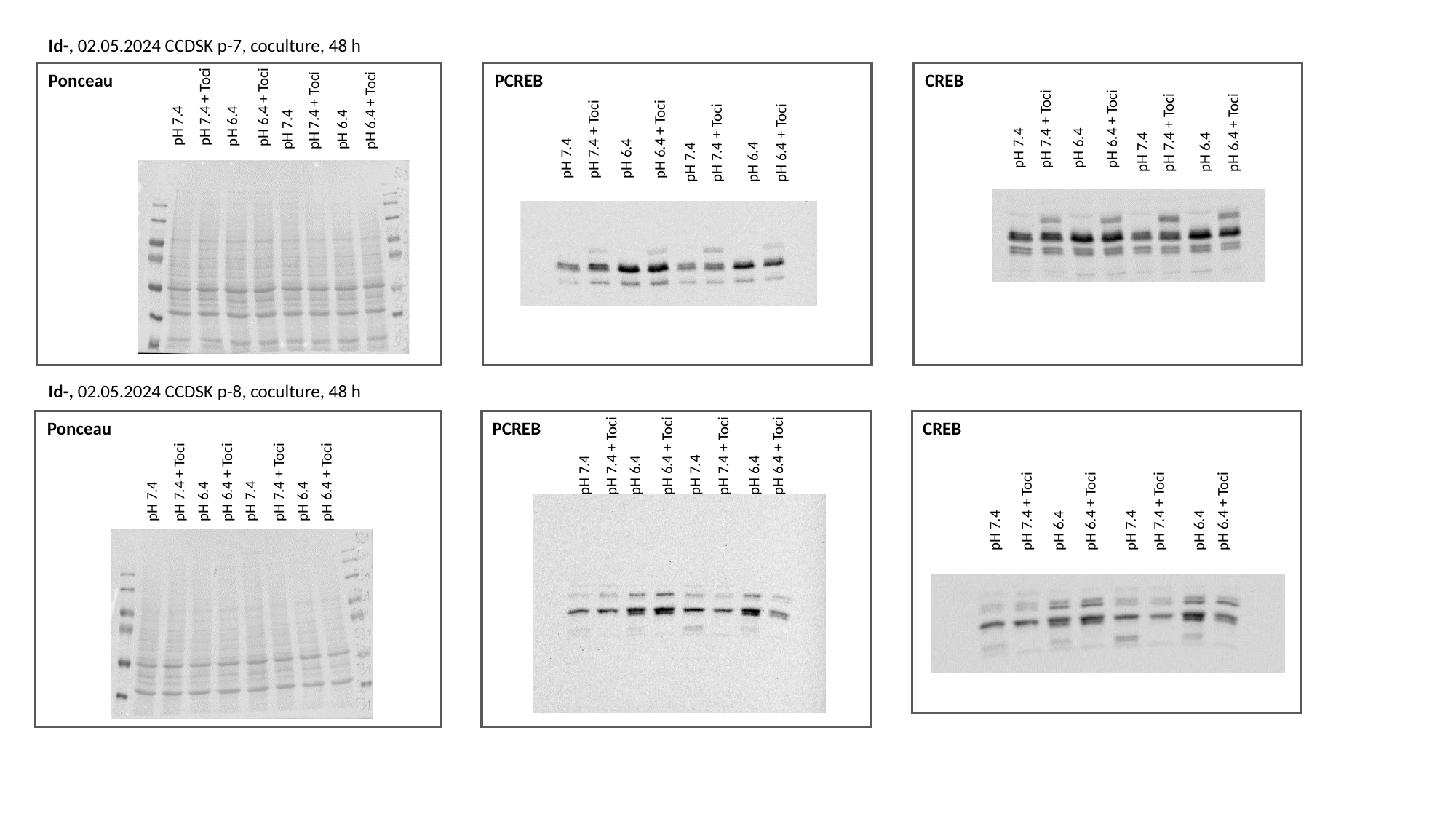

Id-, 02.05.2024 CCDSK p-7, coculture, 48 h
PCREB
CREB
Ponceau
pH 7.4 + Toci
pH 7.4 + Toci
pH 6.4 + Toci
pH 6.4 + Toci
pH 6.4
pH 6.4
pH 7.4 + Toci
pH 7.4
pH 7.4 + Toci
pH 7.4
pH 6.4 + Toci
pH 6.4 + Toci
pH 7.4 + Toci
pH 7.4 + Toci
pH 6.4
pH 6.4 + Toci
pH 6.4
pH 6.4 + Toci
pH 7.4
pH 6.4
pH 7.4
pH 6.4
pH 7.4
pH 7.4
Id-, 02.05.2024 CCDSK p-8, coculture, 48 h
Ponceau
PCREB
CREB
pH 7.4 + Toci
pH 7.4 + Toci
pH 6.4 + Toci
pH 6.4 + Toci
pH 6.4
pH 6.4
pH 7.4
pH 7.4
pH 7.4 + Toci
pH 7.4 + Toci
pH 6.4 + Toci
pH 6.4 + Toci
pH 6.4
pH 6.4
pH 7.4
pH 7.4
pH 7.4 + Toci
pH 7.4 + Toci
pH 6.4 + Toci
pH 6.4 + Toci
pH 6.4
pH 6.4
pH 7.4
pH 7.4

## Slide 104
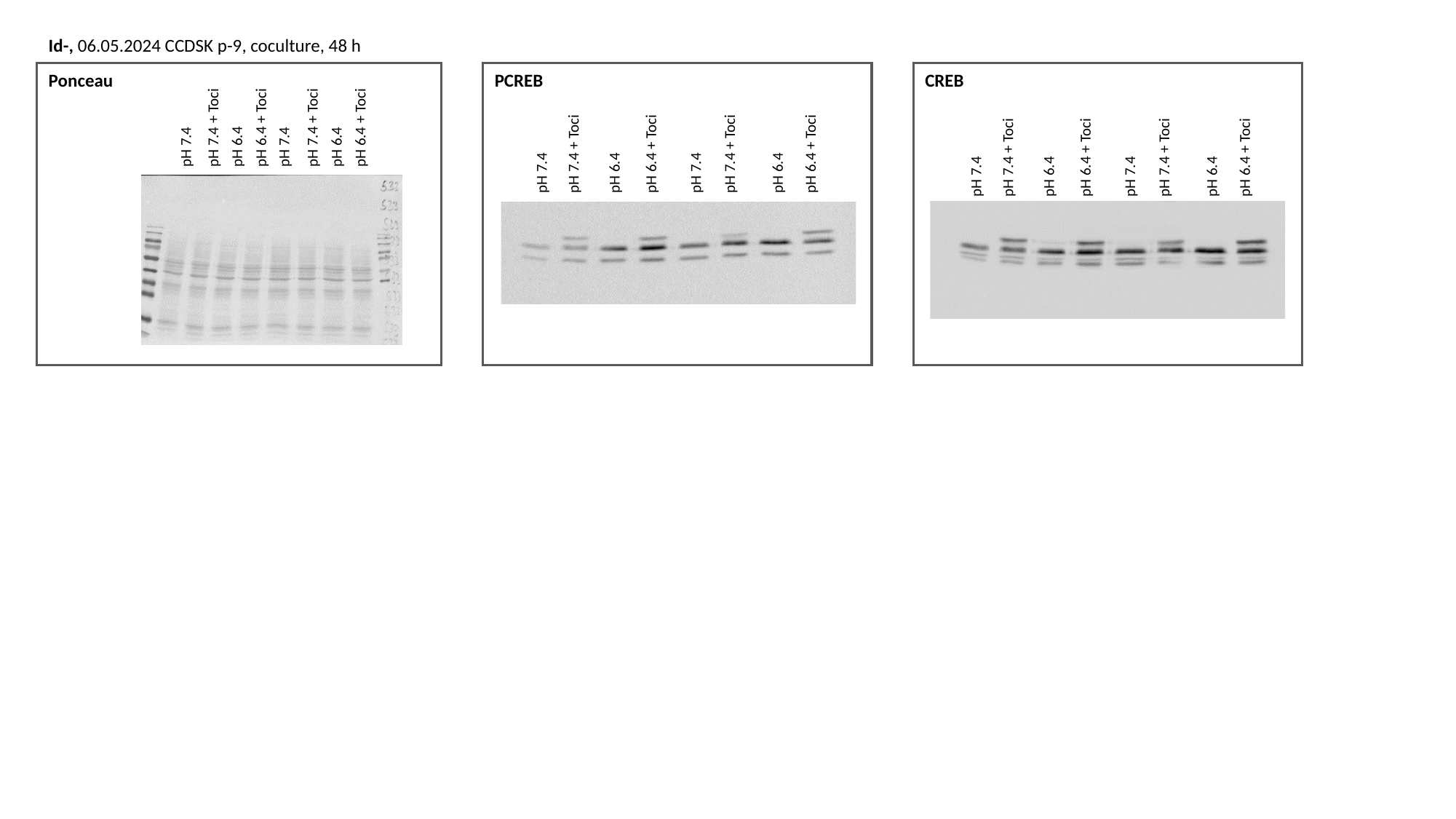

Id-, 06.05.2024 CCDSK p-9, coculture, 48 h
PCREB
CREB
Ponceau
pH 7.4 + Toci
pH 7.4 + Toci
pH 6.4 + Toci
pH 6.4 + Toci
pH 6.4
pH 6.4
pH 7.4
pH 7.4
pH 7.4 + Toci
pH 7.4 + Toci
pH 7.4 + Toci
pH 7.4 + Toci
pH 6.4 + Toci
pH 6.4 + Toci
pH 6.4 + Toci
pH 6.4 + Toci
pH 6.4
pH 6.4
pH 6.4
pH 6.4
pH 7.4
pH 7.4
pH 7.4
pH 7.4

## Slide 105
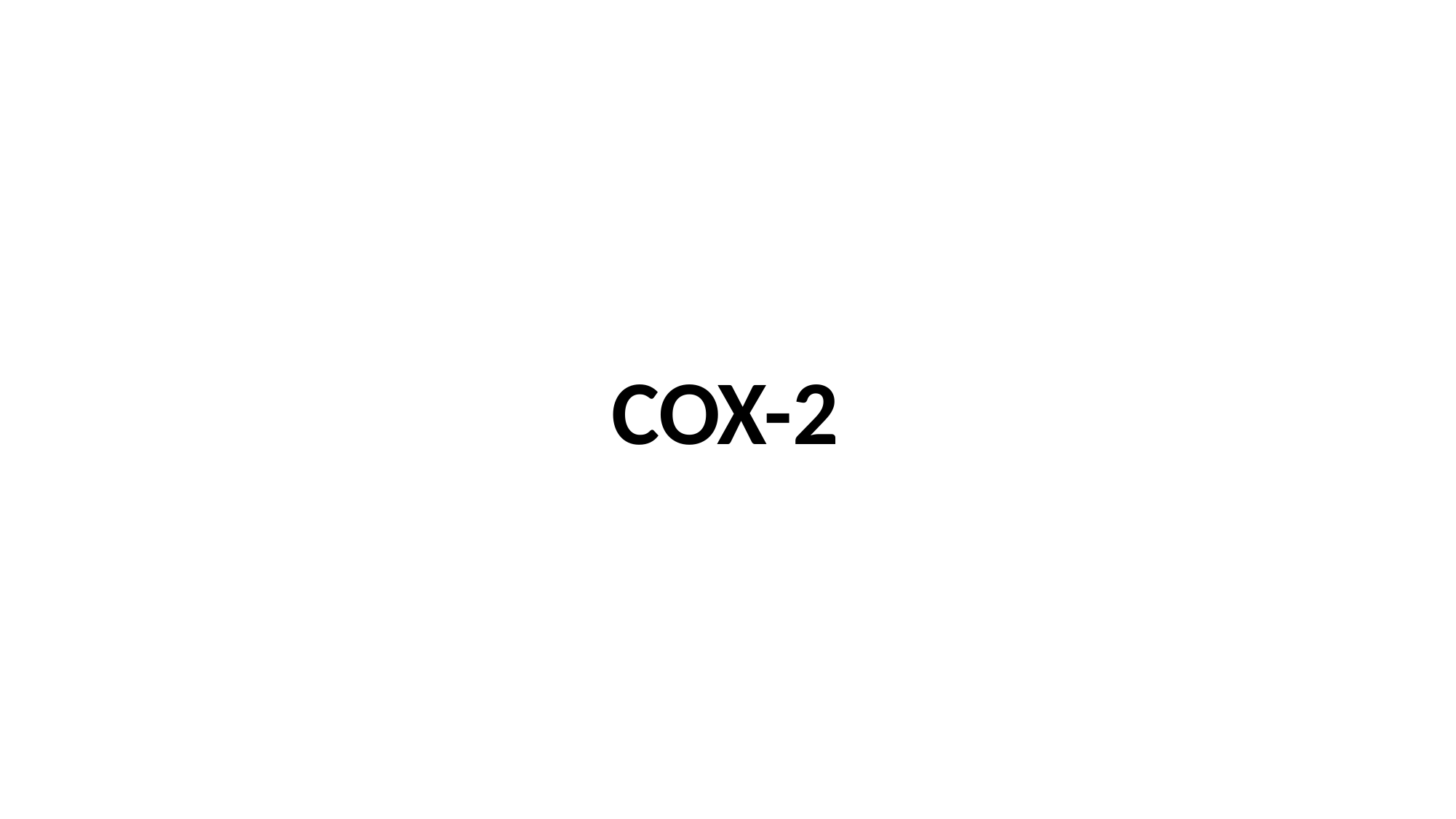

COX-2

## Slide 106
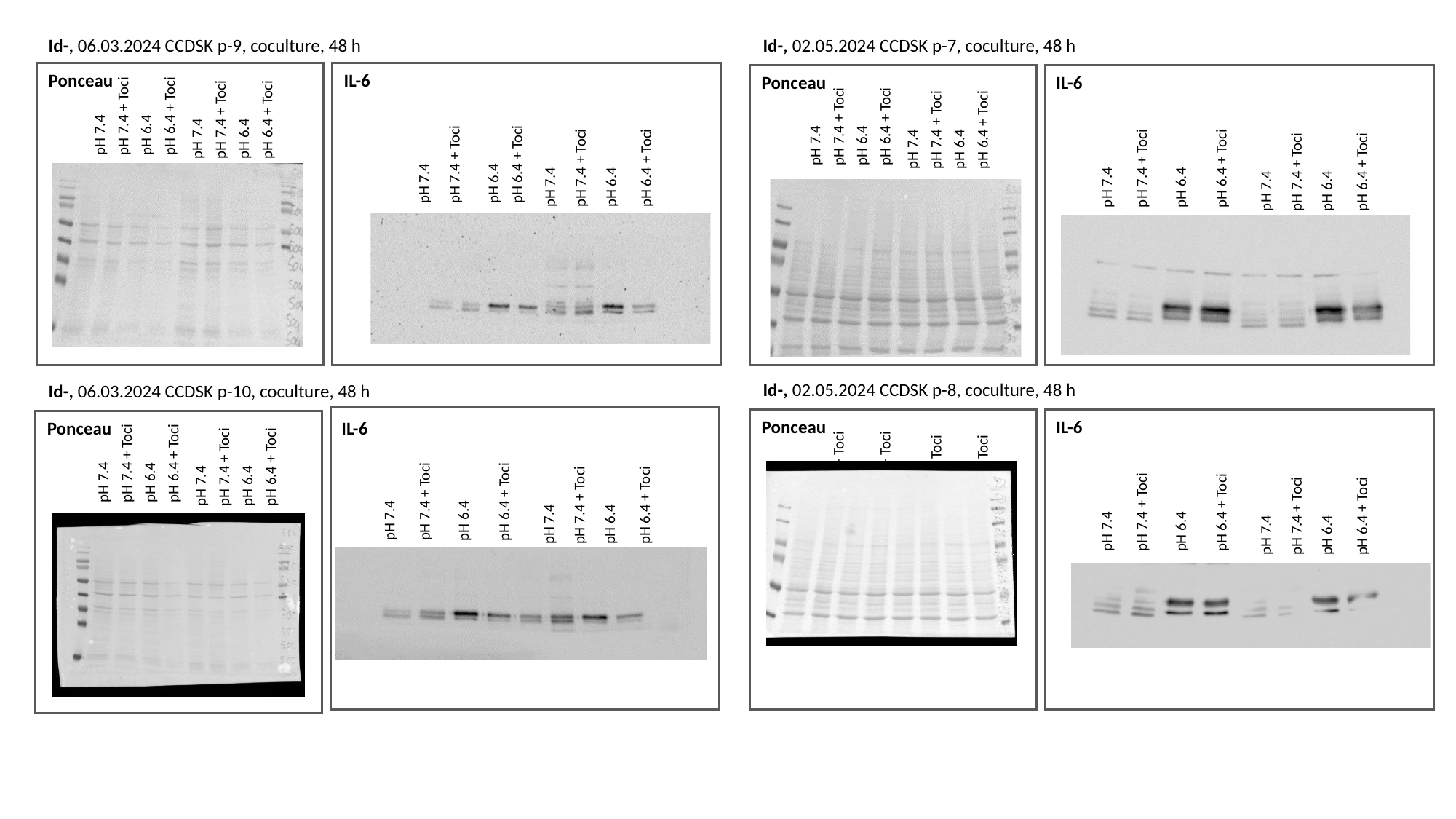

Id-, 06.03.2024 CCDSK p-9, coculture, 48 h
Id-, 02.05.2024 CCDSK p-7, coculture, 48 h
IL-6
Ponceau
Ponceau
IL-6
pH 7.4 + Toci
pH 7.4 + Toci
pH 6.4 + Toci
pH 6.4 + Toci
pH 7.4 + Toci
pH 7.4 + Toci
pH 6.4
pH 6.4 + Toci
pH 6.4
pH 6.4 + Toci
pH 7.4
pH 6.4
pH 7.4
pH 6.4
pH 7.4
pH 7.4
pH 7.4 + Toci
pH 7.4 + Toci
pH 7.4 + Toci
pH 6.4 + Toci
pH 7.4 + Toci
pH 6.4 + Toci
pH 6.4 + Toci
pH 6.4 + Toci
pH 6.4
pH 6.4
pH 6.4
pH 6.4
pH 7.4
pH 7.4
pH 7.4
pH 7.4
Id-, 02.05.2024 CCDSK p-8, coculture, 48 h
Id-, 06.03.2024 CCDSK p-10, coculture, 48 h
Ponceau
IL-6
Ponceau
IL-6
pH 7.4 + Toci
pH 7.4 + Toci
pH 6.4 + Toci
pH 7.4 + Toci
pH 6.4 + Toci
pH 7.4 + Toci
pH 6.4 + Toci
pH 6.4
pH 6.4 + Toci
pH 6.4
pH 6.4
pH 7.4
pH 6.4
pH 7.4
pH 7.4
pH 7.4
pH 7.4 + Toci
pH 7.4 + Toci
pH 6.4 + Toci
pH 6.4 + Toci
pH 7.4 + Toci
pH 7.4 + Toci
pH 6.4
pH 6.4 + Toci
pH 6.4
pH 6.4 + Toci
pH 7.4
pH 6.4
pH 7.4
pH 6.4
pH 7.4
pH 7.4

## Slide 107
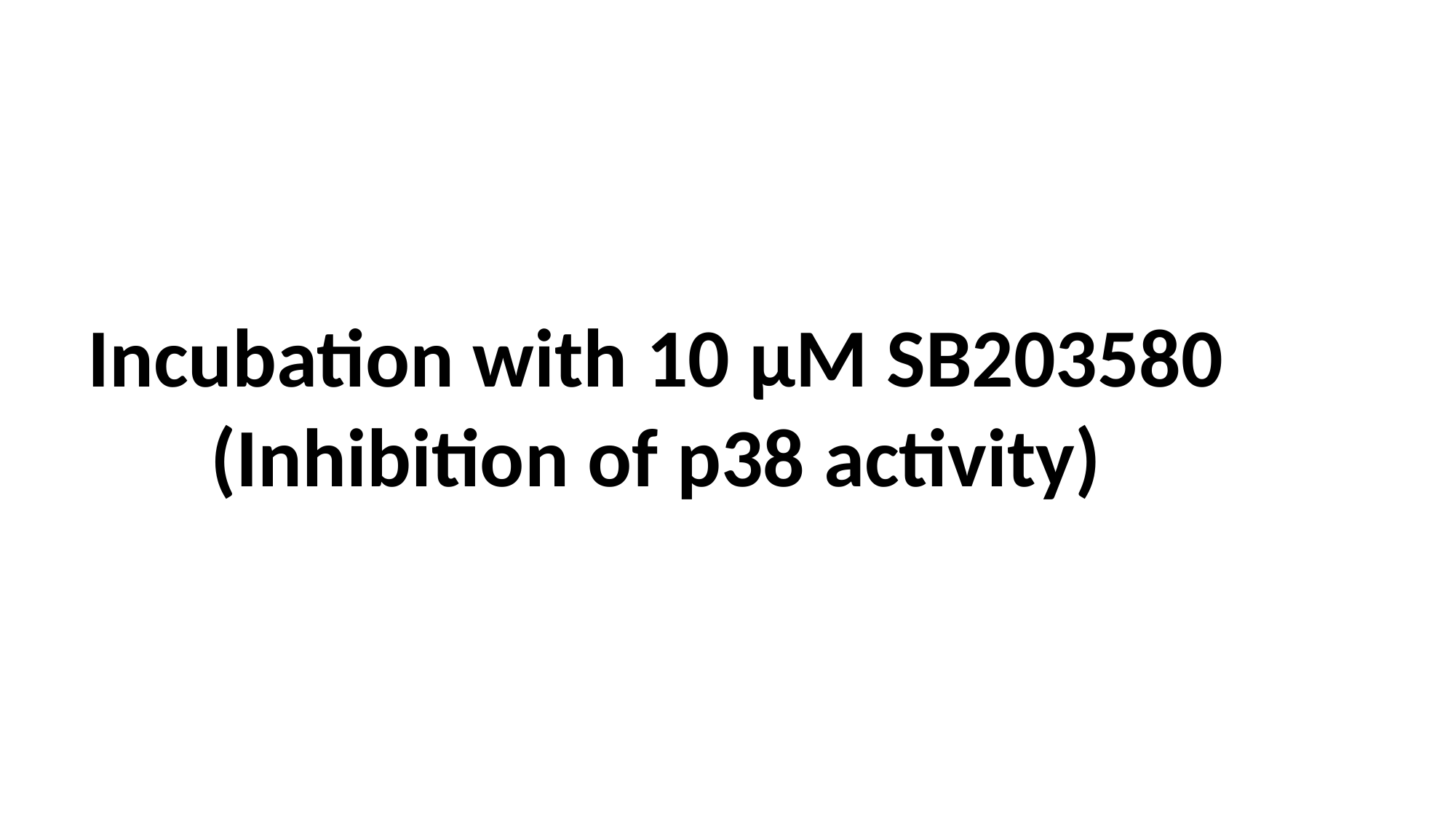

Incubation with 10 µM SB203580
(Inhibition of p38 activity)

## Slide 108
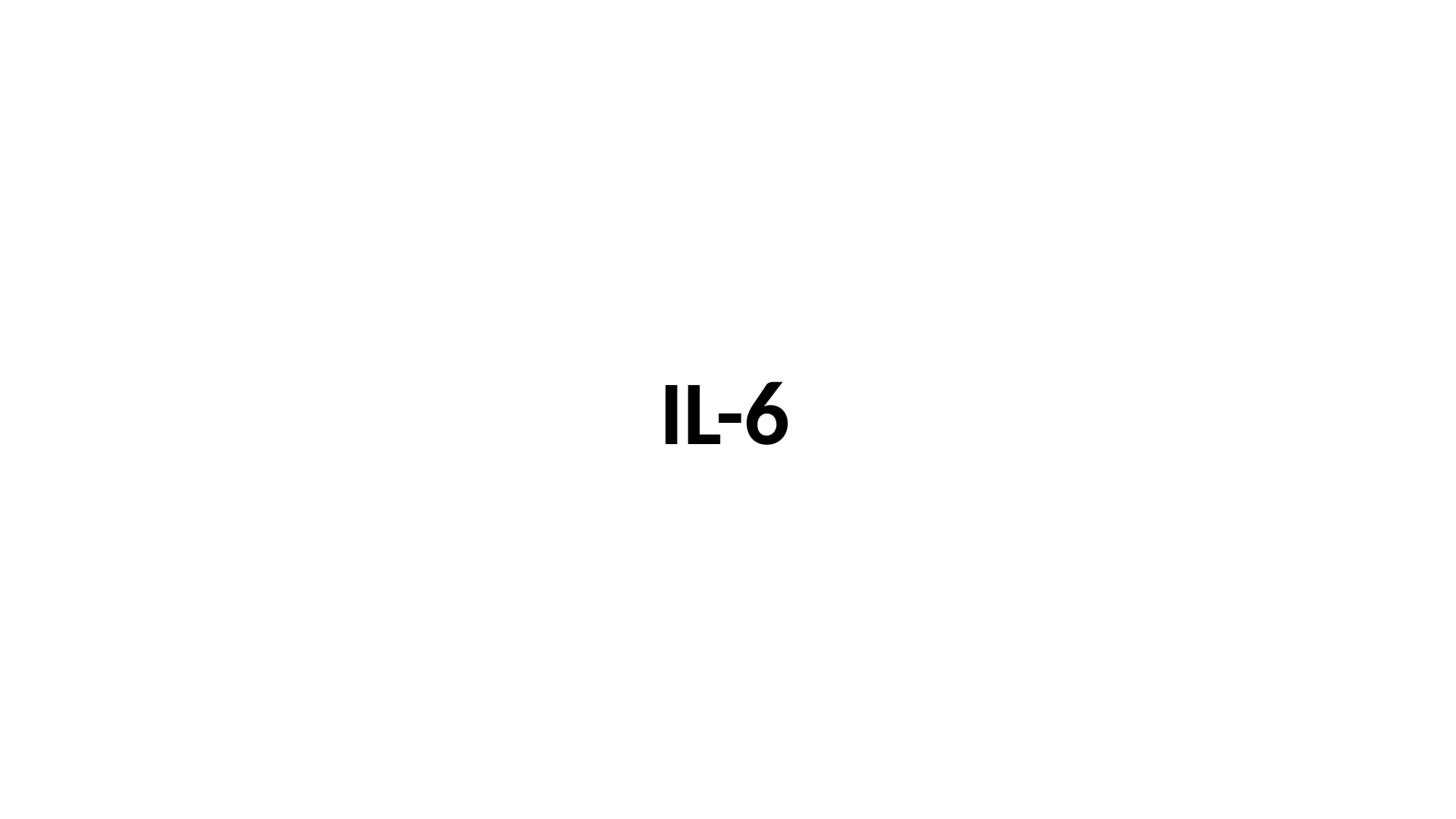

IL-6

## Slide 109
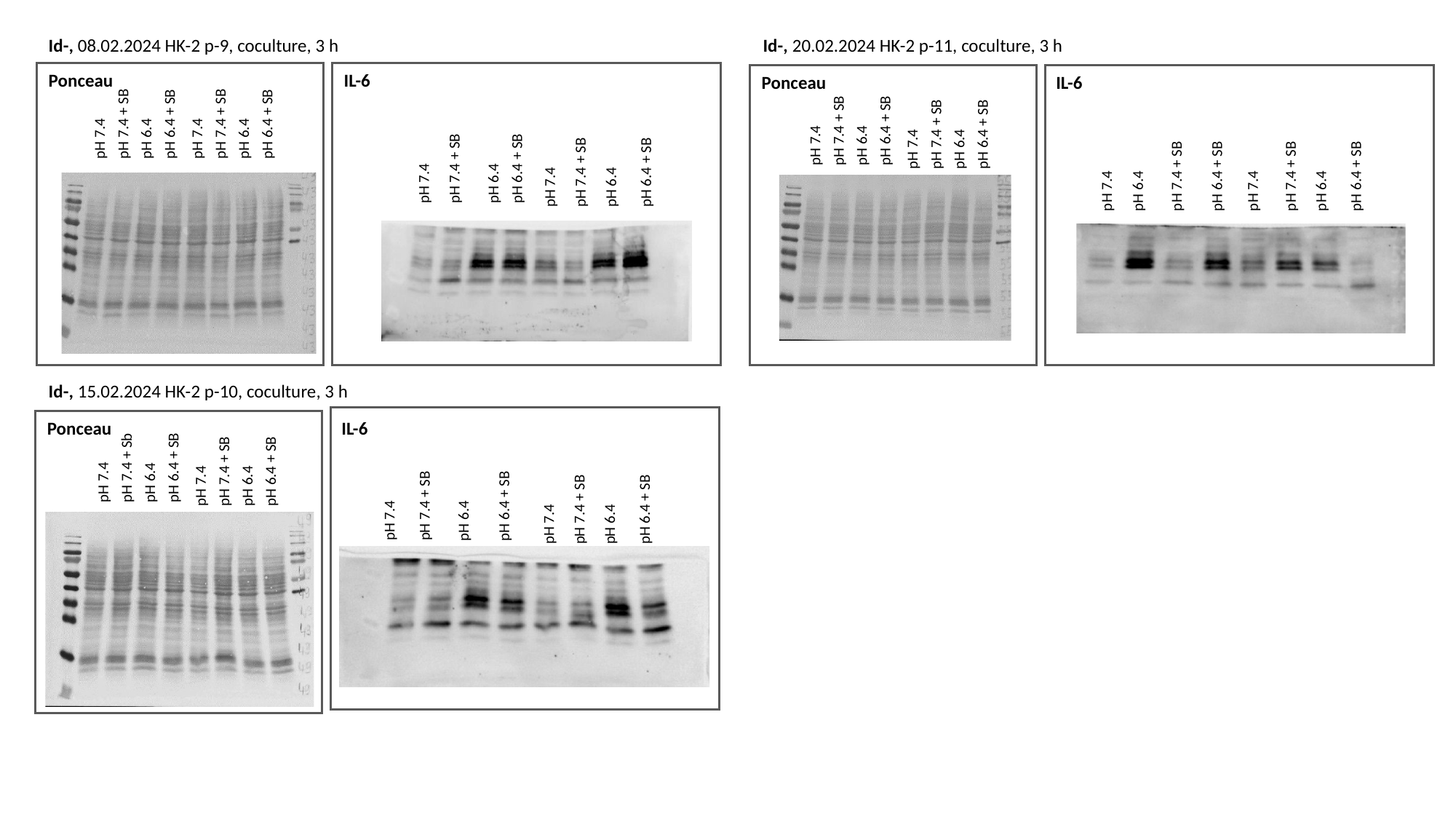

Id-, 08.02.2024 HK-2 p-9, coculture, 3 h
Id-, 20.02.2024 HK-2 p-11, coculture, 3 h
IL-6
Ponceau
Ponceau
IL-6
pH 7.4 + SB
pH 7.4 + SB
pH 6.4 + SB
pH 6.4 + SB
pH 7.4 + SB
pH 7.4 + SB
pH 6.4 + SB
pH 6.4
pH 6.4
pH 6.4 + SB
pH 6.4
pH 7.4
pH 7.4
pH 6.4
pH 7.4
pH 7.4
pH 7.4 + SB
pH 7.4 + SB
pH 6.4 + SB
pH 7.4 + SB
pH 7.4 + SB
pH 6.4 + SB
pH 6.4 + SB
pH 6.4 + SB
pH 6.4
pH 6.4
pH 6.4
pH 6.4
pH 7.4
pH 7.4
pH 7.4
pH 7.4
Id-, 15.02.2024 HK-2 p-10, coculture, 3 h
Ponceau
IL-6
pH 7.4 + Sb
pH 7.4 + SB
pH 6.4 + SB
pH 6.4 + SB
pH 6.4
pH 6.4
pH 7.4
pH 7.4
pH 7.4 + SB
pH 7.4 + SB
pH 6.4 + SB
pH 6.4 + SB
pH 6.4
pH 6.4
pH 7.4
pH 7.4

## Slide 110
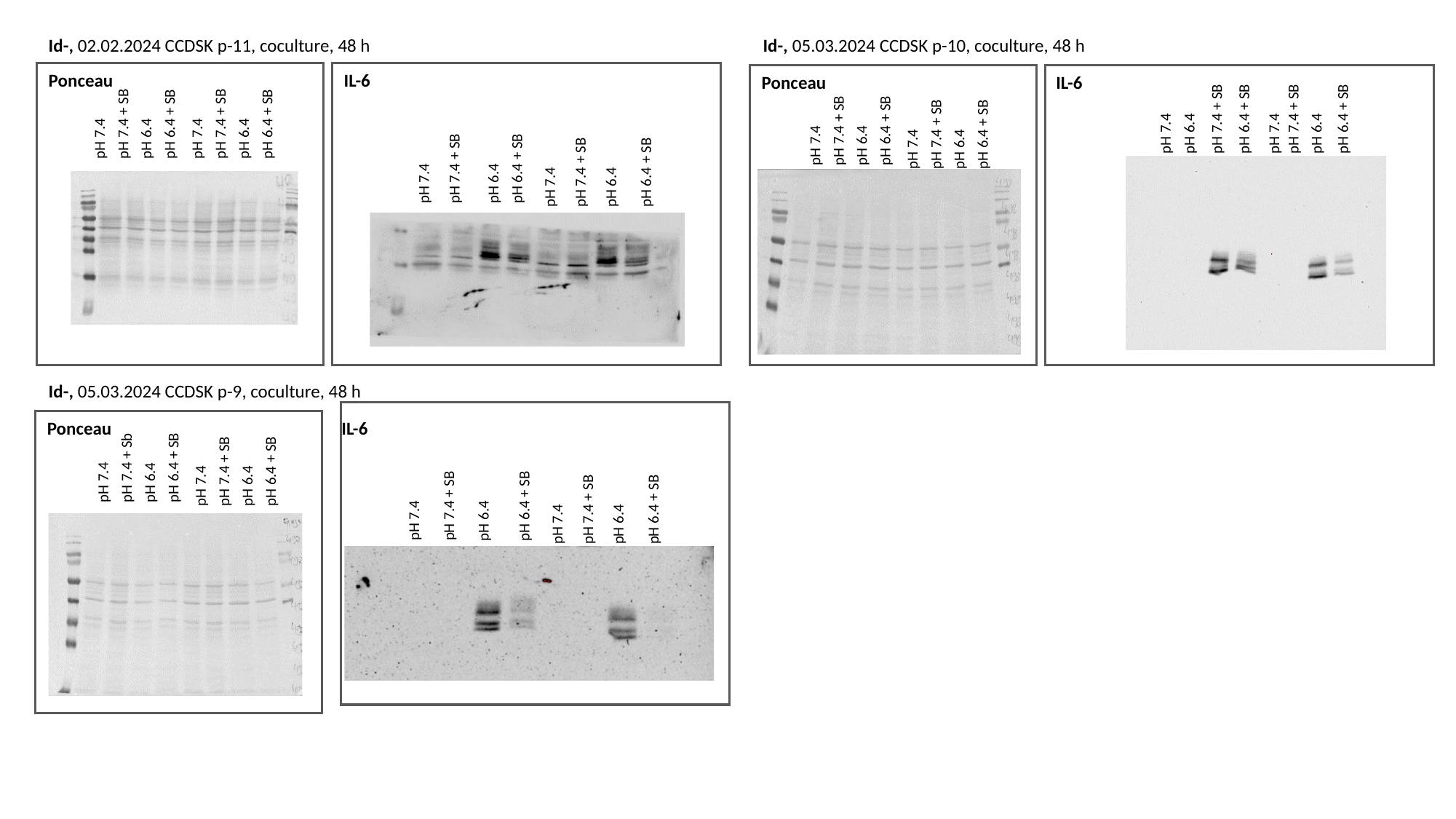

Id-, 02.02.2024 CCDSK p-11, coculture, 48 h
Id-, 05.03.2024 CCDSK p-10, coculture, 48 h
IL-6
Ponceau
Ponceau
IL-6
pH 7.4 + SB
pH 7.4 + SB
pH 7.4 + SB
pH 6.4 + SB
pH 6.4 + SB
pH 7.4 + SB
pH 6.4 + SB
pH 6.4 + SB
pH 7.4 + SB
pH 7.4 + SB
pH 6.4
pH 6.4
pH 6.4 + SB
pH 6.4
pH 6.4
pH 6.4 + SB
pH 7.4
pH 7.4
pH 6.4
pH 7.4
pH 7.4
pH 6.4
pH 7.4
pH 7.4
pH 7.4 + SB
pH 7.4 + SB
pH 6.4 + SB
pH 6.4 + SB
pH 6.4
pH 6.4
pH 7.4
pH 7.4
Id-, 05.03.2024 CCDSK p-9, coculture, 48 h
Ponceau
IL-6
pH 7.4 + Sb
pH 7.4 + SB
pH 6.4 + SB
pH 6.4 + SB
pH 6.4
pH 6.4
pH 7.4
pH 7.4
pH 7.4 + SB
pH 7.4 + SB
pH 6.4 + SB
pH 6.4 + SB
pH 6.4
pH 6.4
pH 7.4
pH 7.4

## Slide 111
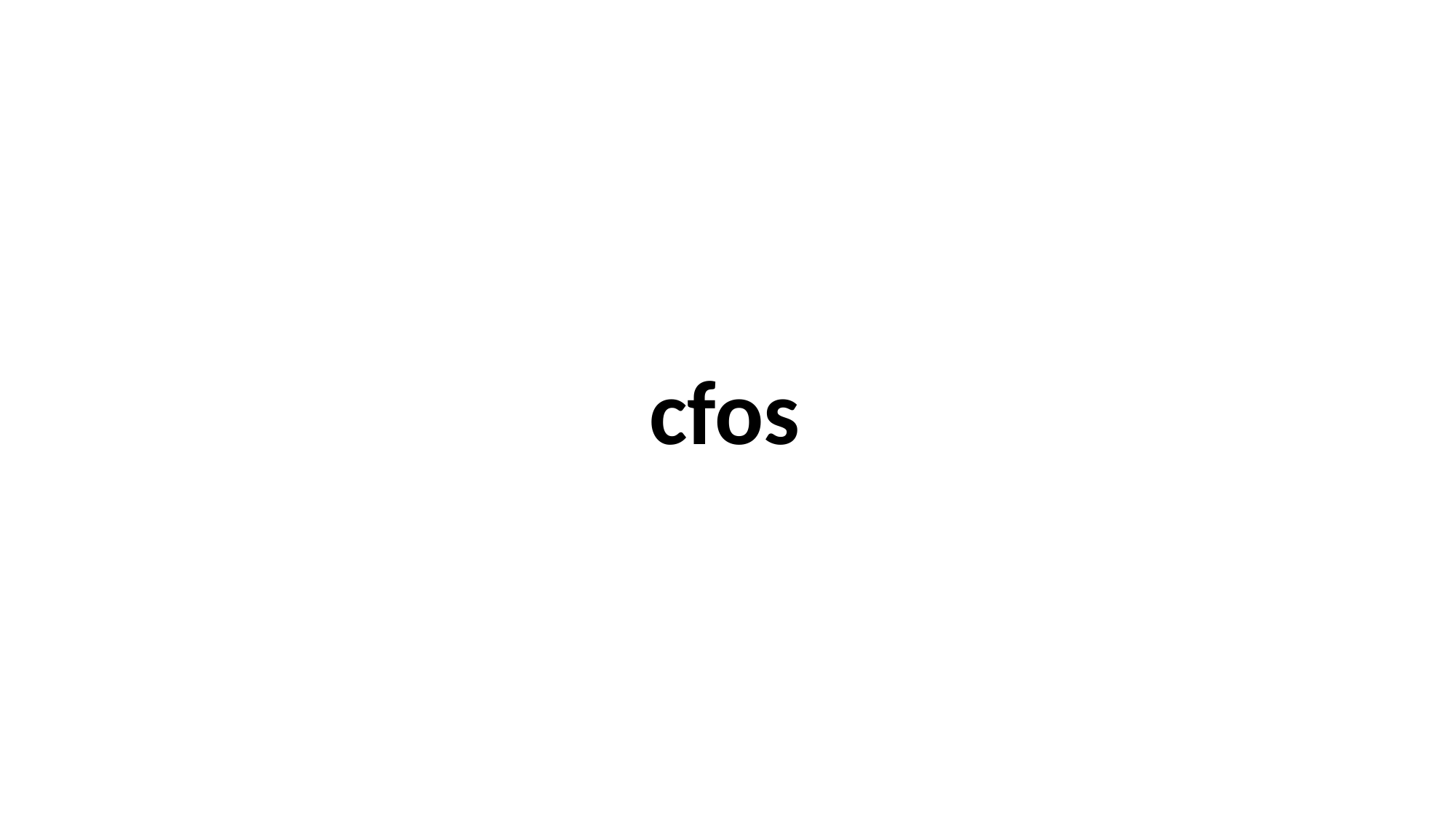

cfos

## Slide 112
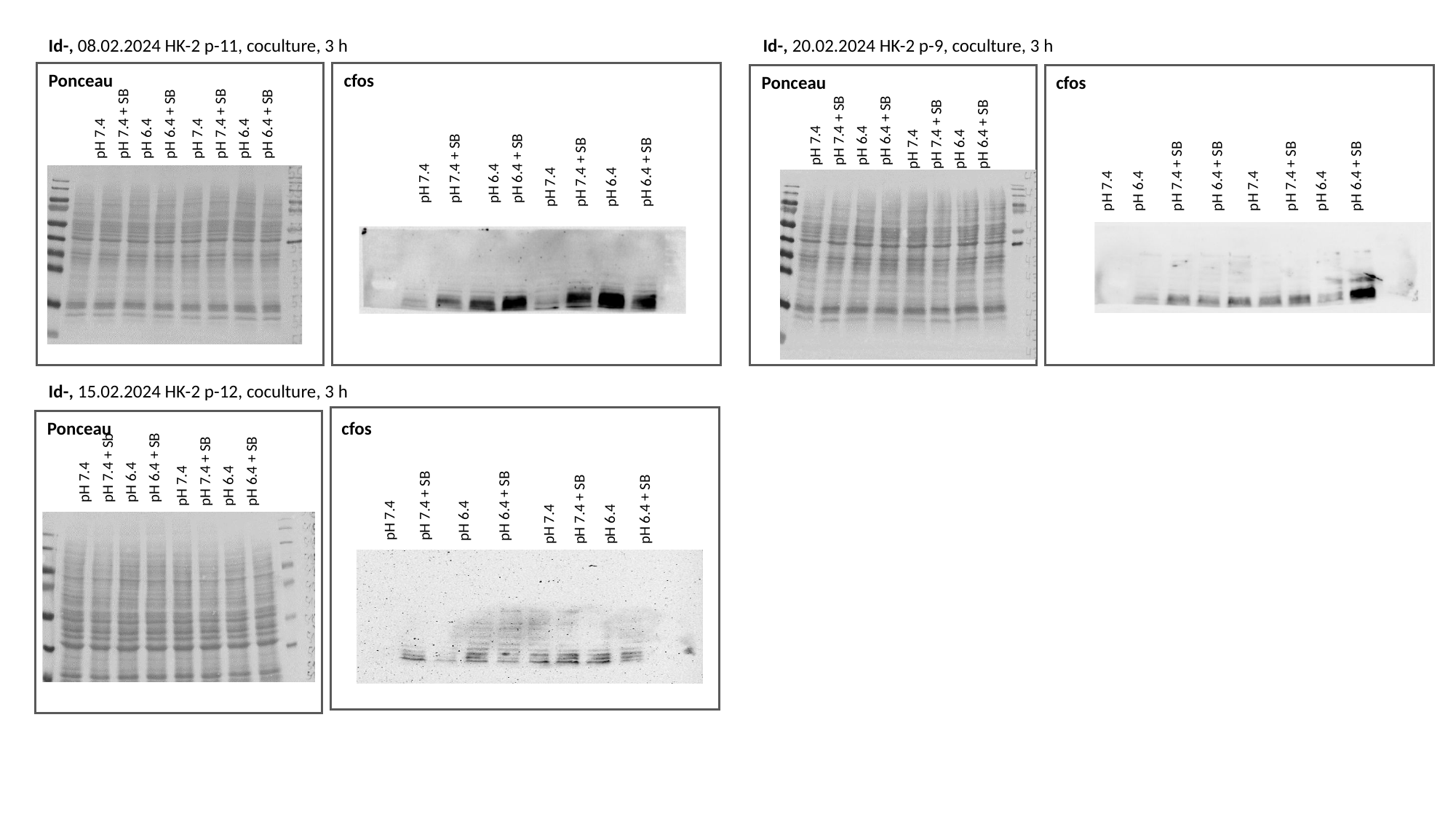

Id-, 08.02.2024 HK-2 p-11, coculture, 3 h
Id-, 20.02.2024 HK-2 p-9, coculture, 3 h
cfos
Ponceau
Ponceau
cfos
pH 7.4 + SB
pH 7.4 + SB
pH 6.4 + SB
pH 6.4 + SB
pH 7.4 + SB
pH 7.4 + SB
pH 6.4 + SB
pH 6.4
pH 6.4
pH 6.4 + SB
pH 6.4
pH 7.4
pH 7.4
pH 6.4
pH 7.4
pH 7.4
pH 7.4 + SB
pH 7.4 + SB
pH 6.4 + SB
pH 7.4 + SB
pH 7.4 + SB
pH 6.4 + SB
pH 6.4 + SB
pH 6.4 + SB
pH 6.4
pH 6.4
pH 6.4
pH 6.4
pH 7.4
pH 7.4
pH 7.4
pH 7.4
Id-, 15.02.2024 HK-2 p-12, coculture, 3 h
Ponceau
cfos
pH 7.4 + Sb
pH 7.4 + SB
pH 6.4 + SB
pH 6.4 + SB
pH 6.4
pH 6.4
pH 7.4
pH 7.4
pH 7.4 + SB
pH 7.4 + SB
pH 6.4 + SB
pH 6.4 + SB
pH 6.4
pH 6.4
pH 7.4
pH 7.4

## Slide 113
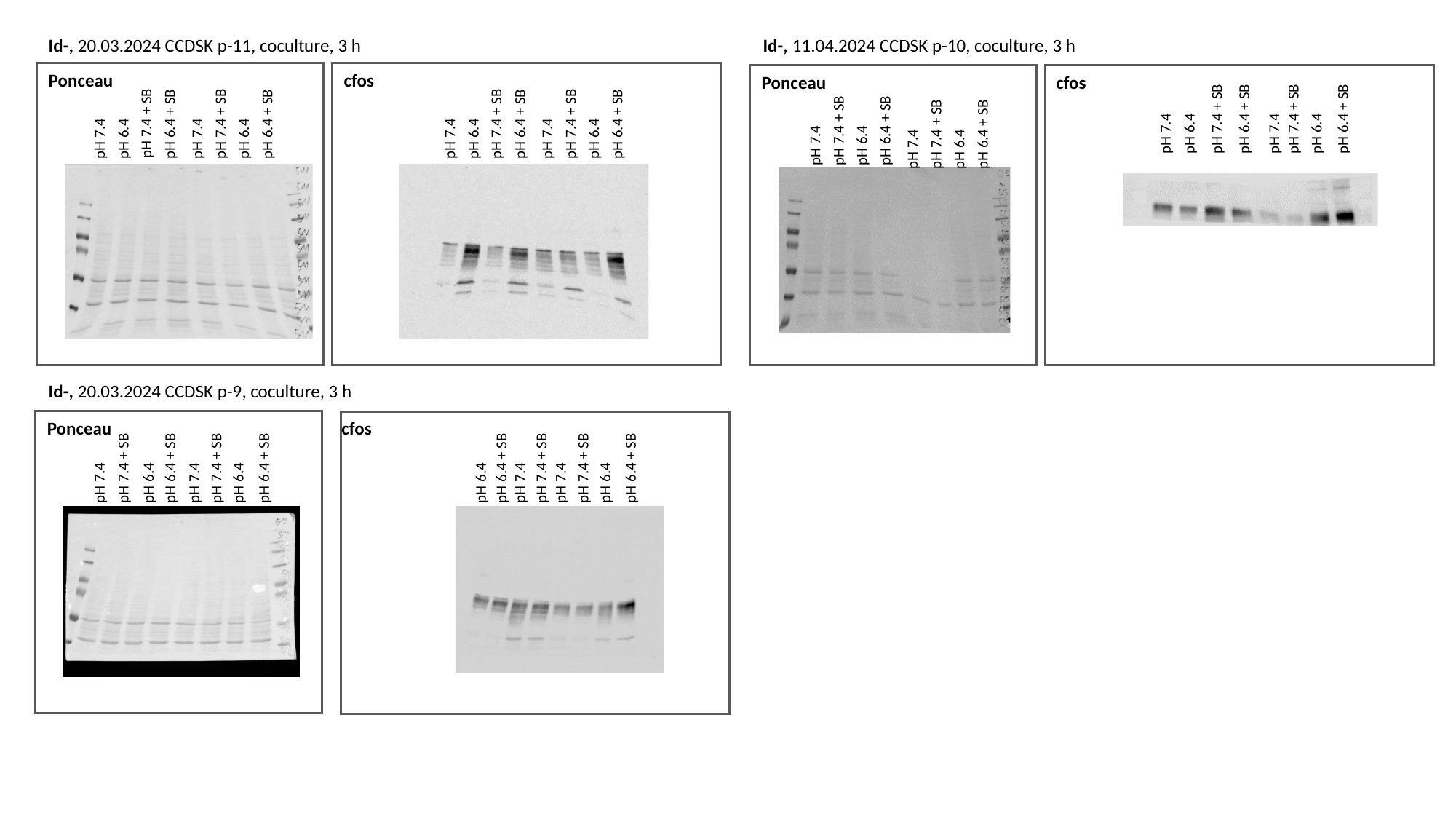

Id-, 20.03.2024 CCDSK p-11, coculture, 3 h
Id-, 11.04.2024 CCDSK p-10, coculture, 3 h
cfos
Ponceau
Ponceau
cfos
pH 7.4 + SB
pH 7.4 + SB
pH 7.4 + SB
pH 7.4 + SB
pH 6.4 + SB
pH 6.4 + SB
pH 7.4 + SB
pH 7.4 + SB
pH 6.4 + SB
pH 6.4 + SB
pH 6.4 + SB
pH 6.4 + SB
pH 7.4 + SB
pH 7.4 + SB
pH 6.4
pH 6.4
pH 6.4 + SB
pH 6.4
pH 6.4
pH 6.4
pH 6.4
pH 6.4 + SB
pH 7.4
pH 7.4
pH 6.4
pH 7.4
pH 7.4
pH 7.4
pH 7.4
pH 6.4
pH 7.4
pH 7.4
Id-, 20.03.2024 CCDSK p-9, coculture, 3 h
Ponceau
cfos
pH 7.4 + SB
pH 7.4 + SB
pH 7.4 + SB
pH 7.4 + SB
pH 6.4 + SB
pH 6.4 + SB
pH 6.4 + SB
pH 6.4 + SB
pH 6.4
pH 6.4
pH 6.4
pH 6.4
pH 7.4
pH 7.4
pH 7.4
pH 7.4

## Slide 114
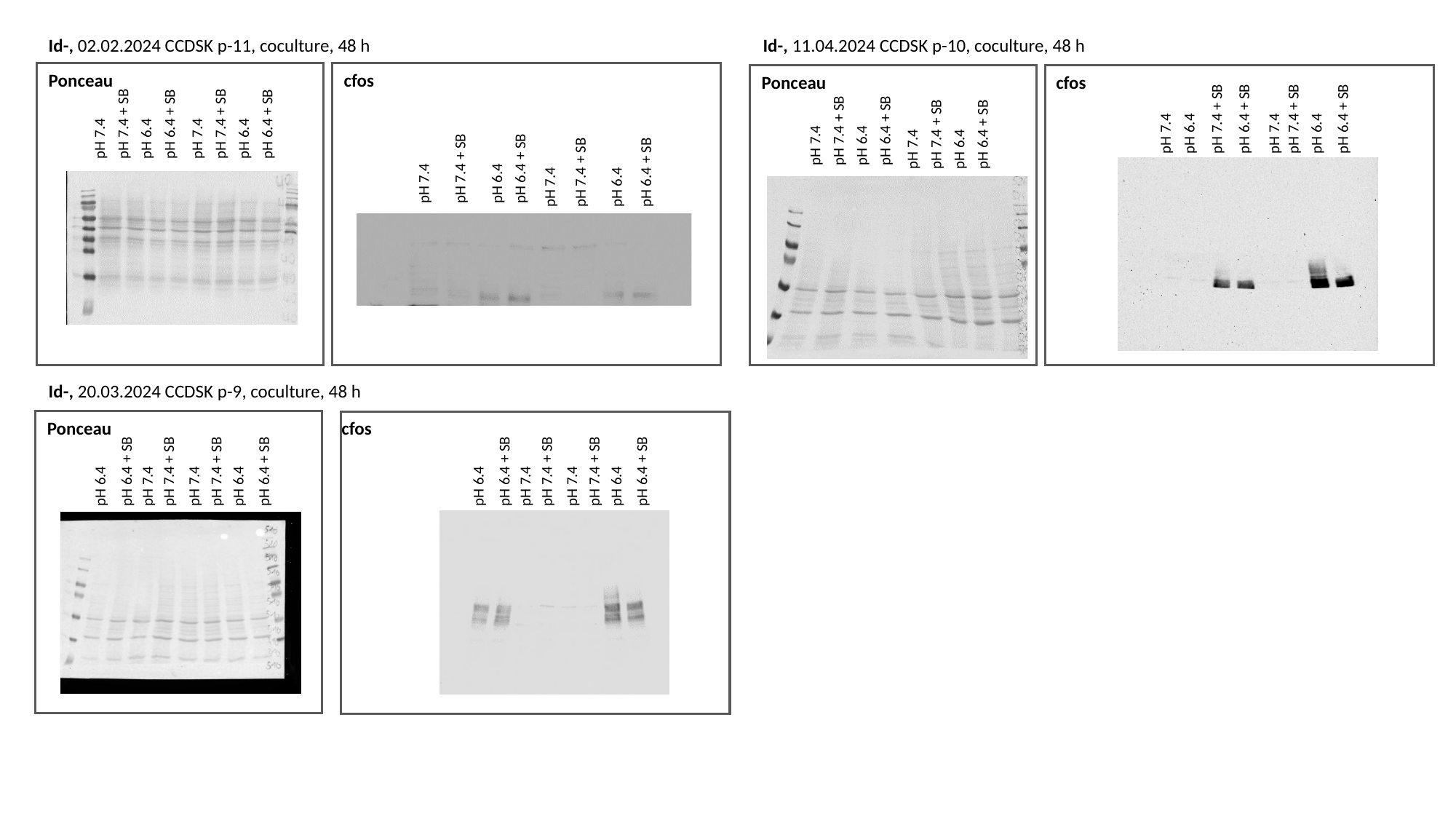

Id-, 02.02.2024 CCDSK p-11, coculture, 48 h
Id-, 11.04.2024 CCDSK p-10, coculture, 48 h
cfos
Ponceau
Ponceau
cfos
pH 7.4 + SB
pH 7.4 + SB
pH 7.4 + SB
pH 6.4 + SB
pH 6.4 + SB
pH 7.4 + SB
pH 6.4 + SB
pH 6.4 + SB
pH 7.4 + SB
pH 7.4 + SB
pH 6.4
pH 6.4
pH 6.4 + SB
pH 6.4
pH 6.4
pH 6.4 + SB
pH 7.4
pH 7.4
pH 6.4
pH 7.4
pH 7.4
pH 6.4
pH 7.4
pH 7.4
pH 7.4 + SB
pH 7.4 + SB
pH 6.4 + SB
pH 6.4 + SB
pH 6.4
pH 6.4
pH 7.4
pH 7.4
Id-, 20.03.2024 CCDSK p-9, coculture, 48 h
Ponceau
cfos
pH 7.4 + SB
pH 7.4 + SB
pH 7.4 + SB
pH 7.4 + SB
pH 6.4 + SB
pH 6.4 + SB
pH 6.4 + SB
pH 6.4 + SB
pH 6.4
pH 6.4
pH 6.4
pH 6.4
pH 7.4
pH 7.4
pH 7.4
pH 7.4

## Slide 115
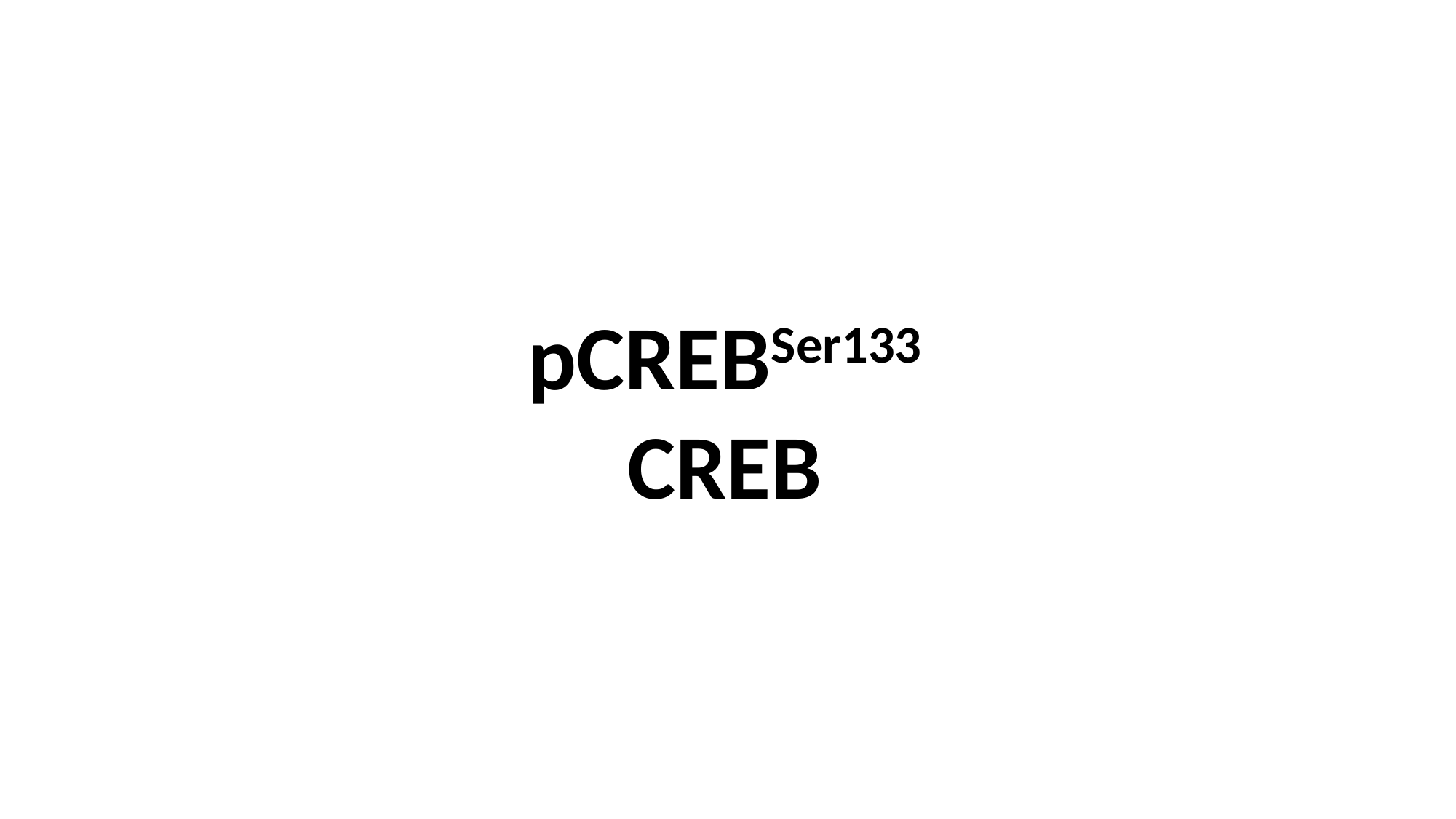

pCREBSer133
CREB

## Slide 116
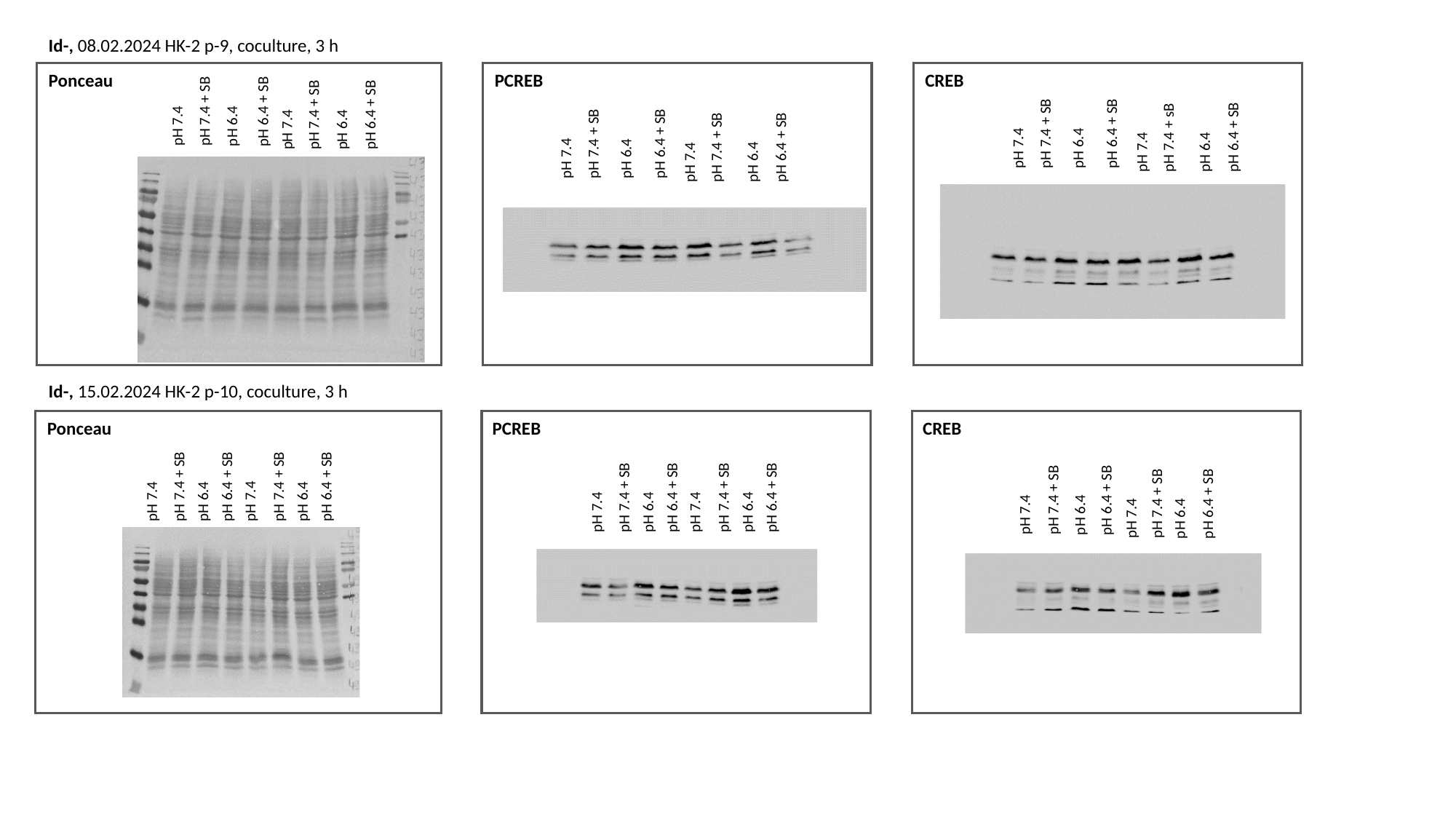

Id-, 08.02.2024 HK-2 p-9, coculture, 3 h
PCREB
CREB
Ponceau
pH 7.4 + SB
pH 7.4 + SB
pH 6.4 + SB
pH 6.4 + SB
pH 6.4
pH 6.4
pH 7.4 + SB
pH 7.4
pH 7.4 + sB
pH 7.4
pH 6.4 + SB
pH 6.4 + SB
pH 7.4 + SB
pH 7.4 + SB
pH 6.4
pH 6.4 + SB
pH 6.4
pH 6.4 + SB
pH 7.4
pH 6.4
pH 7.4
pH 6.4
pH 7.4
pH 7.4
Id-, 15.02.2024 HK-2 p-10, coculture, 3 h
Ponceau
PCREB
CREB
pH 7.4 + SB
pH 7.4 + SB
pH 6.4 + SB
pH 6.4 + SB
pH 7.4 + SB
pH 7.4 + SB
pH 7.4 + SB
pH 6.4
pH 6.4
pH 7.4 + SB
pH 6.4 + SB
pH 6.4 + SB
pH 6.4 + SB
pH 6.4 + SB
pH 7.4
pH 7.4
pH 6.4
pH 6.4
pH 6.4
pH 6.4
pH 7.4
pH 7.4
pH 7.4
pH 7.4

## Slide 117
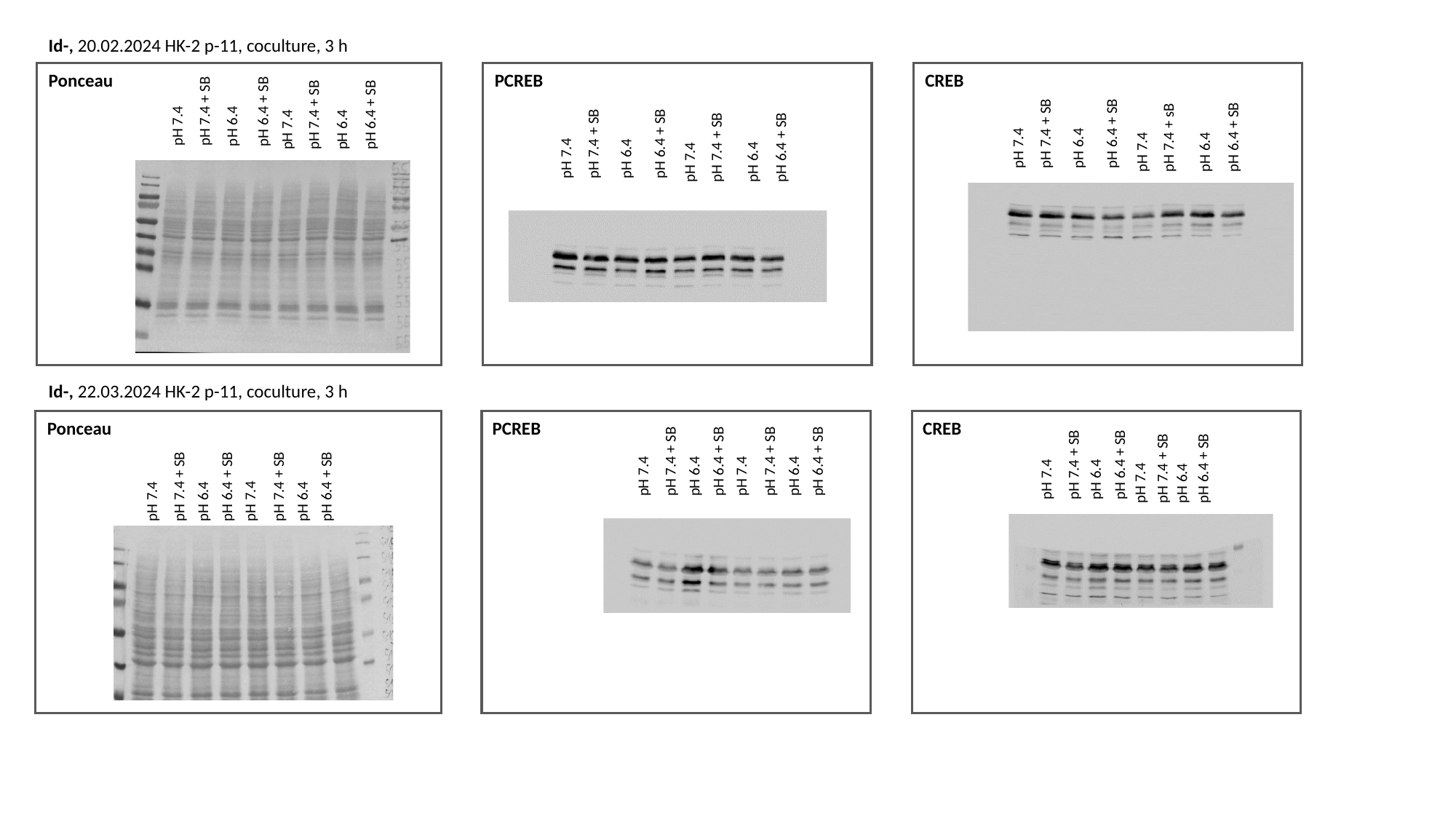

Id-, 20.02.2024 HK-2 p-11, coculture, 3 h
PCREB
CREB
Ponceau
pH 7.4 + SB
pH 7.4 + SB
pH 6.4 + SB
pH 6.4 + SB
pH 6.4
pH 6.4
pH 7.4 + SB
pH 7.4
pH 7.4 + sB
pH 7.4
pH 6.4 + SB
pH 6.4 + SB
pH 7.4 + SB
pH 7.4 + SB
pH 6.4
pH 6.4 + SB
pH 6.4
pH 6.4 + SB
pH 7.4
pH 6.4
pH 7.4
pH 6.4
pH 7.4
pH 7.4
Id-, 22.03.2024 HK-2 p-11, coculture, 3 h
Ponceau
PCREB
CREB
pH 7.4 + SB
pH 7.4 + SB
pH 7.4 + SB
pH 6.4 + SB
pH 6.4 + SB
pH 7.4 + SB
pH 6.4 + SB
pH 6.4 + SB
pH 6.4
pH 6.4
pH 6.4
pH 6.4
pH 7.4
pH 7.4
pH 7.4 + SB
pH 7.4 + SB
pH 7.4
pH 7.4
pH 6.4 + SB
pH 6.4 + SB
pH 6.4
pH 6.4
pH 7.4
pH 7.4

## Slide 118
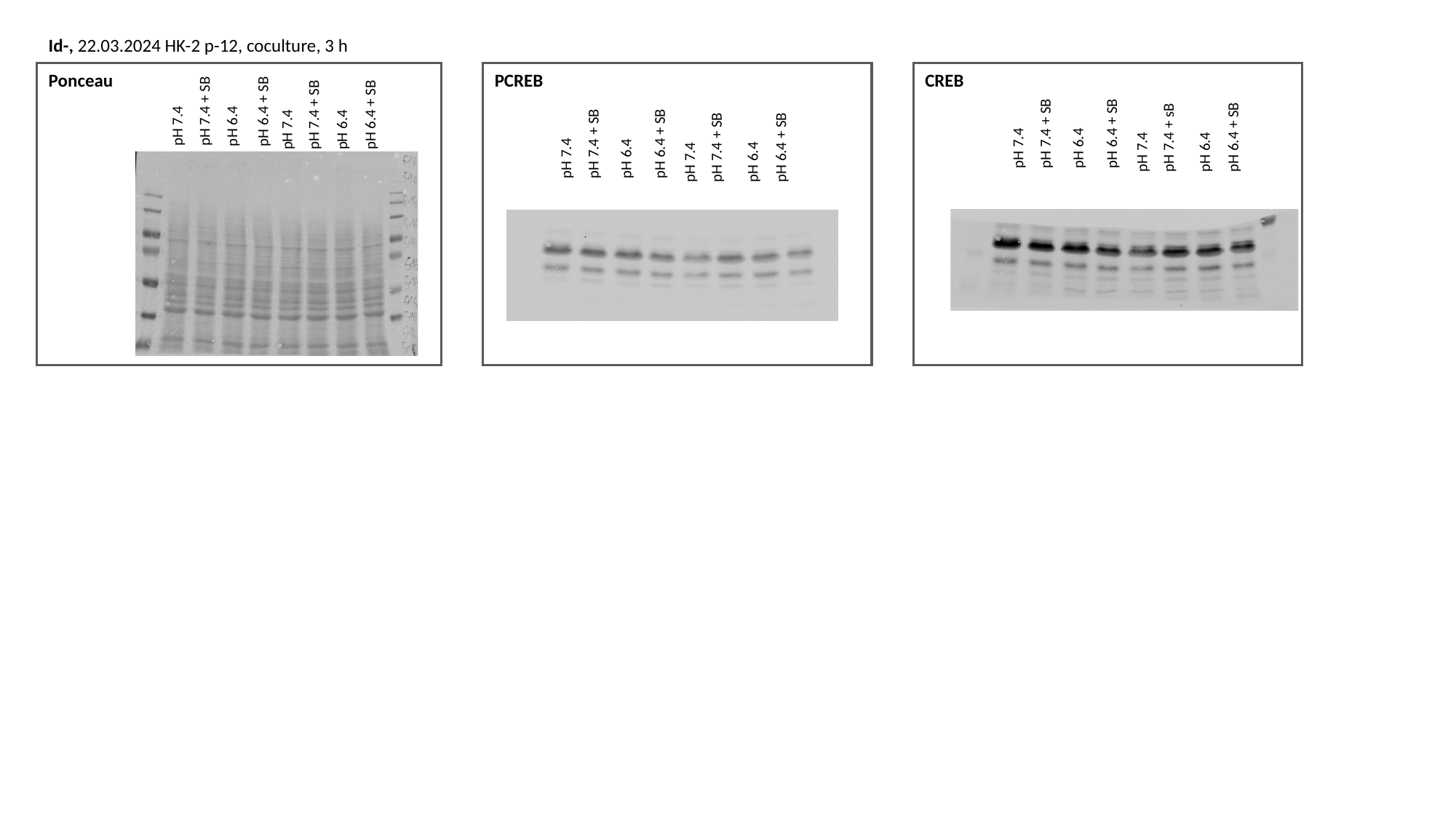

Id-, 22.03.2024 HK-2 p-12, coculture, 3 h
PCREB
CREB
Ponceau
pH 7.4 + SB
pH 7.4 + SB
pH 6.4 + SB
pH 6.4 + SB
pH 6.4
pH 6.4
pH 7.4 + SB
pH 7.4
pH 7.4 + sB
pH 7.4
pH 6.4 + SB
pH 6.4 + SB
pH 7.4 + SB
pH 7.4 + SB
pH 6.4
pH 6.4 + SB
pH 6.4
pH 6.4 + SB
pH 7.4
pH 6.4
pH 7.4
pH 6.4
pH 7.4
pH 7.4

## Slide 119
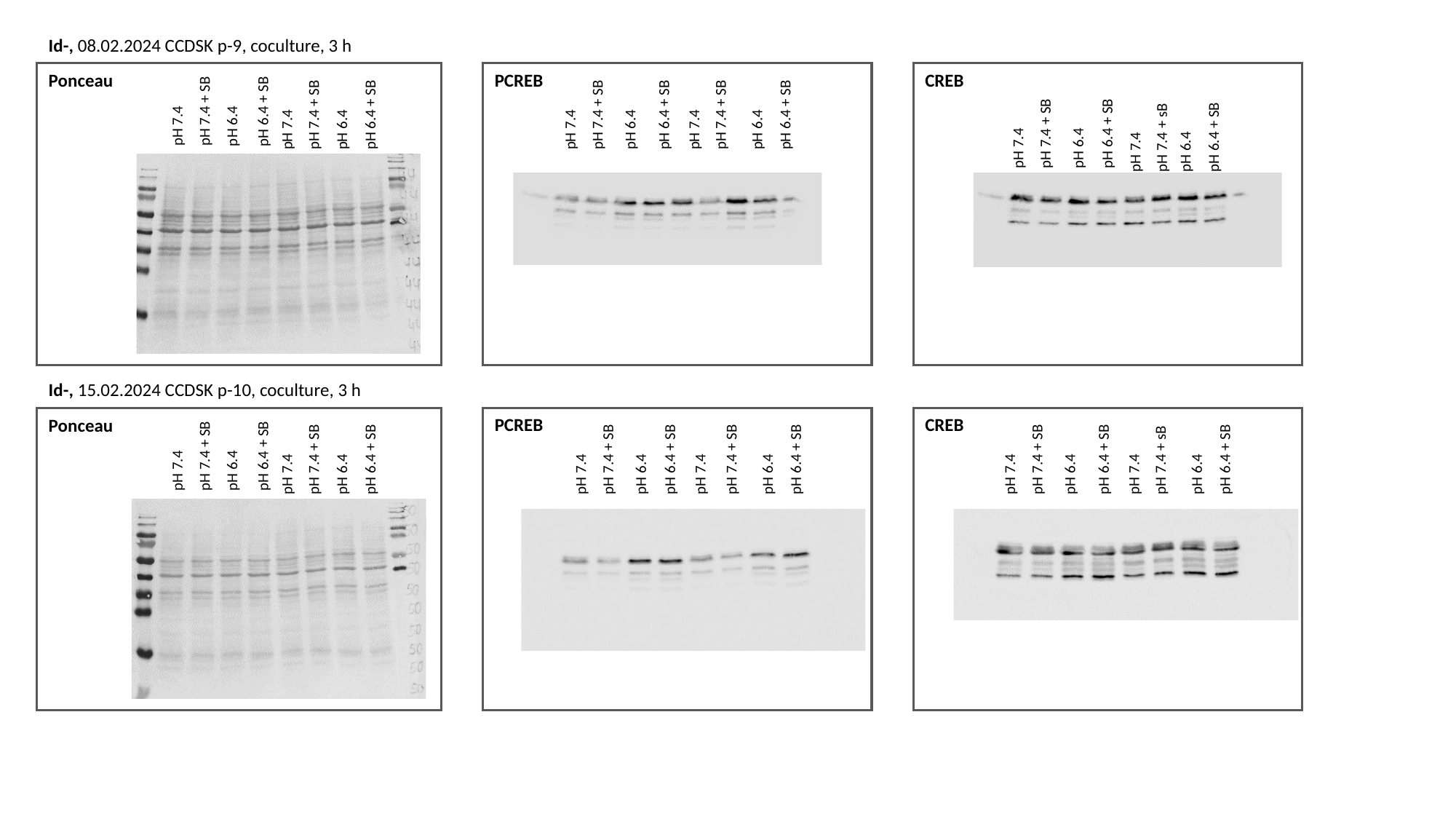

Id-, 08.02.2024 CCDSK p-9, coculture, 3 h
PCREB
CREB
Ponceau
pH 7.4 + SB
pH 7.4 + SB
pH 7.4 + SB
pH 7.4 + SB
pH 6.4 + SB
pH 6.4 + SB
pH 6.4 + SB
pH 6.4 + SB
pH 6.4
pH 6.4
pH 6.4
pH 6.4
pH 7.4 + SB
pH 7.4
pH 7.4 + sB
pH 7.4
pH 7.4
pH 7.4
pH 6.4 + SB
pH 6.4 + SB
pH 6.4
pH 6.4
pH 7.4
pH 7.4
Id-, 15.02.2024 CCDSK p-10, coculture, 3 h
PCREB
CREB
Ponceau
pH 7.4 + SB
pH 7.4 + SB
pH 7.4 + SB
pH 7.4 + sB
pH 7.4 + SB
pH 7.4 + SB
pH 6.4 + SB
pH 6.4 + SB
pH 6.4 + SB
pH 6.4 + SB
pH 6.4 + SB
pH 6.4 + SB
pH 6.4
pH 6.4
pH 6.4
pH 6.4
pH 6.4
pH 6.4
pH 7.4
pH 7.4
pH 7.4
pH 7.4
pH 7.4
pH 7.4

## Slide 120
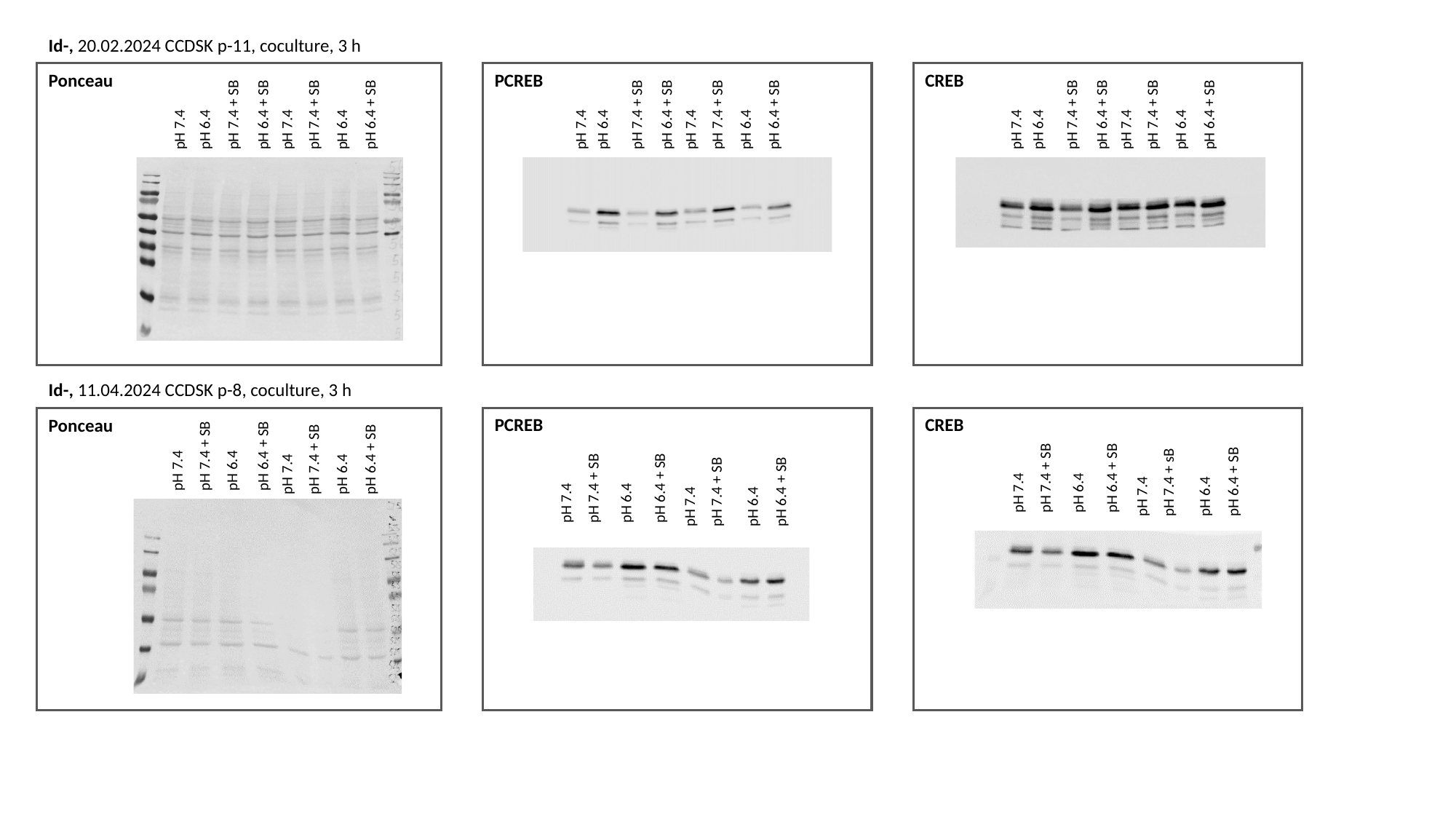

Id-, 20.02.2024 CCDSK p-11, coculture, 3 h
PCREB
CREB
Ponceau
pH 7.4 + SB
pH 7.4 + SB
pH 7.4 + SB
pH 7.4 + SB
pH 7.4 + SB
pH 7.4 + SB
pH 6.4 + SB
pH 6.4 + SB
pH 6.4 + SB
pH 6.4 + SB
pH 6.4 + SB
pH 6.4 + SB
pH 6.4
pH 6.4
pH 6.4
pH 6.4
pH 6.4
pH 6.4
pH 7.4
pH 7.4
pH 7.4
pH 7.4
pH 7.4
pH 7.4
Id-, 11.04.2024 CCDSK p-8, coculture, 3 h
PCREB
CREB
Ponceau
pH 7.4 + SB
pH 7.4 + SB
pH 6.4 + SB
pH 6.4 + SB
pH 6.4
pH 6.4
pH 7.4 + SB
pH 7.4
pH 7.4 + sB
pH 7.4
pH 6.4 + SB
pH 6.4 + SB
pH 7.4 + SB
pH 7.4 + SB
pH 6.4
pH 6.4 + SB
pH 6.4
pH 6.4 + SB
pH 7.4
pH 6.4
pH 7.4
pH 6.4
pH 7.4
pH 7.4

## Slide 121
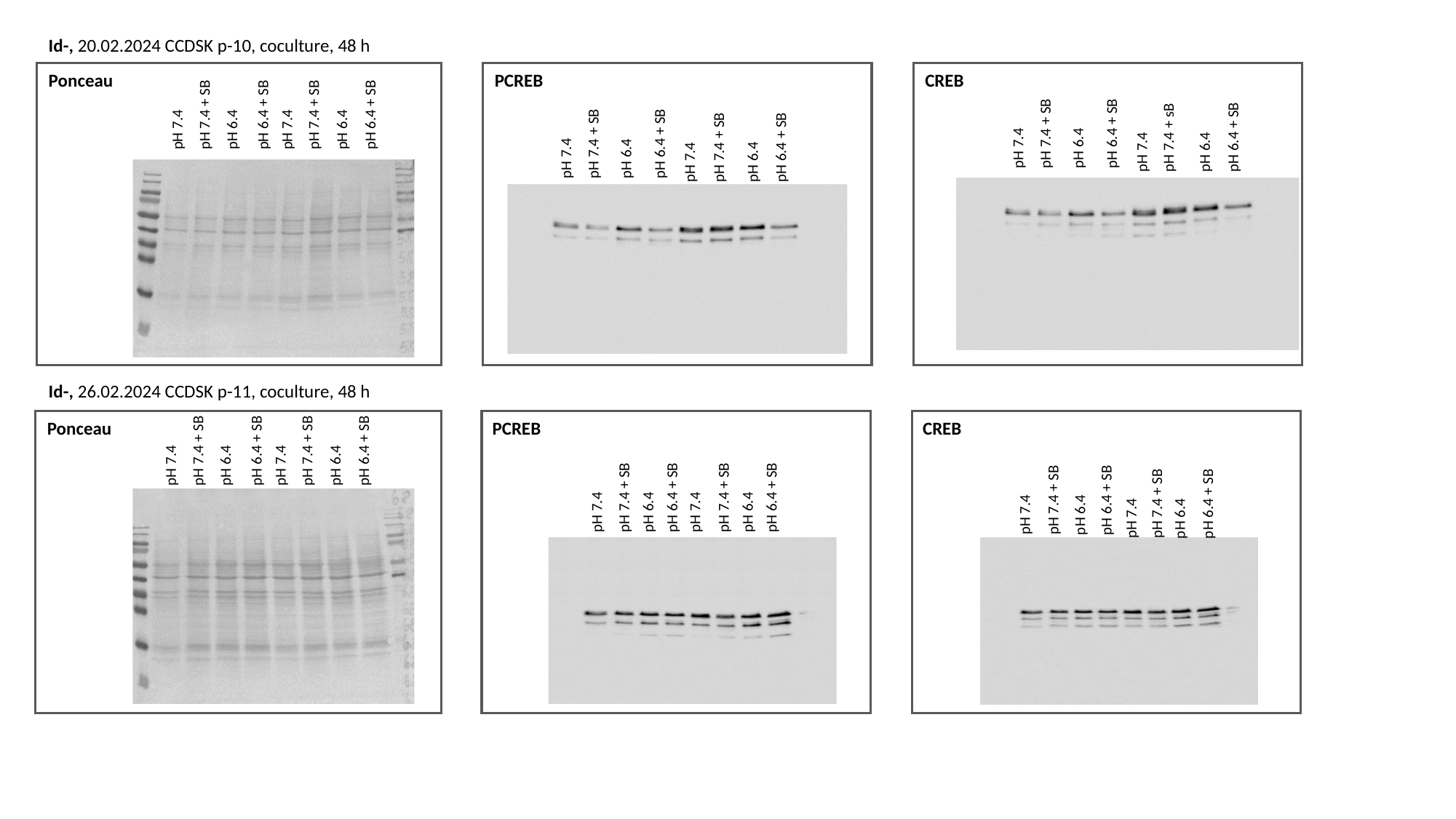

Id-, 20.02.2024 CCDSK p-10, coculture, 48 h
PCREB
CREB
Ponceau
pH 7.4 + SB
pH 7.4 + SB
pH 6.4 + SB
pH 6.4 + SB
pH 6.4
pH 6.4
pH 7.4 + SB
pH 7.4 + sB
pH 7.4
pH 7.4
pH 6.4 + SB
pH 6.4 + SB
pH 7.4 + SB
pH 7.4 + SB
pH 6.4
pH 6.4 + SB
pH 6.4
pH 6.4 + SB
pH 7.4
pH 6.4
pH 7.4
pH 6.4
pH 7.4
pH 7.4
Id-, 26.02.2024 CCDSK p-11, coculture, 48 h
Ponceau
PCREB
CREB
pH 7.4 + SB
pH 7.4 + SB
pH 6.4 + SB
pH 6.4 + SB
pH 6.4
pH 6.4
pH 7.4
pH 7.4
pH 7.4 + SB
pH 7.4 + SB
pH 7.4 + SB
pH 7.4 + SB
pH 6.4 + SB
pH 6.4 + SB
pH 6.4 + SB
pH 6.4 + SB
pH 6.4
pH 6.4
pH 6.4
pH 6.4
pH 7.4
pH 7.4
pH 7.4
pH 7.4

## Slide 122
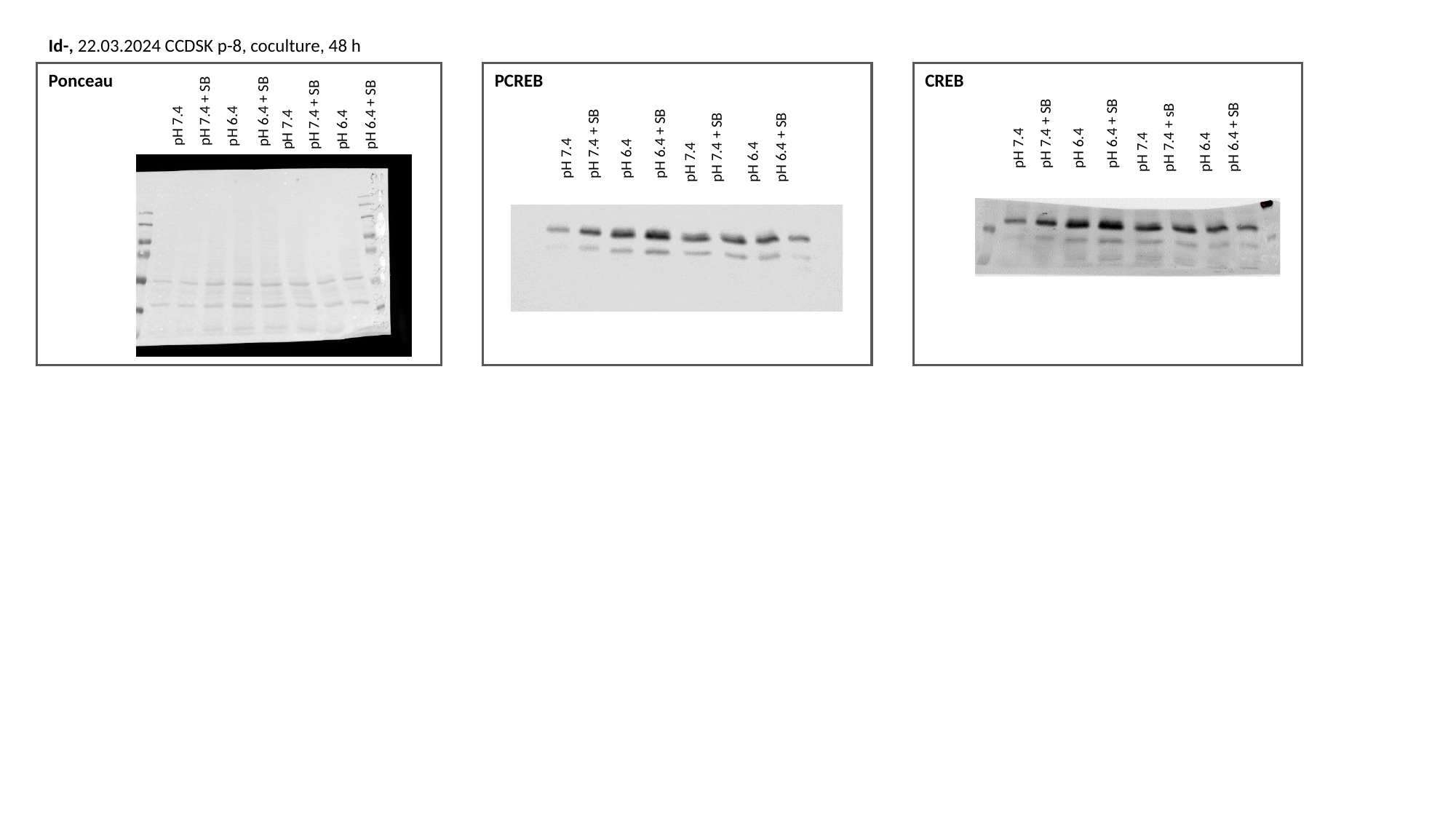

Id-, 22.03.2024 CCDSK p-8, coculture, 48 h
PCREB
CREB
Ponceau
pH 7.4 + SB
pH 7.4 + SB
pH 6.4 + SB
pH 6.4 + SB
pH 6.4
pH 6.4
pH 7.4 + SB
pH 7.4
pH 7.4 + sB
pH 7.4
pH 6.4 + SB
pH 6.4 + SB
pH 7.4 + SB
pH 7.4 + SB
pH 6.4
pH 6.4 + SB
pH 6.4
pH 6.4 + SB
pH 7.4
pH 6.4
pH 7.4
pH 6.4
pH 7.4
pH 7.4

## Slide 123
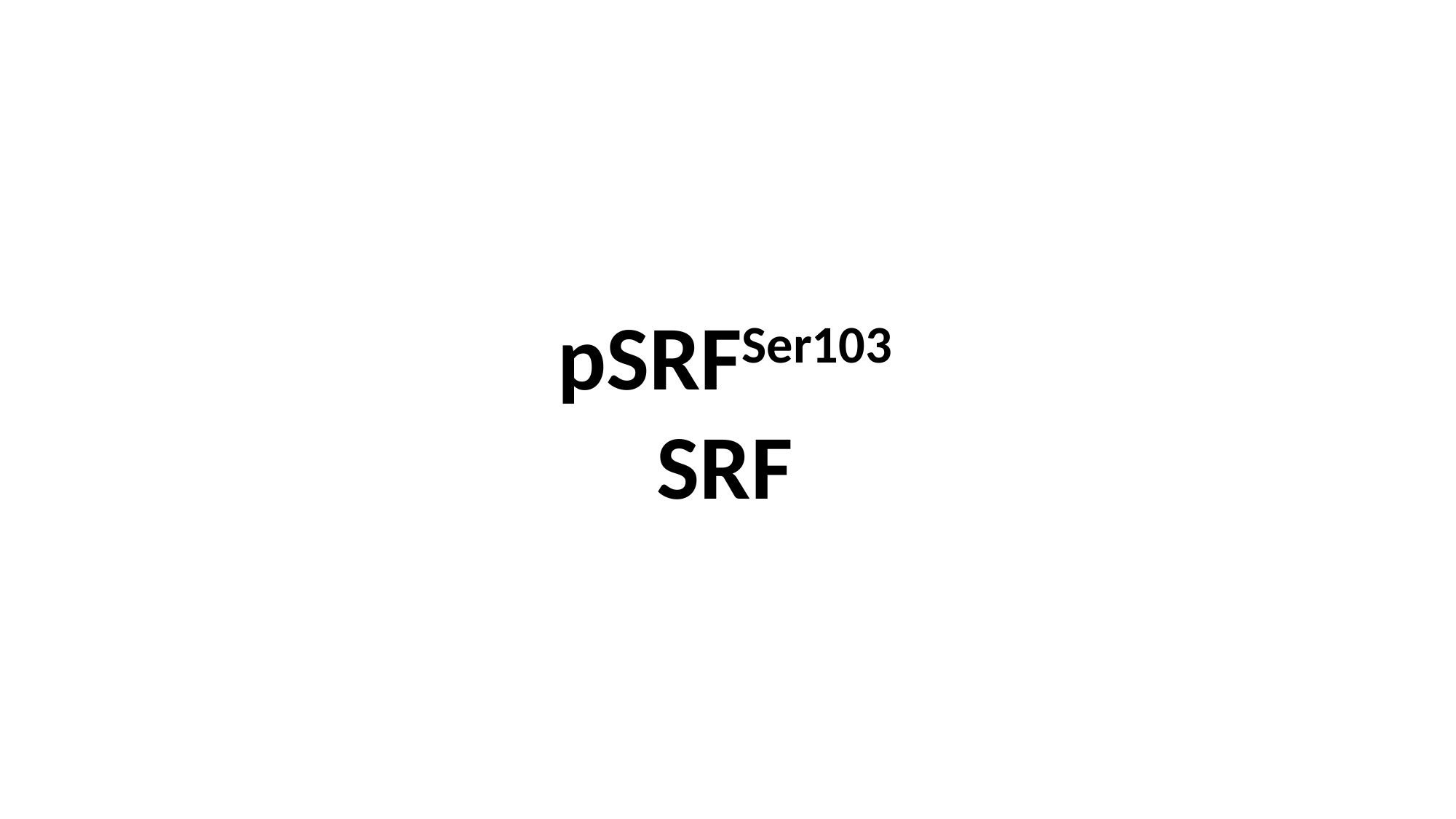

pSRFSer103
SRF

## Slide 124
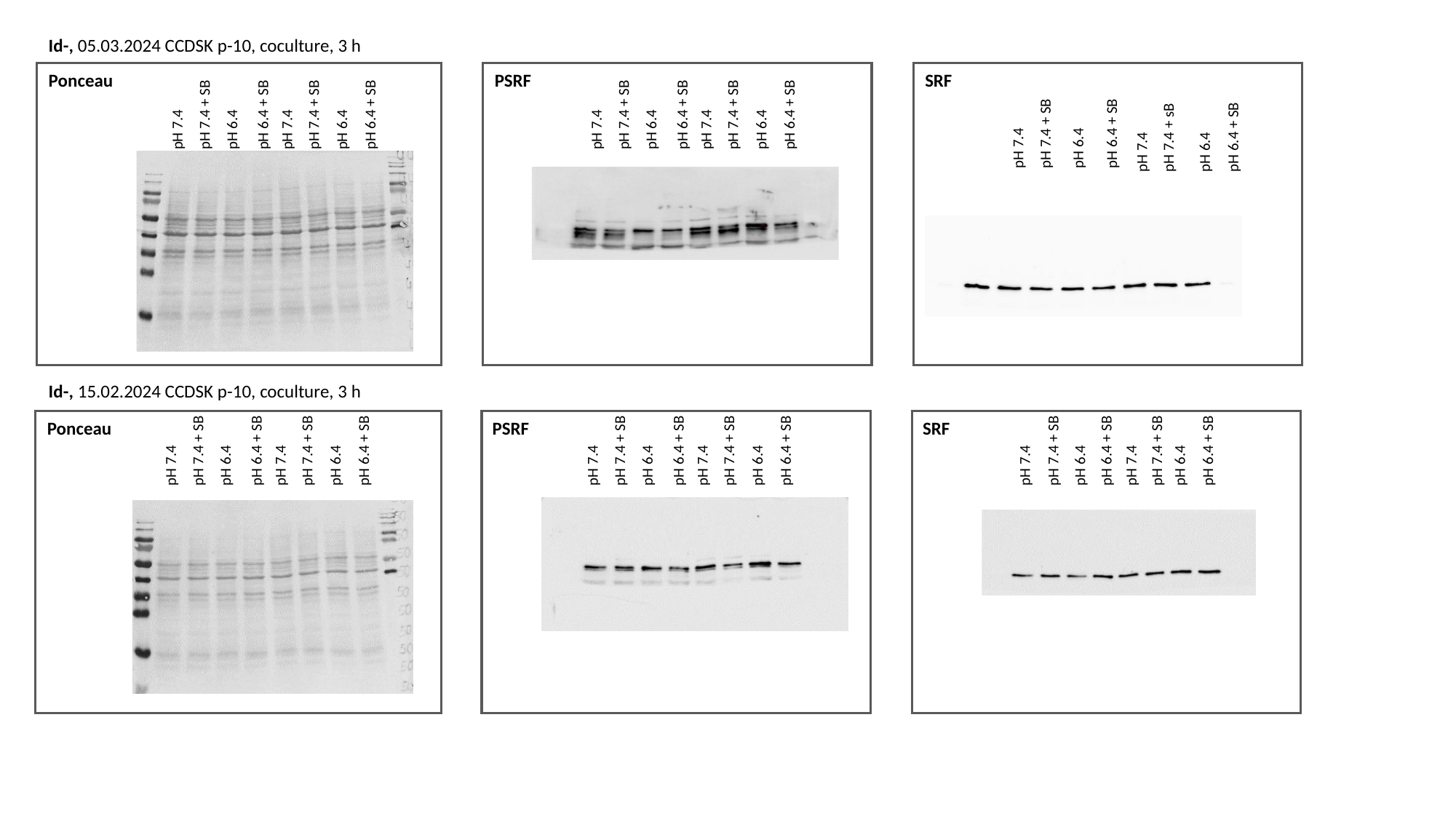

Id-, 05.03.2024 CCDSK p-10, coculture, 3 h
PSRF
SRF
Ponceau
pH 7.4 + SB
pH 7.4 + SB
pH 7.4 + SB
pH 7.4 + SB
pH 6.4 + SB
pH 6.4 + SB
pH 6.4 + SB
pH 6.4 + SB
pH 6.4
pH 6.4
pH 6.4
pH 6.4
pH 7.4 + SB
pH 7.4 + sB
pH 7.4
pH 7.4
pH 7.4
pH 7.4
pH 6.4 + SB
pH 6.4 + SB
pH 6.4
pH 6.4
pH 7.4
pH 7.4
Id-, 15.02.2024 CCDSK p-10, coculture, 3 h
Ponceau
PSRF
SRF
pH 7.4 + SB
pH 7.4 + SB
pH 7.4 + SB
pH 7.4 + SB
pH 7.4 + SB
pH 7.4 + SB
pH 6.4 + SB
pH 6.4 + SB
pH 6.4 + SB
pH 6.4 + SB
pH 6.4 + SB
pH 6.4 + SB
pH 6.4
pH 6.4
pH 6.4
pH 6.4
pH 6.4
pH 6.4
pH 7.4
pH 7.4
pH 7.4
pH 7.4
pH 7.4
pH 7.4

## Slide 125
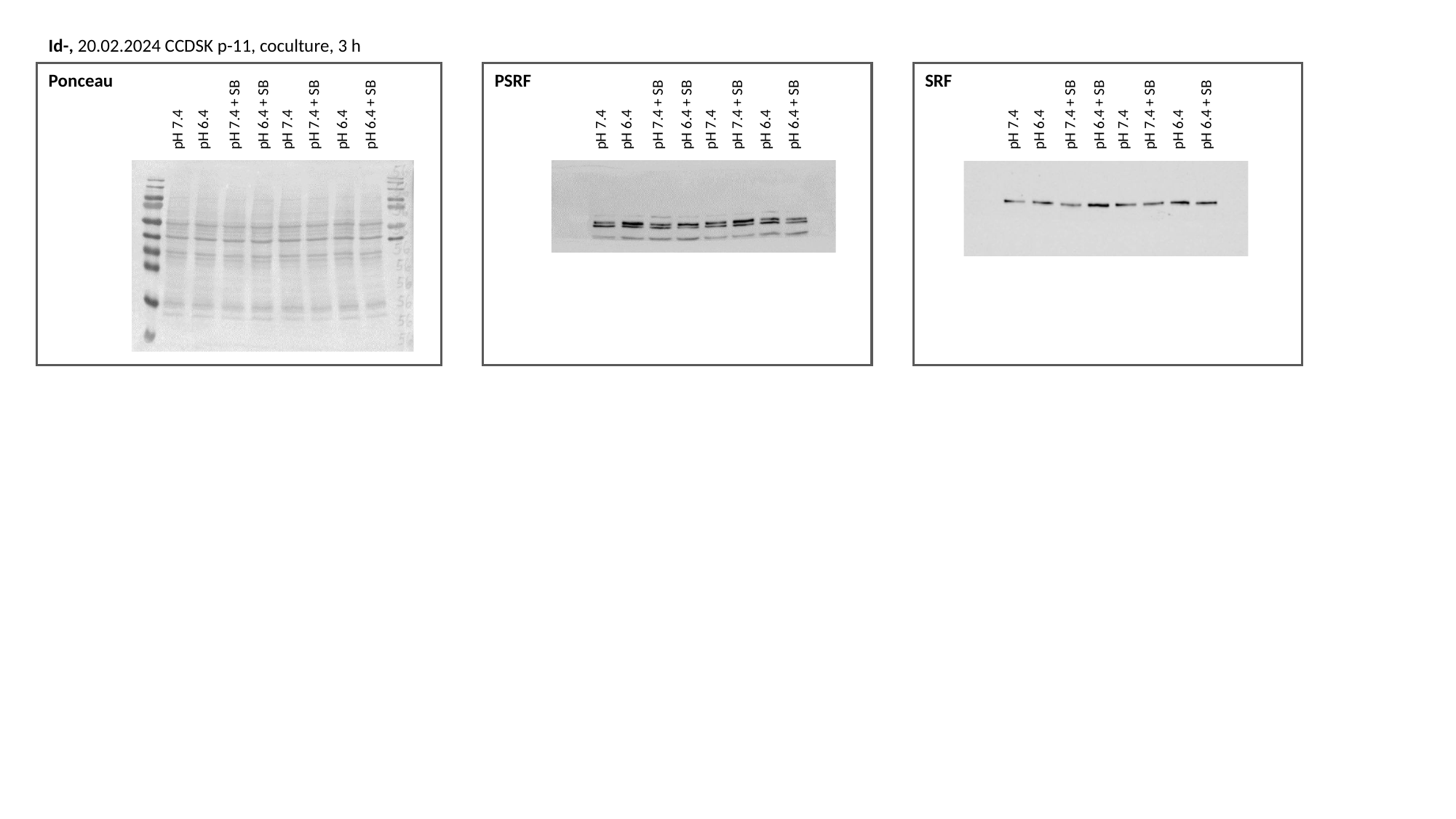

Id-, 20.02.2024 CCDSK p-11, coculture, 3 h
PSRF
SRF
Ponceau
pH 7.4 + SB
pH 7.4 + SB
pH 7.4 + SB
pH 7.4 + SB
pH 7.4 + SB
pH 7.4 + SB
pH 6.4 + SB
pH 6.4 + SB
pH 6.4 + SB
pH 6.4 + SB
pH 6.4 + SB
pH 6.4 + SB
pH 6.4
pH 6.4
pH 6.4
pH 6.4
pH 6.4
pH 6.4
pH 7.4
pH 7.4
pH 7.4
pH 7.4
pH 7.4
pH 7.4

## Slide 126
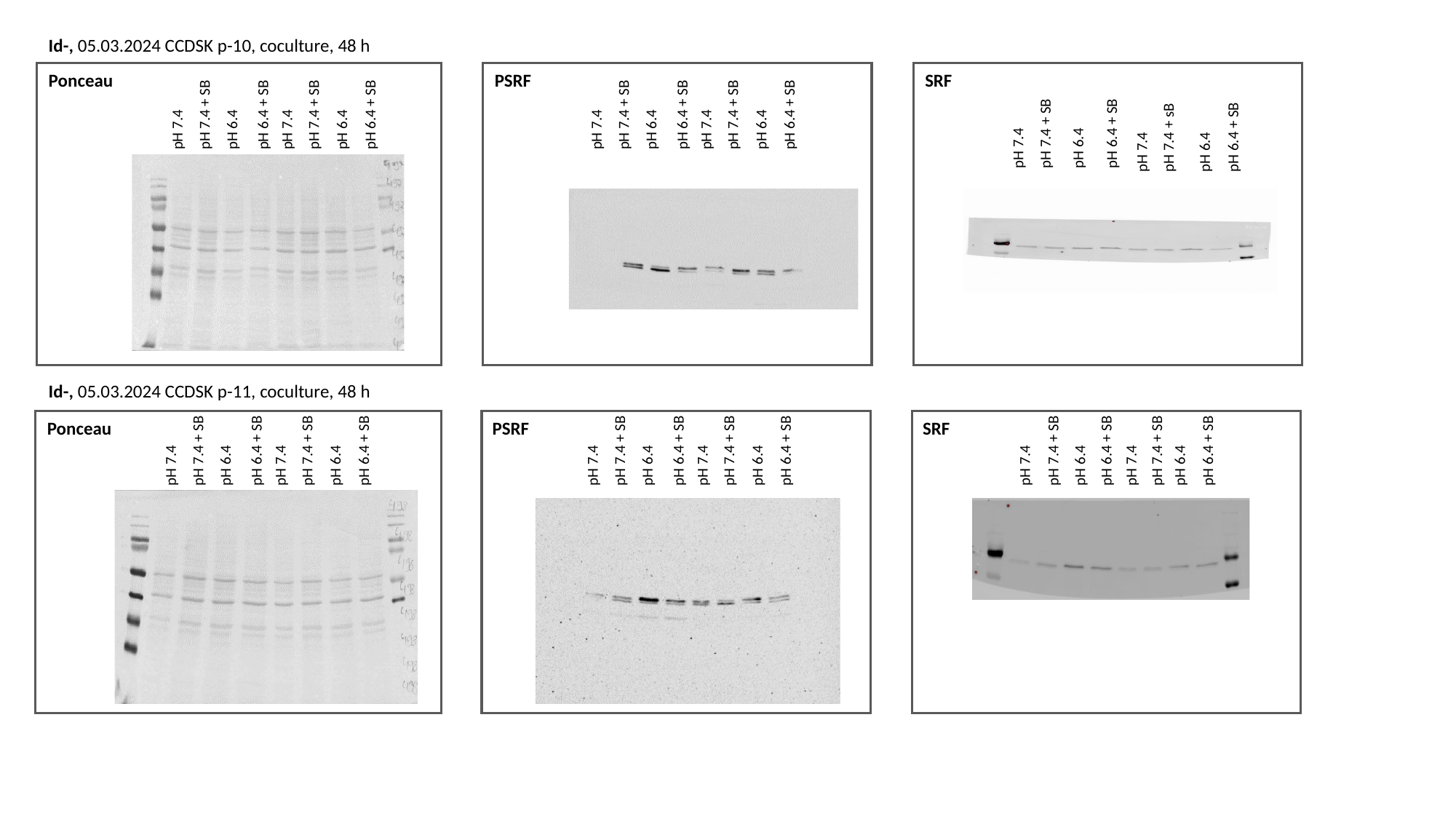

Id-, 05.03.2024 CCDSK p-10, coculture, 48 h
PSRF
SRF
Ponceau
pH 7.4 + SB
pH 7.4 + SB
pH 7.4 + SB
pH 7.4 + SB
pH 6.4 + SB
pH 6.4 + SB
pH 6.4 + SB
pH 6.4 + SB
pH 6.4
pH 6.4
pH 6.4
pH 6.4
pH 7.4 + SB
pH 7.4 + sB
pH 7.4
pH 7.4
pH 7.4
pH 7.4
pH 6.4 + SB
pH 6.4 + SB
pH 6.4
pH 6.4
pH 7.4
pH 7.4
Id-, 05.03.2024 CCDSK p-11, coculture, 48 h
Ponceau
PSRF
SRF
pH 7.4 + SB
pH 7.4 + SB
pH 7.4 + SB
pH 7.4 + SB
pH 7.4 + SB
pH 7.4 + SB
pH 6.4 + SB
pH 6.4 + SB
pH 6.4 + SB
pH 6.4 + SB
pH 6.4 + SB
pH 6.4 + SB
pH 6.4
pH 6.4
pH 6.4
pH 6.4
pH 6.4
pH 6.4
pH 7.4
pH 7.4
pH 7.4
pH 7.4
pH 7.4
pH 7.4

## Slide 127
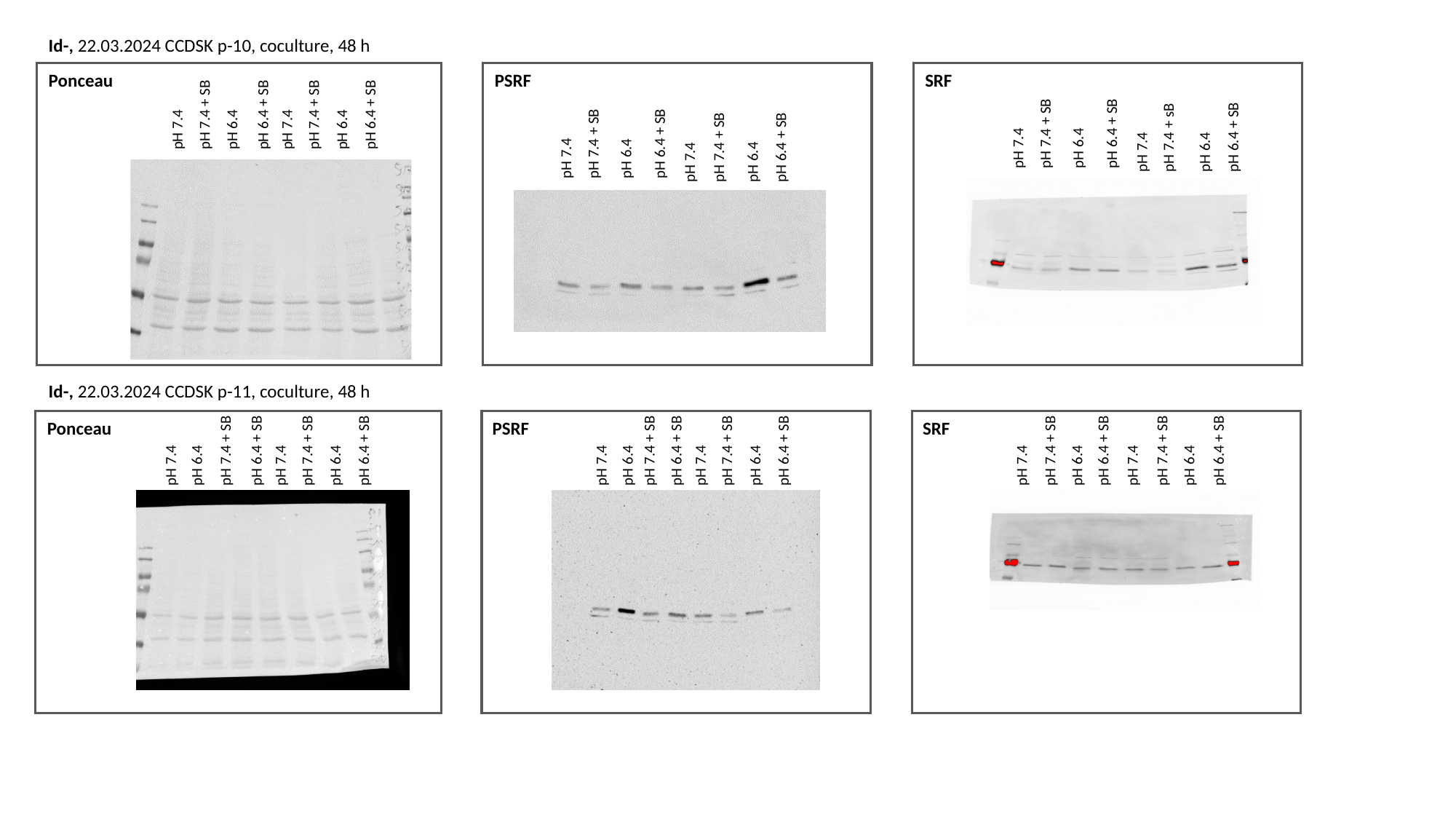

Id-, 22.03.2024 CCDSK p-10, coculture, 48 h
PSRF
SRF
Ponceau
pH 7.4 + SB
pH 7.4 + SB
pH 6.4 + SB
pH 6.4 + SB
pH 6.4
pH 6.4
pH 7.4 + SB
pH 7.4 + sB
pH 7.4
pH 7.4
pH 6.4 + SB
pH 6.4 + SB
pH 7.4 + SB
pH 7.4 + SB
pH 6.4
pH 6.4 + SB
pH 6.4
pH 6.4 + SB
pH 7.4
pH 6.4
pH 7.4
pH 6.4
pH 7.4
pH 7.4
Id-, 22.03.2024 CCDSK p-11, coculture, 48 h
Ponceau
PSRF
SRF
pH 7.4 + SB
pH 7.4 + SB
pH 7.4 + SB
pH 7.4 + SB
pH 7.4 + SB
pH 7.4 + SB
pH 6.4 + SB
pH 6.4 + SB
pH 6.4 + SB
pH 6.4 + SB
pH 6.4 + SB
pH 6.4 + SB
pH 6.4
pH 6.4
pH 6.4
pH 6.4
pH 6.4
pH 6.4
pH 7.4
pH 7.4
pH 7.4
pH 7.4
pH 7.4
pH 7.4
