# Supplementary material for: Acidosis-induced p38-kinase activation triggers an IL-6-mediated crosstalk of renal proximal tubule cells with fibroblasts leading to their inflammatory response
Source: Cell Commun Signal. 2025 Apr 11;23:180. doi: 10.1186/s12964-025-02180-5 (PMC11987431; doi:10.1186/s12964-025-02180-5)
Supplement: Supplementary file 2 — Supplementary Material 2 [file 12964_2025_2180_MOESM2_ESM.pdf]

Supplementary material table of content

Supplementary figure 1 (S1): Effect of acidic media on the secretion of IL-6 in monoculture.

Supplementary figure 2 (S2): Proportion of total CREB in the cytosol, nucleus and chromatin of HK-2 cells.

Supplementary figure 3 (S3): Significance of p38 activity for measured acidosis-effects in HK-2 and CCDSK cells in coculture.

Supplementary figure 4 (S4): Significance of IL-6 receptor for acidosis-induced effects in HK-2 cells.

Supplementary figure 5 (S5): Significance of IL-6 for acidosis-induced effects in CCDSK cells.

Supplementary table 1: Basal IL-6 concentration [pg/ml] in monoculture.

Supplementary table 2 List of used antibodies.

Supplementary table 3 List of buffer composition.

Supplementary table 4 Gene expression of CREB, cFos, IL-6, IL-6R, p38, COX-2 and SRF.

Methods are described in main text.

S1. Supplementary results

S1.1. IL-6 secretion in monoculture.

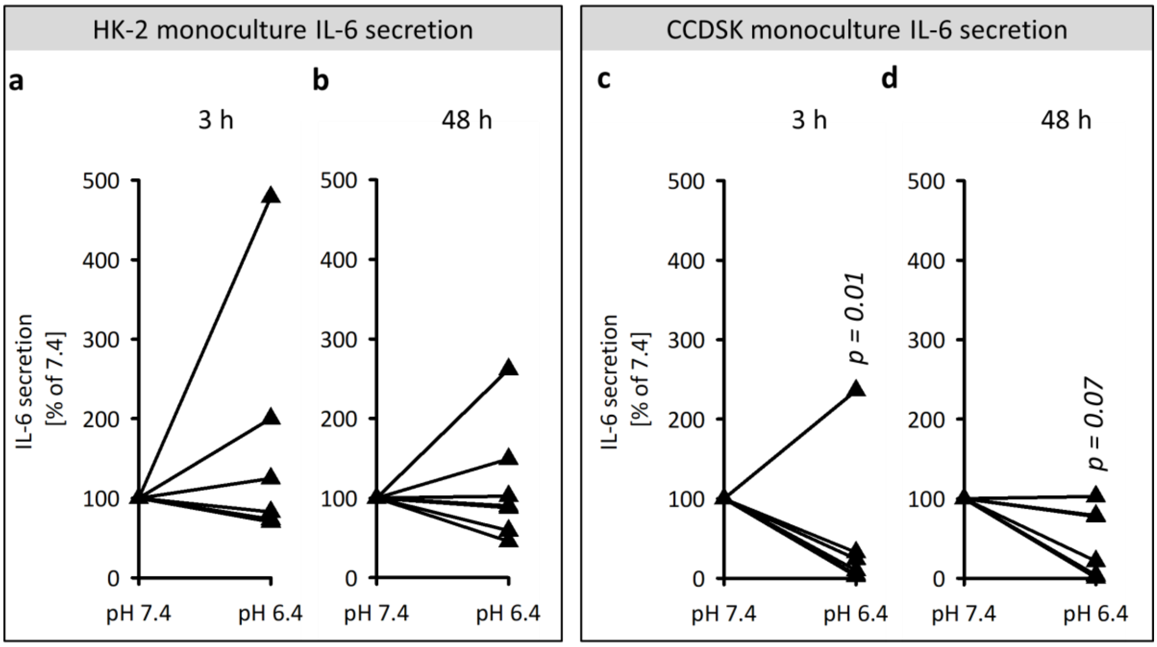

Supplementary figure 1 Effect of acidic media on the secretion of IL-6 in monoculture. Acidosis effect on IL-6 secretion after 3 h and 48 h in media from HK-2 (a, b), CCDSK (c, d). n = 7, significant changes compared to pH 7.4 = $p < 0.05$.

Figure S1 shows the impact of acidosis on the IL-6 secretion in HK-2 and CCDSK in monoculture, measured by ELISA. In contrast to results in coculture, acidosis caused no change in the IL-6 secretion in HK-2 cells (S1 a, b). In CCDSK cells, an acidic media led to a decrease of IL-6 secretion after 3 h and 48 h (S1 c, d). Supplementary table 1 shows that HK-2 cells in monoculture secrete between 0.46 and 16.3 pg/ml IL-6, while fibroblasts secrete between 0.05 and 0.11 pg/ml IL-6. The Kd value of the IL-6 receptor is 360 pg/ml (15 pM). Consequently, the receptor saturation with the measured IL-6 concentrations is 0.01-0.1%, making its activation and thus biological efficacy unlikely.

Supplementary table 1 Basal IL-6 concentration [pg/ml/µg] and secretion rate [pg/ml/h] in monoculture

basal IL-6 concentration [pg/ml/µg] median ± confidence interval			basal IL-6 secretion rate [pg/ml/h] median ± confidence interval	
	3 h	48 h	3 h	48 h
HK-2 mono	0.007 ± [0.1, 1.7]	0.142 ± [11.8, 20.5]	0.15 ± [0.04, 0.5]	0.3 ± [0.2, 0.4]
CCDSK mono	0.001 ± [0.001, 0.001]	0.003 ± [0.019, 0.001]	0.02 ± [0.01, 0.02]	0.002 ± [0.002, 0.008]

S1.2. Distribution of CREB to cell compartments in HK-2.

Figure S2 shows that acidosis had no impact on the distribution of total CREB to the cytosol, nucleus and chromatin after 48 h in HK-2 cells.

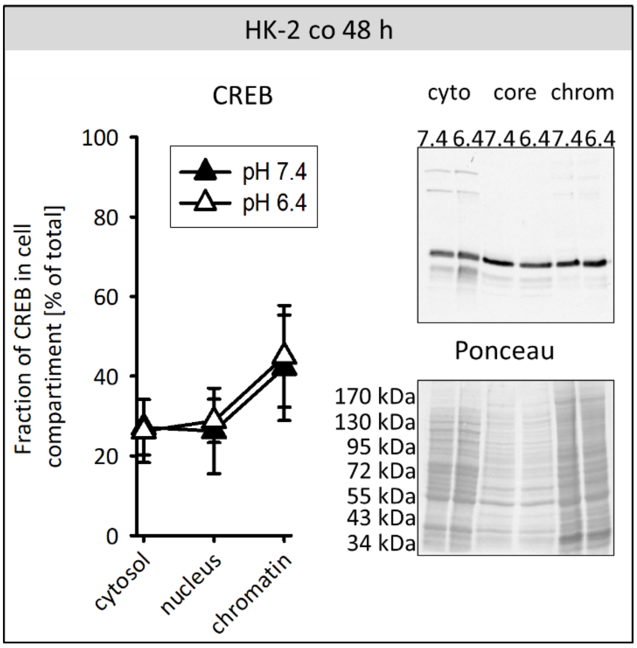

Supplementary figure 2 Impact of acidosis on proportion of total CREB in the cytosol, nucleus and chromatin of HK-2 cells. Effect of acidic or control media on the distribution of CREB in HK-2 cells in coculture after 48 h. n = 5, significant changes compared to pH 7.4 = $p < 0.05$.

S1.3. Significance of p38 activity for acidosis-effects.

Figure S3 shows that p38-activity had no impact on the acidosis-effect on total CREB expression in HK-2 cells after 3h (S3a). Moreover, p38-activity is necessary for the acidosis-induced increase of total CREB in CCDSK after 3 h but plays no role on the expression of total SRF (S3b-c). After 48 h in CCDSK cells, the p38-activity mediates the acidosis-induced increase of total SRF, but had no impact on total CREB expression (S3d-e).

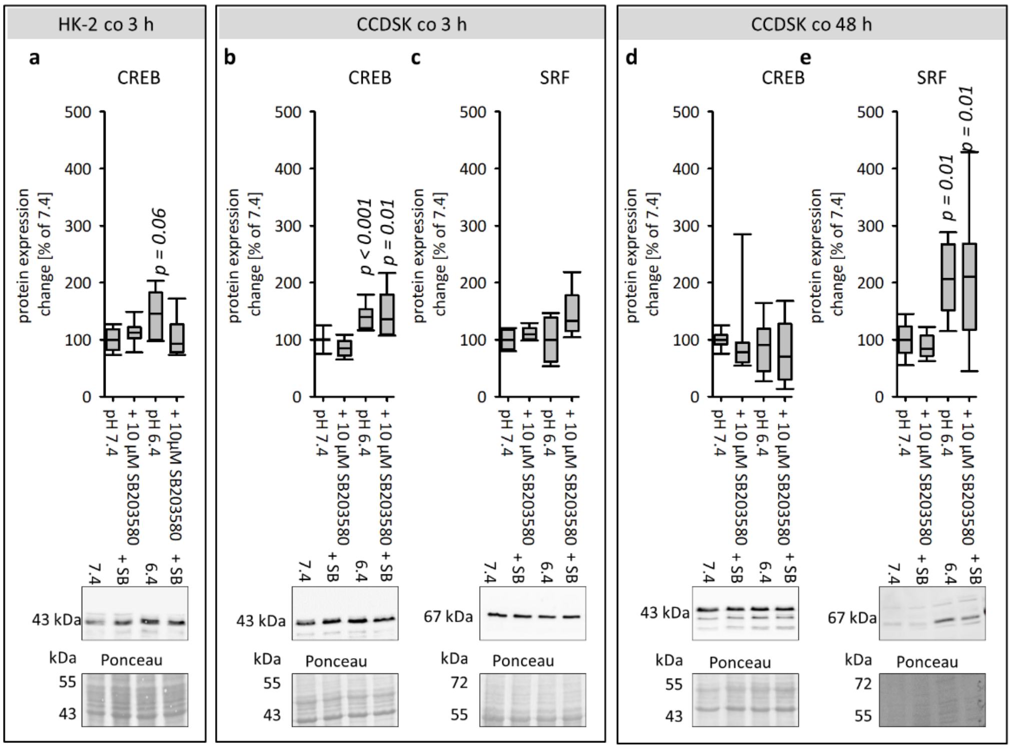

Supplementary figure 3 Significance of p38 activity for acidosis-effects. Impact of p38-inhibition (10 µM SB203580) in control or acidic media on the expression of total CREB in HK-2 after 3h (a), of total CREB and SRF in CCDSK after 3 h (b, c) and 48 h (d, e) n = 4-8, significant changes compared to pH 7.4 = $p < 0.05$.

S1.4 Significance of IL-6 receptor for acidosis-induced effects in HK-2 cells.

Figure S4 a-e shows that an inhibition of the IL-6 receptor had no impact on the acidosis-induced increase of IL-6, phospho-p38 or phospho-CREB or total p38 and CREB after 3 h in HK-2 cells.

S1.5 Significance of IL-6 receptor for acidosis-induced effects in CCDSK.

S5 a-e depicts that after 48 h the inhibition of the IL-6 receptor by tocilizumab had no impact on the acidosis effect on IL-6 or phospho- and total p38, CREB in CCDSK cells in coculture.

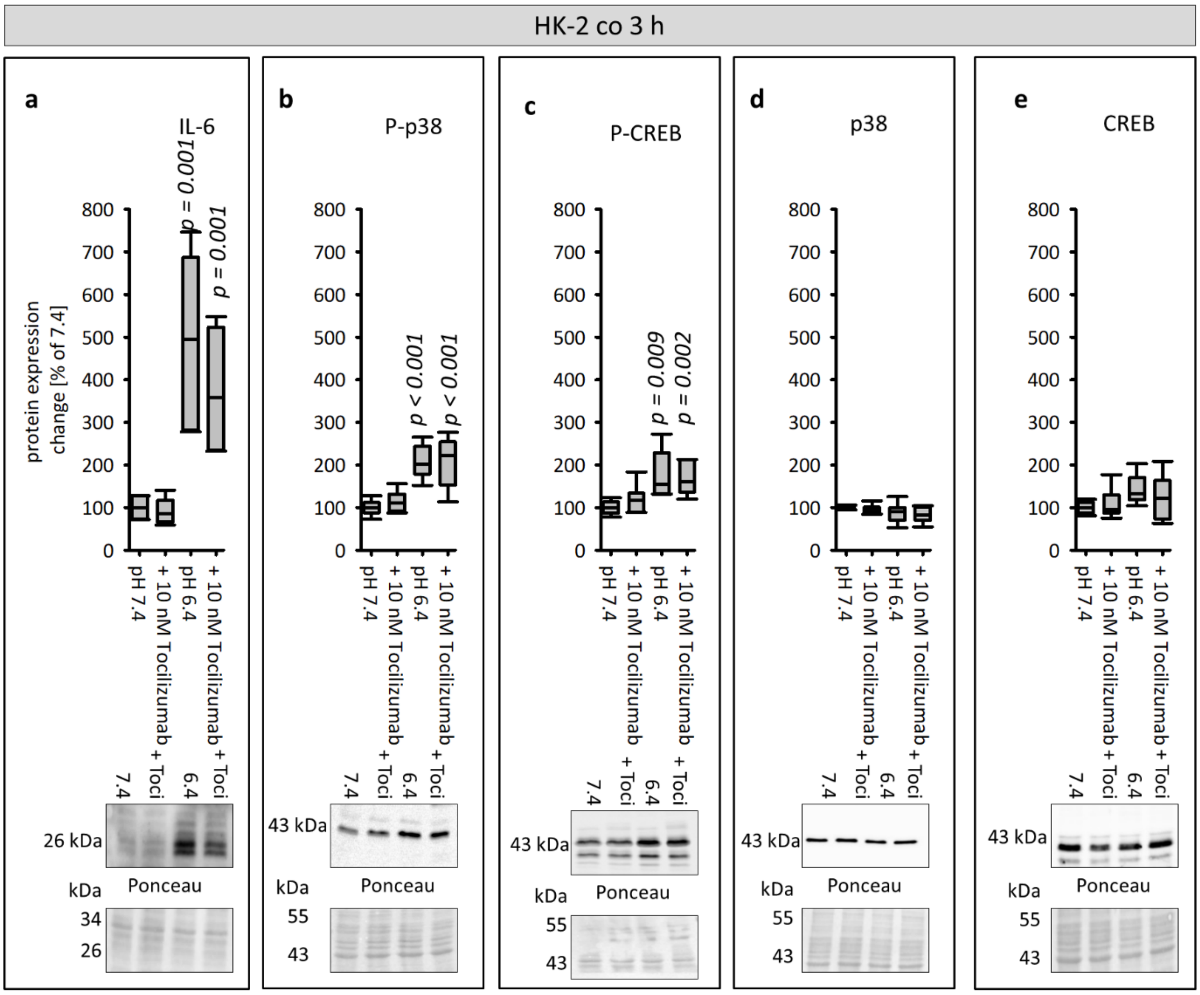

Supplementary figure 4 Significance of IL-6 for acidosis effects. Role of interleukin-6 receptor in HK-2 cells after 3 h for acidosis effects on the expression of IL-6, phospho- and total p38 and CREB after 3 h in acidic media (a-e). n = 7, significant changes compared to pH 7.4 = $p < 0.05$.

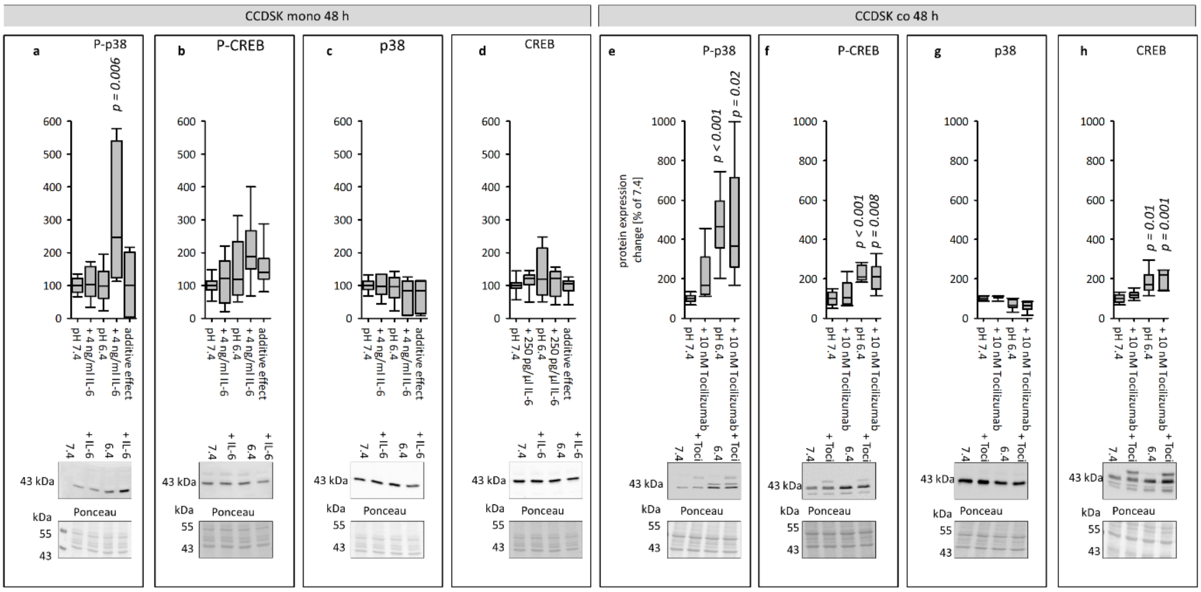

Supplementary figure 5 Significance of IL-6 for acidosis effects. Significance of IL-6 in media for the expression of phospho and total p38 or CREB in CCDSK cells in monoculture (a-d). Role of interleukin-6 receptor for acidosis effects on phospho and total p38 and CREB in CCDSK cells in coculture (e-h). n = 4-8, significant changes compared to pH 7.4 = $p < 0.05$.

Supplementary table 2 List of used antibodies, order number, host and dilutions used.

Target	Company	Order number	Host	Dilution
COX-2	Abcam, Cambridge, UK	ab15191	Rabbit	1:500
CREB	Cell Signaling, Danvers, USA	9197	Rabbit	1:1000
Phospho-CREB (Ser133)	Cell Signaling, Danvers, USA	9198	Rabbit	1:1000
cfos	Cell Signaling, Danvers, USA	2250	Rabbit	1:1000
IL-6	Cell Signaling, Danvers, USA	12153	Rabbit	1:500
IL-6Rα	Cell Signaling, Danvers, USA	18935	Rabbit	1:500
p38	Cell Signaling, Danvers, USA	9212	Rabbit	1:1000
Phospho-p38 (T180/Y182)	Cell Signaling, Danvers, USA	9211	Rabbit	1:1000
SRF	Cell Signaling, Danvers, USA	5147	Rabbit	1:1000
Phospho-SRF (Ser103)	Cell Signaling, Danvers, USA	4261	Rabbit	1:1000
Anti-Mouse IgG HRP	Cell Signaling, Danvers, USA	7076	horse	1:2000
Anti-Rabbit IgG HRP	Cell Signaling, Danvers, USA	7074	goat	1:2000

Abbreviations: COX-2 cyclooxygenase, CREB cyclic adenosine monophosphate responsive element binding protein 1, IL-6 interleukin-6, IL-6R interleukin receptor, SRF serum response factor, HRP horseradish peroxidase,

Supplementary table 3 List of buffer composition

**Buffer**	**Composition**
EDTA buffer	• 136.8 mM NaCl<br>• 2.68 mM KCl<br>• 8.1 mM Na_2HPO_4<br>• 2 mM $H_2PO_4^-$<br>• 0,7 mM EDTA<br>• pH 7,2
Equilibration buffer	• 10 mM TRIS (pH 7.3)<br>• 10 mM KCl<br>• 1.5 mM MgCl<br>• 0.5 mM ß-Mercaptoethanol
Extraction buffer	• 20 mM HEPES<br>• 0.4 M NaCl<br>• 1 mM EDTA<br>• 1 mM DTT
Lysis buffer	• equilibration buffer<br>• 0.4 % nonidet P40
1x PBS	• 1370 mM NaCl<br>• 27 mM KCl<br>• 81 mM Na_2HPO_4 * 2 H_2O<br>• 15 mM KH_2PO_4<br>• pH 7.4
RIPA buffer	• 150 mM NaCl<br>• 10 mM Tris (Base), pH 7.4<br>• 1 % (w/w) Nonidet P-40<br>• 0.1 % SDS<br>• 1 % (w/v) Na-Deoxycholate<br>• 0.1 % Triton X-100<br>• protease inhibitor cocktail<br>• 1 mM EDTA<br>• 1 mM Na-Orthovanadate<br>• 1 mM NaF
6 x Redmix/Lämmli buffer	• 124.8 mM TRIS HCl<br>• 6 % SDS<br>• 1,42 mM ß-Mercaptoethanol<br>• 4,7 mM Glycerol<br>• 155.2 mM Bromphenol Blue<br>• pH 6.8
Running buffer	• 25 mM TRIS<br>• 3,5 mM SDS<br>• 192 mM glycine
1×Turbo-Transferbuffer	• 20 % 5× Transferbuffer (Bio-Rad, Feldkirchen, Ger)<br>• 20 % ethanol (100 %)<br>• 60 % pure water
Trypsin solution	• 154 mM NaCl<br>• 2.7 mM KCl<br>• 8.2 mM Na_2HPO_4<br>• 1.5 mM KH_2PO_4

	• 0,7 mM EDTA<br>• 0,2 mM streptomycin<br>• 0,18 mM penicillin<br>• 0.02 mM trypsin<br>• pH 7,1-7,3
1xTBS TWEEN	• 3 mM TRIS base<br>• 140 mM NaCl<br>• 0.17 mM TRIS-HCl<br>• 1 % TWEEN 20<br>• pH 7.4

Supplementary table 4 Gene expression of CREB, cFos, IL-6, IL-6R, p38, COX-2 and SRF.

FPM [mean ± s.d.]	**HK-2 mono**		**HK-2 co**		**CCDSK mono**		**CCDSK co**	
	**7.4**	**6.4**	**7.4**	**6.4**	**7.4**	**6.4**	**7.4**	**6.4**
CREB1	50 ± 4	48 ± 3	46 ± 5	42 ± 6	47 ± 4	45 ± 3	37 ± 12	41 ± 15
cFos	4 ± 1	4 ± 1	5 ± 1	5 ± 1	4 ± 2	5 ± 1	3 ± 3	57 ± 72
IL6	6 ± 2	5 ± 1	6 ± 3	12 ± 4	7 ± 3	6 ± 2	14 ± 14	253 ± 459
IL6R	20 ± 4	17 ± 1	23 ± 2	20 ± 2	6 ± 1	10 ± 3	6 ± 2	39 ± 41
IL6ST (GP130)	106 ± 12	115 ± 12	90 ± 18	105 ± 22	431 ± 47	376 ± 37	359 ± 161	250 ± 67
MAPK14	74 ± 4	72 ± 2	79 ± 6	83 ± 6	68 ± 2	72 ± 3	57 ± 11	55 ± 14
PTGS2 (COX-2)	0 ± 0	0 ± 0	0 ± 0	0 ± 0	17 ± 4	7 ± 3	5 ± 5	148 ± 203
SRF	51 ± 4	46 ± 2	70 ± 5	53 ± 5	78 ± 11	92 ± 6	49 ± 11	93 ± 42
